# A Series of Potent CREBBP Bromodomain Ligands Reveals an Induced-Fit Pocket Stabilized by a Cation–π Interaction**

**DOI:** 10.1002/anie.201402750

**Published:** 2014-05-12

**Authors:** Timothy P C Rooney, Panagis Filippakopoulos, Oleg Fedorov, Sarah Picaud, Wilian A Cortopassi, Duncan A Hay, Sarah Martin, Anthony Tumber, Catherine M Rogers, Martin Philpott, Minghua Wang, Amber L Thompson, Tom D Heightman, David C Pryde, Andrew Cook, Robert S Paton, Susanne Müller, Stefan Knapp, Paul E Brennan, Stuart J Conway

**Affiliations:** Department of Chemistry, Chemistry Research Laboratory, University of Oxford Mansfield Road, Oxford, OX1 3TA (UK); Nuffield Department of Clinical Medicine, Structural Genomics Consortium, University of Oxford Old Road Campus Research Building, Roosevelt Drive, Oxford, OX3 7DQ (UK); Neusentis, The Portway Building Granta Park, Great Abington, Cambridge, CB21 6GS (UK)

**Keywords:** bromodomain, CREBBP, enzyme inhibitors, epigenetics, ligand discovery

## Abstract

The benzoxazinone and dihydroquinoxalinone fragments were employed as novel acetyl lysine mimics in the development of CREBBP bromodomain ligands. While the benzoxazinone series showed low affinity for the CREBBP bromodomain, expansion of the dihydroquinoxalinone series resulted in the first potent inhibitors of a bromodomain outside the BET family. Structural and computational studies reveal that an internal hydrogen bond stabilizes the protein-bound conformation of the dihydroquinoxalinone series. The side chain of this series binds in an induced-fit pocket forming a cation–π interaction with R1173 of CREBBP. The most potent compound inhibits binding of CREBBP to chromatin in U2OS cells.

Small molecules that modulate a disease phenotype in preclinical assays are important tools in the validation of putative therapeutic targets.[1] Such compounds have proved invaluable in elucidating the biology of the acetyl-lysine (KAc)-binding bromodomain and extra C-terminal domain (BET) family of bromodomain-containing proteins (BCPs), which have emerged as important therapeutic targets for cancer and inflammation.[2–7] To date there are few potent bromodomain ligands with nanomolar binding affinity for BCPs that are not part of the BET family. Although ligands for the cAMP response element binding protein (CREB) binding protein (CREBBP, Figure 1 A) bromodomain have been previously identified, they have relatively low binding affinities or selectivities.[7–12] Here we report the development of a dihydroquinoxalinone-based series of compounds, (*R*)-**1** and (*R*)-**2** (Figure 1 B), that show nanomolar affinities for the CREBBP bromodomain.

**Figure 1:**
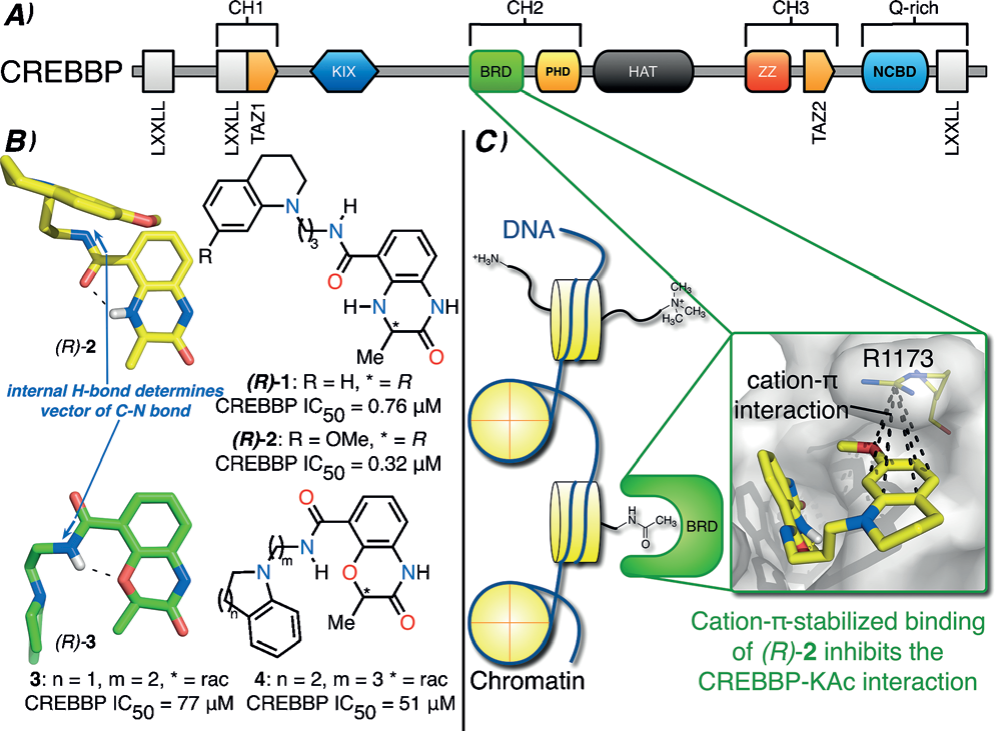
A) The domain architecture of CREBBP.[13] B) The internal hydrogen bonds of the benzoxazinone and dihydroquinoxalinone series determine the vector of the C–N bond. C) The dihydroquinoxalinone ligands, (*R*)-**1** and (*R*)-**2**, bind to the CREBBP BRD in an induced-fit pocket, forming a cation–π interaction with R1173.

Using an AlphaScreen assay we identified *N*-methyl-2-pyrrolidone (NMP) as a weak (IC_50_=1.9 mm) but ligand-efficient (0.54) CREBBP bromodomain ligand.[14, 15] Further screening of NMP analogues revealed that 3,4-dihydro-3-methyl-2(1*H*)-quinazolinone also binds to the CREBBP bromodomain, mimics the interactions made by KAc, and forms an additional hydrogen bond between the NH of the dihydroquinazolinone ring and the carbonyl oxygen atom of N1168 in CREBBP (Figure S1A in the Supporting Information). This fragment was employed in the development of BET bromodomain ligands by Chung et al.[16] and Fish et al.[5] However, our previous work[15, 17] revealed that this dihydroquinazolinone is susceptible to oxidation at the benzylic position, and consequently we sought to develop a KAc mimic that addressed this problem. We initially investigated the benzoxazinone moiety (**3**, **4**, Figure 1 B), in which an oxygen atom replaces the oxidation-prone methylene group. Synthesis and biological evaluation of benzoxazinone-based fragments indicated that 8-position substitution was tolerated (Table S1). Additionally, the high-resolution X-ray crystal structure of dihydroquinazolinone bound to the CREBBP bromodomain indicated that modification at this position would direct substituents into the ZA channel (Figure S1B). However, at that time we were unaware that this channel is permanently occupied by water molecules. Ranking of the results from an in silico screen of approximately 6000 commercially available amines, conjugated to an 8-position carboxylic acid on the benzoxazinone moiety, identified 50 target compounds. Parallel synthesis and biological evaluation of these compounds (AlphaScreen) showed the most active compounds **3** and **4** as having IC_50_ values of 77 μm and 51 μm, respectively, against CREBBP (Table S2).

The low affinity of these compounds for the CREBBP bromodomain led us to redesign the KAc mimic giving consideration to two key points. Firstly, the X-ray crystal structure of dihydroquinazolinone bound to the CREBBP bromodomain indicated that the benzoxazinone oxygen atom would be placed ca. 3.5 Å from the backbone carbonyl group of P1110, potentially causing an unfavorable interaction (Figure S1A). To overcome this problem, we employed the dihydroquinoxalinone group as the KAc mimic [(*R*)-**1**, (*R*)-**2**; Figure 1 B]. In this group, the oxygen atom is replaced with a secondary amine, and hence any unfavorable interaction with P1110 will be ameliorated. Secondly, a structure of unbound (*R*)-**3** determined using high-resolution single-crystal X-ray diffraction data confirmed that an internal hydrogen bond [NH⋅⋅⋅O 2.696(2) Å] is formed between the amide and the benzoxazinone (Figure 1 B and Figure S2). Therefore, a consequence of the change to the KAc mimic was the altered nature of the internal hydrogen bond that exists in both of these molecules. Both enantiomers of compound **1**, with the tetrahydroquinoline side chain from **4** attached to the dihydroquinoxalinone KAc mimic, were synthesized (Scheme S1 and S4). Pleasingly, compound (*R*)-**1** showed an increase in CREBBP potency, with IC_50_=758 nm (Table 1).

**Table 1:** AlphaScreen data for compounds 1, 2, and 5–8

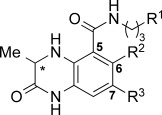
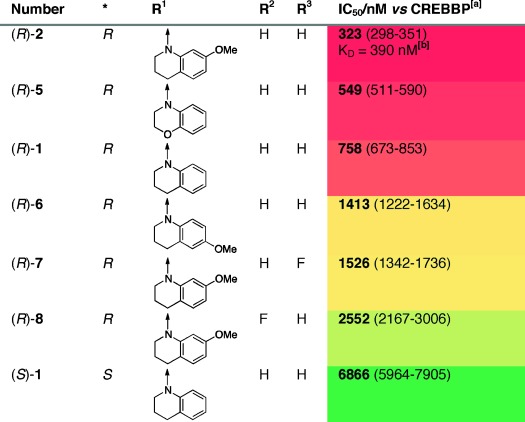

[a] Peptide and protein concentration=25 nm. [b] Isothermal titration calorimetry.

An X-ray crystal structure of (*R*)-**1** bound to the CREBBP bromodomain revealed that the dihydroquinoxalinone was acting as the KAc mimic (Figure 2 A). The amide of the dihydroquinoxalinone formed two hydrogen bonds with N1168, and the methyl group resided at the base of the KAc-binding pocket (Figure 2 A).

**Figure 2:**
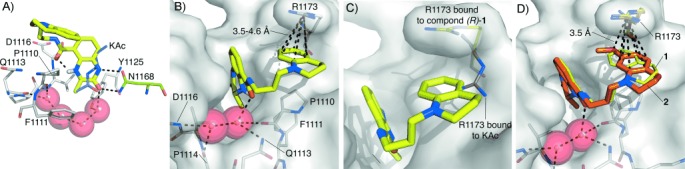
A) Overlaid X-ray crystal structures of compound (*R*)-**1** (PDB code 4NYW, carbon: yellow) and KAc (PDB code 3P1C, carbon: purple)[18] both bound to the CREBBP bromodomain. The dihydroquinoxalinone moiety forms two hydrogen bonds with N1168 and a water-mediated hydrogen bond with Y1125. B) The carbonyl group of (*R*)-**1** forms a hydrogen bond with one of the conserved ZA channel water molecules. This contact, coupled with the internal hydrogen bond, directs the tetrahydroquinoline side chain into an induced-fit pocket under R1173 that is stabilized by a cation–π interaction. C) R1173 alters its conformation in the KAc-bound structure (PDB code 3P1C, carbon: gray)[18] to accommodate the tetrahydroquinoline side chain of (*R*)-**1**. D) The side chain of (*R*)-**2** (PDB code 4NYX, carbon: orange) moves relative to that of (*R*)-**1** (PDB code 4NYW, carbon: yellow) maximizing the interaction between the positively charged R1173 and the electron-donating methoxy group.

The internal hydrogen bond between the dihydroquinoxalinone and the amide is evident from the conformation of the ligand (Figure 2 A). The amide carbonyl oxygen also interacts with the bottom ZA channel water molecule. These two interactions orient the tetrahydroquinoline moiety away from the water-occupied ZA channel into an induced-fit pocket below R1173 (Figure 2 B). This pocket was not evident in previous CREBBP bromodomain structures (Figure 2 C) and hence this interaction was impossible to predict in our initial docking studies. The 3.5–4.6 Å distance between the tetrahydroquinoline ring and R1173 (Figure 2 B) suggests that ligand binding is stabilized by a cation–π interaction.[19] To investigate this hypothesis, and potentially optimize the interaction, we synthesized a range of tetrahydroquinoline derivatives that possessed electron-donating groups on the aromatic ring (Scheme S4 and S11). AlphaScreen data revealed that (*R*)-**2**, which has a methoxy group at the 7-position of the tetrahydroquinoline ring, showed enhanced CREBBP bromodomain affinity, with IC_50_=323 nm and *K*_D_=390 nm (isothermal titration calorimetry, ITC).

(*R*)-**2** has modest selectivity over BRD4(1) [*K*_D_=1.4 μm (ITC)] and good selectivity over seven other phylogentically diverse BCPs (Figure S4). Compound (*R*)-**5**, which has a 3,4-dihydro-2*H*-benzo[*b*][1,4]oxazine side chain, also displays enhanced CREBBP affinity, whereas the two fluorine-containing derivatives (*R*)-**7** and (*R*)-**8** as well as (*S*)-**1** were less potent (Table 1). Single-crystal X-ray diffraction studies confirmed that the dihydroquinoxalinone moieties of (*R*)-**2** and (*R*)-**1** bind in identical positions. However, the side chain of (*R*)-**2** has moved relative to the position of (*R*)-**1**, allowing interaction of the methoxy group oxygen atom with the positively charged R1173 (Figure 2 D). Compound (*R*)-**6**, with the methoxy group in the 6-position of the tetrahydroquinoline, shows lower CREBBP affinity than (*R*)-**1**, suggesting that the methoxy group in this position cannot interact with R1173 without experiencing a steric clash with the protein.

To confirm that the crystallographically observed cation–π interaction is an important component of ligand binding in the solution phase we undertook molecular dynamics (MD) simulations. In an aqueous environment, the interaction between R1173 and the aromatic ring of (*R*)-**2** was observed to form spontaneously during the simulation (0.1 μs, Figure S6). Using a cut-off distance of 6 Å, as proposed by Dougherty,[19] the cation–π interaction was maintained for approximately 40 % of the trajectory time. Using counterpoise-corrected dispersion corrected DFT calculations we estimated the strength of the cation–π interaction to be in the region of 3.2–4.7 kcal mol^−1^ (Figure S8). These calculated values agree well with the experimentally measured average strengths of cation–π interactions involving either Lys (−3.3±1.5 kcal mol^−1^) or Arg (−2.9±1.4 kcal mol^−1^) (see Supporting Information).[19] Hence the cation–π interaction appears to be of relevance to the protein–ligand binding in solvent. This observation is consistent with literature reports in which optimization of cation–π interactions between ligands and proteins has resulted in enhanced binding affinities.[20–23] The MD simulations also identified that the hydrogen bonds formed between (*R*)-**2** and N1168 and dispersive interactions between V1174 and the ring face of the KAc mimic inside the binding pocket contribute to the binding energy (Figure S6). The combination of AlphaScreen data, structural information and MD studies demonstrate that the tetrahydroquinoline ring is forming a cation–π interaction with R1173 through binding in an induced-fit pocket. It seems that this interaction might be important for ligands to show high affinity for the CREBBP bromodomain.

To explore the significance of the internal hydrogen bond in pre-organizing the ligand conformation for bromodomain binding, we performed DFT (wB97XD/TZVP) calculations on simplified model compounds (*R*)-**9** and (*R*)-**10** (Figure 3 and Table S6). These calculations indicate that benzoxazinone-derived (*R*)-**9** will adopt a single conformer at room temperature stabilized by the internal hydrogen bond (Figure 3 A). The DFT-calculated conformation (C_carbonyl_–C_aryl_ dihedral angle *θ*=210°) is similar to the conformation adopted by the high-resolution crystal structure [*θ*=186.4 (2)°]. In the dihydroquinoxalinone, the internal hydrogen bond forms between the NH of the dihydroquinoxalinone and the amide carbonyl oxygen atom, reversing the sense of the internal hydrogen bond and consequently changing the vector of the amide substituent (Figure 3 C). DFT calculations indicate that two of the lowest energy conformations adopted by (*R*)-**10** (*θ*=30° and 330°) possess the internal hydrogen bond (Figure 3 B). When bound to CREBBP (*R*)-**2** adopts a conformation that is intermediate between these two (*θ*=2°). An MD simulation (250 ns) in explicit water indicated that the values observed for the O-C-C-C dihedral angle are fully consistent with the DFT computed energy profile, with the value maintained close to the global energy minimum due to the existence of an intramolecular hydrogen bond (Figure S7). This reinforces the idea that this H-bond plays a key role in determining the solution-phase conformation of (*R*)-**10**. Therefore, while the internal hydrogen bond in (*R*)-**1** and (*R*)-**2** orients the tetrahydroquinoline towards R1173 and the induced-fit pocket, the internal hydrogen bond in (*R*)-**3** directs the side chain in the opposite direction. This observation explains the low affinity of the benzoxazinone series for the CREBBP bromodomain, as orienting the side chain in this direction would result in a clash with the protein and ZA channel waters and low binding affinity. To rotate the C_carbonyl_–C_aryl_ bond away from the optimal 186° observed in compound (*R*)-**3** and avoid this clash, the internal hydrogen bond must be broken, resulting in a low affinity CREBBP bromodomain ligand.

**Figure 3:**
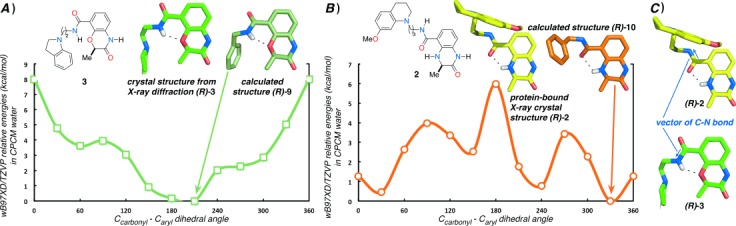
A) A plot of CPCM-wB97XD/TZVP relative energetics (kcal mol^−1^) in implicit water for rotation about the C_carbonyl_–C_aryl_ bond of the model compounds (*R*)-**9** (green) and (*R*)-**10** (orange). Compound (*R*)-**9** adopts a single lowest energy conformation, stabilized by an internal hydrogen bond between the O atom of the benzoxazinone ring and the amide NH. This conformation is similar to that observed in the small-molecule X-ray crystal structure of (*R*)-**3**. B) The two lowest energy conformations of (*R*)-**10** are stabilized by internal hydrogen bonds between the NH of the dihydroquinoxalinone and the amide O atom. These conformations are similar to that observed in the CREBBP bromodomain-bound X-ray crystal structure of (*R*)-**2**. C) The reversed internal hydrogen bond changes the amide bond vector in benzoxazinone and dihydroquinoxalinone analogues (blue arrows).

To determine the utility of (*R*)-**2** in a cellular setting a FRAP assay was conducted. Data from this assay demonstrated that (*R*)-**2** can displace the CREBBP bromodomain from chromatin (Figure 4 and S9) in U2OS cells in a dose-dependent manner. Measurement of the time taken for fluorescence recovery, in a bleached area of cells expressing GFP chimerized to multimerized CREBBP bromodomains, shows that *t*_1/2_ is dose-dependently reduced in the presence of (*R*)-**2** compared to wild-type cells, in the presence of suberanilohydroxamic acid (SAHA/Vorinostat) (2.5 μm). The *t*_1/2_ in the presence of 5 and 10 μm (*R*)-**2** is similar to the N1168F mutant, which shows reduced binding to KAc. These data indicate that (*R*)-**2** interacts with the KAc binding site of the CREBBP bromodomain to displace it from chromatin.

**Figure 4:**
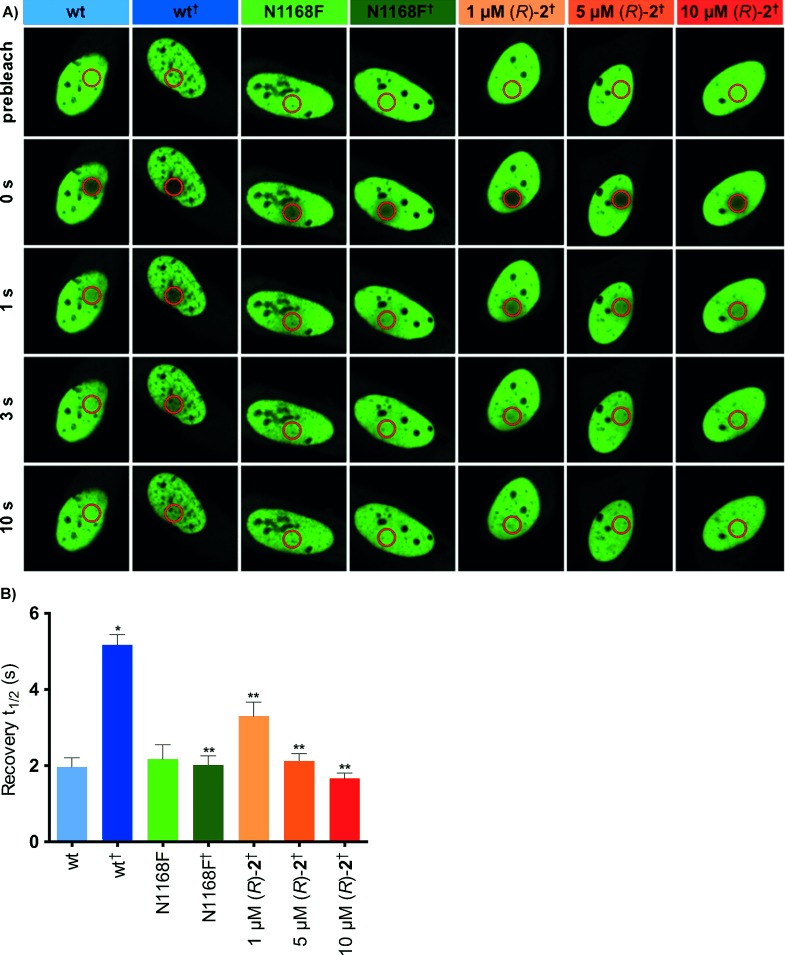
A) Fluorescence recovery after photobleaching (FRAP) demonstrating compound (*R*)-**2** can displace the CREBBP bromodomain from chromatin. Nuclei of U2OS cells transfected with plasmids encoding GFP chimerized to wild-type (wt) or mutant (N1168F) multimerized CREBBP bromodomain, with or without 2.5 µm SAHA (indicated by †) and the inhibitor (*R*)-**2** at the indicated concentrations. The bleached area is indicated by a red circle. B) Half times of fluorescence recovery (*t*_1/2_) of U2OS cells expressing green fluorescent protein (GFP) chimerized to multimerized CREBBP bromodomains with or without 2.5 μm SAHA (indicated by †). Bars represent the mean *t*_1/2_ calculated from individual recovery curves of 15 cells per group. * significantly different from wt, *p*<0.0001. ** significantly different from wt†, *p*<0.0001.

In summary, we have developed a series of dihydroquinoxalinone-derived CREBBP bromodomain ligands. MD and DFT calculations indicate that an internal hydrogen bond helps to preorganize the solution phase conformation of (*R*)-**2** in a manner that is favorable for CREBBP bromodomain binding, resulting in high-affinity ligands. The tetrahydroquinoline side chain of these compounds binds in a previously unknown induced-fit pocket, and this interaction is stabilized by a cation–π interaction. MD simulations and DFT calculations indicate that this cation–π interaction forms spontaneously in water and contributes to the ligand’s affinity for the CREBBP bromodomain. Compound (*R*)-**2** inhibits binding of the CREBBP bromodomain to chromatin in U2OS cells. These compounds demonstrate that it is possible to develop potent “drug-like” ligands (Table S7) for non-BET bromodomains. They will be invaluable tools to validate inhibition of the CREBBP bromodomain as a therapeutic target in diseases including leukemias, Rubinstein–Taybi syndrome, ovarian, breast and lung cancers and systemic lupus erythematosus.

## Supporting Information

As a service to our authors and readers, this journal provides supporting information supplied by the authors. Such materials are peer reviewed and may be re-organized for online delivery, but are not copy-edited or typeset. Technical support issues arising from supporting information (other than missing files) should be addressed to the authors.

miscellaneous_information
