# Supplementary material for: A Series of Potent CREBBP Bromodomain Ligands Reveals an Induced-Fit Pocket Stabilized by a Cation–π Interaction
Source: Angew Chem Int Ed Engl. 2014 May 12;53(24):6126–30. doi: 10.1002/anie.201402750 (PMC4298791; doi:10.1002/anie.201402750)

Angewandte Chemie
Eine Zeitschrift der Gesellschaft Deutscher Chemiker

Supporting Information

© Wiley-VCH 2014

69451 Weinheim, Germany

A Series of Potent CREBBP Bromodomain Ligands Reveals an Induced-Fit Pocket Stabilized by a Cation–π Interaction**

*Timothy P. C. Rooney, Panagis Filippakopoulos, Oleg Fedorov, Sarah Picaud, Wilian A. Cortopassi, Duncan A. Hay, Sarah Martin, Anthony Tumber, Catherine M. Rogers, Martin Philpott, Minghua Wang, Amber L. Thompson, Tom D. Heightman, David C. Pryde, Andrew Cook, Robert S. Paton, Susanne Müller, Stefan Knapp, Paul E. Brennan, and Stuart J. Conway**

anie_201402750_sm_miscellaneous_information.pdf

Table of Contents

Figure S1: Comparison of protein X-ray crystal structures with predicted binding modes. S3

Figure S2: Crystal structure from X-ray diffraction of (*R*)-**3**. S4

Figure S3: ITC data for (*R*)-**2**. S5

Figure S4: Selectivity data for (*R*)-**2**. S6

Figure S5: MD data for CREBBP binding (*R*)-**2**. S7

Figure S6: MD data for interactions between CREBBP and (*R*)-**2**. S8

Figure S7: MD data for intramolecular H-bond in dihydroquinoxalinone amides. S9

Figure S8: Quantification of the cation-π interaction.. S9

Figure S9: FRAP data for (*R*)-**2**. S10

Scheme S1: Synthesis of dihydroquinoxalinone carboxylic acid. S11

Scheme S2: Synthesis of 6-fluoro-dihydroquinoxalinone derivatives. S11

Scheme S3: Synthesis of 7-fluoro-dihydroquinoxalinone derivatives. S12

Scheme S4: Synthesis of dihydroquinoxalinone amides. S12

Scheme S5: Synthesis of benzoxazinone fragments. S13

Scheme S6: Synthesis of benzoxazinone amide analogues. S13

Scheme S7. Synthesis of single enantiomer benzoxazinone amides. S13

Scheme S8. Synthesis of DHQX-carboxylic acid from 2-hydroxy-3-nitrobenzoic acid. S14

Scheme S9: Synthesis of DHQX-carboxylic acid from methyl 3-nitro-2-hydroxybenzoate. S14

Scheme S10: Synthesis of 7-methoxy-1,2,3,4-tetrahydroquinoline. S15

Scheme S11: Synthesis of amine coupling partners. S15

Table S1: AphaScreen data for fragment benzoxazinone-based CREBBP bromodomain inhibitors. S16

Table S2: AphaScreen data for inhibition of CREBBP by 8-amido substituted benzoxazinones. S17

Table S3: Protein crystallization data collection and refinement statistics. S18

Table S4: Coordinates for ligand in MD simulation. S19

Table S5: Coordinates for ligand in QM calculation. S20

Table S6: DFT calculation results. S21

Table S7: Calculated ligand properties and ligand efficiency. S22

Computational Methods. S23

Physical Methods. S25

Biochemical Methods. S25

Synthetic Methods. S27

General Procedures. S30

Synthesis and Characterisation of Compounds. S32
References. S67
1H and 13C spectra. S70

Supporting Figure S1: Comparison of protein X-ray crystal structures with predicted binding modes.

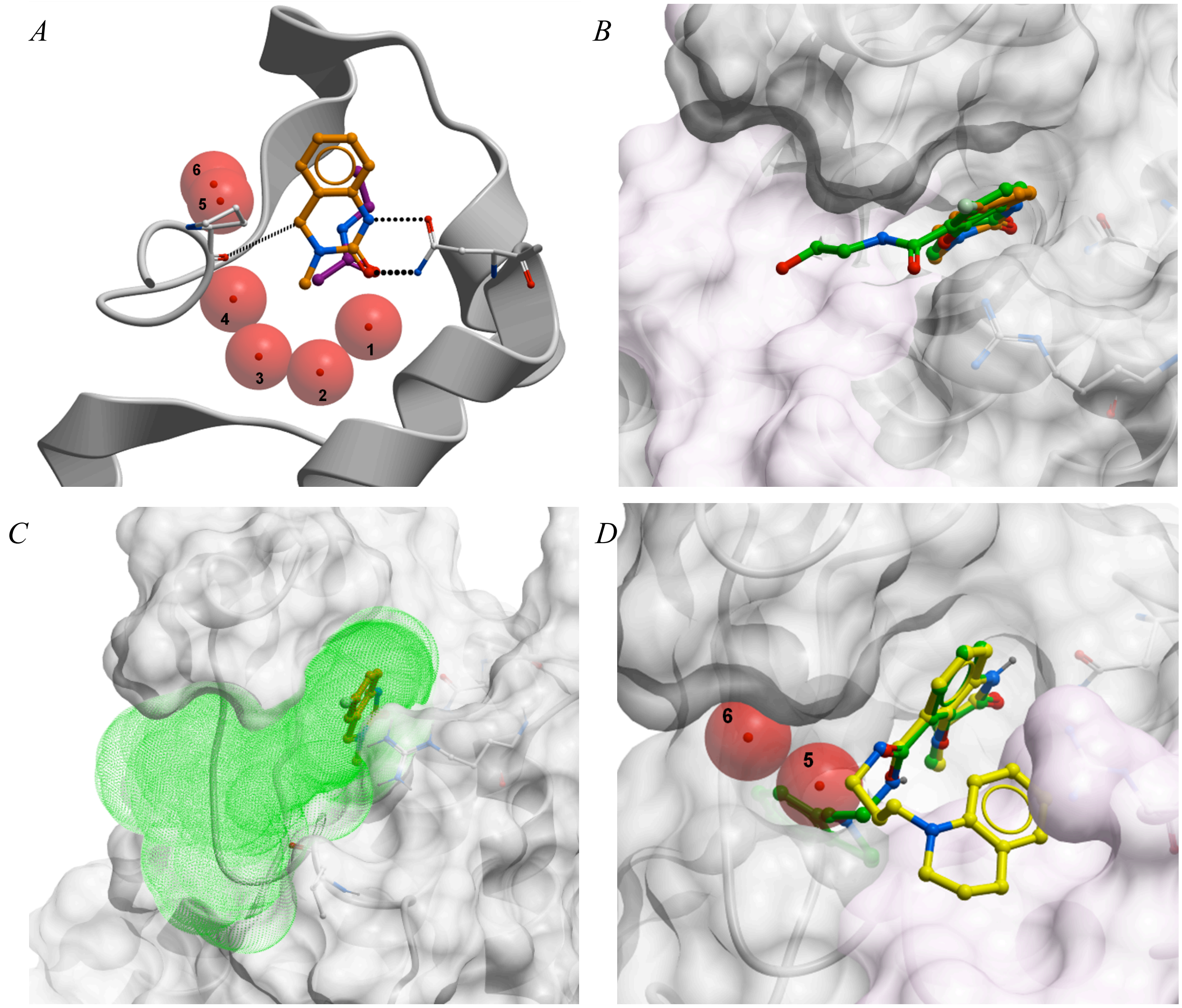

A: Overlayed X-ray crystal structures of KAc (PDB code 3P1C, carbon = purple) and DHQ (PDB code 3P1F, carbon = orange) both bound to the CREBBP bromodomain. Hydrogen bonds to N1168 are shown. Also shown is the distance between the benzylic position and backbone carbonyl group of P1110, measured as 3.5 Å. This has the potential for a lone pair clash when the benzylic carbon is replaced with oxygen, or a hydrogen bond when replaced with NH. **B**: Overlay of the crystal structure of 6-bromo-DHQ (PDB ID: 4NVY, carbon = orange) and the predicted binding mode of a benzoxazinone (carbon = green) bound to CREBBP. The ZA loop region, which 8-position substituents are directed towards, is shaded pink. **C**: Crystal structure of 6-bromo-DHQ (PDB ID: 4NYV, carbon = orange) with the predicted occupancy of the ZA loop by docked benzoxazinones substituted at the 8-position (green dot representation). **D**: Comparison of the protein bound X-ray crystal structure of DHQX (*R*)-**1** (PDB ID = 4NYW, carbon = yellow) with the small molecule crystal structure of BNZ (*R*)-**3** (carbon = green). Whilst DHQX substituents are directed towards the induced-fit pocked (shaded pink), BZN analogues clash with the ZA channel and water molecule 5.

Supporting Figure S2: Crystal structure from X-ray diffraction of (*R*)-**3**.

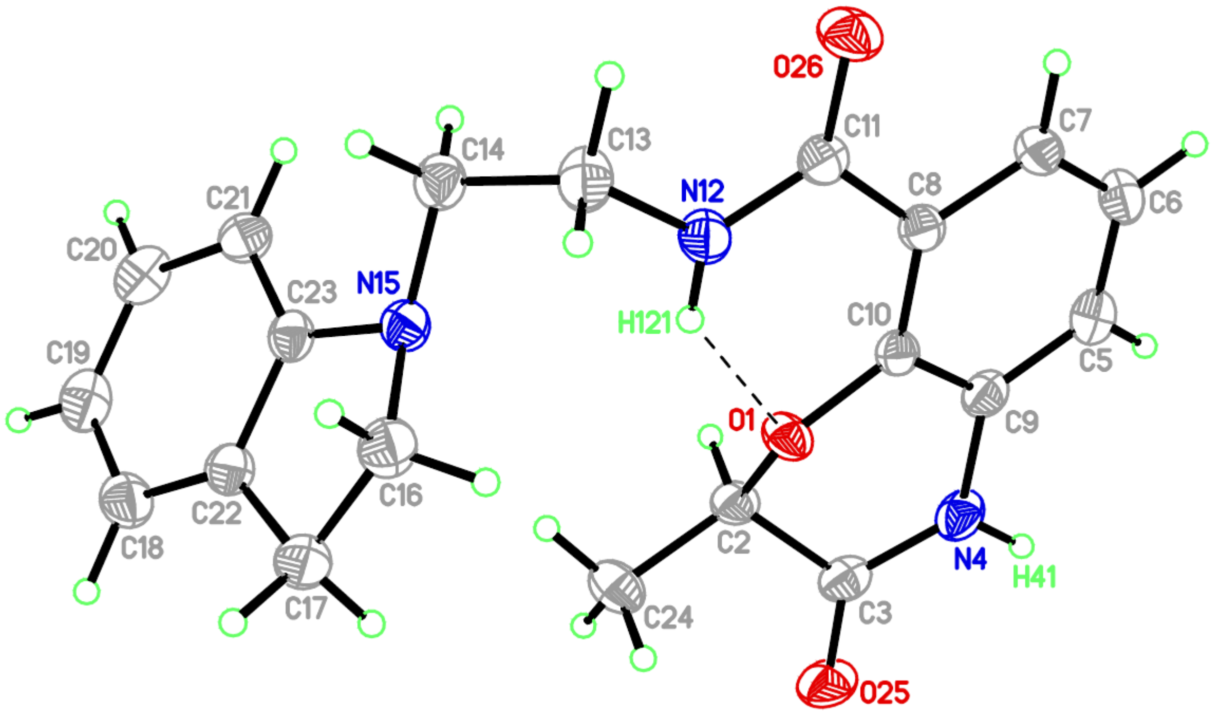

Grey = carbon, blue = nitrogen, red = oxygen and green = hydrogen. The intramolecular H-bond between H121 and O1 is shown as a dotted line.

X-ray crystal structure data for (*R*)-**3** [$C_{20}H_{21}N_3O_3$]: M = 351.40, monoclinic, space group $P\ 2_1$, a = 7.5117(2) Å, b = 5.7867(1) Å, c = 19.8673(5) Å, α = 90º, β = 100.241(2)º, γ = 90º, V = 849.83(3) Å^3, Z = 2, μ = 0.763 mm^{-1}, colorless plate, crystal dimensions = 0.18 × 0.08 × 0.01 mm^3. A total of 3549 unique reflections were measured and all were used in the refinement. The final parameters were wR_2 = 0.0635 and R_1 = 0.0247 [I>2σ(I)]. The Flack x parameter[1,2] was refined to a value of 0.03(12)[3] which is consistent with the stereochemistry of the crystallographic model.

Supporting Figure S3: ITC data for (*R*)-**2** against CREBBP (left) and BRD4 (right).

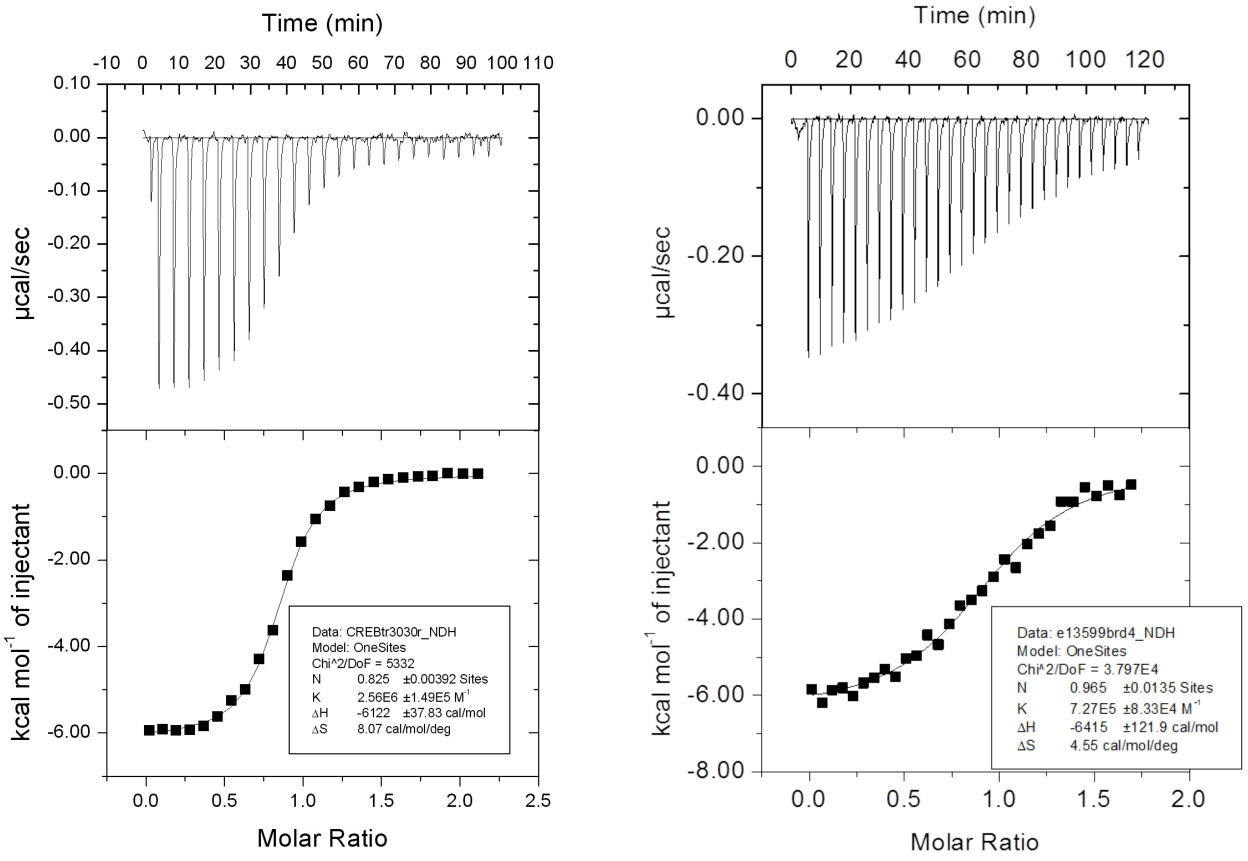

$K_D = 0.391 \pm 0.023$ µM

$\Delta H = -6.12 \pm 0.04$ kcal/mol

$T\Delta S = -2.40$ kcal/mol

$K_D = 1.38 \pm 0.16$ µM

$\Delta H = -6.42 \pm 0.12$ kcal/mol

$-T\Delta S = -1.36$ kcal/mol

* Errors in K_D and ΔH represent error in fitting of data.

Supporting Figure S4: Selectivity data for (*R*)-**2**.

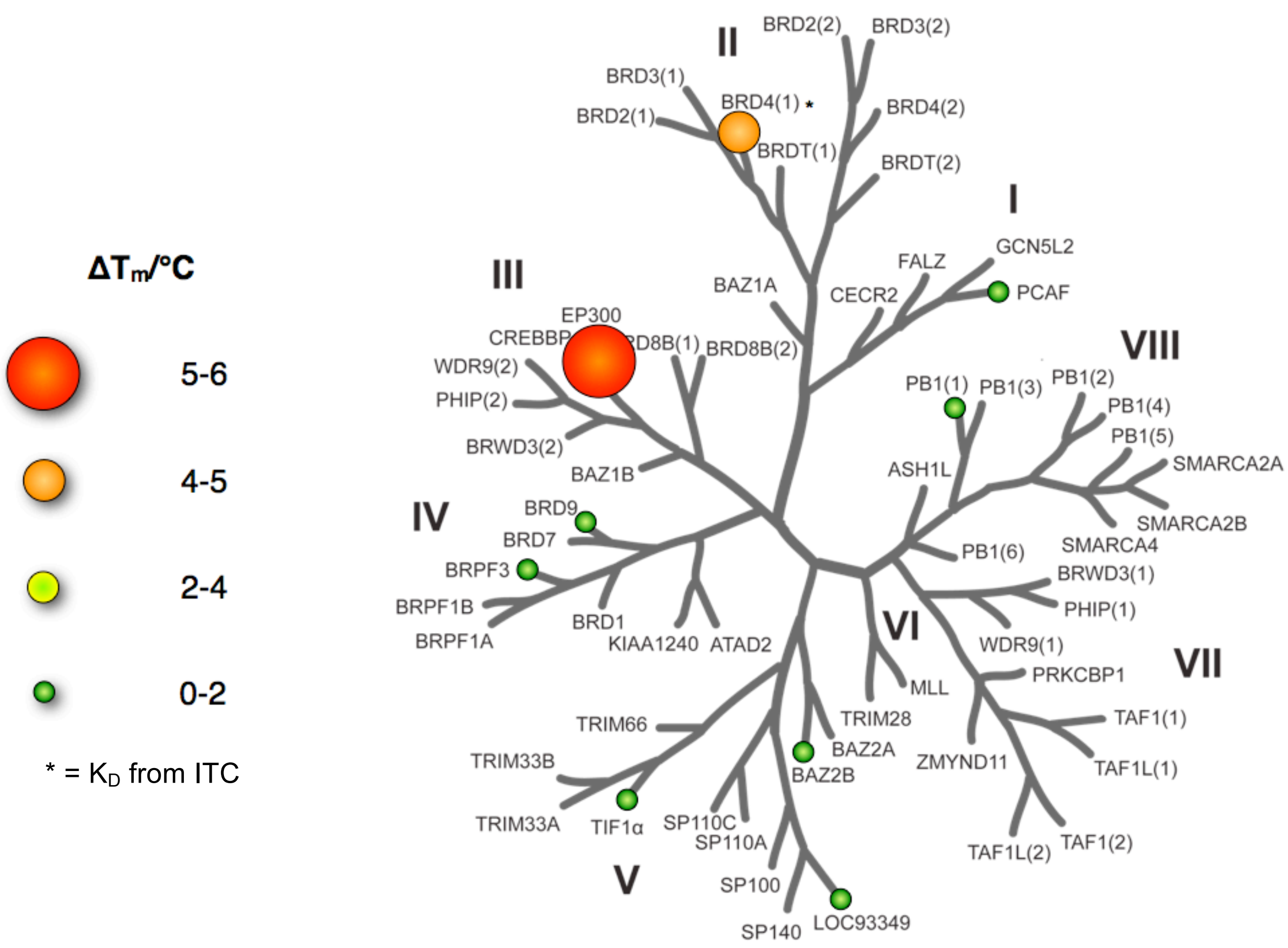

	CREBBP	BAZ2B	BRD9	BRPF3	LOC93349A	PB1(1)	PCAF	TIF1α
ΔTm/°C	5.4	-0.5	-1.1	0.5	0.3	-0.1	0.0	0.7

	CREBBP	BRD4(1)
K_D (ITC)/μM	0.391	1.38

Selectivity evaluation of (*R*)-**2** as determined by Differential Scanning Fluorimetry (ΔT_m) and Isothermal Titration Calorimetry (K_D) against a panel of different bromodomains. Heat map shows relative ΔTm or K_D values (red indicates most potent and green indicates least), also plotted onto the bromodomain phylogenetic tree.

Supporting Figure S5: MD data for CREBBP binding (*R*)-**2**.

A

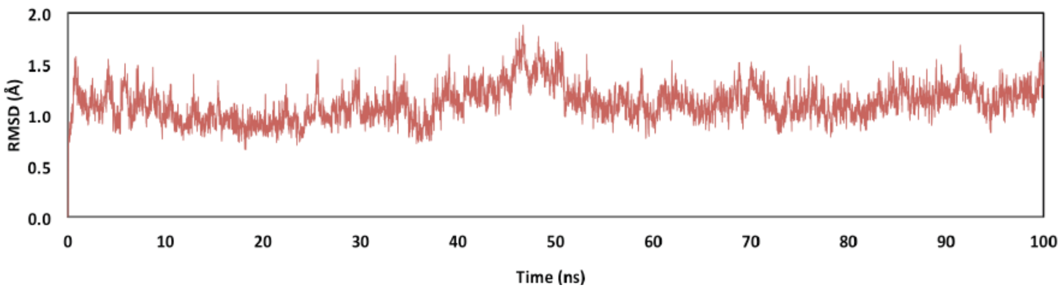

A: Temporal RMSD for Cα atoms from the starting structure of the MD calculations. The average RMSD was observed to be stabilized at around 1.2 Å during the simulation, while temperature, volume and water density (data not shown) were almost constant around 310 K, 53 000 $Å^3$ and 1.1 g cm^{-3}, respectively, during all the 100 ns of the simulation.

B

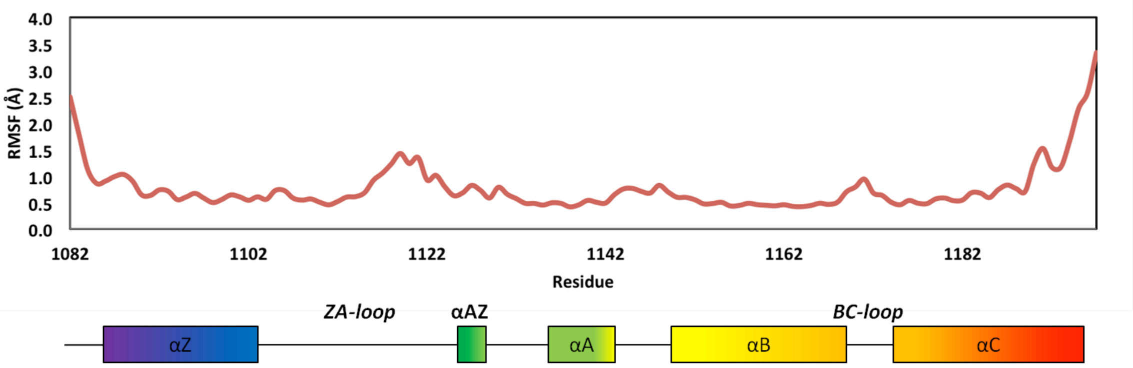

B: Per-residue Root Mean Square Fluctuations (RMSF) of the Cα atoms of the protein. The highest fluctuations of the residues were observed for the ZA-loop, BC-loop, and termini, as has been discovered for the CREBBP/acetyl-lysine complex during repeated µs MD simulation in explicit solvent.[4]

Supporting Figure S6: MD data for interactions between CREBBP and (*R*)-**2**.

A

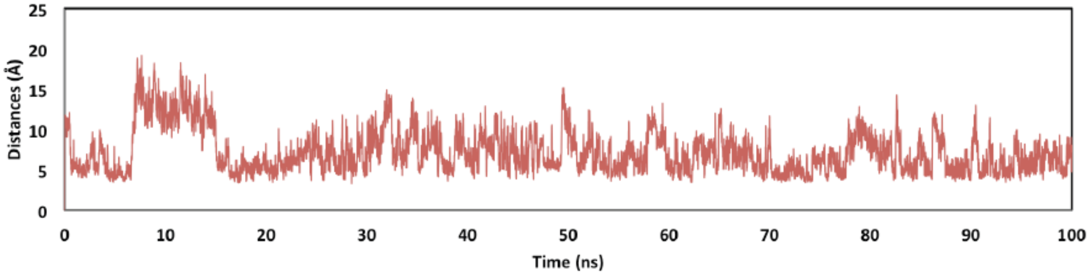

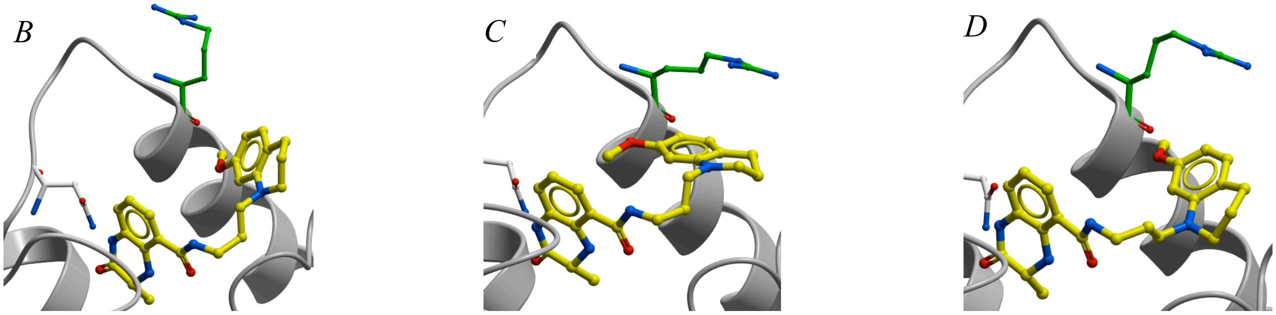

A: Distance (Å) between the guanidium C atom in R1173 and the center of mass of the aromatic ring of (*R*)-**2**. At the start of the MD trajectory in explicit water R1173 is oriented away from the ligand into bulk solvent such that the distance between the THQ center of mass and the guanidinium central C atom is around 12 Å (**B**). However, during the course of the simulation the cation-π interaction is observed to form. Applying a distance cut-off of 6 Å, as proposed by Dougherty,[5] we find that this interaction is present during more than 40% of the simulation time, suggesting that this interaction is indeed present in the solution phase. We also evaluated the conformation of the guanidium in the presence of the inhibitor, as proposed by Kaznessis.[6] An angle between the two planes of 0-45° and 135-180° was considered in the parallel conformation (**C**). In the T-shaped conformation (**D**), the two planes are perpendicular with an angle between 45° and 135°. During the 100 ns of the MD it is possible to observe this interaction is predominantly in the parallel conformation (approximately 70% of the simulation), as observed in the crystal structure.

E

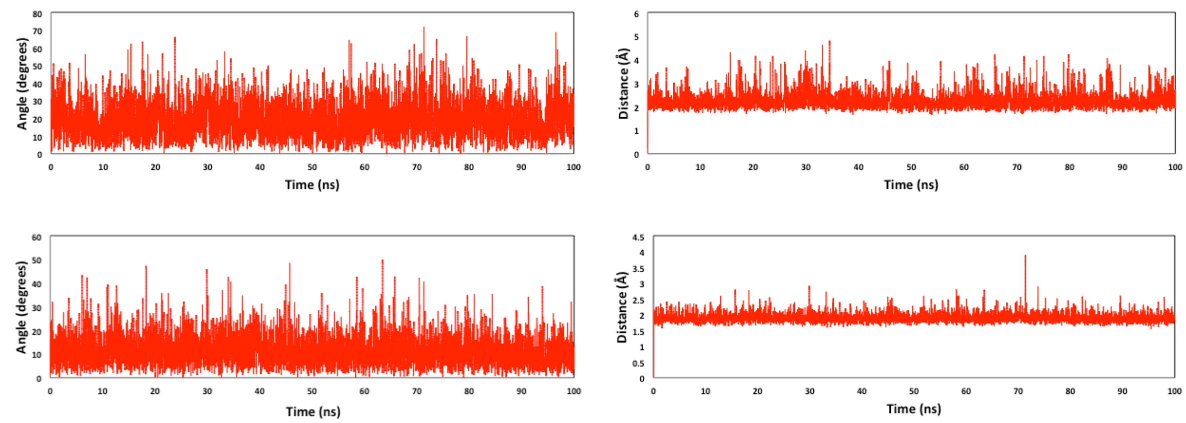

E: Distance (Å) between the –NH hydrogen atom of the ligand and the O atom of Asn1168 (top), and between the –NH hydrogen atom of N1168 and the O atom of the ligand (bottom). Both hydrogen bonds were largely maintained during the simulation length indicative of a relatively strong intermolecular interaction energy.

Supporting Figure S7: MD data for intramolecular H-bond in dihydroquinoxalinone amides.

A

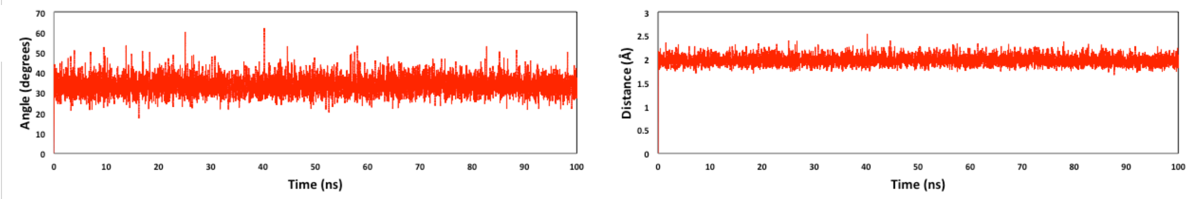

A: Distance (Å) between the –NH hydrogen atom and the O atom of the intramolecular hydrogen bond during simulation of (*R*)-**2** bound to CREBBP. No disruption of this interaction is observed during the 100 ns of MD.

B

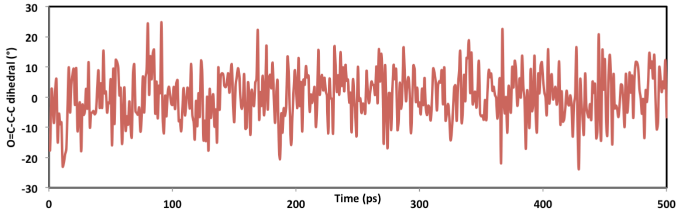

B: Impact of solvent on the conformational bias of inhibitor (*R*)-**2**. To verify the conformational preference of dihydroquinoxalinone amides obtained from DFT in implicit solvent (Figure 3B, **(*R*)-10**), we performed a molecular dynamics simulation using the full structure of **(*R*)-2** (500 ps) in explicit water. Parameters for the organic molecule were taken from the General Amber Force Field (GAFF), along with RESP atomic charges which was immersed in a cubic 12 Å x 12 Å x 12 Å box of TIP-3P water molecules. For the duration of the simulation the O=C-C-C dihedral angle is maintained close to the global energy minimum as is predicted from DFT, due to the existence of the intramolecular N-H---O=C hydrogen bond.

Supporting Figure S8: Quantification of the cation-π interaction.

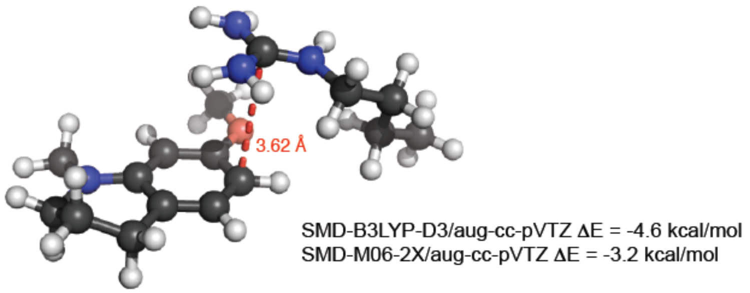

Quantum chemical computed interaction energy between ligand and R1173 in the crystallographic coordinates. These calculations provide a quantitative estimate of the interaction strength as in the region of −3.2 to −4.6 kcal/mol. These computed values agree well with the experimentally measured average strengths of cation-π interactions involving either Lys (−3.3 ± 1.5 kcal/mol) or Arg (−2.9 ± 1.4 kcal/mol).

Supporting Figure S9: FRAP data for (*R*)-**2** in U2OS cells.

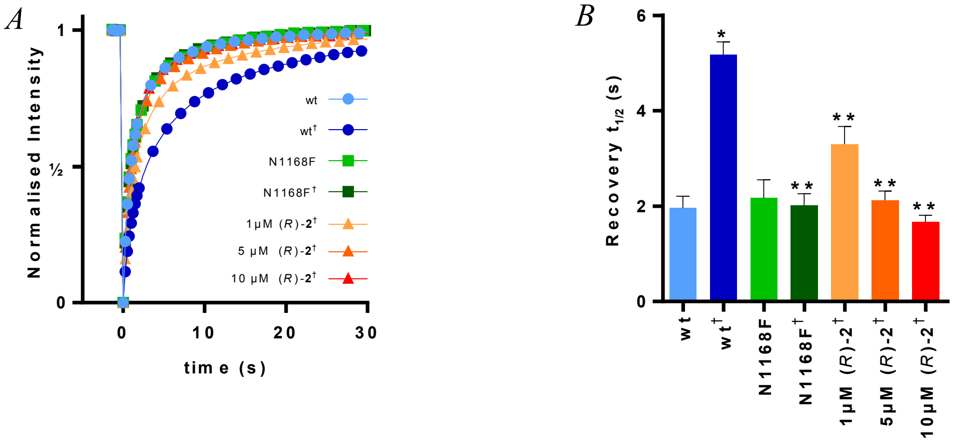

Fluorescence Recovery After Photo-bleaching (FRAP) demonstrating compound (*R*)-**2** can displace the CREBBP bromodomain from chromatin. **A)** Time dependence of fluorescent recovery in the bleached area of U2OS cells expressing GFP chimerised to multimerised CREBBP bromodomains. Curves represent the means at each time point of 15 cells in each group (see Figure 3A for representative images of cell nuclei). **B)** Half times of fluorescence recovery (t½) of cells expressing GFP chimerised to multimerised CREBBP bromodomains. Bars represent the mean t½ calculated from individual recovery curves of 15 cells per group. * significantly different from wt, $p<0.0001$. ** significantly different from wt†, $p<0.0001$.

Supporting Scheme S1: Synthesis of dihydroquinoxalinone carboxylic acid.

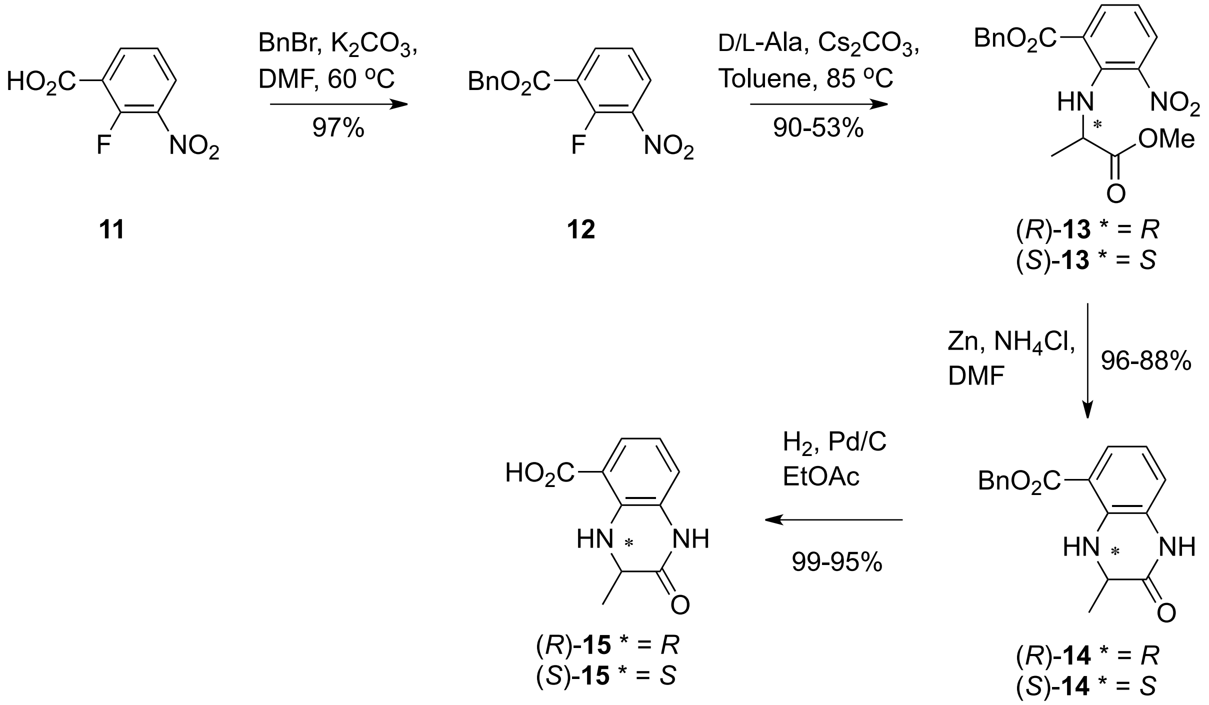

Supporting Scheme S2: Synthesis of 6-fluoro-dihydroquinoxalinone derivatives.

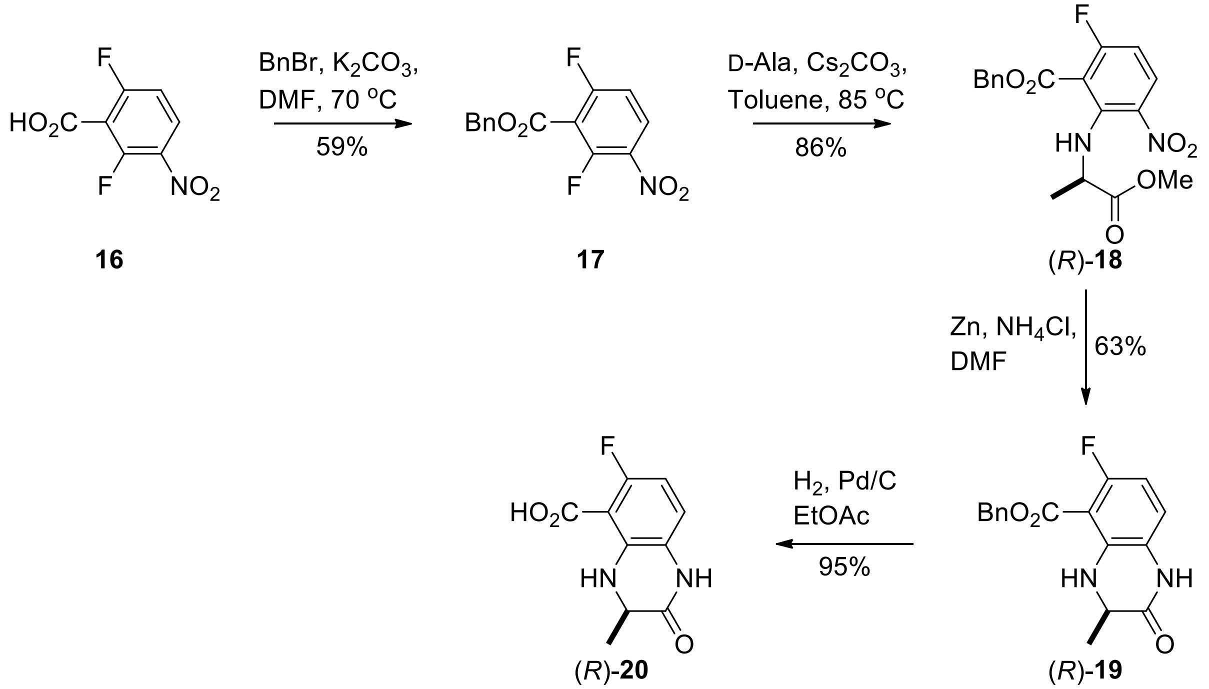

Supporting Scheme S3: Synthesis of 7-fluoro-dihydroquinoxalinone derivatives.

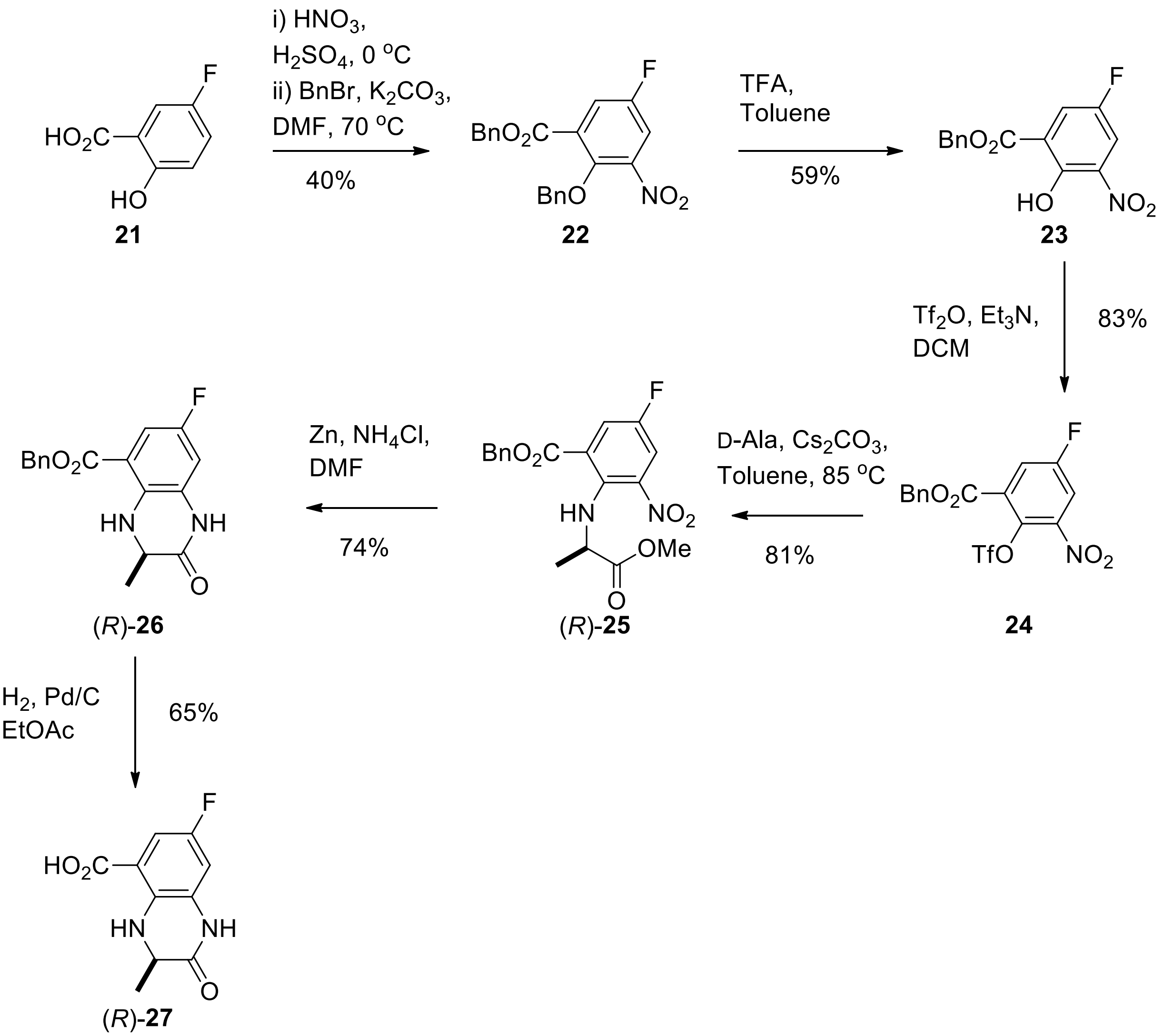

Supporting Scheme S4: Synthesis of dihydroquinoxalinone amides.

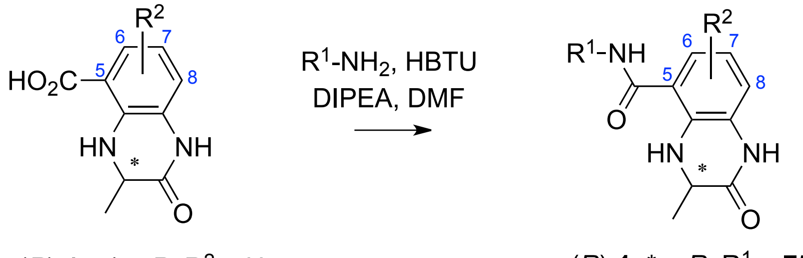

(R)-**15**: * = R, R^2 = H
(S)-**15**: * = S, R^2 = H
(R)-**20**: * = R, R^2 = 6-F
(R)-**27**: * = R, R^2 = 7-F

(R)-**1**: * = R, R^1 = **75**, R^2 = H
(S)-**1**: * = S, R^1 = **75**, R^2 = H
(R)-**6**: * = R, R^1 = **76**, R^2 = H
(R)-**2**: * = R, R^1 = **74**, R^2 = H
(R)-**5**: * = R, R^1 = **77**, R^2 = H
(R)-**8**: * = R, R^1 = **74**, R^2 = 6-F
(R)-**7**: * = R, R^1 = **74**, R^2 = 7-F

Supporting Scheme S5: Synthesis of benzoxazinone fragments.

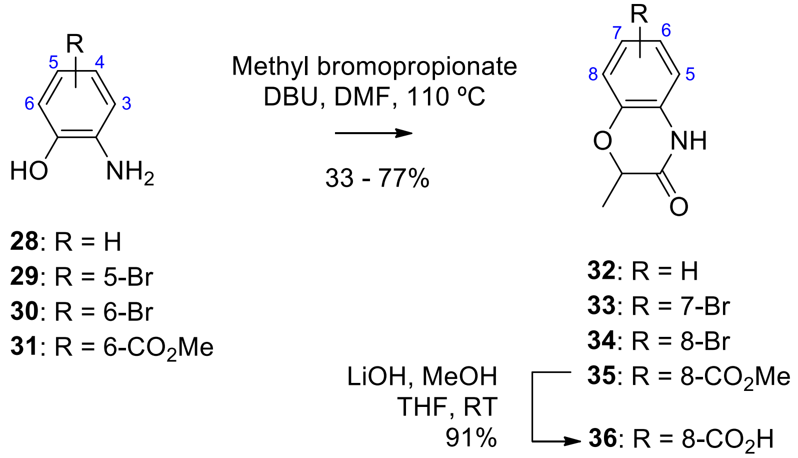

Supporting Scheme S6: Synthesis of benzoxazinone amide analogues.

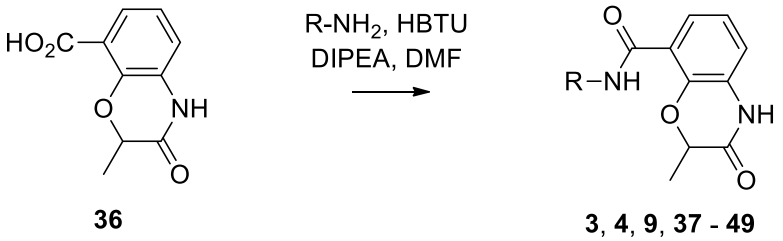

Supporting Scheme S7: Synthesis of single enantiomer benzoxazinone amide.

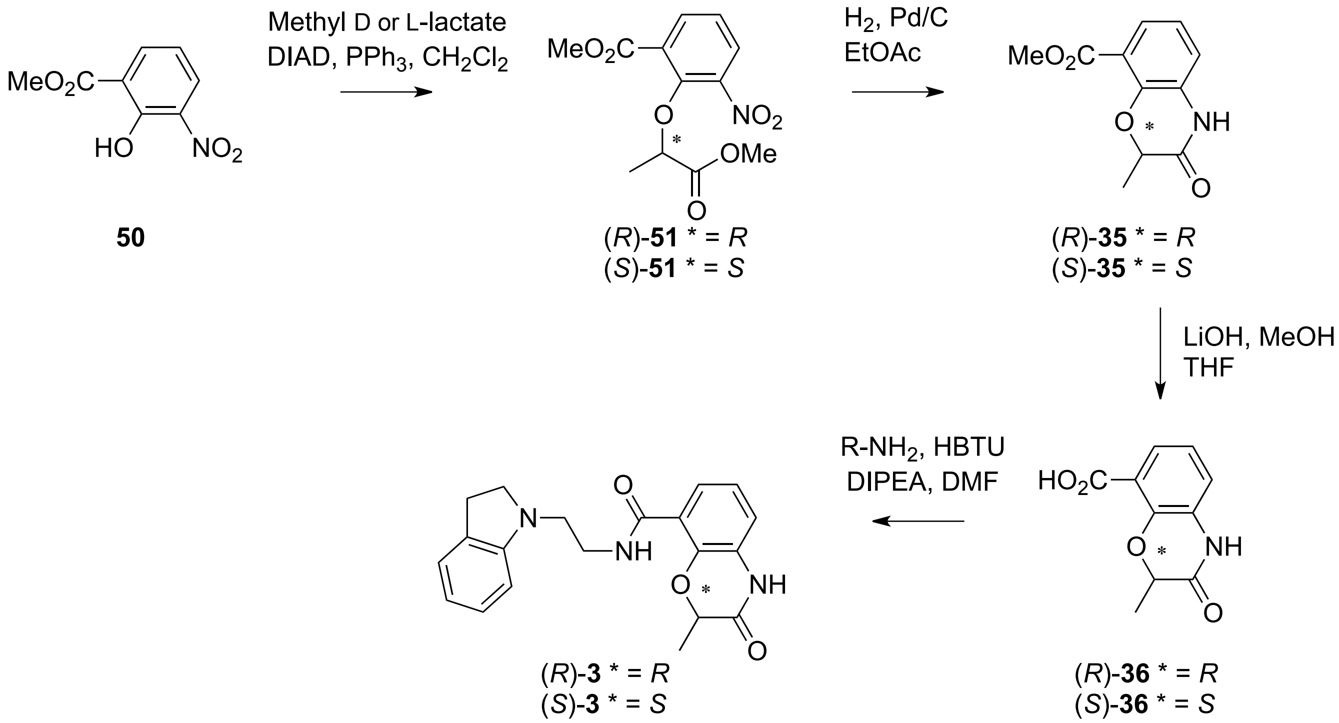

Supporting Scheme S8: Synthesis of DHQX-carboxylic acid from 2-hydroxy-3-nitrobenzoic acid.*

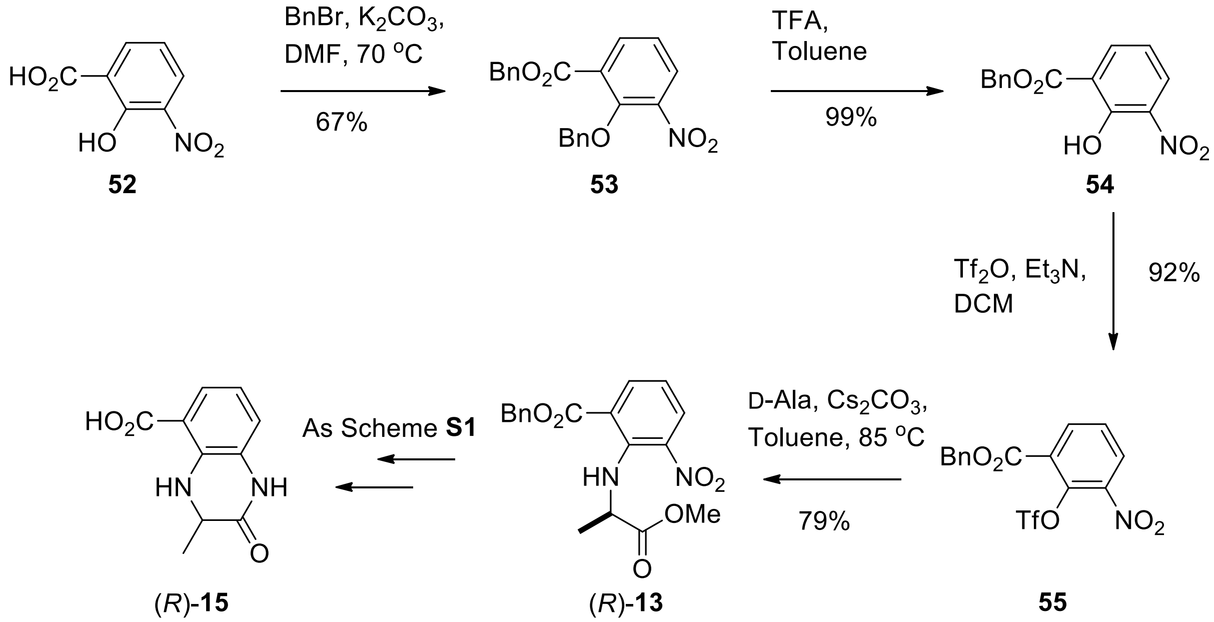

*This route gave a lower overall yield than Scheme S1, although compound **52** is more readily available than compound **11**.

Supporting Scheme S9: Synthesis of DHQX-carboxylic acid from methyl 3-nitro-2-hydroxybenzoate.*

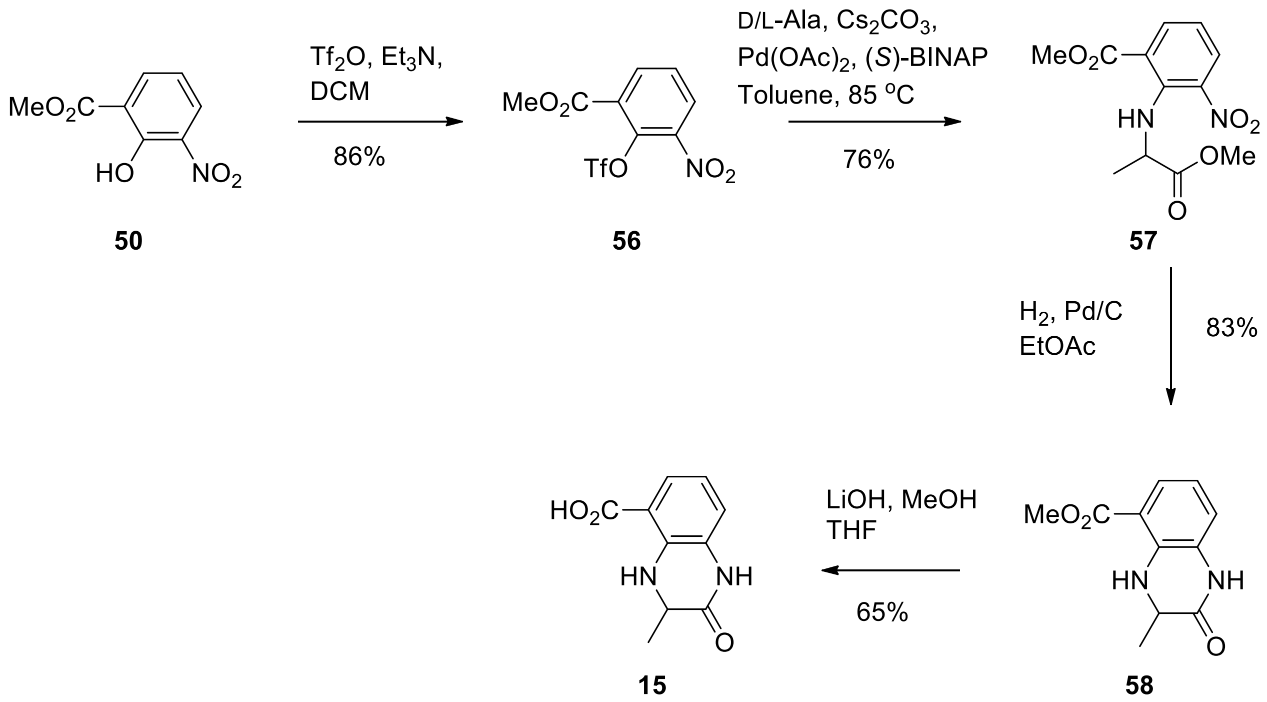

*Scheme S1 was preferred for the syntheses of (*R*)-**15** since deprotection of (*R*)-**58** resulted in some racemisation.

Supporting Scheme S10: Synthesis of 7-methoxy-1,2,3,4-tetrahydroquinoline.

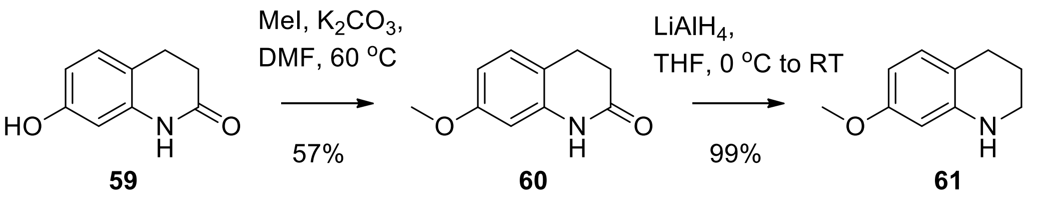

Supporting Scheme S11: Synthesis of amine coupling partners.

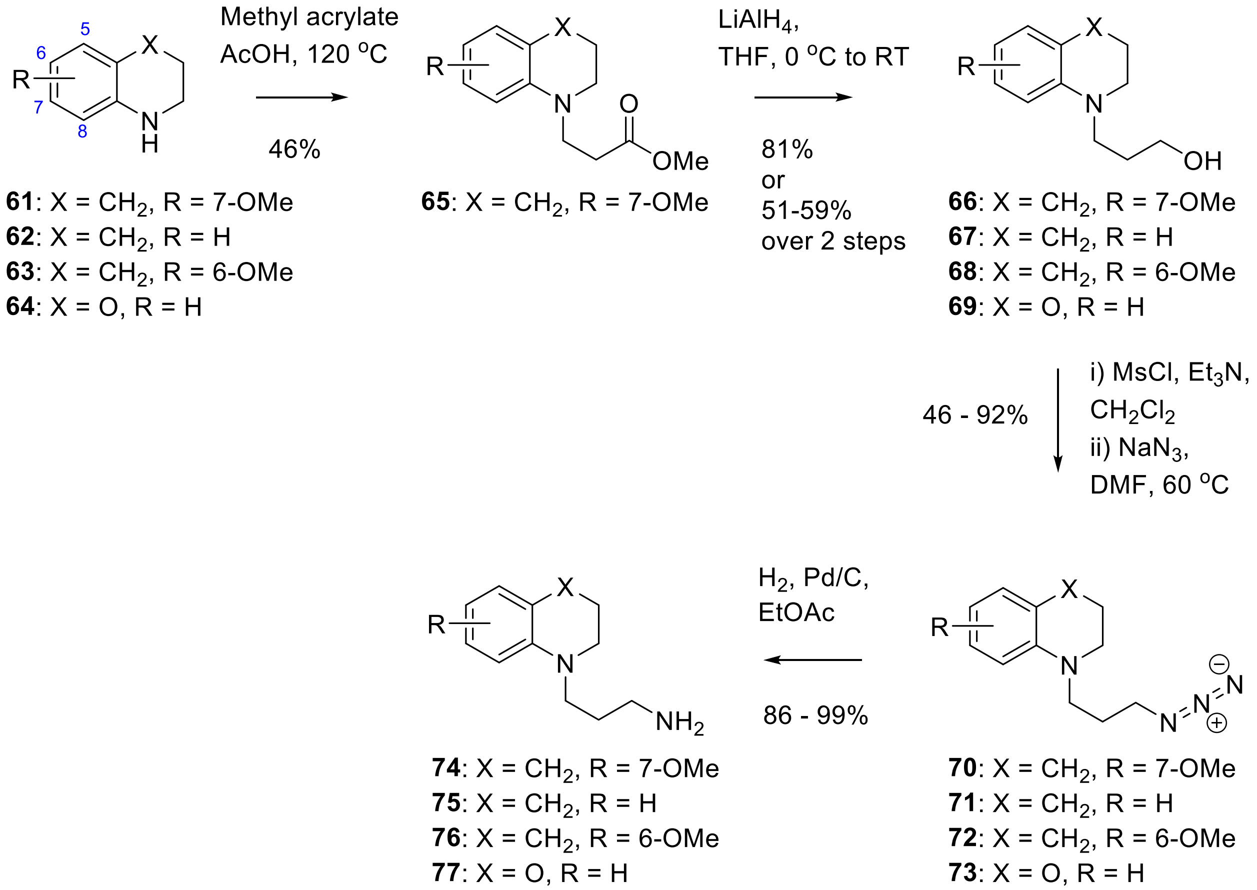

Supporting Table S1: AlphaScreen data for fragment benzoxazinone-based CREBBP bromodomain inhibitors. Heat map shows relative IC_{50} values obtained in an ALPHA assay. Red indicates low IC_{50} values, and green/yellow indicates high IC_{50} values. Ranges in parentheses represent 95% confidence intervals resulting from sigmoidal curve fitting to duplicate data.

Number	Structure	IC_{50} / µM CREBBP peptide & protein = 400 nM
**DHQ**	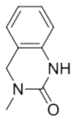	29.4 (25.5-33.8)
**28**	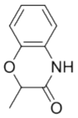	107 (93.1-123)
**31**	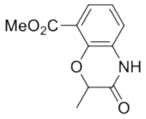	222 (158-311)
**30**	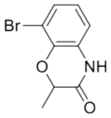	396 (198-791)
**29**	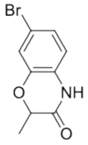	7450 (348->10^6)

Supporting Table S2: AlphaScreen data for inhibition of CREBBP by 8-amido substituted benzoxazinones. Heat map shows relative IC_{50} values obtained in an ALPHA assay. Red indicates low IC_{50} values, and green/yellow indicates high IC_{50} values. Ranges in parentheses represent 95% confidence intervals resulting from sigmoidal curve fitting to duplicate data.

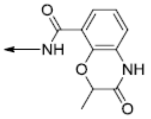

Number	Structure	IC_{50} / µM CREBBP peptide & protein = 400 nM	Number	Structure	IC_{50} / µM CREBBP peptide & protein = 400 nM	Number	Structure	IC_{50} / µM CREBBP peptide & protein = 400 nM
**4**	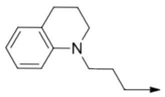	51.4 (46.7-56.4)	**36**	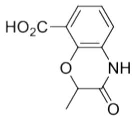	167 (151-186)	**40**	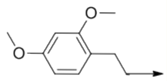	329 (294-367)
**37**	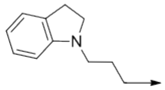	68.6 (62.6-75.2)	**41**	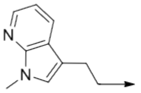	173 (161-186)	**9**	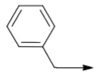	499 (336-740)
**3**	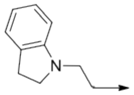	76.9 (70.6-83.8)	**45**	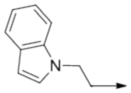	199 (174-227)	**47**	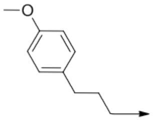	1280 (682-2400)
**42**	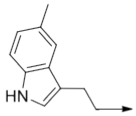	81.2 (74.9-88.1)	**49**	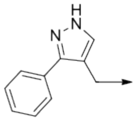	214 (201-226)	**44**	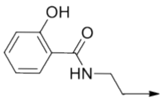	1532 (562-4180)
**39**	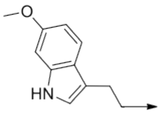	97.0 (89.0-106)	**48**	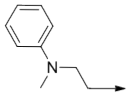	242 (228-256)	**38**	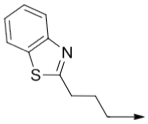	15100 (2410-94100)

Supporting Table S3: Protein crystallization data collection and refinement statistics.

**Data Collection**			
PDB ID	4NYW	4NYX	4NYV
Protein/Ligand	CREBBP/ (*R*)-**1**	CREBBP/ (*R*)-**2**	CREBBP/<br>6-bromo-DHQ
Space group	$P2_12_12_1$	I2	P1
Cell dimensions: a, b, c (Å)<br>α, β, γ (deg)	34.96 49.91 80.14<br>90.00 90.00 90.00	40.05 34.36 90.93<br>90.00 97.72 90.00	36.15 55.68 49.08<br>90.21 104.03 108.61
Resolution* (Å)	1.42 (1.50-1.42)	1.10 (1.16-1.10)	1.83 (1.93-1.83)
Unique observations*	26470 (3748)	49269 (6971)	40271 (5717)
Completeness* (%)	98.4 (97.2)	98.6 (96.6)	92.5 (89.2)
Redundancy*	11.7 (7.4)	3.1 (2.6)	2.4 (2.4)
Rmerge*	0.097 (0.866)	0.022 (0.105)	0.053 (0.399)
I/ σI*	15.1 (2.0)	23.4 (7.0)	10.1 (2.0)
**Refinement**			
Resolution (Å)	1.42	1.10	1.83
R_{work} / R_{free} (%)	16.4/18.7	11.9/13.5	19.5/25.1
Number of atoms<br>(protein/other/water)	985/43/186	983/149/30	3879/52/262
B-factors ($Å^2$)<br>(protein/other/water)21.85	15.59/18.95/29.37	14.12/26.29/14.59	27.62/34.64/29.52
r.m.s.d bonds (Å)<br>r.m.s.d angles (^{o})	0.014<br>1.529	0.014<br>1.741	0.016<br>1.497
Ramachadran Favoured (%)<br>Allowed (%)<br>Disallowed (%)	99.11<br>0.89<br>0.00	100.00<br>0.00<br>0.00	100.00<br>0.00<br>0.00

* Values in parentheses correspond to the highest resolution shell.

Supporting Table S4: Optimized coordinates of ligand (*R*)-**2** following HF/6-31G**//B3LYP/cc-pVTZ optimization//MEP calculations and RESP charges obtained using the R.E.D. Server.

Atom	X	Y	Z	Resp Charge
C1	-4.49463	-1.35448	1.953902	-0.1530
H1	-5.29576	-1.19487	2.654711	0.1465
C2	-3.73991	-2.51916	2.004497	-0.2038
H2	-3.9635	-3.27445	2.734955	0.1535
C3	-2.71314	-2.69765	1.107984	-0.1690
H3	-2.15389	-3.61457	1.132241	0.1178
C4	-2.42085	-1.73501	0.136494	-0.0402
C5	-1.28993	-1.93951	-0.82930	0.3926
N1	-0.26591	-2.70445	-0.39902	-0.3077
H4	-0.23670	-2.96645	0.556343	0.2761
C6	0.871796	-3.0273	-1.24578	-0.1390
H5	0.50623	-3.12722	-2.25731	0.1071
H6	1.241003	-3.99945	-0.93195	0.1071
C7	2.001156	-1.99749	-1.20690	0.0148
H7	1.622716	-1.03802	-1.54035	0.0357
H8	2.75476	-2.30583	-1.92776	0.0357
C8	2.652575	-1.87042	0.175937	-0.0331
H9	2.98234	-2.85437	0.499043	0.0452
H10	1.921987	-1.54983	0.90847	0.0452
N2	3.805225	-0.99904	0.243739	-0.1610
C9	5.056628	-1.56102	-0.22896	-0.0056
H11	5.139689	-1.50542	-1.31692	0.0612
H12	5.071507	-2.61172	0.038381	0.0612
C10	6.238283	-0.84577	0.405625	-0.0277
H13	7.166726	-1.30563	0.079361	0.0213
H14	6.175563	-0.95566	1.484489	0.0213
C11	6.200879	0.627594	0.022355	0.0068
H15	6.514882	0.734634	-1.01524	0.0197
H16	6.914419	1.189652	0.617765	0.0197
C12	4.812075	1.20601	0.203364	0.0348
C13	3.675459	0.390302	0.27848	-0.0433
C14	2.415302	0.992398	0.425076	-0.1354
H17	1.537003	0.386224	0.465895	0.1420
C15	2.290525	2.368159	0.507818	0.1640
O1	1.111765	3.006161	0.649698	-0.2809
C16	-0.08937	2.287968	0.621905	0.0097
H18	-0.88103	3.017623	0.707404	0.0602
H19	-0.2091	1.744924	-0.30882	0.0602
H20	-0.15984	1.593841	1.454048	0.0602
C17	3.415621	3.180543	0.444972	-0.2219
H21	3.303557	4.246087	0.511057	0.1268
C18	4.644669	2.581986	0.294063	-0.2264
H22	5.522154	3.205625	0.244025	0.1434
O2	-1.28986	-1.44676	-1.93443	-0.4999
C19	-3.20268	-0.57883	0.061186	0.1399
C20	-4.224	-0.39533	1.005443	0.0694
N3	-4.93593	0.809892	0.965407	-0.3492
H23	-5.50661	1.068056	1.73905	0.3089
C21	-4.96671	1.658972	-0.09634	0.4766
O3	-5.61054	2.66699	-0.08402	-0.5475
C22	-4.16899	1.18481	-1.29896	0.1676
H24	-4.84637	0.534742	-1.86114	0.0522
N4	-2.99902	0.447927	-0.84598	-0.4646
H25	-2.39839	0.175732	-1.59427	0.3090
C23	-3.74957	2.346583	-2.18553	-0.2196
H26	-3.22767	1.975326	-3.06163	0.0718
H27	-4.61865	2.90298	-2.50765	0.0718
H28	-3.08965	3.016321	-1.64728	0.0718

Supporting Table S5: Coordinates of the truncated model used for evaluation of the strength of the cation-π interaction energy.

Atom	X	Y	Z
C(Fragment=2)	11.20700	38.51700	20.37700
C(Fragment=2)	10.77900	39.84200	19.75100
C(Fragment=2)	11.28501	41.08801	20.47401
C(Fragment=2)	11.03604	42.35093	19.69004
C(Fragment=2)	11.74906	43.16897	17.44897
N(Fragment=2)	10.86301	44.14001	17.48401
N(Fragment=2)	11.86589	42.34809	18.47600
N(Fragment=2)	12.56700	43.06700	16.42400
O(Fragment=1)	10.40500	40.46500	15.74100
N(Fragment=1)	7.78700	43.50200	13.08300
C(Fragment=1)	8.35300	43.09700	11.82900
C(Fragment=1)	6.59600	44.35000	12.97600
C(Fragment=1)	6.34600	45.12400	14.24900
C(Fragment=1)	6.21500	44.11100	15.36500
C(Fragment=1)	7.33100	43.15300	15.42800
C(Fragment=1)	7.59800	42.47200	16.57500
C(Fragment=1)	8.62199	41.54100	16.64998
C(Fragment=1)	9.39501	41.31000	15.56000
C(Fragment=1)	9.13100	41.99101	14.35101
C(Fragment=1)	8.12100	42.89800	14.27700
C(Fragment=1)	11.27900	40.22100	14.70400
H(Fragment=2)	9.68233	39.88944	19.71001
H(Fragment=2)	11.10333	39.86220	18.70109
H(Fragment=2)	10.79578	41.18557	21.44912
H(Fragment=2)	12.35998	41.00239	20.69300
H(Fragment=2)	11.26766	43.24319	20.28517
H(Fragment=2)	9.98368	42.39465	19.38212
H(Fragment=2)	12.67436	41.74099	18.49601
H(Fragment=2)	10.22423	44.20254	18.26145
H(Fragment=2)	10.49098	44.49951	16.61495
H(Fragment=2)	13.05913	42.20479	16.24563
H(Fragment=2)	12.50152	43.70923	15.64906
H(Fragment=2)	10.86159	38.43327	21.41291
H(Fragment=1)	12.03275	39.52124	15.07356
H(Fragment=1)	10.78835	39.76182	13.83483
H(Fragment=1)	11.78219	41.13825	14.35438
H(Fragment=1)	9.72031	41.76308	13.47369
H(Fragment=1)	8.81177	40.98001	17.55989
H(Fragment=1)	6.97564	42.65061	17.44947
H(Fragment=1)	6.09950	44.61761	16.33227
H(Fragment=1)	5.26911	43.56648	15.21908
H(Fragment=1)	7.18533	45.80610	14.43476
H(Fragment=1)	5.44029	45.72882	14.15252
H(Fragment=1)	6.75028	45.03561	12.13711
H(Fragment=1)	5.70955	43.74162	12.73164
H(Fragment=1)	9.44476	43.22145	11.80967
H(Fragment=1)	8.13620	42.04477	11.58657
H(Fragment=1)	7.94587	43.71427	11.02860
H(Fragment=2)	10.79372	37.67294	19.81885
H(Fragment=2)	12.29790	38.41308	20.38368

Supporting Table S6: Conformational energy profiles for a simplified model benzoxazinone and dihyrdoquinoxalinone. CPCM-wB97XD/TZVP//wB97XD/TZVP energies are calculated for rotation about the O=C-C=C dihedral angle for molecules (*R*)-**9** and (*R*)-**10**.

Dihedral Angle (°)	(*R*)-9 E (Hartree)	(*R*)-10 E (Hartree)
0	-992.6666449	-972.8184132
30	-992.6717798	-972.8204279
60	-992.6736122	-972.8168118
90	-992.6730923	-972.8149650
120	-992.6745265	-972.8192018
150	-992.6779346	-972.8176322
180	-992.6791168	-972.8108877
210	-992.6793833	-972.8164043
240	-992.6761782	-972.8150656
270	-992.6757483	-972.8140823
300	-992.6748365	-972.8162256
330	-992.6713736	-972.8197029
360	-992.6666449	-972.8184132

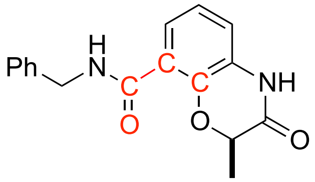

(*R*)-**9**

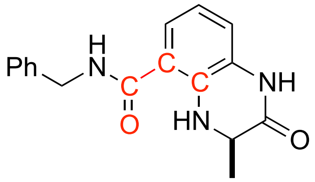

(*R*)-**10**

The conformational energy profiles were calculated for structures (*R*)-**9** and (*R*)-**10** with density functional theory (DFT), using *Gaussian 09*, rev D.01.[7] The dihedral angle associated with an out-of-plane rotation of the amide carbonyl group was constrained in increments of 30° while the rest of the structure was fully optimized to produce the energy profiles shown in **Figure 3** in the main text. Energies include the effects of solvation using an implicit conductor-like polarizable continuum model (CPCM) description of water solvation,[8] and are tabulated above. All optimizations employ the range-separated, dispersion corrected, wB97XD density functional of Chai and Head- Gordon,[9] which performs well in its description of H-bonding and non-bonding interactions, in conjuction with the TZVP basis set of Aldrich and co- workers.[10] The energies are quoted in kcal/mol exclusive of zero-point vibrational energy.

Supporting Table S7: Calculated ligand properties and ligand efficiency.

Number	Structure	MW	H-donor	H-acceptor	cLogP	Rot. Bonds	TPSA	Heavy atom	K_D (nM)	IC_{50} (nM)	pIC_{50}	LE	LLE
**NMP**	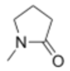	99.13	0	2	-0.40	0	20.31	7	NA	1900000	2.72	0.54	3.12
**DHQ**	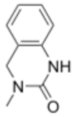	162.19	1	3	0.66	0	32.34	12	NA	29400	4.53	0.53	3.87
**28**	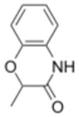	163.17	1	3	0.98	0	38.33	12	NA	107000	3.97	0.46	2.99
**4**	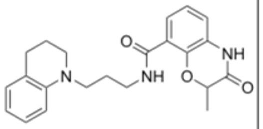	379.45	2	6	2.68	5	70.67	28	NA	51400	4.29	0.21	1.61
(*R*)-**1**	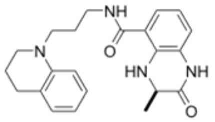	378.47	3	6	3.73	5	73.47	28	NA	758	6.12	0.31	2.39
(*R*)-**2**	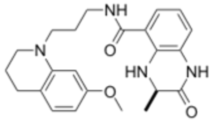	408.49	3	7	3.63	6	82.70	30	391	323	6.49	0.30	2.86
(*R*)-**7**	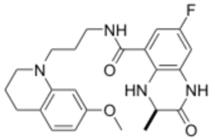	426.48	3	7	4.81	6	82.70	31	NA	1526	5.82	0.26	1.01

cLogP was calculated using ACD/I-Lab. TPSA = total polar surface area. K_D values were obtained by ITC. IC_{50} values were obtained by AlphaScreen [Table 1, S1, S2 and ref(11)], although run under different conditions for fragment screening. $pIC_{50} = -\log_{10} IC_{50}$. Ligand efficiency LE = ($pIC_{50} \times 1.4$)/Heavy atom. Lipophilic ligand efficiency LLE = $pIC_{50} - cLogP$.[12]

Computational Methods

Docking Studies: Docking of 8-amido substituted benzoxazinones was carried out using ICM-Pro 3.6-1 (Molsoft LLC). The X-ray crystal structure of 6-bromo-DHQ bound to CREBBP (PDB ID: 4NYV) was used to define the receptor and the bicyclic core used as a template for placement of ligands. Four structurally important water molecules (1-4 in Figure S1A) at the base of the ligand pocket were retained in the model of the receptor. A database of primary amines compiled from commercially available sources was used to generate an *in silico* library of benzoxazinones which was docked using the in-built docking procedure in ICM-Pro.

Molecular Dynamics: The R.E.D. (RESP and ESP Derivation) server version 4.0[13] was used for geometry optimization of ligand (*R*)-**2** and for RESP charge derivation, using the methodology previously proposed by Duan and coworkers.[14] Geometry optimization was done in the Hartree Fock 6-31G** level of QM theory using Gaussian 09 revision D. Molecular electrostatic potentials (MEP) derived from the Connolly Surface Algorithm were computed with the B3LYP exchange and correlation density functional with a triple-ζ valence polarized cc-pVTZ basis set,[14,15] employing a IEFPCM continuum solvation model to describe the relatively hydrophobic protein site (ε=4, Et_2O as solvent). The electrostatic potentials of the inhibitor were saved and were used in charge fitting. RESP charges and Cartesian coordinates are available as a supplementary material.

AutoDock Vina 1.1.2[16] was used for docking ligand (*R*)-**2** to the protein structure. The size of the docking grid was 13.3 Å × 16.7 Å × 14.5 Å, centered on the inhibitor, with all other parameters maintained as default values. The low value of the root-mean-square deviation (RMSD), equal to 0.81 Å, between the pose with the lowest docking energy and the X-ray crystal structure of (*R*)-**2** bound to (*R*)-**2** suggested that this starting point for MD is in accordance with structural data available from experiment.[17]

For molecular dynamics (MD) simulation molecular mechanics parameters for inhibitor (*R*)-**2** were taken from the General AMBER Force Field (GAFF)[18] with RESP atomic charges. Hydrogens were removed from amino acid residues using the *MolProbity* Server[19] and added using *tLeap*.[20] Glutamate and aspartate residues were assigned as negatively charged and lysine and arginine as positively charged. Minimization and MD calculations were performed using the AMBER Force Field 99SB within *AMBER* version 12.[20] Crystallographic waters were not removed, while the protein was further solvated by a box of TIP-3P water molecules.[21] Simulations were carried out in cubic boxes of 0.19 nm × 0.19 nm × 0.19 nm (an initial volume of 72.80 nm^3) containing 1,166 water molecules. Two counter-cations (Na^+) were added to equilibrate the

system. Energy minimization was performed in two steps. Firstly, we used steepest descent followed by conjugate gradients with position of the inhibitor restrained (PR). Secondly, the same minimization methodology was performed without PR. The minimized complex was then subjected to 500 ps of equilibration and 100 ns of production MD simulation in the NPT ensemble using a Langevin thermostat to simulate a constant temperature at 310 K (τ_T = 0.1 ps). Isotropic position scaling was used to maintain the pressure of 1 atm (τ_p = 2 ps).[22] MD simulation was carried out using 1 fs of integration time and a nonbonding cut-off of 8 Å, with the Shake algorithm[23] turned on to constrain bonds involving hydrogen. A total of 5,000 snapshots were obtained at intervals of 20 ps in producing plots of the geometric variation during the simulation. Trajectory visualization and graphical analysis were generated using Visual Molecular Dynamics (VMD).[24] The formation of intramolecular or intermolecular hydrogen bonds was judged according to whether the criteria of a donor−acceptor distance of less than 3.0 Å and an acceptor−donor−hydrogen angle smaller than 35° were satisfied.

Density Functional Theory calculations: A truncated model of the cation-π interaction was constructed from the crystallographic coordinates for which the positions of hydrogen atoms were optimized with density functional theory (DFT) calculations.[25] The energy of this complex relative to the separated substrate and arginine residue was evaluated at the B3LYP-D3/aug-cc-pVTZ level of theory,[26] with a Boys-Bernadi counterpoise correction for basis set superposition error (BSSE), and with an implicit solvent model[27] (diethylether, ρ=4) to mimic the surrounding hydrophobic protein residues. Grimme's D3-correction for London dispersion was employed with Becke-Johnson damping in the short range.[28] Additionally we also evaluated this energy at the M06-2X/aug-cc-pVTZ level of theory,[29] since this functional also captures medium-range correlation (and hence weak dispersive interactions) through the inclusion of the kinetic energy density in the exchange correlation functional.

SMD-B3LYP/aug-cc-pVTZ energies (Hartree)

Complex: -921.7209747

Fragment A: -558.4296904

Fragment B: -363.2834692

BSSE: 0.000424677

SMD-M06-2X/aug-cc-pVTZ energies (Hartree)

Complex: -921.2198694

Fragment A: -558.1390479

Fragment B: -363.0753134

Detail of the internal H-bond DFT calculation is given in Table S6.

Physical Methods

X-Ray Diffraction: Low temperature X-ray diffraction data were collected using an Oxford Diffraction (Agilent) SuperNova Diffractometer with microfocus Cu-Kα radiation using standard procedures at 150 K.[30] The structure was solved by direct methods (SIR92)[31] within the CRYSTALS[32] suite as per the CIF/ESI; all non-hydrogen atoms were refined with anisotropic thermal parameters. Hydrogen atoms were positioned geometrically and optimised prior to inclusion in the final refinements using a riding model.[33] These data (Figure S2) can be obtained free of charge from The Cambridge Crystallographic Data Centre (CDCC ZZZZZZ) *via* www.ccdc.cam.ac.uk/data_request/cif.

Biochemical Methods

Peptide Synthesis: H3K56Ac peptide (H_2N-ALREIRRYQK(Ac)STELLIRKLK-(biotin)-COOH) for use in the CREBBP AlphaScreen assay was synthesized by Tufts University Core Facility.

AlphaScreen Peptide Displacement Assay: Bromodomain AlphaScreen assays were carried out as previously described.[11,34] All experiments were carried out in duplicate and, within a table, on the same plate.

Isothermal Titration Calorimetry (ITC): Experiments were carried out on a VP-ITC titration microcalorimeter from MicroCal™, LLC (Northampton, MA) with a cell volume of 1.4189 ml and a 250 µl microsyringe equipped with a ThermoVac module. All experiments were carried out at 15 °C while stirring at 295 rpm, in ITC buffer (50 mM HEPES pH 7.4 (at 25 °C), 150 mM NaCl). The microsyringe was loaded with a solution of the protein sample (200-500 µM protein in ITC buffer) and was carefully inserted into the calorimetric cell which was filled with an amount of the ligand (200 µl, 20-30 µM in ITC buffer). The system was first allowed to equilibrate until the cell temperature reached 15 °C and an additional delay of 120 sec was applied. All titrations were conducted using an initial control injection of 2 µl followed by up to 34 identical injections of 8 µl with a duration of 4 sec per injection and a spacing of 250 sec between injections. The titration experiments were designed in such a fashion, as to ensure complete saturation of the enzymes before the final injection. The heat of dilution for the proteins were independent of their concentration and corresponded to the heat observed from the last injection, following saturation of ligand binding, thus facilitating the estimation of the baseline of each titration from the last injection. The collected data were corrected for protein heats of dilution (measured on separate experiments by titrating the proteins into ITC buffer) and deconvoluted using the MicroCal™ Origin software supplied with the instrument to yield enthalpies of binding (ΔH) and binding constants (K_B) in the same fashion to that previously described in detail by Wiseman and coworkers.[35] Thermodynamic parameters were calculated using the basic equation of thermodynamics ($\Delta G = \Delta H - T\Delta S = -RT\ln K_B$, where ΔG, ΔH and ΔS are the changes in free energy, enthalpy

and entropy of binding respectively). In all cases a single binding site model was employed, supplied with the MicroCal™ Origin software package. Dissociation constants and thermodynamic parameters are listed in Figure S3.

Differential Scanning Fluorimetry: Bromodomain ΔTm assay was performed as reported previously,[34] with protein at a final concentration of 2 μM and compound at 10 μM.

Protein Expression and Purification: cDNAs encoding human CREBBP (NCBI accession number 004371.1) was obtained from FivePrime and was used as template to amplify the bromodomain region of the protein. The protein was cloned, expressed and purified as previously described.[36]

Crystallization: Aliquots of the purified protein were set up for crystallization using a mosquito® crystallization robot (TTP Labtech, Royston UK). Coarse screens were typically setup onto Greiner 3-well plates using three different drop ratios of precipitant to protein per condition (100+50 nl, 75+75 nl and 50+100 nl). Initial hits were optimized further scaling up the drop sizes. All crystallizations were carried out using the sitting drop vapor diffusion method at 4 °C. CREBBP crystals with (*R*)-**1** were grown by mixing 200 nl of protein (9.7 mg/ml and 1 mM final ligand concentration) with 100 nl of reservoir solution containing 20 % PEG 3350, 9 % ethylene glycol and 0.18 M KSCN. CREBBP crystals with (*R*)-**2** were grown by mixing 150 nl of the protein (8.0 mg/ml with 1 mM of final ligand concentration) with an equal volume of reservoir solution containing 0.05 M $CaCl_2$, 0.1 M TRIS pH 8.0, 20 % PEG 6K and 10 % ethylene glycol. CREBBP crystals with 6-bromo-DHQ were grown by mixing 150 nl of the protein (8.0 mg/ml with 1 mM of final ligand concentration) with an equal volume of reservoir solution containing 0.15 M KSCN, 25 % PEG 3350 and 5 % ethylene glycol. In all cases diffraction quality crystals grew within a few days.

Data Collection and Structure Solution: All crystals were cryo-protected using the well solution supplemented with additional ethylene glycol and were flash frozen in liquid nitrogen. Data were collected at Diamond beamline I04.1 ((*R*)-**1**) or I04 ((*R*)-**2**) at a wavelength of 0.9173 Å (I04-1) or 0.92 Å (I04) or in house (6-bromo-DHQ) at an FRE Superbright with an RAXIS-VI detector at a wavelength of 1.52 Å. Indexing and integration was carried out using XDS[37,38] and scaling was performed with SCALA[39]. Initial phases were calculated by molecular replacement with PHASER[40]using an ensemble of known bromodomain models (PDB IDs 3DAI, 3HMH, 2GRC, 2OO1, 2OSS, 2OUO, 3D7C, 3DWY). Initial models were built by ARP/wARP[41] followed by manual building in COOT[42]. Refinement was carried out in REFMAC5.[43] Thermal motions were analyzed using TLSMD[44] and hydrogen atoms were included in late refinement cycles. Data collection and refinement statistics can be found in Table S3. The model and structure factors have been deposited with PDB accession codes: 4NYW (CREBBP/(*R*)-**1**); 4NYX (CREBBP/(*R*)-**2**); 4NYV (CREBBP/6-bromo-DHQ).

Fluorescence recovery after photobleaching (FRAP) assay: FRAP assay was performed as previously described.[45]

Synthetic Methods

Reagents and solvents used, unless otherwise stated, were of commercially available reagent grade quality and were used without further purification. Where appropriate and if not stated otherwise, all non-aqueous reactions were carried out in a flame dried flask under an inert atmosphere of nitrogen or argon. Anhydrous solvent was purchased from SigmaAldrich UK in SureSeal™ bottles, or dried according to the procedure outlined by Pangborn *et al.*,[46] and used without purification unless otherwise indicated. Solvents for use in organometallic reactions were degassed by three freeze-thaw cycles under vacuum and were stored under an argon atmosphere over 3 Å molecular sieves. Triethylamine for use in organometallic reactions was dried with KOH and distilled onto KOH pellets. *In vacuo* refers to solvent removal under reduced pressure using a Buchi™ rotary evaporator. Brine refers to a saturated aqueous solution of sodium chloride. Petroleum ether refers to the fraction of light petroleum ether boiling in the range 40-60 °C.

Analytical thin layer chromatography (TLC) was carried out on Merck silica gel 60 F_{254} aluminium-supported thin layer chromatography sheets. Visualisation was by absorption of UV light (λ_{max} 254 or 365 nm), or thermal development after dipping in one of: **a** ethanolic solution of phosphomolybdic acid; **b** aqueous solution of potassium permanganate, potassium carbonate and sodium hydroxide; **c** ethanolic solution of ninhydrin.

Flash Column chromatography was carried out either on Merck silica gel 60 (240-400 mesh), eluting with solvents as supplied under a positive pressure of compressed air, or on a Biotage SP1 system using KP-Sil™ cartridges.

Melting points were determined using a Kofler hot stage microscope and are uncorrected.

Specific optical rotations were measured using a Perkin-Elmer 241 or 341 polarimeter with a water-jacketed 1 dm path-length cell maintained at 20 °C. The light source was maintained at 589 nm. The concentration (*c*) is expressed in g/100 mL and specific rotations are denoted $[\alpha]_D$ with implied units of 10^{-1} deg cm^2 g^{-1}.

Infrared spectra were obtained as a thin film on sodium chloride discs or from neat samples using a diamond ATR module. The spectra were recorded on a Bruker Tensor 27 spectrometer and a representative number of absorption maxima are reported in wavenumbers (cm^{-1}).

^{1}H NMR spectra were recorded on Bruker DPX400, AVII400 or AVIII400 (400 MHz) and Bruker DRX500 or AVII500 with cryoprobe (126 MHz) spectrometers using deuterochloroform (unless indicated otherwise) as a reference for internal deuterium lock. The chemical shift data for each signal are given as δ_H in units of parts per million (ppm) relative to tetramethylsilane (TMS) where δ_H (TMS) = 0.00 ppm. The multiplicity of each signal is indicated by: s (singlet); br s (broad singlet); d (doublet); t (triplet); q (quartet); dd (doublet of doublets); dq (doublet of quartets); tt (triplet of triplets); qd (quartet of doublets); ddd (doublet of doublet of doublets); or m (multiplet). The number of protons (n) for a given resonance signal is indicated by nH.

Coupling constants (J) are expressed in Hz and are recorded to the nearest 0.1 Hz. Identical proton coupling constants (J) are averaged in each spectrum and reported to the nearest 0.1 Hz. The coupling constants are determined using Bruker TopSpin software.

^{13}C NMR spectra were recorded on Bruker AVII400 or AVIII400 (101 MHz) and Bruker DRX500 or AVII500 with cryoprobe (126 MHz) spectrometers using broadband proton decoupling and an internal deuterium lock. The chemical shift data for each signal are given as δ_C in units of parts per million (ppm) relative to tetramethylsilane (TMS) where δ_C (TMS) = 0.0 ppm. Where appropriate, coupling constants (J) are quoted in Hz and are recorded to the nearest 0.1 Hz. ^{1}H and ^{13}C spectra were assigned using 2D NMR experiments including COSY, HSQC and HMBC.

^{19}F NMR spectra were recorded on Bruker AVIII400 (377 MHz) and Bruker AVII500 (470 MHz) spectrometers using deuterochloroform (unless indicated otherwise) as a reference for internal deuterium lock. The chemical shift data for each signal are given as δ_F in units of parts per million (ppm) relative to trichlorofluoromethane where δF = 0.00 ppm. ^{19}F spectra with multiple ^{19}F signals were assigned using a HOESY 2D NMR experiment.

Mass spectra were acquired on either a Micromass LCT Premier spectrometer, Agilent 6120 Quadrapole spectrometer or Bruker MicroTOF spectrometer using electrospray ionization, operating in positive or negative mode, from solutions of methanol. *m/z* values are reported in Daltons and followed by their percentage abundance in parentheses.

Elemental analyses were obtained by the microanalysis service of the London Metropolitan University, UK.

High-Performance Liquid Chromatography was carried out using a PerkinElmer Flexar system with a Binary LC pump and UV/VIS LC detector. For determination of compound purity a Dionex Acclaim 120 column (C18, 5 µm, 120 Å, 4.6 × 150 mm) was used with the method described below (Table A). Samples were injected in $(CH_3)_2SO$ or $(CH_3)_2CHOH$. For determination of enantio-purity a Daicel ChiralPak® (AD-H, 5 µm 4.6 × 250 mm) column was used with the method described below (Table B). Samples were injected in $(CH_3)_2CHOH$. Chromera software was used to determine purity and enantiomeric excess from relative peak areas of UV/VIS absorbance at 254 nm.

Table A: Solvents; A = 95%H_2O/5%MeCN + 0.1% TFA. B = 95%MeCN/5%H_2O + 0.1% TFA.

Step lenght (min)	Elapsed time (min)	%A	%B
1	1	100	0
10	11	0	100
3	14	0	100
1	15	100	0
5	20	100	0

Table B: Solvents; A = *n*-hexane. B = $(CH_3)_2CHOH$.

Step lenght (min)	Elapsed time (min)	%A	%B
1	1	90	10
12	13	50	50
10	23	50	50
1	24	90	10
6	30	90	10

General Procedure 1 for coupling of carboxylic acids to amines.

To a solution of carboxylic acid (0.19 mmol, 1.0 eq) and HBTU (81 mg, 0.21 mmol, 1.1 eq) in DMF (1 mL) was added *N*,*N*-diisopropylethylamine (68 μL, 50 mg, 0.39 mmol, 2.0 eq). The reaction mixture was stirred for 10 min, then a solution of the requisite amine (0.21 mmol, 1.1 eq) in DMF (1 mL) was added and stirring continued at rt. Upon completion, the reaction mixture was diluted with EtOAc (50 mL), washed with H_2O (3 × 50 mL) and brine (50 mL), dried (Mg_2SO_4), filtered and concentrated *in vacuo*.

General Procedure 2 for preparation of 1,4-benzoxazin-3-one analogues.

Method 1: A dry 2-5 mL microwave flask was charged with the requisite aminophenol (1.1 eq) and sealed under N_2. DMF (2 mL) was added, followed by 1,8-diazabicyclo[5.4.0]undec-7-ene (1.2 eq) and methyl bromopropionate (1.0 eq). The reaction mixture was heated at 110 °C under microwave irradiation for 90 min, then diluted with EtOAc (20 mL), washed with H_2O (3 × 20 mL), dried ($MgSO_4$), filtered and concentrated *in vacuo*.

Method 2: A dry 2-5 mL microwave flask was charged with the requisite aminophenol (1.1 eq) and sealed under N_2. DMF (2 mL) was added, followed by 1,8-diazabicyclo[5.4.0]undec-7-ene (1.2 eq) and methyl bromopropionate (1.0 eq). The reaction mixture was heated at 110 °C in an oil bath for 18 h, then diluted with EtOAc (20 mL), washed with H_2O (3 × 20 mL), dried ($MgSO_4$), filtered and concentrated *in vacuo*.

General procedure 3 for coupling of benzoxazinone-carboxylic acid 36 to a library of amines.

Stock solutions of carboxylic acid **36** (312 mM in DMF) and HBTU (250 mM in DMF) were prepared. Pre-weighed amines (125 μM, 1.3 eq) in 7.5 mL vials were arranged into the desired plate format. DMF (600 μL) and *N*,*N*-diisopropylethylamine (74 mg, 100 μL, 574 μM, 4.6 eq) were added to each. If required, vials were sonicated and gently heated to aid dissolution. Aliquots of **36** (400 μL, 1.0 eq) and HBTU (400 μL, 1.2 eq) were added sequentially to each amine vial using a multi-dispenser pipette. The reaction mixtures were placed on a rocking platform for 20 h before being placed in a centrifugal evaporator to remove DMF and volatile reagents. To the resulting residue was added CH_2Cl_2 (1 mL) and H_2O (1 mL) and dissolution encouraged through gentle shaking and sonication. Mixtures that remained insoluble were put aside for a separate work-up. The organic components were transferred into a 96-well plate *via* a phase separator and concentrated using a centrifugal evaporator. Conversion of the reaction to the desired product was determined by LC-MS with an ELSD trace. Further purification was not carried out and consequently only samples with a purity > 90% (ELSD) were considered for *in vitro* evaluation.

General Procedure 4 for preparation of alcohols 67-69.

A solution of the requisite quinoline/benzoxazine (190 mg, 1.43 mmol, 1.0 eq) and methyl prop-2-enoate

(258 μL, 247 mg, 2.87 mmol, 2.0 eq) in acetic acid (1 mL) was heated at 120 °C for 16 h. The reaction mixture was concentrated *in vacuo* and azeotroped with methanol (15 mL) to yield the methyl ester as a brown oil that was used without further purification. To a solution of the ester (287 mg, 1.31 mmol, 1.0 eq) in THF (14 mL) at 0 °C was added a solution of $LiAlH_4$ in THF (1M, 1.57 mL, 1.57 mmol, 1.2 eq) dropwise over 5 min. The reaction mixture was allowed to warm to rt and stirred for 23 h before being cooled to 0 °C and quenched by sequential addition of EtOAc (2 mL) and H_2O (1 mL). The resulting mixture was dried ($MgSO_4$), filtered through Celite® (eluent EtOAc) and concentrated *in vacuo*.

General Procedure 5 for preparation of azides 71-73.

To a solution of the requisite alcohol (82 mg, 0.43 mmol, 1.0 eq) in CH_2Cl_2 (3 mL) at 0 °C was added Et_3N (87 mg, 120 μL, 0.86 mmol, 2.0 eq) and stirred for 5 min. Mesyl chloride (74 mg, 50 μL, 0.65 mmol, 1.5 eq) was added and the reaction mixture was stirred at rt for 40 min before being concentrated *in vacuo*. The resulting residue was passed through a plug of silica gel (eluent EtOAc) and concentrated *in vacuo* to give 3-(3,4-dihydroquinolin-1(2*H*)-yl)propyl methanesulfonate (96 mg, 0.36 mmol) as a brown oil. A solution of the sulfonate (96 mg, 0.36 mmol, 1.0 eq) and NaN_3 (93 mg, 1.43 mmol, 4.0 eq) in DMF (3 mL) was heated at 60 °C for 1 h. The reaction mixture was cooled to rt, then diluted with EtOAc (25 mL), washed with H_2O (2 × 25 mL) and brine (25 mL), dried ($MgSO_4$), filtered and concentrated *in vacuo*.

General Procedure 6 for preparation of amines 74-77.

A mixture of the requisite azide (43 mg, 0.20 mmol, 1.0 eq) and Pd/C (11 mg, 0.010 mmol, 0.05 eq) in EtOAc (2 mL) was stirred under a H_2 atmosphere for 30 h. The reaction mixture was filtered through Celite® (eluent EtOAc) and concentrated *in vacuo*.

Benzyl 2-fluoro-3-nitrobenzoate (12)

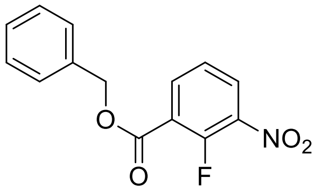

A suspension of 2-fluoro-3-nitrobenzoic acid (4.15 g, 22.4 mmol, 1.0 eq) and K_2CO_3 (4.65 g, 33.1 mmol, 1.5 eq) in DMF (50 mL) was stirred at rt for 5 min, then BnBr (4.01 mL, 5.75 g, 33.1 mmol, 1.5 eq) was added and the reaction mixture stirred at 60 ºC for 3 h. After cooling to rt the suspension was diluted with EtOAc (250 mL), washed with 2 M K_2CO_3 (3 × 250 mL) and brine (250 mL), dried ($MgSO_4$), filtered and concentrated *in vacuo*. Purification *via* silica gel chromatography (1:10 EtOAc:petroleum ether) yielded **12** (5.95 g, 21.6 mmol, 97%) as a pale yellow oil. R_f 0.35 (1:6 EtOAc:petroleum ether); ν_{max}/cm^{-1} 3092 (w), 2361 (w), 1730 (s), 1535 (s), 1350 (m), 1263 (m); ^1H NMR (500 MHz; $CDCl_3$): δ 8.24 (ddd, *J* 7.8 6.2 1.6 Hz, 1H), 8.19 (ddd, *J* 8.2 6.2 1.6 Hz, 1H), 7.49-7.45 (m, 2H), 7.44-7.34 (m, 4H), 5.43 (s, 2H); ^{13}C NMR (126 MHz; $CDCl_3$): δ 162.5 (d, *J* 3.6 Hz), 154.9 (d, *J* 278.3 Hz), 138.7 (d, *J* 8.8 Hz), 137.3, 135.0, 129.9 (d, *J* 1.4 Hz), 128.7, 128.6, 128.3, 124.0 (d, *J* 5.6 Hz), 121.6 (d, *J* 9.7 Hz), 67.9; ^{19}F NMR (377 MHz; $CDCl_3$): δ −116.13 (dd, *J* 6.2 6.2 Hz, 1F); LRMS *m/z* (ES+) 298 ($[M+Na]^+$, 100%); HRMS *m/z* (ES+) [Found: $(M+Na)^+$ 298.0476. $C_{14}H_{10}FNNaO_4^+$ requires 298.0486]; Anal. calcd for $C_{14}H_{10}FNO_4$. C 61.09%, H 3.66%, N 5.09%; Found: C 61.21%, H 3.57%, N 5.23%.

Benzyl 2-{[(2'*R*)-1'-methoxy-1'-oxopropan-2'-yl]amino}-3-nitrobenzoate [(*R*)-13]

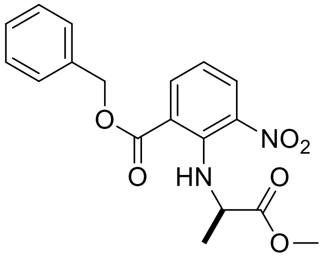

To a solution of **12** (2.50 g, 9.08 mmol, 1.0 eq) in toluene (150 mL) was added Cs_2CO_3 (6.51 g, 20.0 mmol, 2.2 eq) and D-alanine methyl ester hydrochloride (1.39 g, 9.99 mmol, 1.1 eq). The resulting yellow solution was stirred at 85 ºC for 24 h. Upon cooling to rt the reaction mixture was diluted with EtOAc (300 mL), washed with 2 M K_2CO_3 (3 × 250 mL) and brine (250 mL), dried ($MgSO_4$), filtered and concentrated *in vacuo*. Purification *via* silica gel chromatography (1:10 EtOAc:petroleum ether) yielded (*R*)-**13** (2.93 g, 8.18 mmol, 90%) as a yellow oil. R_f 0.56 (1:3 EtOAc:petroleum ether); $[\alpha]_D$ −10.9 (*c* 1.0 in $CHCl_3$); ν_{max}/cm^{-1} 3266 (w), 1745 (m), 1690 (s), 1530 (s), 1344 (m), 1251 (s); ^1H NMR (400 MHz; $CDCl_3$): δ 8.78 (br d, *J* 8.4 Hz, 1H), 8.14 (dd, *J* 7.9 1.7 Hz, 1H), 7.96 (dd, *J* 8.1 1.7 Hz, 1H), 7.48-7.33 (m, 5H), 6.77 (dd, *J* 8.1 7.9, Hz 1H), 5.36 (s, 2H), 4.05 (dq, *J* 8.4 7.0 Hz, 1H), 3.69 (s, 3H), 1.43 (d, *J* 7.0 Hz, 3H); ^{13}C NMR (101 MHz; $CDCl_3$): δ

173.5, 166.8, 144.1, 138.8, 137.1, 135.3, 131.3, 128.7, 128.6, 128.5, 118.4, 116.3, 67.3, 54.0, 52.6, 19.1; LRMS *m/z* (ES+) 359 ($[M+H]^+$, 100%); HRMS *m/z* (ES+) [Found: $(M+Na)^+$ 381.1071. $C_{18}H_{18}N_2NaO_6^+$ requires 381.1057]; Anal. calcd for $C_{18}H_{18}N_2O_6$: C 60.33%, H 5.06%, N 7.82%; Found: C 60.37%, H 4.97%, N 7.74%.

Benzyl 2-{[(2'*S*)-1'-methoxy-1'-oxopropan-2'-yl]amino}-3-nitrobenzoate [(*S*)-13]

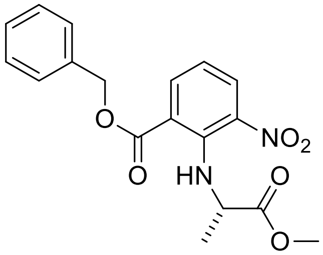

Compound **12** (305 mg, 1.11 mmol, 1.0 eq) and L-alanine methyl ester hydrochloride (186 mg, 1.33 mmol, 1.2 eq) were reacted as described above for 4.5 h. Purification *via* silica gel chromatography (gradient elution 12 to 100% CH_2Cl_2 in petroleum ether) yielded (*S*)-**13** (212 mg, 0.592 mmol, 53%) as a yellow oil. R_f 0.38 (1:1 CH_2Cl_2:petroleum ether); $[\alpha]_D$ +4.4 (*c* 1.0 in $CHCl_3$); ^{1}H NMR (400 MHz; $CDCl_3$): δ 8.77 (br d, *J* 8.4 Hz, 1H), 8.14 (dd, *J* 7.9 1.7 Hz, 1H), 7.97 (dd, *J* 8.1 1.7 Hz, 1H), 7.48-7.33 (m, 5H), 6.77 (dd, *J* 8.1 7.9 Hz, 1H), 5.37 (s, 2H), 4.04 (dq, J 8.4 6.9 Hz, 1H), 3.70 (s, 3H), 1.43 (d, *J* 6.9 Hz, 3H); LRMS *m/z* (ES+) 359 ($[M+H]^+$, 100%). These data are in accordance with the enantiomer (*R*)-**13**.

Benzyl (3*R*)-3-methyl-2-oxo-1,2,3,4-tetrahydroquinoxaline-5-carboxylate [(*R*)-14]

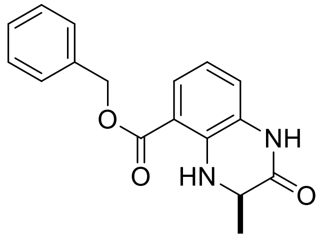

To a solution of (*R*)-**13** (106 mg, 0.296 mmol, 1.0 eq) in DMF (6 mL) was added zinc dust (484 mg, 7.40 mmol, 25 eq) and NH_4Cl (396 mg, 7.40 mmol, 25 eq). The reaction mixture was stirred at rt for 24 h, then diluted with EtOAc (50 mL) and filtered through Celite® (eluent EtOAc). The filtrate was washed with brine (3 × 50 mL), dried ($MgSO_4$), filtered and concentrated *in vacuo*. Purification *via* silica gel chromatography (1:4 to 1:2 EtOAc:petroleum ether) yielded (*R*)-**14** (84 mg, 0.28 mmol, 96%) as a colorless solid. R_f 0.40 (1:1 EtOAc:petroleum ether); mp 199-200 °C (from EtOAc); $[\alpha]_D$ −48.4 (*c* 1.0 in $CHCl_3$); ν_{max}/cm^{-1} 3354 (w), 3065 (w), 1687 (s), 1498 (m), 1239 (s); ^{1}H NMR (500 MHz; $CDCl_3$): δ 8.80 (br s, 1H), 7.65 (d, *J* 7.8 Hz, 1H), 7.60 (br s, 1H), 7.51-7.33 (m, 5H), 6.87 (d, *J* 7.5 Hz, 1H), 6.65 (dd, *J* 7.8 7.5, Hz 1H), 5.34 (s, 2H), 4.24-4.14 (m, 1H), 1.52 (d, *J* 6.5 Hz, 3H); ^{13}C NMR (126 MHz; $CDCl_3$): δ 168.6, 167.7, 137.8,

135.9, 128.6, 128.3, 128.0, 126.0, 125.8, 119.1, 116.6, 111.2, 66.4, 51.1, 18.9; LRMS *m/z* (ES+) 615 ($[2M+Na]^+$, 100%); HRMS *m/z* (ES+) [Found: $(M+Na)^+$ 319.1051. $C_{17}H_{16}N_2NaO_3^+$ requires 319.1053]; Anal. calcd for $C_{17}H_{16}N_2O_3$: C 68.91%, H 5.44%, N 9.45%; Found: C 69.01%, H 5.39%, N 9.39%.

Benzyl (3*S*)-3-methyl-2-oxo-1,2,3,4-tetrahydroquinoxaline-5-carboxylate [(*S*)-14]

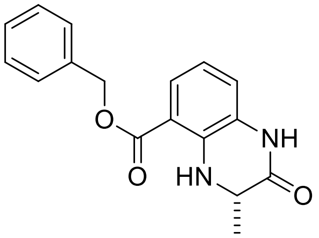

Compound (*S*)-**13** (186 mg, 0.512 mmol, 1.0 eq) was reacted as described above for 24 h. Purification *via* silica gel chromatography (gradient elution 5 to 80% EtOAc in petroleum ether) yielded (*S*)-**14** (134 mg, 0.452 mmol, 88%) as a colorless solid. R_f 0.29 (1:2 EtOAc:petroleum ether); mp 204-206 °C (from EtOAc); $[\alpha]_D$ +42.0 (*c* 1.0 in $CHCl_3$); ^1H NMR (400 MHz; $CDCl_3$): δ 7.83 (br s, 1H), 7.65 (d, *J* 8.0 1.1 Hz, 1H), 7.59 (br s, 1H), 7.46-7.32 (m, 5H), 6.80 (d, *J* 7.7 1.1 Hz, 1H), 6.65 (dd, *J* 8.0 7.7 Hz, 1H), 5.34 (d, *J* 1.0 Hz, 2H), 4.16 (qd, *J* 6.8 1.3 Hz, 1H), 1.51 (d, *J* 6.8 Hz, 3H); LRMS *m/z* (ES+) 297 ($[M+H]^+$, 100%). These data are in accordance with the enantiomer (*R*)-**14**.

(*R*)-3-Methyl-2-oxo-1,2,3,4-tetrahydroquinoxaline-5-carboxylic acid [(*R*)-15]

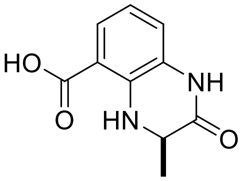

A mixture of (*R*)-**14** (1.01 g, 3.41 mmol, 1.0 eq) and Pd/C (72 mg, 0.068 mmol, 0.02 eq) in EtOAc (250 mL) was placed under a H_2 atmosphere. The reaction mixture was stirred for 16 h then diluted with EtOAc (250 mL), filtered through Celite® (eluent EtOAc) and the filtrate retained. The filter cake was resuspended in EtOAc (500 mL), stirred for 3 h, then filtered through Celite®, combined with the previous filtrate and concentrated *in vacuo* to give (*R*)-**15** (686 mg, 3.33 mmol, 98%) as a colorless solid that was used without further purification. R_f 0.30 (EtOAc); mp 230 °C (dec.); $[\alpha]_D$ −124.6 (*c* 0.5 in MeOH); ν_{max}/cm^{-1} 2972 (br), 1666 (s), 1420 (m), 1231 (s); ^1H NMR (500 MHz; $(CD_3)_2SO$): δ 12.89 (br s, 1H), 10.44 (s, 1H), 7.61 (s, 1H), 7.43 (dd, *J* 8.0 1.3 Hz, 1H), 6.93 (dd, *J* 7.7 1.3 Hz, 1H), 6.63 (dd, *J* 8.0 7.7 Hz, 1H), 4.05 (q, *J* 6.7 Hz, 1H), 1.30 (d, *J* 6.7 Hz, 3H); ^{13}C NMR (500 MHz; $(CD_3)_2SO$): δ 169.7, 167.8, 137.6, 127.4, 125.2, 118.8, 116.6, 111.8, 50.6, 19.0; LRMS *m/z* (ES+) 207 ($[M+H]^+$, 100%); HRMS *m/z* (ES+) [Found: $(M+Na)^+$ 229.0592. $C_{10}H_{10}N_2NaO_3^+$ requires 229.0584].

(*S*)-3-Methyl-2-oxo-1,2,3,4-tetrahydroquinoxaline-5-carboxylic acid [(*S*)-15]

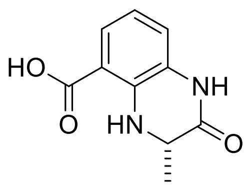

Compound (*S*)-**14** (121 mg, 0.408 mmol, 1.0 eq) was reacted as described above to afford (*S*)-**15** (84 mg, 0.407 mmol, 99%) as a colorless solid that was used without further purification. R_f 0.27 (EtOAc); mp 227 °C (dec.); $[\alpha]_D$ +127.8 (*c* 0.5 in MeOH); ^{1}H NMR (500 MHz; $(CD_3)_2SO$): δ 12.93 (br s, 1H), 10.44 (s, 1H), 7.63 (s, 1H), 7.41 (dd, *J* 7.9 1.4 Hz, 1H), 6.91 (dd, *J* 7.7 1.4 Hz, 1H), 6.62 (dd, *J* 7.9 7.7 Hz, 1H), 4.03 (q, *J* 6.7 Hz, 1H), 1.29 (d, *J* 6.7 Hz, 3H); LRMS *m/z* (ES−) 205 ($[M-H]^-$, 100%). These data are in accordance with the enantiomer (*R*)-**15**.

Benzyl 2,6-difluoro-3-nitrobenzoate (17)

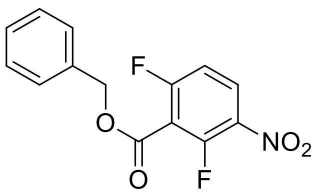

A 2 mL microwave vial was charged with 2,6-difluoro-3-nitrobenzoic acid (100 mg, 0.492 mmol, 1.0 eq), K_2CO_3 (206 mg, 1.49 mmol, 3.0 eq) and DMF (1.5 mL). The vial was sealed and the mixture stirred at rt for 5 min before BnBr (178 µL, 255 mg, 1.49 mmol, 3.0 eq) was added *via* syringe. The reaction mixture was stirred at 110 °C for 30 min before being cooled to rt and stirred for an additional 16 h. The mixture was diluted with EtOAc (25 mL), washed with 2 M K_2CO_3 (3 × 25 mL) and brine (25 mL), dried ($MgSO_4$), filtered and concentrated *in vacuo*. Purification *via* silica gel chromatography (gradient elution 2 to 20% EtOAc in petroleum ether) gave **17** (86 mg, 0.29 mmol, 59%) as a waxy yellow solid. R_f 0.32 (1:10 EtOAc:petroleum ether); mp 74 °C; ν_{max}/cm^{-1} 3095 (w), 1736 (s), 1537 (s), 1351 (s), 1292 (s); ^{1}H NMR (400 MHz; $CDCl_3$): δ 8.24 (ddd, *J* 9.4 8.2 5.5 Hz, 1H), 7.49-7.35 (m, 5H), 7.12 (ddd, *J* 9.4 7.9 1.7 Hz, 1H), 5.44 (s, 2H); ^{13}C NMR (126 MHz; $CDCl_3$): δ 163.2 (dd, *J* 267.0 5.6 Hz), 159.5, 154.7 (dd, *J* 274.8 7.4 Hz), 134.6, 134.5 (br), 129.5 (d, *J* 11.6 Hz), 128.93, 128.88, 128.6, 113.8 (dd, *J* 19.9 18.4 Hz), 112.8 (dd, *J* 23.7 4.5 Hz), 68.7; ^{19}F NMR (377 MHz; $CDCl_3$): δ −97.9 (ddd, *J* 9.5 7.9 5.5 Hz, 1F), −112.5 (ddd, *J* 9.5 8.2 1.7 Hz, 1F); LRMS *m/z* (ES+) 316 ($[M+Na]^+$, 50%), 404 (100%); HRMS *m/z* (ES+) [Found: $(M+Na)^+$ 316.0394. $C_{14}H_9F_2NNaO_4^+$ requires 316.0392]; Anal. calcd for $C_{14}H_9F_2NO_4$: C 57.35%, H 3.09%, N 4.78%; Found: C 57.49%, H 2.98%, N 4.80%.

Benzyl 6-fluoro-2-{[(2*R*)-1-methoxy-1-oxopropan-2-yl]amino}-3-nitrobenzoate [(*R*)-18]

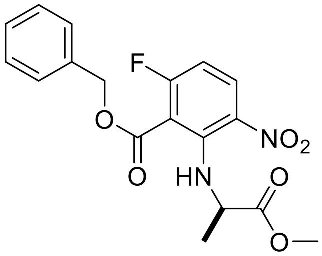

A solution of **17** (261 mg, 0.890 mmol, 1.0 eq), D-alanine methyl ester hydrochloride (124 mg, 0.890 mmol, 1.0 eq) and Cs_2CO_3 (580 mg, 1.78 mmol, 2.0 eq) was heated to 85 °C, causing a deep yellow color to develop, and stirring was continued for 13 h. The reaction mixture was cooled to rt, diluted with EtOAc (200 mL), washed with 2 M K_2CO_3 (3 × 100 mL) and brine (100 mL), dried ($MgSO_4$), filtered and concentrated *in vacuo*. Purification *via* silica gel chromatography (gradient elution 1 to 15% EtOAc in petroleum ether) yielded (*R*)-**18** (288 mg, 0.765 mmol, 86%) as a yellow oil. R_f 0.45 (1:4 EtOAc:petroleum ether); $[\alpha]_D$ −96.1 (*c* 1.0 in $CHCl_3$); ν_{max}/cm^{-1} ν_{max}/cm^{-1} 3328 (w), 2956 (w), 1739 (s), 1614 (s), 1500 (s), 1261 (s); ^1H NMR (400 MHz; $CDCl_3$): δ 8.53 (br d, *J* 8.6 Hz, 1H), 8.26 (dd, *J* 9.4 6.5 Hz, 1H), 7.50-7.43 (m, 2H), 7.43-7.33 (m, 3H), 6.52 (dd *J* 9.4 7.6 Hz, 1H), 5.43 (d, *J* 12.1 Hz, 1H), 5.33 (d, *J* 12.1 Hz, 1H), 4.07 (qd, *J* 8.6 7.0 Hz, 1H), 3.65 (s, 3H), 1.38 (d, *J* 6.9 Hz, 3H); ^{13}C NMR (101 MHz; $CDCl_3$): δ 172.7, 164.5 (d, *J* 260.5 Hz), 164.3, 144.1 (d, *J* 7.9 Hz), 134.4, 132.8, 131.1 (d, *J* 12.8 Hz), 128.84, 128.82, 128.76, 110.6 (d, *J* 21.2 Hz), 105.4 (d, *J* 25.1 Hz), 68.5, 53.5, 52.7, 19.5; ^{19}F NMR (470 MHz; $CDCl_3$): δ −99.9 (dd, *J* 7.6 6.5 Hz, 1F); LRMS *m/z* (ES+) 377 ($[M+H]^+$, 100%); HRMS *m/z* (ES+) [Found: $(M+Na)^+$ 399.0962. $C_{18}H_{17}FN_2NaO_6^+$ requires 399.0963]; Anal. calcd for $C_{18}H_{17}FN_2O_6$: C 57.45%, H 4.55%, N 7.44%; Found: C 57.36%, H 4.48%, N 7.52%.

Benzyl (3*R*)-6-fluoro-3-methyl-2-oxo-1,2,3,4-tetrahydroquinoxaline-5-carboxylate [(*R*)-19]

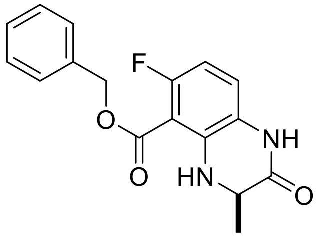

To a solution of (*R*)-**18** (265 mg, 0.704 mmol, 1.0 eq) in DMF (14 mL) was added zinc dust (1.15 g, 17.6 mmol, 25 eq) and NH_4Cl (1.88 g, 35.2 mmol, 50 eq). The reaction mixture was stirred at rt for 25 h, then diluted with EtOAc (150 mL) and filtered through Celite® (eluent EtOAc). The filtrate was washed with brine (3 × 150 mL), dried ($MgSO_4$), filtered and concentrated *in vacuo*. Purification *via* silica gel chromatography (1:6 EtOAc:petroleum ether) yielded (*R*)-**19** (140 mg, 0.445 mmol, 63%) as a colorless solid. R_f 0.10 (1:4 EtOAc:petroleum ether); mp 162-163 °C (from $CHCl_3$); $[\alpha]_D$ −30.3 (*c* 0.9 in $CHCl_3$); ν_{max}/cm^{-1} 3320 (w), 2361 (w), 1683 (s), 1312 (m), 1232 (s); ^1H NMR (500 MHz; $(CD_3)_2CO$): δ 9.43 (br s, 1H), 7.55 (br s, 1H),

7.50 (d, J 7.4 Hz, 2H), 7.40 (dd, J 7.4 7.4 Hz, 2H), 7.37-7.31 (m, 1H), 7.00 (dd, J 8.6 5.1 Hz, 1H), 6.45 (dd, J 11.3 8.6 Hz, 1H), 5.40 (s, 2H), 4.11 (qd, J 6.7 1.6 Hz, 1H), 1.40 (d, J 6.7 Hz, 3H); ^{13}C NMR (126 MHz; $(CD_3)_2CO$): δ 167.6, 166.9 (d, J 4.0 Hz), 159.3 (d, J 251.4 Hz), 139.1 (d, J 4.5 Hz), 137.1, 129.3, 128.9, 128.7, 124.4 (d, J 2.8 Hz), 119.3 (d, J 11.0 Hz), 104.4 (d, J 25.7 Hz), 102.4 (d, J 15.9 Hz), 67.2, 51.5, 18.9; ^{19}F NMR (377 MHz; $(CD_3)_2CO$): δ −114.4 (dd, J 11.3 5.1 Hz, 1F); LRMS *m/z* (ES−) 313 ($[M{-}H]^-$, 100%); HRMS *m/z* (ES+) [Found: $(M+Na)^+$ 337.0968. $C_{17}H_{15}FN_2NaO_3^+$ requires 337.0959].

(3*R*)-6-Fluoro-3-methyl-2-oxo-1,2,3,4-tetrahydroquinoxaline-5-carboxylic acid [(*R*)-20]

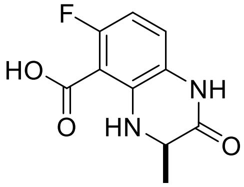

A mixture of (*R*)-**19** (126 mg, 0.401 mmol, 1.0 eq) and Pd/C (21 mg, 0.020 mmol, 0.05 eq) in EtOAc (30 mL) was placed under a H_2 atmosphere. The reaction mixture was stirred for 18 h, then diluted with EtOAc (60 mL), filtered through Celite® (eluent EtOAc) and concentrated *in vacuo* to give (*R*)-**20** (85 mg, 0.38 mmol, 95%) as a colorless solid, which was used without further purification. R_f 0.55 (1:99 AcOH:EtOAc); mp 222-226 °C (from EtOAc); $[\alpha]_D$ −92.2 (*c* 0.5 in MeOH); ν_{max}/cm^{-1} 3310 (w), 2973 (br), 1684 (s), 1488 (m), 1205 (m); ^{1}H NMR (500 MHz; $(CD_3)_2SO$): δ 13.30 (br s, 1H), 10.40 (s, 1H), 7.49 (s, 1H), 6.84 (dd, J 8.6 4.8 Hz, 1H), 6.44 (dd, J 11.1 8.6 Hz, 1H), 4.00 (q, J 6.7 Hz, 1H), 1.27 (d, J 6.7 Hz, 3H); ^{13}C NMR (126 MHz; $(CD_3)_2SO$): δ 167.6 (d, J 2.7 Hz), 169.4, 158.1 (d, J 249.0 Hz), 137.7 (d, J 3.9 Hz), 123.4 (d, J 2.3 Hz), 118.24 (d, J 11.1 Hz), 103.7 (d, J 103.7 Hz), 102.6 (d, J 15.8 Hz), 50.4, 18.8; ^{19}F NMR (470 MHz; $(CD_3)_2SO$): δ −114.6 (br s, 1F); LRMS *m/z* (ES−) 223 ($[M{-}H]^-$, 100%); HRMS *m/z* (ES+) [Found: $(M+Na)^+$ 247.0501. $C_{10}H_9FN_2NaO_3^+$ requires 247.0489].

Benzyl 2-(benzyloxy)-5-fluoro-3-nitrobenzoate (22)

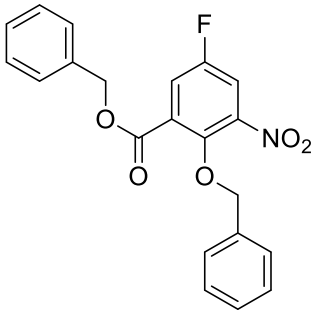

To a solution of 5-fluoro-2-hydroxybenzoic acid (1.39 g, 8.90 mmol, 1.0 eq) at 0 °C in H_2SO_4 (98%, 15 mL) was added a solution of HNO_3 (70% w/w, 882 µL, 1.25 g, 13.8 mmol, 1.6 eq) in H_2SO_4 (3 mL) over 5 min. The reaction mixture was stirred at 0 °C for 2 h and then quenched by pouring onto crushed ice (200 mL). The

resulting yellow precipitate was extracted with EtOAc (3 × 100 mL), the combined organics were washed with brine (100 mL), dried ($MgSO_4$), filtered and concentrated *in vacuo* to give 5-fluoro-2-hydroxy-3-nitrobenzoic acid as a yellow solid (1.54 g, 7.66 mmol) that was used without further purification. A 5 mL microwave vial was charged with the carboxylic acid (200 mg, 0.994 mmol, 1.0 eq), K_2CO_3 (412 mg, 2.98 mmol, 3.0 eq) and DMF (3 mL). The vial was sealed and the mixture stirred at rt for 5 min before BnBr (355 µL, 510 mg, 2.98 mmol, 3.0 eq) was added *via* syringe. The reaction mixture was stirred at 110 °C for 1 h before being cooled to rt and stirred for an additional 48 h. The mixture was diluted with EtOAc (50 mL), washed with 2 M K_2CO_3 (3 × 50 mL) and brine (50 mL), dried ($MgSO_4$), filtered and concentrated *in vacuo*. Purification *via* silica gel chromatography (gradient elution 1 to 14% EtOAc in petroleum ether) gave **22** (173 mg, 0.454 mmol, 40%) as a yellow oily solid. R_f 0.16 (1:12 EtOAc:petroleum ether); mp 72-73 °C (from Et_2O); ν_{max}/cm^{-1} 3089 (w), 1734 (s), 1539 (s), 1378 (m), 1247 (m); ^1H NMR (500 MHz; $CDCl_3$): δ 7.80 (dd, *J* 7.9 3.3 Hz, 1H), 7.69 (dd, *J* 7.0 3.3 Hz, 1H), 7.43-7.33 (m, 10H), 5.36 (s, 2H), 5.12 (s, 2H); ^{13}C NMR (126 MHz; $CDCl_3$): δ 163.1 (d, *J* 1.9 Hz), 156.8 (d, *J* 250.0 Hz), 148.0 (d, *J* 3.7 Hz), 146.0 (d, *J* 8.5 Hz), 135.4, 134.8, 129.6 (d, *J* 7.4 Hz), 128.8 – 128.7 (Ar-C), 128.65, 128.61, 128.5, 122.6 (d, *J* 24.5 Hz), 116.0 (d, *J* 27.0 Hz), 78.9, 68.0; ^{19}F NMR (377 MHz; $CDCl_3$): δ −113.48 (dd, *J* 7.9 7.0 Hz, 1F); LRMS *m/z* (ES+) 404 ($[M+Na]^+$, 100%); HRMS *m/z* (ES+) [Found: $(M+Na)^+$ 404.0904. $C_{21}H_{16}FNNaO_5^+$ requires 404.0905]; Anal. calcd for $C_{21}H_{16}FNO_5$: C 66.14%, H 4.23%, N 3.67%; Found: C 66.04%, H 4.29%, N 3.72%.

Benzyl 5-fluoro-2-hydroxy-3-nitrobenzoate (23)

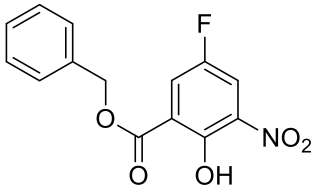

A solution of **22** (996 mg, 2.61 mmol, 1.0 eq) in trifluoroacetic acid (13 mL) and toluene (13 mL) was stirred at rt for 23 h. The reaction mixture was concentrated *in vacuo* and azeotroped with toluene (2 × 50 mL). Purification *via* crystalisation from Et_2O yielded **23** (449 mg, 1.54 mmol, 59%) as yellow needles. R_f 0.48 (1:4 EtOAc:petroleum ether); mp 58 °C (from Et_2O); ν_{max}/cm^{-1} 3092 (w), 1687 (m), 1534 (s), 1396 (m), 1208 (s); ^1H NMR (400 MHz; $CDCl_3$): δ 11.7 (s, 1H), 7.93 (dd, *J* 7.7 3.3 Hz, 1H), 7.90 (dd, *J* 7.8 3.3 Hz, 1H), 7.49-7.38 (m, 5H), 5.45 (s, 2H); ^{13}C NMR (126 MHz; $CDCl_3$): δ 167.7 (d, *J* 2.5 Hz), 153.1 (d, *J* 244.0 Hz), 152.4 (d, *J* 2.2 Hz), 137.8 (d, *J* 7.6 Hz), 134.2, 129.3, 129.0, 128.9, 122.7 (d, *J* 23.8 Hz), 119.0 (d, *J* 26.7 Hz), 116.6 (d, *J* 6.7 Hz), 68.7; ^{19}F NMR (377 MHz; $CDCl_3$): δ −121.0 (dd, *J* 7.8 7.7 Hz, 1F); LRMS *m/z* (ES+) 314 ($[M+Na]^+$, 100%); HRMS *m/z* (ES+) [Found: $(M+Na)^+$ 314.0431. $C_{14}H_{10}FNNaO_5^+$ requires 314.0435].

Benzyl 5-fluoro-3-nitro-2-{[(trifluoromethyl)sulfonyl]oxy}benzoate (24)

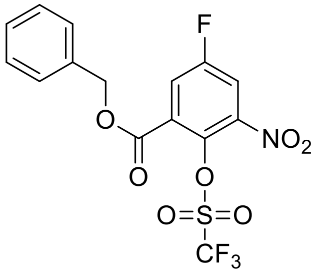

To a solution of **23** (230 mg, 0.790 mmol, 1.0 eq) and Et_3N (120 mg, 165 μL, 1.18 mmol, 1.5 eq) in CH_2Cl_2 (8 mL) at 0 °C was added trifluoromethanesulfonic anhydride (267 mg, 159 μL, 0.947 mmol, 1.2 eq) dropwise over 5 min. The reaction mixture was allowed to warm to rt, stirred for a further 1.5 h, then concentrated *in vacuo* and purified by passing through a short path of silica gel (eluent 1:1 EtOAc:petroleum ether) to give **24** (279 mg, 0.659 mmol, 83%) as a colorless oil. R_f 0.51 (1:4 EtOAc:petroleum ether); ν_{max}/cm^{-1} 3094 (w), 1737 (s), 1552 (s), 1433 (m), 1306 (m), 1222 (s); ^1H NMR (500 MHz; $CDCl_3$): δ 7.99 (dd, *J* 7.1 3.3 Hz, 1H), 7.94 (dd, *J* 6.8 3.3 Hz, 1H), 7.48-7.43 (m, 2H), 7.43-7.36 (m, 3H), 5.43 (s, 2H); ^{13}C NMR (126 MHz; $CDCl_3$): δ 161.6, 159.7 (d, *J* 256.8 Hz), 143.9 (d, *J* 8.1 Hz), 136.5 (d, *J* 4.5 Hz), 134.2, 129.6 (d, *J* 7.5 Hz), 129.2, 128.9, 123.9 (d, *J* 24.8 Hz), 118.5 (q, *J* 321.1 Hz), 117.6 (d, *J* 27.8 Hz), 69.1; ^{19}F NMR (377 MHz; $CDCl_3$): δ −73.3 (s, 3F), −105.9 (dd, *J* 7.1 6.8 Hz, 1F); LRMS *m/z* (ES+) 446 ($[M+Na]^+$, 100%); HRMS *m/z* (ES+) [Found: $(M+Na)^+$ 445.9927. $C_{15}H_9F_4NNaO_7S^+$ requires 445.9928].

Benzyl 5-fluoro-2-{[(2*R*)-1-methoxy-1-oxopropan-2-yl]amino}-3-nitrobenzoate [(*R*)-25]

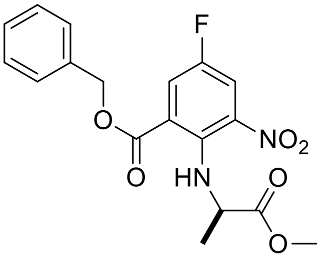

A solution of **24** (397 mg, 0.938 mmol, 1.0 eq), D-alanine methyl ester hydrochloride (144 mg, 1.03 mmol, 1.1 eq) and Cs_2CO_3 (672 mg, 2.06 mmol, 2.2 eq) was heated to 85 °C, causing a deep orange color to develop, and stirring was continued for 13 h. The reaction mixture was cooled to rt, diluted with EtOAc (200 mL), washed with 2 M K_2CO_3 (3 × 100 mL) and brine (100 mL), dried ($MgSO_4$), filtered and concentrated *in vacuo*. Purification *via* silica gel chromatography (1:10 EtOAc:petroleum ether) yielded (*R*)-**25** (285 mg, 0.757 mmol, 81%) as a yellow oil. R_f 0.30 (1:10 EtOAc:petroleum ether); $[\alpha]_D$ +25.0 (*c* 1.0 in $CHCl_3$); ν_{max}/cm^{-1} 3281 (w), 2360 (w), 1744 (s), 1703 (m), 1539 (s), 1342 (m), 1201 (s); ^1H NMR (500 MHz; $CDCl_3$): δ 8.37 (br s, 1H), 7.90 (dd, *J* 8.2 3.2 Hz, 1H), 7.77 (dd, *J* 7.6 3.2 Hz, 1H), 7.48-7.36 (m, 5H), 5.38 (s, 2H), 3.99 (q, *J* 6.9 Hz, 1H), 3.68 (s, 3H), 1.44 (d, *J* 6.9 Hz, 3H); ^{13}C NMR (126 MHz; $CDCl_3$): δ 173.4, 165.6 (d, *J* 2.6 Hz), 152.4 (d,

J 241.7 Hz), 141.2 (d, *J* 1.6 Hz), 138.7 (d, *J* 7.6 Hz), 134.9, 128.8-128.7 (Ar-C), 128.6, 124.2 (d, *J* 24.1 Hz), 120.0 (d, *J* 6.1 Hz), 118.0 (d, *J* 26.6 Hz), 67.7, 54.4, 52.5, 19.0; ^{19}F NMR (377 MHz; $CDCl_3$): δ −124.29 (dd, *J* 8.2 7.6 Hz, 1F); LRMS *m/z* (ES+) 377 ($[M+H]^+$, 100%); HRMS *m/z* (ES+) [Found: $(M+Na)^+$ 399.0962. $C_{18}H_{17}FN_2NaO_6^+$ requires 399.0963]; Anal. calcd for $C_{18}H_{17}FN_2O_6$: C 57.45%, H 4.55%, N 7.44%; Found: C 57.56%, H 4.59%, N 7.32%.

Benzyl (3*R*)-7-fluoro-3-methyl-2-oxo-1,2,3,4-tetrahydroquinoxaline-5-carboxylate [(*R*)-26]

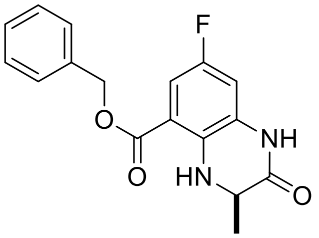

To solution of (*R*)-**25** (261 mg, 0.694 mmol, 1.0 eq) in DMF (14 mL) was added zinc dust (1.13 g, 17.3 mmol, 25 eq) and NH_4Cl (1.85 g, 34.5 mmol, 50 eq). The reaction mixture was stirred at rt for 19 h, then diluted with EtOAc (100 mL) and filtered through Celite® (eluent EtOAc). The filtrate was washed with brine (3 × 100 mL), dried ($MgSO_4$), filtered and concentrated *in vacuo*. Purification *via* silica gel chromatography (1:6 EtOAc:petroleum ether) yielded (*R*)-**26** (161 mg, 0.512 mmol, 74%) as a pale yellow solid. R_f 0.60 (1:1 EtOAc:petroleum ether); mp 191-192 °C (from $CHCl_3$); $[\alpha]_D$ −47.9 (*c* 1.0 in $CHCl_3$); ν_{max}/cm^{-1} 3341 (w), 2969 (w), 1678 (s), 1319 (m), 1217 (s); ^{1}H NMR (500 MHz; $(CD_3)_2CO$): δ 9.57 (br s, 1H), 7.54-7.49 (m, 2H), 7.48 (br s, 1H), 7.44-7.40 (m, 2H), 7.39-7.34 (m, 1H), 7.25 (dd, *J* 9.6 2.9 Hz, 1H), 6.90 (dd, *J* 9.2 2.9 Hz, 1H), 5.37 (s, 2H), 4.12 (qd, *J* 6.7 1.6 Hz, 1H), 1.41 (d, *J* 6.7 Hz, 3H); ^{13}C NMR (126 MHz; $(CD_3)_2CO$): δ 168.4, 167.5 (d, *J* 2.9 Hz), 154.8 (d, *J* 233.3 Hz), 137.2, 135.8, 130.0 (d, *J* 10.4 Hz), 129.4, 129.10, 129.05, 111.6 (d, *J* 8.4 Hz), 109.5 (d, *J* 23.6 Hz), 107.7 (d, *J* 27.9 Hz), 67.2, 51.7, 18.8; ^{19}F NMR (470 MHz; $(CD_3)_2CO$): δ −127.9 (dd, *J* 9.6 9.2 Hz, 1F); LRMS *m/z* (ES+) 315 ($[M+H]^+$, 100%); HRMS *m/z* (ES+) [Found: $(M+Na)^+$ 337.0965. $C_{17}H_{15}FN_2NaO_3^+$ requires 337.0959].

(3*R*)-7-Fluoro-3-methyl-2-oxo-1,2,3,4-tetrahydroquinoxaline-5-carboxylic acid [(*R*)-27]

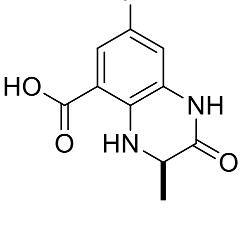

A mixture of (*R*)-**26** (136 mg, 0.433 mmol, 1.0 eq) and Pd/C (23 mg, 0.022 mmol, 0.05 eq) in EtOAc (30 mL) was placed under a H_2 atmosphere. The reaction mixture was stirred for 18 h, then diluted with EtOAc (60 mL), filtered through Celite® (eluent EtOAc) and concentrated *in vacuo* to give (*R*)-**27** (63 mg, 0.28 mmol,

65%) as a yellow solid. R_f 0.58 (1:99 AcOH:EtOAc); mp 240 °C (dec.); $[\alpha]_D$ −78.8 (*c* 0.4 in MeOH); ν_{max}/cm^{-1} 3343 (w), 2923 (br), 1688 (s), 1668 (s), 1315 (m), 1231 (s); ^{1}H NMR (500 MHz; $(CD_3)_2SO$): δ 13.3 (br s, 1H), 10.56 (s, 1H), 7.51 (br s, 1H), 7.13 (dd, *J* 9.5 3.0 Hz, 1H), 6.77 (dd, *J* 9.3 3.0 Hz, 1H), 4.03 (q, *J* 6.7 Hz, 1H), 1.29 (d, *J* 6.7 Hz, 3H); ^{13}C NMR (126 MHz; $(CD_3)_2SO$): δ 168.4 (d, *J* 2.7 Hz), 167.9, 153.1 (d, *J* 232.0), 134.2, 128.6 (d, *J* 10.7 Hz), 111.8 (d, *J* 7.7 Hz), 108.9 (d, *J* 23.0 Hz), 106.2 (d, *J* 27.6 Hz), 50.2, 18.3; ^{19}F NMR (470 MHz; $(CD_3)_2SO$): δ −126.7 (dd, *J* 9.5 9.3 Hz, 1F); LRMS *m/z* (ES−) 223 ($[M-H]^-$, 100%); HRMS *m/z* (ES+) [Found: $(M+Na)^+$ 247.0492. $C_{10}H_9FN_2NaO_3^+$ requires 247.0489].

(3*R*)-*N*-[3-(3,4-Dihydroquinolin-1(2*H*)-yl)propyl]-3-methyl-2-oxo-1,2,3,4-tetrahydroquinoxaline-5-carboxamide [(*R*)-1]

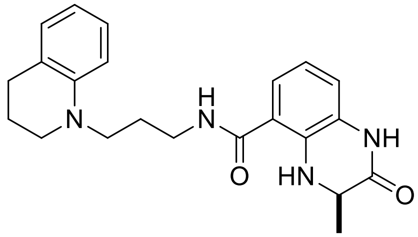

Compounds (*R*)-**15** (40 mg, 0.19 mmol, 1.0 eq) and **75** (47 mg, 0.21 mmol, 1.1 eq) were reacted according to general procedure 1 for 18 h. Purification *via* silica gel chromatography (gradient elution 8 to 100% EtOAc in petroleum ether) gave (*R*)-**1** (23 mg, 0.061 mmol, 32%) as a brown oil. R_f 0.50 (2:1 EtOAc:petroleum ether); ν_{max}/cm^{-1} 3328 (br), 2925 (s), 2854 (m), 1682 (s), 1601 (m), 1505 (m), 1288 (s); $[\alpha]_D$ −23.8 (*c* 0.5 in $(CH_3)_2CO$); ^{1}H NMR (400 MHz; $(CD_3)_2CO$): δ 9.34 (br s, 1H), 8.03 (br s, 1H), 7.80 (br s, 1H), 7.30 (dd, *J* 8.0 1.3 Hz, 1H), 7.00-6.90 (m, 2H), 6.88-6.84 (m, 1H), 6.65 (dd, *J* 8.0 7.9 Hz, 1H), 6.64-6.60 (m, 1H), 6.46 (ddd, *J* 7.3 7.3 1.0 Hz, 1H), 4.02 (qd, *J* 6.7 1.6 Hz, 1H), 3.51-3.43 (m, 2H), 3.39 (t, *J* 7.4 Hz, 2H), 3.31 (t, *J* 5.6 Hz, 2H), 2.71 (t, *J* 6.4 Hz, 2H), 1.97-1.87 (m, 4H), 1.39 (d, *J* 6.7 Hz, 3H); ^{13}C NMR (126 MHz; $(CD_3)_2CO$): δ 169.4, 168.7, 146.2, 137.4, 129.8, 128.8, 127.7, 123.1, 122.2, 117.8, 117.5, 116.9, 116.3, 111.5, 51.6, 50.1, 49.7, 38.2, 28.8, 27.2, 23.1, 18.7; LRMS *m/z* (ES+) 379 ($[M+H]^+$, 100%); HRMS *m/z* (ES+) [Found: $(M+Na)^+$ 401.1934. $C_{22}H_{26}N_4NaO_2^+$ requires 401.1948]; HPLC (C-18) retention time = 9.48 min, 96.8%; (AD-H) retention time = 13.31 min, 98.6%.

(3*S*)-*N*-[3-(3,4-Dihydroquinolin-1(2*H*)-yl)propyl]-3-methyl-2-oxo-1,2,3,4-tetrahydroquinoxaline-5-carboxamide [(*S*)-1]

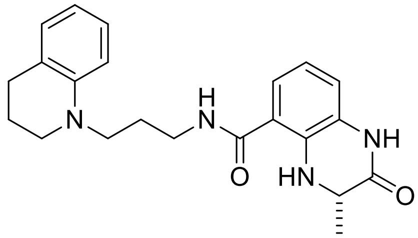

Compounds (*S*)-**15** (35 mg, 0.17 mmol, 1.0 eq) and **75** (36 mg, 0.19 mmol, 1.1 eq) were reacted according to general procedure 1 for 19 h. Purification *via* silica gel chromatography (gradient elution 8 to 100% EtOAc in petroleum ether) gave (*S*)-**1** (31 mg, 0.082 mmol, 48%) as a brown oil. R_f 0.37 (1:1 EtOAc:petroleum ether); $[\alpha]_D$ +20.8 (*c* 1.0 in $CHCl_3$); ^{1}H NMR (400 MHz; $(CD_3)_2CO$): δ 9.33 (br s, 1H), 8.03 (br s, 1H), 7.80 (br s, 1H), 7.30 (dd, *J* 8.0 1.3 Hz, 1H), 6.99-6.91 (m, 2H), 6.89-6.84 (m, 1H), 6.65 (dd, *J* 8.0 7.9 Hz, 1H), 6.64-6.59 (m, 1H), 6.46 (ddd, *J* 7.3 7.3 1.0 Hz, 1H), 4.02 (qd, *J* 6.6 1.5 Hz, 1H), 3.51-3.43 (m, 2H), 3.39 (t, *J* 7.4 Hz, 2H), 3.32 (t, *J* 5.6 Hz, 2H), 2.71 (t, *J* 6.4 Hz, 2H), 1.96-1.87 (m, 4H), 1.39 (d, *J* 6.6 Hz, 3H); LRMS *m/z* (ES+) 379 ($[M+H]^+$, 100%); HPLC (C-18) retention time = 9.42 min, 95.6%; (AD-H) retention time = 14.82 min, 97.1%. These data are in agreement with the enantiomer (*R*)-**1**.

(3*R*)-*N*-[3-(6-Methoxy-3,4-dihydroquinolin-1(2*H*)-yl)propyl]-3-methyl-2-oxo-1,2,3,4-tetrahydroquinoxaline-5-carboxamide [(*R*)-6]

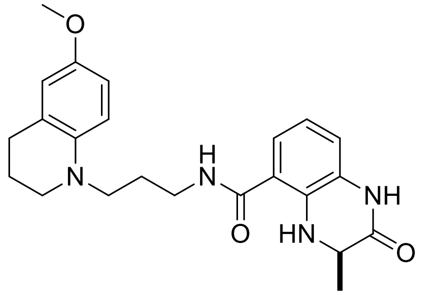

Compounds (*R*)-**15** (40 mg, 0.19 mmol, 1.0 eq) and **76** (47 mg, 0.21 mmol, 1.1 eq) were reacted according to general procedure 1 for 19 h. Purification *via* silica gel chromatography (gradient elution 12 to 100% EtOAc in petroleum ether) gave (*R*)-**6** (37 mg, 0.091 mmol, 48%) as a brown oil. R_f 0.32 (2:1 EtOAc:petroleum ether); $[\alpha]_D$ −41.0 (*c* 0.1 in MeOH); ν_{max}/cm^{-1} 3305 (br), 2927 (s), 2829 (w), 1681 (s), 1505 (m), 1288 (s); ^{1}H NMR (400 MHz; $(CD_3)_2CO$): δ 9.33 (br s, 1H), 8.03 (br s, 1H), 7.80 (br s, 1H), 7.29 (dd, *J* 8.0 1.0 Hz, 1H), 6.96 (dd, *J* 7.7 1.0 Hz, 1H), 6.65 (dd, *J* 8.0 7.7 Hz, 1H), 6.60-6.57 (m, 2H), 6.55-6.53 (m, 1H), 4.01 (qd, *J* 6.7 1.5 Hz, 1H), 3.66 (s, 3H), 3.51-3.42 (m, 2H), 3.33 (t, *J* 7.3 Hz, 2H), 3.23 (t, *J* 5.6 Hz, 2H), 2.71 (t, *J* 6.4 Hz, 2H), 1.95-1.85 (m, 4H), 1.39 (d, *J* 6.7 Hz, 3H); ^{13}C NMR (126 MHz; $(CD_3)_2CO$): δ 169.8, 169.1, 152.2, 141.2, 137.8, 129.2, 125.1, 122.6, 118.3, 117.9, 117.3, 116.3, 113.5, 113.4, 56.1, 52.0, 50.8, 50.5, 38.8, 29.4, 27.5, 23.7, 19.1; LRMS *m/z* (ES+) 409 ($[M+H]^+$, 100%); HRMS *m/z* (ES+) [Found: $(M+Na)^+$ 431.2054. $C_{23}H_{28}N_4NaO_3^+$ requires 431.2054]; HPLC (C-18) retention time = 9.12 min, 98.5%; (AD-H) retention time = 16.57 min, 95.1%.

(3*R*)-*N*-[3-(7-Methoxy-3,4-dihydroquinolin-1(2*H*)-yl)propyl]-3-methyl-2-oxo-1,2,3,4-tetrahydroquinoxaline-5-carboxamide [(*R*)-2]

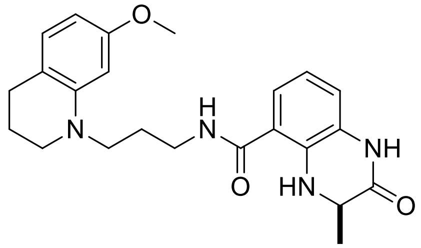

Compounds (*R*)-**15** (83 mg, 0.40 mmol, 1.0 eq) and **74** (98 mg, 0.44 mmol, 1.1 eq) were reacted according to general procedure 1 for 24 h. Purification *via* silica gel chromatography (gradient elution 40 to 100% Et_2O in petroleum ether) gave (*R*)-**2** (107 mg, 262 mmol, 65%) as a colorless solid. R_f 0.37 (2:1 EtOAc:petroleum ether); mp 101-103 °C (from $(CH_3)_2CO$); $[\alpha]_D$ −23.2 (*c* 0.5 in $(CH_3)_2CO$); ν_{max}/cm^{-1} 3328 (br), 2933 (m), 1682 (s), 1609 (m), 1509 (s), 1288 (s); ^1H NMR (500 MHz; $(CD_3)_2CO$): 9.40 (br s, 1H), 8.04 (br s, 1H), 7.83 (br s, 1H), 7.30 (dd, *J* 7.9 1.1 Hz, 1H), 6.95 (d, *J* 7.6 Hz, 1H), 6.75 (d, *J* 8.1 Hz, 1H), 6.64 (dd, *J* 7.9 7.6 Hz, 1H), 6.16 (d, *J* 2.4 Hz, 1H), 6.07 (dd, *J* 8.1 2.4 Hz, 1H), 4.01 (qd, *J* 6.7 1.4 Hz, 1H), 3.65 (s, 3H), 3.50-3.44 (m, 2H), 3.36 (t, *J* 7.4 Hz, 2H), 3.27 (t, *J* 5.6 Hz, 2H), 2.62 (t, *J* 6.3 Hz, 2H), 1.96-1.82 (m, 4H), 1.38 (d, *J* 6.7 Hz, 3H); ^{13}C NMR (126 MHz; $(CD_3)_2CO$): δ 169.5, 168.8, 160.3, 146.9, 137.4, 130.2, 128.8, 122.3, 117.9, 117.5, 116.9, 115.7, 101.1, 97.9, 55.1, 51.7, 50.0, 49.8, 38.2, 28.1, 27.1, 23.3, 18.7; LRMS *m/z* (ES+) 431 ($[M+Na]^+$, 100%); HRMS *m/z* (ES+) [Found: $(M+H)^+$ 409.2236. $C_{23}H_{29}N_4O_3^+$ requires 409.2234]; HPLC (C-18) retention time = 10.19 min, 98.7%; (AD-H) retention time = 14.96 min, 98.2%.

(3*R*)-*N*-[3-(2,3-Dihydro-4*H*-1,4-benzoxazin-4-yl)propyl]-3-methyl-2-oxo-1,2,3,4-tetrahydroquinoxaline-5-carboxamide [(*R*)-5]

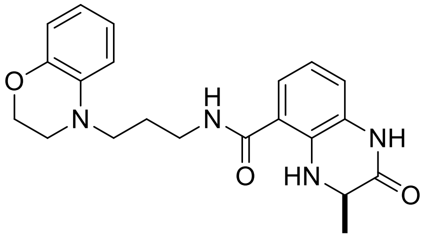

Compounds (*R*)-**15** (60 mg, 0.29 mmol, 1.0 eq) and **77** (62 mg, 0.32 mmol, 1.1 eq) were reacted according to general procedure 1 for 16 h. Purification *via* silica gel chromatography (gradient elution 12 to 100% EtOAc in petroleum ether) gave (*R*)-**5** (84 mg, 0.22 mmol, 76%) as a colorless oil. R_f 0.28 (2:1 EtOAc:petroleum ether); $[\alpha]_D$ −41.6 (*c* 0.5 in $(CH_3)_2CO$); ν_{max}/cm^{-1} 3320 (br), 2932 (m), 2875 (m), 1695 (s), 1502 (m), 1286 (s); ^1H NMR (400 MHz; $(CD_3)_2CO$): δ 9.32 (br s, 1H), 8.02 (br s, 1H), 7.82 (br s, 1H), 7.30 (dd, *J* 7.8 1.2 Hz, 1H), 6.96 (d, *J* 7.8 Hz, 1H), 6.76-6.71 (m, 2H), 6.69-6.61 (m, 2H), 6.55-6.47 (m, 1H), 4.22-4.18 (m, 2H), 4.01 (qd, *J* 6.7 1.5 Hz, 1H), 3.51-3.44 (m, 2H), 3.42-3.35 (m, 4H), 1.93 (tt, *J* 7.4 7.4 Hz, 2H), 1.37 (d, *J* 6.7 Hz, 3H); ^{13}C

NMR (126 MHz; $(CD_3)_2CO$): δ 168.6, 167.8, 144.3, 136.5, 135.4 , 127.9, 121.4, 121.3, 116.99, 116.97, 116.92, 116.6, 116.0, 112.2, 64.3, 50.8, 48.3, 46.8, 37.2, 26.0, 17.8; LRMS *m/z* (ES+) 381 ($[M+H]^+$, 100%); HRMS *m/z* (ES+) [Found: $(M+Na)^+$ 403.1734. $C_{21}H_{24}N_4NaO_3^+$ requires 403.1741]; HPLC (C-18) retention time = 10.74 min, 98.3%; (AD-H) retention time = 15.00 min, 99.5%.

(3*R*)-*N*-[3-(7-methoxy-3,4-dihydroquinolin-1(2*H*)-yl)propyl]-6-fluoro-3-methyl-2-oxo-1,2,3,4-tetrahydroquinoxaline-5-carboxamide [(*R*)-8]

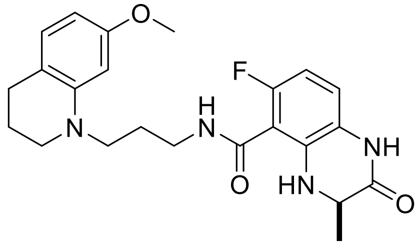

Compounds (*R*)-**20** (39 mg, 0.17 mmol, 1.0 eq) and **74** (42 mg, 0.19 mmol, 1.1 eq) were reacted according to general procedure 1 for 21 h. Purification *via* silica gel chromatography (3:1 then 1:0 Et_2O:petroleum ether) yielded (*R*)-**8** (20 mg, 0.047 mmol, 27%) as a pale yellow oil. R_f 0.70 (2:1 EtOAc:petroleum ether); $[\alpha]_D$ −6.1 (*c* 0.5 in $(CH_3)_2CO$); ν_{max}/cm^{-1} 3270 (br), 2937 (s), 1685 (s), 1508 (s), 1307 (s); ^1H NMR (500 MHz; $(CD_3)_2CO$): δ 9.34 (br s, 1H), 7.84 (br s, 1H), 7.64 (br s, 1H), 6.92 (dd, *J* 8.6 5.3 Hz, 1H), 6.77 (d, *J* 8.1 Hz, 1H), 6.46 (dd, *J* 11.8 8.6 Hz, 1H), 6.19 (d, *J* 2.4 Hz, 1H), 6.09 (dd, *J* 8.1 2.4 Hz, 1H), 4.03 (qd, *J* 6.7 1.5 Hz, 1H), 3.69 (s, 3H), 3.53 (dt, *J* 6.7 6.2 Hz, 2H), 3.39 (t, *J* 7.3 Hz, 2H), 3.30 (t, *J* 5.6 Hz, 2H), 2.65 (t, *J* 6.3 Hz, 2H), 1.95 (tt, *J* 7.3 6.7 Hz, 2H), 1.93-1.87 (m, 2H), 1.39 (d, *J* 6.7 Hz, 3H); ^{13}C NMR (126 MHz; $(CD_3)_2CO$): δ 168.0, 165.8 (d, *J* 2.0 Hz), 160.3, 157.9 (d, *J* 239.4 Hz), 146.9, 138.1 (d, *J* 5.7 Hz), 130.2, 124.8 (d, *J* 1.8 Hz), 117.4 (d, *J* 11.6 Hz), 115.7, 106.4 (d, *J* 17.9 Hz), 103.9 (d, *J* 27.2 Hz), 101.2, 98.0, 55.1, 51.4, 50.0, 49.7, 38.3, 28.1, 27.0, 23.3, 18.7; ^{19}F NMR (470 MHz; $(CD_3)_2CO$): δ −120.3 - −120.4 (m, 1F); LRMS *m/z* (ES+) 427 ($[M+H]^+$, 100%); HRMS *m/z* (ES+) [Found: $(M+H)^+$ 427.2137. $C_{23}H_{28}FN_4O_3^+$ requires 427.2140]; HPLC (C-18) retention time = 10.72 min, 97.5%; (AD-H) retention time = 13.31 min, 98.4%.

(3*R*)-*N*-[3-(7-methoxy-3,4-dihydroquinolin-1(2*H*)-yl)propyl]-7-fluoro-3-methyl-2-oxo-1,2,3,4-tetrahydroquinoxaline-5-carboxamide [(*R*)-7]

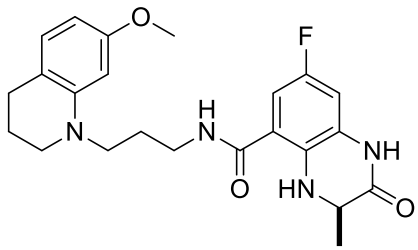

Compounds (*R*)-**27** (38 mg, 0.17 mmol, 1.0 eq) and **74** (42 mg, 0.19 mmol, 1.1 eq) were reacted according to general procedure 1 for 21 h. Purification *via* silica gel chromatography (2:1 then 1:0 Et_2O:petroleum ether) yielded (*R*)-**7** (21 mg, 0.049 mmol, 29%) as a pale yellow oil. R_f 0.45 (Et_2O); $[\alpha]_D$ −21.2 (*c* 0.5 in $(CH_3)_2CO$); ν_{max}/cm^{-1} 3322 (br), 2935 (s), 1686 (s), 1611 (s), 1509 (s), 1308 (s); ^1H NMR (500 MHz; $(CD_3)_2CO$): δ 9.46 (br s, 1H), 7.85 (br s, 2H), 7.11 (dd, *J* 9.8 2.7 Hz, 1H), 6.81 (dd, *J* 9.3 2.7 Hz, 1H), 6.75 (d, *J* 8.1 Hz, 1H), 6.16 (d, *J* 2.3 Hz, 1H), 6.07 (dd, *J* 8.1 2.3, 1H), 3.98 (qd, *J* 6.7 1.5 Hz, 1H), 3.66 (s, 3H), 3.47 (q, *J* 6.6 Hz, 2H), 3.37 (t, *J* 7.4 Hz, 2H), 3.28 (t, *J* 5.6 Hz, 2H), 2.63 (t, *J* 6.3 Hz, 2H), 1.96-1.84 (m, 4H), 1.37 (d, *J* 6.7, 3H); ^{13}C NMR (126 MHz; $(CD_3)_2CO$): δ 169.0, 168.5 (d, *J* 2.7 Hz), 160.3, 155.3 (d, *J* 232.8 Hz), 146.9, 134.1, 130.2, 130.1, 116.8 (d, *J* 6.8 Hz), 115.7, 107.3 (d, *J* 23.5 Hz), 105.5 (d, *J* 27.4 Hz), 101.1, 98.0, 55.1, 51.6, 50.0, 49.7, 38.3, 28.1, 27.0, 23.3, 18.4; ^{19}F NMR (470 MHz; $(CD_3)_2CO$): δ −125.9 (dd, J 9.8 9.3 Hz, 1F); LRMS *m/z* (ES+) 427 ($[M+H]^+$, 100%); HRMS *m/z* (ES+) [Found: $(M+H)^+$ 427.2137. $C_{23}H_{28}FN_4O_3^+$ requires 427.2140]; HPLC (C-18) retention time = 10.51 min, 98.1%; (AD-H) retention time = 15.76 min, 96.1%.

2-Methyl-2*H*-1,4-benzoxazin-3(4H)-one (32)

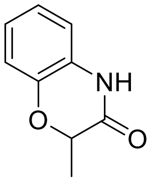

2-Aminophenol (108 mg, 0.986 mmol) was reacted according to general procedure 2 method 2. Purification *via* silica gel chromatography (gradient elution 5 to 40% EtOAc in petroleum ether) gave **32** (54 mg, 0.325 mmol, 33%) as a colorless solid. R_f 0.33 (1:2 EtOAc:petroleum ether); mp 141-142 °C (from EtOAc, lit.[47] 143-144 °C); ^1H NMR (400 MHz; $CDCl_3$): δ 7.78 (s, 1H), 7.01-6.93 (m, 3H), 6.81-6.77 (m, 1H), 4.67 (q, *J* 6.8 Hz, 1H), 1.60 (d, *J* 6.8 Hz, 3H); LRMS *m/z* (ES+) 186 ($[M+Na]^+$, 100%). These data are in accordance with the literature.[47]

7-Bromo-2-methyl-2*H*-1,4-benzoxazin-3(4H)-one (33)

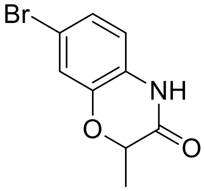

2-Amino-5-bromophenol (350 mg, 1.86 mmol) was reacted according to general procedure 2 method 1. Purification *via* silica gel chromatography (gradient elution 6 to 48% EtOAc in petroleum ether) gave **33** (230 mg, 0.954 mmol, 51%) as a brown solid. R_f 0.25 (1:2 EtOAc: petroleum ether); mp 127-128 °C (from $(CH_3)_2CO$); ν_{max}/cm^{-1} 3048 (w), 1682 (s), 1499 (m), 1396 (m); ^1H NMR (500 MHz; CD_3OD): δ 7.14 (d, *J*

2.1 Hz, 1H), 7.12 (dd, *J* 8.3 2.1 Hz, 1H), 6.82 (d, *J* 8.3 Hz, 1H), 4.67 (q, *J* 6.8 Hz, 1H), 1.52 (d, *J* 6.8 Hz, 3H); ^{13}C NMR (126 MHz; CD_3OD): δ 169.3, 145.5, 128.0, 126.5, 121.0, 118.0, 116.3, 74.7, 16.6; LRMS *m/z* (ES+) 264 ([M(^{79}Br)+Na]$^+$, 100%), 266 ([M(^{81}Br)+Na]$^+$, 100%); HRMS *m/z* (ES+) [Found: (^{79}M+Na)$^+$ 263.9632. $C_9H_8{}^{79}BrNNaO_2^+$ requires 263.9631], [Found: (^{81}M+Na)$^+$ 265.9604. $C_9H_8{}^{81}BrNNaO_2^+$ requires 265.9610]; Anal. calcd for $C_9H_8BrNO_2$: C 44.66%, H 3.33%, N 5.79%; Found: C 44.79%, H 3.30%, N 5.79%.

8-Bromo-2-methyl-2*H*-1,4-benzoxazin-3(4H)-one (34)

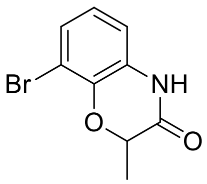

2-Amino-6-bromophenol (840 mg, 4.47 mmol) was reacted according to general procedure 2 method 1. Purification *via* silica gel chromatography (gradient elution 5 to 40% EtOAc in petroleum ether) gave a brown solid. The solid was redissolved in EtOAc, stirred with charcoal for 2 h, filtered through Celite® (eluent EtOAc) and concentrated *in vacuo* to give **34** (830 mg, 3.44 mmol, 77%) as a colorless solid. R_f 0.26 (1:2 EtOAc:petroleum ether); mp 134-136 °C (from $(CH_3)_2CO$); ν_{max}/cm^{-1} 3055 (w), 1682 (s), 1597(m), 1478 (m); ^{1}H NMR (400 MHz; $CDCl_3$): δ 9.97 (br s, 1H), 7.21 (dd, *J* 7.8 1.7 Hz, 1H), 6.85 (dd, *J* 7.9 7.8 Hz, 1H), 6.81 (dd, *J* 7.9 1.7 Hz, 1H), 4.79 (q, *J* 6.9 Hz, 1H), 1.63 (d, *J* 6.9 Hz, 3H); ^{13}C NMR (126 MHz; $CDCl_3$): δ 168.9, 140.4, 127.8, 127.4, 123.3, 115.0, 111.0, 73.9, 16.4; LRMS *m/z* (ES+) 264 ([M(^{79}Br)+Na]$^+$, 98%), 266 ([M(^{81}Br)+Na]$^+$, 100%); HRMS *m/z* (ES+) [Found: (^{79}M+Na)$^+$ 263.9625. $C_9H_8{}^{79}BrNNaO_2^+$ requires 263.9631], [Found: (^{81}M+Na)$^+$ 265.9604. $C_9H_8{}^{81}BrNNaO_2^+$ requires 265.9610]; Anal. calcd for $C_9H_8BrNO_2$: C 44.66%, H 3.33%, N 5.79%; Found: C 44.63%, H 3.21%, N 5.71%.

Methyl 2-methyl-3-oxo-3,4-dihydro-2*H*-1,4benzoxazine-8-carboxylate (35)

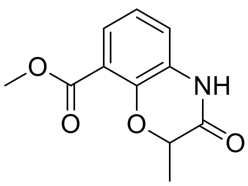

Methyl 3-amino-2-hydroxybenzoate (2.01 g, 12.0 mmol) was reacted according to general procedure 2 method 2. Purification *via* silica gel chromatography (1:4 EtOAc:petroleum ether) gave **35** (1.77 g, 8.00 mmol, 67%) as a colorless solid. R_f 0.13 (1:2 EtOAc:petroleum ether); mp 144-146 °C (from $(CH_3)_2CO$); ν_{max}/cm^{-1} 3076 (w), 1730 (s), 1703 (s), 1477 (m), 1286 (m); ^{1}H NMR (500 MHz; $CDCl_3$): δ 9.44 (br s, 1H), 7.53 (dd, *J* 7.4 2.4 Hz, 1H), 7.03 (dd, *J* 7.7 2.4 Hz, 1H), 7.00 (dd, *J* 7.7 7.4 Hz, 1H), 4.79 (q, *J* 6.9 Hz, 1H), 3.92 (s, 3H), 1.62 (d, *J* 6.9 Hz, 3H); ^{13}C NMR (126 MHz; $CDCl_3$): δ 168.5, 165.6, 143.3, 127.6, 126.1, 122.0, 120.5, 119.5, 73.6, 52.2, 16.3; LRMS *m/z* (ES+) 465 ([2M+Na]$^+$, 100%); HRMS *m/z* (ES+) [Found: (M+H)$^+$ 244.0578.

$C_{11}H_{11}NNaO_4^+$ requires 244.0580]; Anal. calcd for $C_{11}H_{11}NO_4$: C 59.73%, H 5.01%, N 6.33%; Found: C 59.63%, H 4.94%, N 6.24%.

2-Methyl-3-oxo-3,4-dihydro-2*H*-1,4benzoxazine-8-carboxylic acid (36)

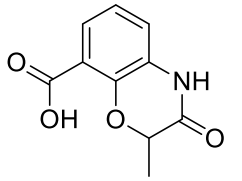

To a solution of **35** (1.94 g, 8.77 mmol, 1.0 eq) in MeOH (70 mL) and THF (70 mL) was added a solution of LiOH aq. (1 M, 44 mL, 5.0 eq). The reaction mixture was stirred at rt for 32 h, then concentrated *in vacuo*, diluted with H_2O (50 mL) and acidified with HCl aq. (1 M). The resulting precipitate was filtered and washed with 1 M HCl (10 mL) and EtOAc (20 mL) to give **36** (1.65 g, 91%) as a colorless solid which was used without further purification. R_f 0.53 (1:99 AcOH:EtOAc); mp 211 °C (dec.); ν_{max}/cm^{-1} 3001 (w), 1739 (m), 1671 (s), 1481 (m), 1220 (m); ^{1}H NMR (500 MHz; $(CD_3)_2SO$): δ 12.81 (br s, 1H), 10.80 (s, 1H), 7.31 (dd, *J* 7.7 1.8 Hz, 1H), 7.05 (dd, *J* 7.7 1.8 Hz, 1H), 7.01 (dd, *J* 7.7 7.7 Hz, 1H), 4.73 (q, *J* 6.8 Hz, 1H), 1.41 (d, *J* 6.8 Hz, 3H); ^{13}C NMR (126 MHz; $CDCl_3$): δ 166.9, 166.4, 142.4, 128.7, 124.3, 121.8, 121.2, 118.7, 72.7, 16.0; LRMS *m/z* (ES−) 206 ([M−H]$^-$, 100%); HRMS *m/z* (ES−) [Found: (M−H)$^-$ 206.0458. $C_{10}H_8NO_4^-$ requires 206.0459]; Anal. calcd for $C_{10}H_9NO_4$: C 57.97%, H 4.38%, N 6.76%; Found: C 57.88%, H 4.47%, N 6.68%.

***N*-[2-(2,3-Dihydro-1*H*-indol-1-yl)ethyl]-2-methyl-3-oxo-3,4-dihydro-2*H*-1,4-benzoxazine-8-carboxamide (3)**

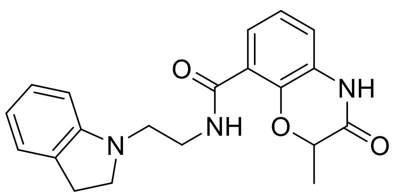

Compound **36** (75 mg, 0.36 mmol, 1.0 eq) and 2-(2,3-dihydro-1*H*-indol-1-yl)ethanamine (65 mg, 0.40 mmol, 1.1 eq) were reacted according to general procedure 1 for 44 h. Purification *via* silica gel chromatography (gradient elution 12 to 100% EtOAc in petroleum ether) gave **3** (112 mg, 0.319 mmol, 88%) as a colorless solid. R_f 0.44 (2:1 EtOAc:petroleum ether); mp 142-144 °C (from MeOH); ν_{max}/cm^{-1} 3391 (w), 3231 (w), 2821 (w), 1693 (s), 1650 (s), 1534 (m), 1305 (m); ^{1}H NMR (500 MHz; $CDCl_3$): δ 9.05 (s, 1H), 8.12 (s, 1H), 7.90 (dd, *J* 8.0 1.6 Hz, 1H), 7.14-7.02 (m, 3H), 6.96 (dd, *J* 7.7 1.6 Hz, 1H), 6.69 (dd, *J* 7.6 7.6 Hz, 1H), 6.54 (d, *J* 7.9 Hz, 1H), 4.49 (q, *J* 6.9 Hz, 1H), 3.83-3.68 (m, 2H), 3.51-3.26 (m, 4H), 3.01 (t, *J* 8.3 Hz, 2H), 1.34 (t, *J* 6.9 Hz, 3H); ^{13}C NMR (126 MHz; $CDCl_3$): δ 167.3, 164.2, 152.5, 141.5, 129.6, 127.4, 127.0, 126.4, 124.7,

122.8, 122.1, 118.9, 118.3, 107.0, 74.1, 53.3, 49.0, 38.0, 28.7, 15.7; LRMS *m/z* (ES+) 352 ($[M+H]^+$, 100%); HRMS *m/z* (ES+) [Found: $(M+Na)^+$ 374.1476. $C_{20}H_{21}N_3NaO_3^+$ requires 374.1475]; Anal. calcd for $C_{20}H_{21}N_3O_3$: C 68.36%, H 6.02%, N 11.96%; Found: C 68.36%, H 5.93%, N 11.86%.

***N*-[3-(2,3-Dihydro-1*H*-indol-1-yl)propyl]-2-methyl-3-oxo-3,4-dihydro-2*H*-1,4-benzoxazine-8-carboxamide (37)**

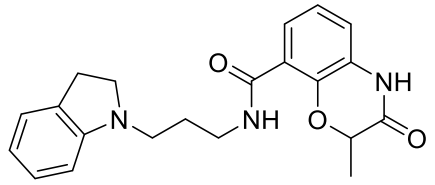

Compound **36** (75 mg, 0.36 mmol, 1.0 eq) and 3-(2,3-dihydro-1*H*-indol-1-yl)propan-1-amine (70 mg, 0.40 mmol, 1.1 eq) were reacted according to general procedure 1 for 44 h. Purification *via* silica gel chromatography (gradient elution 12 to 100% EtOAc in petroleum ether) gave **37** (99 mg, 0.27 mmol, 75%) as a colorless oil. Crystallisation from MeOH afforded a pale grey solid. R_f 0.38 (2:1 EtOAc:petroleum ether); mp 128-130 °C (MeOH); ν_{max}/cm^{-1} 3292 (w), 3062 (w), 2925 (w), , 1680 (s), 1633 (s), 1470 (m), 1314 (m); ^1H NMR (500 MHz; $CDCl_3$): δ 9.07 (s, 1H), 7.89 (dd, *J* 8.0 1.6 Hz, 1H), 7.87-7.81 (m, 1H), 7.13-7.03 (m, 3H), 7.00 (dd, *J* 7.7 1.6 Hz, 1H), 6.68 (dd, *J* 7.3 7.3 Hz, 1H), 6.49 (d, *J* 7.8 Hz, 1H), 4.63 (q, *J* 6.9 Hz, 1H), 3.71-3.57 (m, 2H), 3.41-3.31 (m, 2H), 3.18 (t, *J* 6.9 Hz, 2H), 2.95 (t, *J* 8.3 Hz, 2H), 2.02-1.92 (m, 2H), 1.53 (d, *J* 6.9 Hz, 3H); ^{13}C NMR (126 MHz; $CDCl_3$): δ 167.3, 164.2, 152.5, 141.3, 130.0, 127.3, 126.9, 126.5, 124.5, 122.9, 122.4, 118.9, 117.9, 106.9, 74.1, 53.4, 47.5, 38.2, 28.6, 27.3, 16.2; LRMS *m/z* (ES+) 366 ($[M+H]^+$, 100%); HRMS *m/z* (ES+) [Found: $(M+Na)^+$ 388.1630. $C_{21}H_{23}N_3NaO_3^+$ requires 388.1632]; Anal. calcd for $C_{21}H_{23}N_3O_3$: C 69.02%, H 6.34%, N 11.50%; Found: C 69.02%, H 6.41%, N 11.58%.

***N*-[2-(3,4-Dihydroquinolin-1(2*H*)-yl)propyl]-2-methyl-3-oxo-3,4-dihydro-2*H*-1,4-benzoxazine-8-carboxamide (4)**

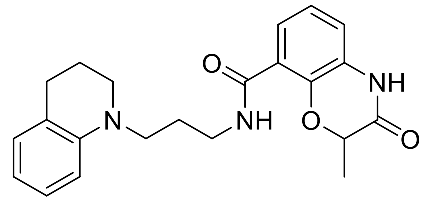

Compound **36** (50 mg, 0.24 mmol, 1.0 eq) and **75** (51 mg, 0.27 mmol, 1.1 eq) were reacted according to general procedure 1 for 44 h. Purification *via* silica gel chromatography (gradient elution 12 to 100% EtOAc in petroleum ether) gave **4** (79 mg, 0.21 mmol, 86%) as a brown oil. R_f 0.32 (2:1 EtOAc:petroleum ether); ν_{max}/cm^{-1} 3400 (w), 2928 (m), 1700 (s), 1643 (m), 1591 (m), 1301 (m); ^1H NMR (400 MHz; $CDCl_3$): δ 8.47 (br s, 1H), 7.88 (dd, *J* 7.9 1.6 Hz, 1H), 7.68 (br t, *J* 5.8 Hz, 1H), 7.09 (dd, *J* 7.9 7.9 Hz, 1H), 7.06-7.00 (m,

1H), 6.98-6.93 (m, 2H), 6.60-6.55 (m, 2H), 4.71 (q, *J* 6.8 Hz, 1H), 3.57 (td, *J* 6.5 5.8 Hz, 2H), 3.37 (td, *J* 7.1 2.5 Hz, 2H), 3.28 (t, *J* 5.6 Hz, 2H), 2.74 (t, *J* 6.3 Hz, 2H), 2.00-1.90 (m, 4H), 1.59 (d, *J* 6.8 Hz, 3H); ^{13}C NMR (126 MHz; $CDCl_3$) δ 167.0, 164.3, 145.3, 141.3, 129.5, 127.2, 127.0, 126.7, 123.2, 122.7, 122.6, 118.9, 116.0, 110.5, 74.3, 49.9, 49.5, 38.2, 28.2, 26.8, 22.3, 16.5; LRMS *m/z* (ES+) 781 ($[2M+Na]^+$, 100%); HRMS *m/z* (ES+) [Found: $(M+H)^+$ 380.1963. $C_{22}H_{26}N_3O_3^+$ requires 380.1969].

N-benzyl-2-methyl-3-oxo-3,4-dihydro-2H-1,4-benzoxazine-8-carboxamide (9)

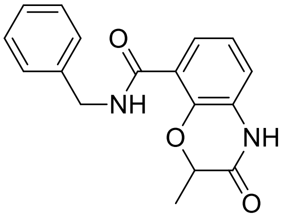

Compound **36** (50 mg, 0.24 mmol, 1.0 eq) and benzylamine (28 mg, 29 µL, 0.27 mmol, 1.1 eq) were reacted according to general procedure 1 for 18 h to give **9** (68 mg, 0.23 mmol, 95%) as a colorless solid. R_f 0.32 (2:1 EtOAc:petroleum ether); mp 137-139 °C (EtOAc); ν_{max}/cm^{-1} 3323 (m), 2926 (m), 2360 (w), 1685 (s), 1627 (s), 1589 (m), 1311 (m); ^{1}H NMR (400 MHz; $(CD_3)_2SO$): δ 10.81 (s, 1H), 8.61 (dd, *J* 5.9 5.8 Hz, 1H), 7.42-7.20 (m, 6H), 7.08-6.99 (m, 2H), 4.80 (q, *J* 6.8 Hz, 1H), 4.54 (dd, *J* 15.3 5.9 Hz, 1H), 4.47 (dd, *J* 15.3 5.8 Hz, 1H), 1.44 (d, *J* 6.8 Hz, 3H); ^{13}C NMR (101 MHz; $(CD_3)_2SO$) δ 166.7, 164.3, 140.8, 139.4, 128.3, 128.2, 126.9, 126.7, 123.9, 123.4, 122.2, 117.6, 73.1, 42.5, 15.8; LRMS *m/z* (ES−) 295 ($[M-H]^-$, 100%); HRMS *m/z* (ES+) [Found: $(M+H)^+$ 319.1052. $C_{17}H_{16}N_2NaO_3^+$ requires 319.1053]; Anal. calcd for $C_{17}H_{16}N_2O_3$: C 68.91%, H 5.44%, N 9.45%; Found: C 69.03%, H 5.38%, N 9.38%.

N-[3-(1,3-benzothiazol-2-yl)propyl]-2-methyl-3-oxo-3,4-dihydro-2H-1,4-benzoxazine-8-carboxamide (38)

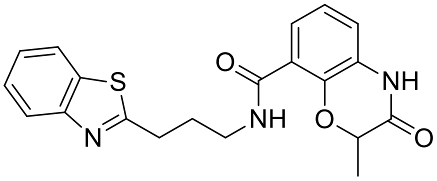

3-(1,3-benzothiazol-2-yl)propan-1-amine and **36** were reacted according to general procedure 3 to afford **38**. LC-MS retention time = 2.89 min (91%), *m/z* (ES+) 382 ($[M+H]^+$, 100%).

N-[2-(6-methoxy-1H-indol-3-yl)ethyl]-2-methyl-3-oxo-3,4-dihydro-2H-1,4-benzoxazine-8-carboxamide (39)

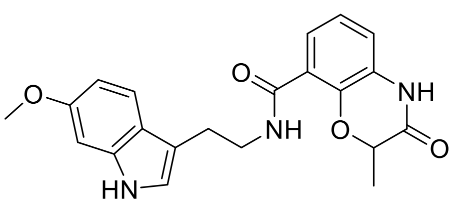

2-(6-methoxy-1*H*-indol-3-yl)ethan-1-amine and **36** were reacted according to general procedure 3 to afford **39**. LC-MS retention time = 2.87 min (92%), *m/z* (ES+) 380 ([M+H]$^+$, 100%).

N-[2-(2,4-dimethoxyphenyl)ethyl]-2-methyl-3-oxo-3,4-dihydro-2H-1,4-benzoxazine-8-carboxamide (40)

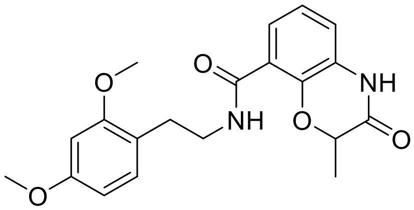

2-(2,4-dimethoxyphenyl)ethan-1-amine and **36** were reacted according to general procedure 3 to afford **40**. LC-MS retention time = 3.08 min (100%), *m/z* (ES+) 371 ([M+H]$^+$, 100%).

2-methyl-N-[2-(1-methyl-1H-pyrrolo[2,3-b]pyridin-3-yl)ethyl]-3-oxo-3,4-dihydro-2H-1,4-benzoxazine-8-carboxamide (41)

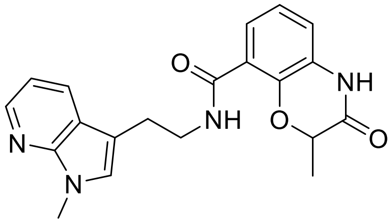

2-(1-methyl-1*H*-pyrrolo[2,3-*b*]pyridin-3-yl)ethan-1-amine and **36** were reacted according to general procedure 3 to afford **41**. LC-MS retention time = 2.61 min (90%), *m/z* (ES+) 365 ([M+H]$^+$, 100%).

2-methyl-N-[2-(5-methyl-1H-indol-3-yl)ethyl]-3-oxo-3,4-dihydro-2H-1,4-benzoxazine-8-carboxamide (42)

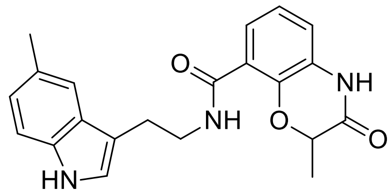

2-(5-methyl-1*H*-indol-3-yl)ethan-1-amine and **36** were reacted according to general procedure 3 to afford **42**. LC-MS retention time = 3.03 min (100%), *m/z* (ES+) 364 ([M+H]$^+$, 100%).

2-methyl-3-oxo-*N*-{2-[4-(trifluoromethyl)phenyl]ethyl}-3,4-dihydro-2*H*-1,4-benzoxazine-8-carboxamide (43)

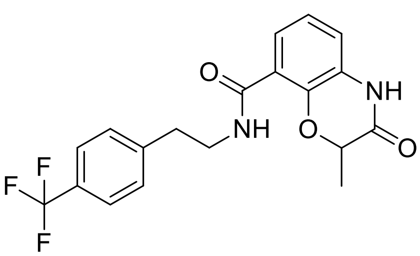

2-[4-(trifluoromethyl)phenyl]ethan-1-amine and **36** were reacted according to general procedure 3 to afford **43**. LC-MS retention time = 3.20 min (90%), *m/z* (ES+) 379 ($[M+H]^+$, 100%).

***N*-{2-[(2-hydroxybenzoyl)amino]ethyl}-2-methyl-3-oxo-3,4-dihydro-2*H*-1,4-benzoxazine-8-carboxamide (44)**

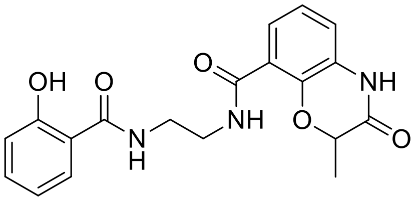

2-[(2-hydroxybenzoyl)amino]ethan-1-amine and **36** were reacted according to general procedure 3 to afford **44**. LC-MS retention time = 2.71 min (100%), *m/z* (ES+) 370 ($[M+H]^+$, 100%).

***N*-[2-(1*H*-indol-1-yl)ethyl]-2-methyl-3-oxo-3,4-dihydro-2*H*-1,4-benzoxazine-8-carboxamide (45)**

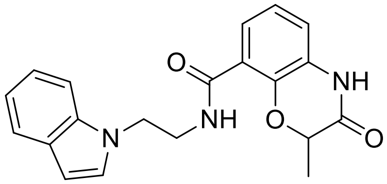

2-(1*H*-indol-1-yl)ethan-1-amine and **36** were reacted according to general procedure 3 to afford **36**. LC-MS retention time = 3.02 min (100%), *m/z* (ES+) 350 ($[M+H]^+$, 100%).

***N*-[2-(2-chlorophenyl)ethyl]-2-methyl-3-oxo-3,4-dihydro-2*H*-1,4-benzoxazine-8-carboxamide (46)**

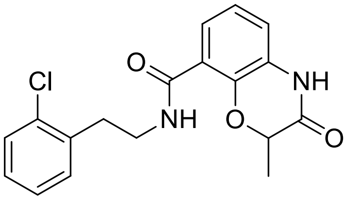

2-(2-chlorophenyl)ethan-1-amine and **36** were reacted according to general procedure 3 to afford **46**. LC-MS retention time = 3.07 min (100%), *m/z* (ES+) 345 ($[M+H]^+$, 100%).

N-[3-(4-methoxyphenyl)propyl]-2-methyl-3-oxo-3,4-dihydro-2H-1,4-benzoxazine-8-carboxamide (47)

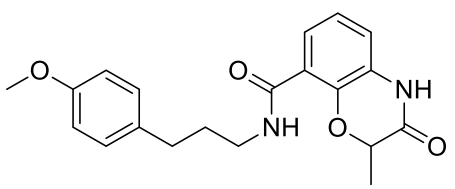

3-(4-methoxyphenyl)propan-1-amine and **36** were reacted according to general procedure 3 to afford **47**. LC-MS retention time = 3.12 min (91%), *m/z* (ES+) 355 ($[M+H]^+$, 100%).

2-methyl-N-{2-[methyl(phenyl)amino]ethyl}-3-oxo-3,4-dihydro-2H-1,4-benzoxazine-8-carboxamide (48)

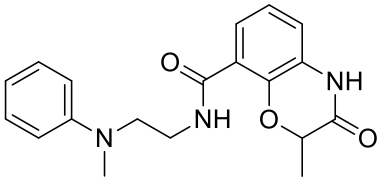

2-[methyl(phenyl)amino]ethan-1-amine and **36** were reacted according to general procedure 3 to afford **48**. LC-MS retention time = 2.99 min (100%), *m/z* (ES+) 340 ($[M+H]^+$, 100%).

2-methyl-3-oxo-N-[(3-phenyl-1H-pyrazol-4-yl)methyl]-3,4-dihydro-2H-1,4-benzoxazine-8-carboxamide (49)

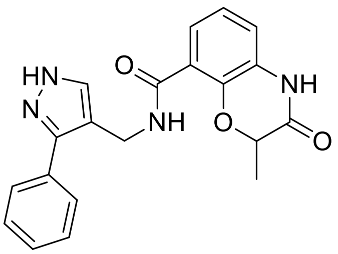

1-(3-phenyl-1H-pyrazol-4-yl)methanamine and **36** were reacted according to general procedure 3 to afford **49**. LC-MS retention time = 2.66 min (100%), *m/z* (ES+) 363 ($[M+H]^+$, 100%).

Methyl 2-{[(2S)-1-methoxy-1-oxopropan-2-yl]oxy}-3-nitrobenzoate [(S)-51]

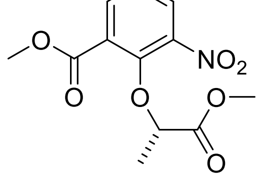

To a solution of methyl 3-nitro-2-hydroxybenzoate (1.00 g, 5.07 mmol, 1.0 eq) in CH_2Cl_2 (15 mL) were added (*R*)-methyl 2-hydroxypropanoate (792 mg, 727 µL, 7.61 mmol, 1.5 eq) and PPh_3 (2.20 g, 8.37 mmol, 1.7 eq). The mixture was stirred at rt for 10 min before being cooled to 0 °C and diisopropyl azodicarboxylate (1.29 g, 1.26 mL, 6.40 mmol, 1.3 eq) was added dropwise *via* syringe. The reaction mixture was stirred at rt for 41 h,

then concentrated *in vacuo* and purified *via* silica gel chromatography (1:5 EtOAc:petroleum ether) to give (*S*)-**51** (1.47 mg, 4.95 mmol, 98%) as a yellow oil. R_f 0.45 (1:2 EtOAc:petroleum ether); $[\alpha]_D$ +53.6 (*c* 1.0 in $CHCl_3$); ν_{max}/cm^{-1} 2955 (w), 1726 (w), 1533 (s), 1446 (m), 1360 (m), 1267 (m); ^{1}H NMR (400 MHz; $CDCl_3$): δ 8.04 (dd, *J* 7.9 1.8 Hz, 1H), 7.93 (dd, *J* 8.0 1.8 Hz, 1H), 7.27 (dd, *J* 8.0 7.9 Hz, 1H), 4.70 (q, *J* 6.8 Hz, 1H), 3.93 (s, 3H), 3.67 (s, 3H), 1.63 (d, *J* 6.8 Hz, 3H); ^{13}C NMR (101 MHz; $CDCl_3$): δ 170.9, 164.8, 150.6, 145.8, 135.7, 128.7, 126.8, 123.9, 80.4, 52.8, 52.4, 18.2; LRMS *m/z* (ES+) 589 ($[2M+Na]^+$, 100%); HRMS *m/z* (ES+) [Found: $(M+Na)^+$ 306.0573. $C_{12}H_{13}NNaO_7$ requires 306.0584]; Anal. calcd for $C_{12}H_{13}NO_7$: C 50.89%, H 4.63%, N 4.95%; Found: C 51.00%, H 4.62%, N 4.94%.

Methyl 2-{[(2*R*)-1-methoxy-1-oxopropan-2-yl]oxy}-3-nitrobenzoate [(*R*)-51]

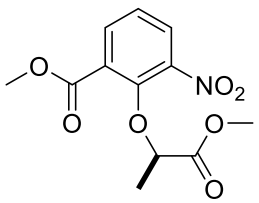

Methyl 3-nitro-2-hydroxybenzoate (1.00 g, 5.07 mmol, 1.0 eq) and (*S*)-methyl 2-hydroxypropanoate (792 mg, 727 μL, 7.61 mmol, 1.5 eq) were reacted as described above to prepare (*R*)-**51** (1.34 mg, 4.51 mmol, 89%) as a yellow oil. R_f 0.45 (1:2 EtOAc:petroleum ether); $[\alpha]_D$ −43.3 (*c* 1.0 in $CHCl_3$); ^{1}H NMR (400 MHz; $CDCl_3$): δ 8.05 (dd, *J* 7.8 1.8 Hz, 1H), 7.95 (dd, *J* 8.0 1.8 Hz, 1H), 7.29 (dd, *J* 8.0 7.8 Hz, 1H), 4.71 (q, *J* 6.8 Hz, 1H), 3.95 (s, 3H), 3.69 (s, 3H), 1.66 (d, *J* 6.8 Hz, 3H); LRMS *m/z* (ES+) 289 ($[2M+Na]^+$, 100%). These data are in accordance with the enantiomer (*S*)-**51**.

Methyl (2*S*)-2-methyl-3-oxo-3,4-dihydro-2*H*-1,4-benzoxazine-8-carboxylate [(*S*)-35]

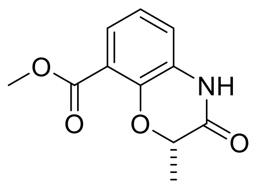

A mixture of (*S*)-**51** (191 mg, 0.643 mmol, 1.0 eq) and Pd/C (34 mg, 0.032 mmol, 0.05 eq) in EtOAc (8 mL) was placed under a H_2 atmosphere. The reaction mixture was stirred at rt for 17 h, then filtered through Celite® (elutant EtOAc), concentrated *in vacuo*. Purification *via* silica gel chromatography (gradient elution 8 to 64% EtOAc in petroleum ether) yielded to give (*S*)-**35** (115 mg, 0.520 mmol, 81%) as a colorless solid. R_f 0.13 (1:2 EtOAc:petroleum ether); mp 136-138 °C (from EtOAc); $[\alpha]_D$ +38.3 (*c* 1.0 in $CHCl_3$); ^{1}H NMR (400 MHz; $CDCl_3$): δ 9.19 (br s, 1H) 7.56-7.51 (m, 1H), 7.03-6.98 (m, 2H), 4.79 (q, *J* 6.8 Hz, 1H), 3.92 (s, 3H), 1.63 (d, *J* 6.8 Hz, 3H); LRMS *m/z* (ES+) 465 ($[2M+Na]^+$, 100%); Anal. calcd for $C_{11}H_{11}NO_4$: C 59.73%, H 5.01%, N 6.33%; Found: C 59.67%, H 4.91%, N 6.18%. These data are in accordance with the racemate **35**.

Methyl (2*R*)-2-methyl-3-oxo-3,4-dihydro-2*H*-1,4-benzoxazine-8-carboxylate [(*R*)-35]

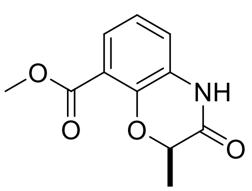

Compound (*R*)-**51** (1.34 mg, 4.51 mmol, 1.0 eq) was reacted as described above to prepare (*R*)-**35** (909 mg, 4.11 mmol, 91%) as a colorless solid. R_f 0.13 (1:2 EtOAc:petroleum ether); mp 134-135 °C (from EtOAc); $[\alpha]_D$ −32.0 (*c* 1.0 in $CHCl_3$); ^{1}H NMR (400 MHz; $CDCl_3$): δ 8.43 (br s, 1H) 7.54 (dd, *J* 7.7 1.9 Hz, 1H), 7.04-6.95 (m, 2H), 4.78 (q, *J* 6.8 Hz, 1H), 3.92 (s, 3H), 1.63 (d, *J* 6.8 Hz, 3H); LRMS *m/z* (ES+) 465 ($[2M+Na]^+$, 100%); Anal. calcd for $C_{11}H_{11}NO_4$: C 59.73%, H 5.01%, N 6.33%; Found: C 59.54%, H 4.90%, N 6.25%. These data are in accordance with the racemate **35**.

(*S*)-2-Methyl-3-oxo-3,4-dihydro-2*H*-1,4-benzoxazine-8-carboxylic acid [(*S*)-36]

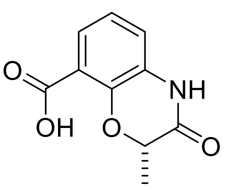

To a solution of (*S*)-**35** (1.09 g, 4.94 mmol, 1.0 eq) in MeOH (40 mL) and THF (40 mL) was added a solution of LiOH aq. (1 M, 25 mL, 5.0 eq). The reaction mixture was stirred at rt for 44 h, then concentrated *in vacuo*, diluted with H_2O (50 mL) and acidified with HCl aq. (1 M). The resulting precipitate was filtered and washed with 1 M HCl (10 mL) and EtOAc (20 mL) to give (*S*)-**36** (640 mg, 3.09 mmol, 63%) as a colorless solid. R_f 0.46 (1:99 AcOH:EtOAc); mp 224 °C (dec.); $[\alpha]_D$ +34.8 (*c* 0.5 in $(CH_3)_2SO$); ^{1}H NMR (400 MHz; $(CD_3)_2SO$): δ 12.83 (br s, 1H), 10.80 (br s, 1H), 7.31 (dd, *J* 7.6 1.9 Hz, 1H), 7.08-6.98 (m, 2H), 4.73 (q, *J* 6.8 Hz, 1H), 1.41 (d, *J* 6.8 Hz, 3H); LRMS *m/z* (ES−) 206 ($[M-H]^-$, 100%). These data are in accordance with the racemate **36**.

(*R*)-2-Methyl-3-oxo-3,4-dihydro-2*H*-1,4-benzoxazine-8-carboxylic acid [(*R*)-36]

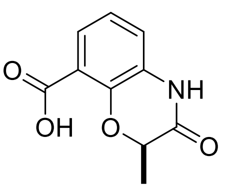

Compound (*R*)-**35** (909 mg, 4.11 mmol, 1.0 eq) was reacted as described above to prepare (*R*)-**36** (643 mg, 3.10 mmol, 76%) as a colorless solid. R_f 0.49 (1:99 AcOH:EtOAc); mp 212 °C (dec.); $[\alpha]_D$ −36.8 (*c* 0.5 in $(CH_3)_2SO$); ^{1}H NMR (400 MHz; $(CD_3)_2SO$): δ 12.83 (br s, 1H), 10.79 (br s, 1H), 7.31 (dd, *J* 7.5 1.9 Hz, 1H), 7.08-6.98 (m, 2H), 4.73 (q, *J* 6.8 Hz, 1H), 1.41 (d, *J* 6.8 Hz, 3H); LRMS *m/z* (ES−) 206 ($[M-H]^-$, 100%). These data are in accordance with the racemate **36**.

(2*S*)-*N*-[2-(2,3-Dihydro-1*H*-indol-1-yl)ethyl]-2-methyl-3-oxo-3,4-dihydro-2*H*-1,4-benzoxazine-8-carboxamide [(*S*)-3]

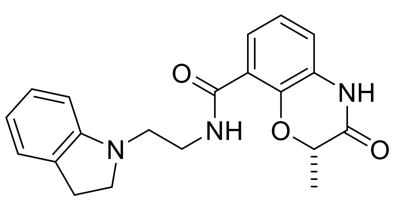

Compound (*S*)-**36** (55 mg, 0.27 mmol, 1.0 eq) and 2-(2,3-dihydro-1*H*-indol-1-yl)ethanamine (48 mg, 0.30 mmol, 1.1 eq) were reacted according to general procedure 1 for 68 h. Purification *via* silica gel chromatography (gradient elution 12 to 100% EtOAc in petroleum ether) gave (*S*)-**3** (66 mg, 0.19 mmol, 71%) as a colorless solid. R_f 0.4 (2:1 EtOAc:petroleum ether); mp 161-163 °C (from MeOH); $[\alpha]_D$ +27.4 (*c* 1.0 in $CHCl_3$); ^1H NMR (400 MHz; $CDCl_3$): δ 8.12 (br s, 1H), 7.93 (br s, 1H), 7.91 (dd, *J* 7.8 1.6 Hz, 1H), 7.14-7.01 (m, 3H), 6.90 (dd, *J* 7.8 1.6 Hz, 1H), 6.69 (dd, *J* 7.0 7.0 Hz, 1H), 6.54 (d, *J* 7.8 Hz, 1H), 4.48 (q, *J* 6.8 Hz, 1H), 3.84-3.67 (m, 2H), 3.51-3.25 (m, 4H), 3.01 (t, *J* 8.3 Hz, 2H), 1.34 (d, *J* 6.8 Hz, 3H); LRMS *m/z* (ES+) 374 ($[M+Na]^+$, 100%); Anal. calcd for $C_{20}H_{21}N_3O_3$: C 68.36%, H 6.02%, N 11.96%; Found: C 68.38%, H 5.96%, N 11.83%. These data are in accordance with the racemate **3**.

(2*R*)-*N*-[2-(2,3-Dihydro-1*H*-indol-1-yl)ethyl]-2-methyl-3-oxo-3,4-dihydro-2*H*-1,4-benzoxazine-8-carboxamide [(*R*)-3]

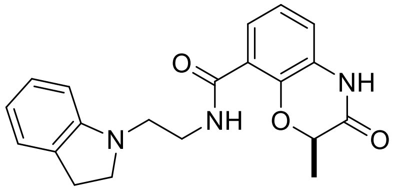

Compound (*R*)-**36** (57 mg, 0.275 mmol, 1.0 eq) was reacted as described above to prepare (*R*)-**3** (85 mg, 0.242 mmol, 88%) as a colorless solid. Crystals for X-ray crystallography were prepared *via* slow evaporation from EtOH and data are reported in Figure S2. R_f 0.4 (2:1 EtOAc:petroleum ether); mp 160-162°C (from EtOH); $[\alpha]_D$ −28.9 (*c* 1.0 in $CHCl_3$); ^1H NMR (400 MHz; $CDCl_3$): δ 8.12 (br s, 1H), 7.97 (br s, 1H), 7.91 (dd, *J* 7.9 1.6 Hz, 1H), 7.14-7.02 (m, 3H), 6.91 (dd, *J* 7.9 1.6 Hz, 1H), 6.69 (dd, *J* 7.4 7.4 Hz, 1H), 6.54 (d, *J* 7.8 Hz, 1H), 4.48 (q, *J* 6.8 Hz, 1H), 3.84-3.67 (m, 2H), 3.52-3.25 (m, 4H), 3.01 (t, *J* 8.3 Hz, 2H), 1.34 (d, *J* 6.8 Hz, 3H); LRMS *m/z* (ES+) 374 ($[M+Na]^+$, 100%); Anal. calcd for $C_{20}H_{21}N_3O_3$: C 68.36%, H 6.02%, N 11.96%; Found: C 68.43%, H 5.94%, N 11.83%. These data are in accordance with the racemate **3**.

Benzyl 2-benzyloxy-3-nitrobenzoate (53)

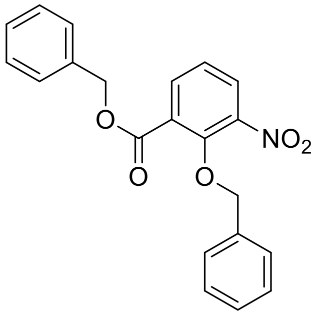

A 5 mL microwave vial was charged with 2-hydroxy-3-nitrobenzoic acid (200 mg, 1.09 mmol, 1.0 eq), K_2CO_3 (755 mg, 5.46 mmol, 5.0 eq) and DMF (3 mL). The vial was sealed and the mixture stirred at rt for 5 min before BnBr (650 µL, 933 mg, 5.46 mmol, 5.0 eq) was added *via* syringe. The reaction mixture was stirred at 70 °C for 39 h. After cooling to rt the mixture was diluted with EtOAc (50 mL), washed with 2 M K_2CO_3 (3 × 50 mL) and brine (50 mL), dried ($MgSO_4$), filtered and concentrated *in vacuo*. Purification *via* silica gel chromatography (gradient elution 2 to 20% EtOAc in petroleum ether) gave **53** (267 mg, 0.735 mmol, 67%) as a pale orange oil. R_f 0.52 (1:4 EtOAc:petroleum ether); ν_{max}/cm^{-1} 3034 (w), 1728 (s), 1531 (s), 1361 (m), 1260 (m); ^{1}H NMR (400 MHz; $CDCl_3$): δ 8.09 (dd, *J* 7.9 1.8 Hz, 1H), 7.93 (dd, *J* 8.0 1.8 Hz, 1H), 7.47-7.34 (m, 10H), 7.29 (dd, *J* 8.0 7.9 Hz, 1H), 5.38 (s, 2H), 5.17 (s, 2H); ^{13}C NMR (101 MHz; $CDCl_3$): δ 164.3, 151.6, 145.9, 135.70, 135.68, 135.2, 128.73, 128.69, 128.62, 128.58, 128.5, 128.2, 124.1, 78.6, 67.7; LRMS *m/z* (ES+) 386 ($[M+Na]^+$, 100%); HRMS *m/z* (ES+) [Found: $(M+Na)^+$ 386.0985. $C_{21}H_{17}NNaO_5^+$ requires 386.0999]; Anal. calcd for $C_{21}H_{17}NO_5$: C 69.41%, 4.72%, 3.85%; Found: C 69.32%, H 4.68%, 3.93%.

Benzyl 3-nitro-2-hydroxybenzoate (54)

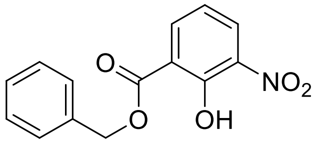

Method 1: 2-hydroxy-3-nitrobenzoic acid (800 mg, 4.37 mmol, 1.0 eq) and $KHCO_3$ (525 mg, 5.24 mmol, 1.2 eq) were dissolved in DMF (12 mL) and stirred at rt for 10 min. BnBr (897 mg, 624 µL, 5.24 mmol, 1.2 eq) was added and the reation mixture was stirred at 40 °C for 23 h. The reaction mixture was diluted with EtOAc (50 mL) and washed with H_2O (2 × 50 mL). Addition of 5% $NaHCO_3$ (50 mL) caused the precipitation of an orange solid that was isolated *via* filtration (eluent EtOAc). The solid was dissolved in 1 M HCl (50 mL) and extracted with EtOAc (50 mL). The organic components were dried ($MgSO_4$), filtered and concentrated *in vacuo* to give **54** (350 mg, 1.28 mmol, 29%) as a yellow solid.

Method 2: A solution of **53** (961 mg, 2.65 mmol, 1.0 eq) in trifluoroacetic acid (13 mL) and toluene (13 mL) was stirred at rt for 1.5 h. The reaction mixture was concentrated *in vacuo* and purified *via* silica gel

chromatography (gradient elution 2 to 80% EtOAc in petroleum ether) to give **54** (721 mg, 2.64 mmol, 99%) as a yellow solid.

R_f 0.38 (1:6 EtOAc:petroleum ether); mp 41-43 °C (from Et_2O); ν_{max}/cm^{-1} 2909 (w), 1672 (s), 1523 (s), 1350 (m), 1266 (s); ^1H NMR (500 MHz; $CDCl_3$): δ 12.0 (br s, 1H), 8.20-8.14 (m, 2H), 7.49-7.37 (m, 5H), 6.99 (t, *J* 8.0 Hz, 1H), 5.44 (s, 2H); ^{13}C NMR (126 MHz; $CDCl_3$): δ 168.5, 155.8, 138.0, 135.8, 134.4, 131.4, 128.9, 128.8, 128.6, 118.4, 115.9, 68.0; LRMS *m/z* (ES+) 296 ($[M+Na]^+$, 100%); HRMS *m/z* (ES+) [Found: $(M+Na)^+$ 296.0527. $C_{14}H_{11}NNaO_5^+$ requires 296.0529]; Anal. calcd for $C_{14}H_{11}NO_5$: C 61.54%, 4.06%, 5.13%; Found: C 61.60%, H 3.92%, 5.13%.

Benzyl 3-nitro-2-{[(trifluoromethyl)sulfonyl]oxy}benzoate (55)

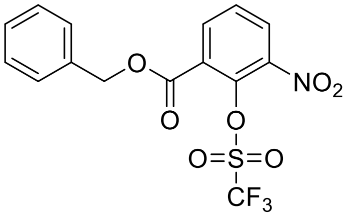

To a solution of **54** (1.65 g, 6.04 mmol, 1.0 eq) and Et_3N (915 mg, 1.26 mL, 9.06 mmol, 1.5 eq) in CH_2Cl_2 (50 mL) at 0 °C was added trifluoromethanesulfonic anhydride (2.05 g, 1.22 mL, 7.25 mmol, 1.2 eq) dropwise over 5 min. The reaction mixture was allowed to warm to rt, stirred for a further 3 h, then concentrated *in vacuo* and purified *via* silica gel chromatography (gradient elution 5 to 40% EtOAc in petroleum ether) to give **55** (2.25 g, 5.55 mmol, 92%) as a yellow oil. R_f 0.22 (1:10 EtOAc:petroleum ether to 1:5 EtOAc:petroleum ether); ν_{max}/cm^{-1} 3265 (w), 1732 (s), 1542 (s), 1431 (s), 1352 (m), 1267 (s); ^1H NMR (400 MHz; $CDCl_3$): δ 8.30 (dd, *J* 8.0 1.7 Hz, 1H), 8.21 (dd, *J* 8.0 1.7 Hz, 1H), 7.63 (dd, *J* 8.0 8.0 Hz, 1H), 7.51-7.45 (m, 2H), 7.45-7.36 (m, 3H), 5.45 (s, 2H); ^{13}C NMR (101 MHz; $CDCl_3$): δ 162.4, 143.1, 140.1, 136.7, 134.4, 129.8, 128.9, 128.9, 128.7, 128.6, 127.8, 118.4 (q, *J* 320.9 Hz), 68.5; ^{19}F NMR (377 MHz; $CDCl_3$): δ −73.4 (s, 3F); LRMS *m/z* (ES+) 833 ($[2M+Na]^+$, 100%); HRMS *m/z* (ES+) [Found: $(M+Na)^+$ 428.0015. $C_{15}H_{10}F_3NNaO_7S^+$ requires 428.0022]; Anal. calcd for $C_{15}H_{10}F_3NO_7S$: C 44.45%, H 2.49%, N 3.46%; Found: C 44.49%, H 2.40%, N 3.42%.

Benzyl 2-{[(2'*R*)-1'-methoxy-1'-oxopropan-2'-yl]amino}-3-nitrobenzoate [(*R*)-13]

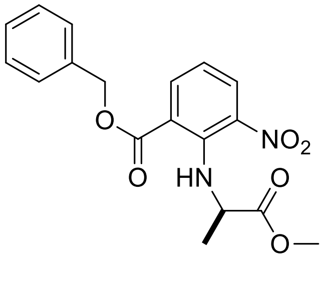

To a solution of **55** (3.37 g, 8.31 mmol, 1.0 eq) in toluene (150 mL) was added sequentially Cs_2CO_3 (8.13 g, 24.9 mmol, 3.0 eq) and D-alanine methyl ester hydrochloride (1.74 g, 12.5 mmol, 1.5 eq). The resulting yellow solution was heated to 85 ºC causing a deep orange color to form and stirring was continued at 85 ºC for 19 h. Upon cooling to rt the reaction mixture was filtered through Celite® (eluent toluene) and concentrated *in vacuo*. Purification *via* silica gel chromatography (gradient elution 3 to 28% EtOAc in petroleum ether) yielded (*R*)-**13** (2.36 g, 6.59 mmol, 79%) as a yellow oil. Data reported above.

Methyl 3-nitro-2-{[(trifluoromethyl)sulfonyl]oxy}benzoate (56)

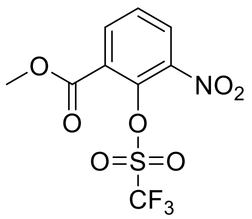

To a solution of methyl 3-nitro-2-hydroxybenzoate (556 mg, 2.82 mmol, 1.0 eq) and Et_3N (428 mg, 590 µL, 4.23 mmol, 2.0 eq) in CH_2Cl_2 (12 mL) at 0 °C was added trifluoromethanesulfonic anhydride (839 mg, 500 µL, 2.97 mmol, 1.1 eq) dropwise over 5 min. The reaction mixture was allowed to warm to rt, stirred for a further 2 h, then concentrated *in vacuo* and purified *via* silica gel chromatography (gradient elution 6 to 42% EtOAc in petroleum ether) to give **56** (836 mg, 2.42 mmol, 86%) as a yellow oil. R_f 0.29 (1:4 EtOAc:petroleum ether); ν_{max}/cm^{-1} 2973 (w), 1735 (s), 1543 (s), 1431 (s), 1353 (m), 1208 (s); ^1H NMR (400 MHz; $CDCl_3$): δ 8.32 (dd, *J* 8.0 1.8 Hz, 1H), 8.23 (dd, *J* 8.1 1.8 Hz, 1H), 8.02 (dd, *J* 8.1 8.0 Hz, 1H), 4.01 (s, 3H); ^{13}C NMR (100 MHz; $CDCl_3$): δ 163.2, 143.2, 140.2, 136.9, 129.9, 128.6, 127.5, 118.4 (q, *J* 322.2 Hz), 53.3; ^{19}F NMR (377 MHz; $CDCl_3$): δ −73.6 (s, 3F); LRMS *m/z* (ES+) 352 ([M+Na]$^+$, 30%), 413 (100%); HRMS *m/z* (ES+) [Found: (M+Na)$^+$ 351.9702. $C_9H_6F_3NNaO_7S$ requires 351.9709]; Anal. calcd for $C_9H_6F_3NO_7S^+$: C 32.84%, H 1.84%, N 4.25%; Found: C 32.98%, H 1.84%, N 4.19%.

Methyl 2-[(1-methoxy-1-oxopropan-2-yl)amino]-3-nitrobenzoate (57)

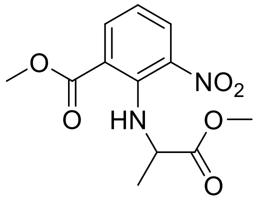

A microwave flask was charged with DL-alanine methyl ester hydrochloride (237 mg, 1.70 mmol, 1.4 eq), $Pd(OAc)_2$ (13 mg, 0.058 mmol, 0.05 eq), BINAP (70 mg, 0.112 mmol, 0.1 eq), and Cs_2CO_3 (1.11 g, 3.41 mmol, 2.9 eq) and sealed under Ar. Toluene (20 mL) was added and the resulting solution was heated to 80 °C for 5 min. A solution of **56** (390 mg, 1.18 mmol, 1 eq) in toluene (5 mL) was added dropwise over 30

min and the reaction mixture stirred at 80 °C for 2.5 h. After cooling to rt the slurry was washed with H_2O (3 × 25 mL) and the organic components concentrated *in vacuo*. Purification *via* silica gel chromatography (gradient elution 5 to 40% EtOAc in petroleum ether) gave **57** (253 mg, 0.896 mmol, 76%). R_f 0.60 (1:2 EtOAc:petroleum ether); ν_{max}/cm^{-1} 2955 (w), 1745 (s), 1698 (s), 1532 (s), 1346 (m), 1260 (s); ^{1}H NMR (500 MHz; $CDCl_3$): δ 8.76 (d, *J* 8.0 Hz, 1H), 8.12 (dd, *J* 7.9 1.7 Hz, 1H), 7.97 (dd, *J* 8.1 1.7 Hz, 1H), 6.79 (dd, *J* 8.1 7.9 Hz, 1H), 4.09-4.01 (m, 1H), 3.94 (s, 3H), 3.72 (s, 3H), 1.46 (d, *J* 7.0 Hz, 3H); ^{13}C NMR (126 MHz; $CDCl_3$): δ 173.4, 167.5, 144.0, 138.7, 137.0, 131.2, 118.4, 116.3, 53.9, 52.5, 52.5, 19.1; LRMS *m/z* (ES+) 587 ($[2M+Na]^+$, 100%); HRMS *m/z* (ES+) [Found: $(M+Na)^+$ 305.0744. $C_{12}H_{14}N_2NaO_6$ requires 305.0748]; Anal. calcd for $C_{12}H_{14}N_2O_6$: C 51.06%, H 5.00%, N 9.93%; Found: C 51.13%, H 4.96%, N 9.88%.

Methyl 3-methyl-2-oxo-1,2,3,4-tetrahydroquinoxaline-5-carboxylate (58)

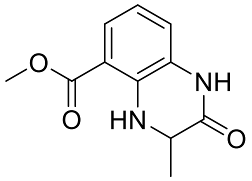

A mixture of **57** (238 mg, 0.843 mmol, 1.0 eq) and Pd/C (45 mg, 0.42 mmol, 0.05 eq) in EtOAc (10 mL) was placed under a H_2 atmosphere. The reaction mixture was stirred for 18 h, then filtered through Celite® (eluent EtOAc), concentrated *in vacuo* and purificied *via* silica gel chromatography (gradient elution 8 to 64% EtOAc in petroleum ether) to give **58** (154 mg, 0.699 mmol, 83%) as a colorless solid. R_f 0.29 (1:2 EtOAc:petroleum ether); mp 152-154 °C (from EtOAc); ν_{max}/cm^{-1} 3348 (w), 2953 (w), 1671 (s), 1436 (m), 1238 (s); ^{1}H NMR (500 MHz; $CDCl_3$): δ 9.03 (br s, 1H), 7.62-7.55 (m, 2H), 6.88 (d, *J* 7.7 Hz, 1H), 6.66 (dd, *J* 7.9 7.7 Hz, 1H), 4.19 (qd, *J* 6.6 1.6 Hz, 1H), 3.89 (s, 3H), 1.52 (d, *J* 6.6 Hz, 3H); ^{13}C NMR (126 MHz; $CDCl_3$): δ 168.8, 168.4, 137.6, 126.0, 125.7, 119.1, 116.6, 111.3, 51.8, 51.1, 18.9; LRMS *m/z* (ES+) 463 ($[2M+Na]^+$, 100%); HRMS *m/z* (ES+) [Found: $(M+Na)^+$ 243.0735. $C_{11}H_{12}N_2NaO_3^+$ requires 243.0740]; Anal. calcd for $C_{11}H_{12}N_2O_3$: C 59.99%, H 5.49%, N 12.72%; Found: C 59.89%, H 5.42%, N 12.69%.

3-Methyl-2-oxo-1,2,3,4-tetrahydroquinoxaline-5-carboxylic acid (15)

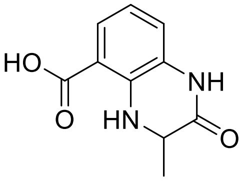

To a solution of **58** (54 mg, 0.245 mmol, 1.0 eq) in MeOH (2 mL) and THF (2 mL) was added a solution of LiOH aq. (2 M, 1.25 mL, 10 eq). The reaction mixture was stirred at rt for 24 h, then concentrated *in vacuo*, diluted with H_2O (50 mL) and acidified with HCl aq. (1 M). The resulting precipitate was extracted into EtOAc (50 mL), dried ($MgSO_4$), filtered and concentrated *in vacuo* to give **15** (33 mg, 0.160 mmol, 65%) as a

yellow solid that was used without further purification. R_f 0.28 (EtOAc); mp 230 °C (dec.); ^{1}H NMR (400 MHz; $(CD_3)_2SO$): δ 12.91 (br s, 1H), 10.46 (s, 1H), 7.60 (s, 1H), 7.42 (dd, *J* 7.9 1.4 Hz, 1H), 6.92 (dd, *J* 7.7 1.4 Hz, 1H), 6.63 (dd, *J* 7.9 7.7 Hz, 1H), 4.09-4.00 (m, 1H), 1.29 (d, *J* 6.7 Hz, 3H); LRMS *m/z* (ES−) 205 ($[M-H]^-$, 100%). These data are in accordance with (*R*)-**15**.

7-methoxy-3,4-dihydroquinolin-2(1*H*)-one (60)

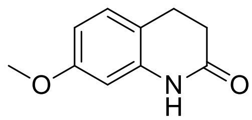

To a solution of 7-hydroxy-3,4-dihydroquinolin-2(1*H*)-one (900 mg, 5.52 mmol, 1.0 eq) and K_2CO_3 (1.52 g, 11.0 mmol, 2.0 eq) in DMF (9 mL) was added MeI (378 µL, 861 mg, 6.1 mmol, 1.1 eq). The reaction mixture was heated to 60 °C for 16 h before being diluted with EtOAc (200 mL), washed with sat. aq. K_2CO_3 (3 × 200 mL) and brine (200 mL), dried ($MgSO_4$), filtered and concentrated *in vacuo*. Purification *via* crystallisation from EtOH yielded **60** (554 mg, 3.13 mmol, 57%) as colorless needles. R_f 0.22 (1:2 EtOAc:petroleum ether); mp 142-143 °C (from EtOH, lit.[48] 147 °C); ^{1}H NMR (400 MHz; $CDCl_3$): δ 7.51 (br s, 1H), 7.07 (d, *J* 8.3 Hz, 1H), 6.54 (dd, *J* 8.3 2.4 Hz, 1H), 6.29 (d, J 2.4 Hz, 1H), 3.79 (s, 3H), 2.91 (t, *J* 7.5 Hz, 2H), 2.63 (t, *J* 7.5 Hz, 2H); LRMS *m/z* (ES+) 200 ($[M+Na]^+$, 100%). These data are in accordance with the literature.[48]

7-methoxy-1,2,3,4-tetrahydroquinoline (61)

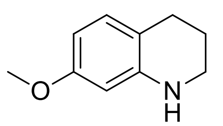

To a solution of **60** (251 mg, 1.42 mmol, 1.0 eq) in THF (17 mL) at 0 °C was added a solution of $LiAlH_4$ in THF (1M, 1.70 mL, 1.70 mmol, 1.2 eq) dropwise over 5 min. The reaction mixture was allowed to warm to rt and stirred for 13 h before being cooled to 0 °C and quenched by sequential addition of EtOAc (2 mL) and H_2O (1 mL). The resulting mixture was dried ($MgSO_4$), filtered through Celite® (eluent EtOAc) and concentrated *in vacuo* to give **61** (230 mg, 1.41 mmol, 99%) as a yellow oil which was used without further purification. R_f 0.51 (1:2 EtOAc:petroleum ether); ^{1}H NMR (400 MHz; $CDCl_3$): δ 6.85 (d, *J* 8.3 Hz, 1H), 6.21 (dd, *J* 8.3 2.5 Hz, 1H), 6.05 (d, *J* 2.5 Hz, 1H), 3.92 (br s, 1H), 3.74 (s, 3H), 3.29 (t, *J* 5.5 Hz, 2H), 2.70 (t, *J* 6.4 Hz, 2H), 1.96-1.89 (m, 2H); LRMS *m/z* (ES+) 164 ($[M+H]^+$, 100%). These data are in accordance with the literature.[49]

Methyl 3-(7-methoxy-3,4-dihydroquinolin-1(2*H*)-yl)propanoate (65)

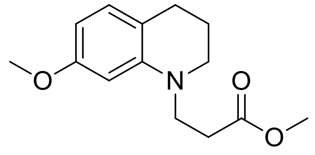

A solution of **61** (507 mg, 3.11 mmol, 1.0 eq) and methyl prop-2-enoate (560 μL, 535 mg, 6.21 mmol, 2.0 eq) in acetic acid (2.5 mL) was heated at 120 °C for 39 h. The reaction mixture was concentrated *in vacuo* and purified *via* silica gel chromatography (gradient elution 3 to 28% EtOAc in petroleum ether) to yield **65** (359 mg, 1.44 mmol, 46%) as a brown oil. R_f 0.17 (1:4 EtOAc:petroleum ether); ν_{max}/cm^{-1} 2943 (m), 1732 (s), 1611 (s), 1508 (s), 1161 (s); ^{1}H NMR (400 MHz; $CDCl_3$): δ 6.87 (d, *J* 8.6 Hz, 1H), 6.22-6.16 (m, 2H), 3.78 (s, 3H), 3.70 (s, 3H), 3.60 (t, *J* 7.2 Hz, 2H), 3.28 (t, *J* 5.6 Hz, 2H), 2.69 (t, *J* 6.4 Hz, 2H), 2.62 (t, *J* 7.2 Hz, 2H), 1.97-1.88 (m, 2H); ^{13}C NMR (101 MHz; $CDCl_3$): δ 172.8, 159.2, 145.3, 129.7, 115.5, 110.3, 97.4, 55.2, 51.7, 49.4, 47.2, 31.0, 27.3, 22.4; LRMS *m/z* (ES+) 250 ($[M+H]^+$, 100%); HRMS *m/z* (ES+) [Found: $(M+Na)^+$ 272.1266. $C_{14}H_{19}NNaO_3$ requires 272.1257]; Anal. calcd for $C_{14}H_{19}NO_3$: C 67.45%, H 7.68%, N 5.62%; Found: C 67.38%, H 7.79%, N 5.72%.

3-(7-methoxy-3,4-dihydroquinolin-1(2*H*)-yl)propan-1-ol (66)

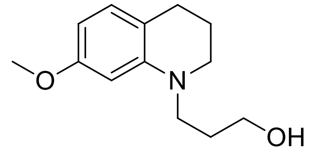

To a solution of **65** (338 mg, 1.54 mmol, 1.0 eq) in THF (10 mL) at 0 °C was added a solution of $LiAlH_4$ in THF (1M, 1.85 mL, 1.85 mmol, 1.2 eq) dropwise over 5 min. The reaction mixture was allowed to warm to rt and stirred for 23 h before being cooled to 0 °C and quenched by sequential addition of EtOAc (2 mL) and H_2O (1 mL). The resulting mixture was dried ($MgSO_4$), filtered through Celite® (eluent EtOAc) and concentrated *in vacuo* to give **66** (275 mg, 1.24 mmol, 81%) as a brown oil that was used without further purification. R_f 0.36 (2:1 EtOAc:petroleum ether); ν_{max}/cm^{-1} 3370 (br) 2934 (m), 1611 (s), 1508 (s), 1166 (s); ^{1}H NMR (300 MHz; $CDCl_3$): δ 6.86 (d, *J* 8.1 Hz, 1H), 6.24 (d, *J* 2.4 Hz, 1H), 6.17 (dd, *J* 8.1 2.4 Hz, 1H), 3.78 (s, 3H), 3.75 (t, *J* 6.0 Hz, 2H), 3.38 (t, *J* 7.0 Hz, 2H), 3.28 (t, *J* 5.6 Hz, 2H), 2.71 (t, *J* 6.3 Hz, 2H), 2.02 (br s, 1H), 1.99-1.91 (m, 2H), 1.91-1.81 (m, 2H); ^{13}C NMR (76 MHz; $CDCl_3$): δ 159.2, 146.3, 129.5, 115.5, 100.0, 97.7, 60.9, 55.5, 49.5, 48.5, 29.4, 27.4, 22.4; LRMS *m/z* (ES+) 221 ($[M+H]^+$, 100%); HRMS *m/z* (ES+) [Found: $(M+Na)^+$ 244.1315. $C_{13}H_{19}NNaO_2$ requires 244.1308]; Anal. calcd for $C_{13}H_{19}NO_2$: C 70.56%, H 8.65%, N 6.33%; Found: C 70.50%, H 8.63%, N 6.39%.

3-(3,4-Dihydroquinolin-1(2*H*)-yl)propan-1-ol (67)

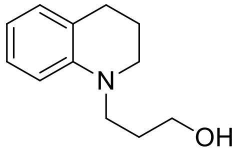

1,2,3,4-tetrahydroquinoline (190 mg, 1.43 mmol, 1.0 eq) was reacted according to general procedure 4. Purification *via* silica gel chromatography (gradient elution 5 to 40% EtOAc in petroleum ether) yielded **67** (161 mg, 0.843 mmol, 59%) as a brown oil. R_f 0.14 (1:4 EtOAc:petroleum ether); ν_{max}/cm^{-1} 3352 (br) 2930 (m), 1601 (s), 1505 (s); ^{1}H NMR (500 MHz; $CDCl_3$): δ 7.06 (dd, *J* 8.0 7.5, 1H), 6.96 (d, *J* 7.0, 1H), 6.67 (d, *J* 8.0, 1H), 6.59 (ddd, *J* 7.5 7.0 0.9 Hz, 1H), 3.77 (t, *J* 6.0 Hz, 2H), 3.41 (t, *J* 7.0 Hz, 2H), 3.30 (t, *J* 5.7 Hz, 2H), 2.77 (t, *J* 6.4 Hz, 2H), 2.00-1.94 (m, 2H), 1.91-1.84 (m, 2H), 1.83 (br s, 1H); ^{13}C NMR (126 MHz; $CDCl_3$): δ 145.4, 129.2, 127.1, 122.7, 115.8, 111.0, 61.1, 49.6, 48.7, 29.3, 28.1, 22.2; LRMS *m/z* (ES+) 214 ([M+Na]$^+$, 100%); HRMS *m/z* (ES+) [Found: (M+Na)$^+$ 214.1204. $C_{12}H_{17}NNaO^+$ requires 214.1202]; Anal. calcd for $C_{12}H_{17}NO$: C 75.35%, H 8.96%, N 7.32%; Found: C 75.22%, H 9.14%, N 7.19%.

3-(6-Methoxy-3,4-dihydroquinolin-1(2*H*)-yl)propan-1-ol (68)

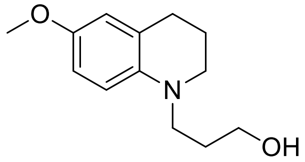

6-methoxy-1,2,3,4-tetrahydroquinoline (206 mg, 1.26 mmol, 1.0 eq) was reacted according to general procedure 4. Purification *via* silica gel chromatography (gradient elution 8 to 66% EtOAc in petroleum ether) yielded **68** (152 mg, 0.687 mmol, 55%) as a brown oil. R_f 0.28 (1:2 EtOAc:petroleum ether); ν_{max}/cm^{-1} 3372 (br), 2933 (m), 1504 (s), 1240 (s); ^{1}H NMR (400 MHz; $CDCl_3$): δ 6.73-6.67 (m, 2H), 6.63-6.60 (m, 1H), 3.79 (t, *J* 6.2 Hz, 2H), 3.77 (s, 3H), 3.36 (t, *J* 6.6 Hz, 2H), 3.22 (t, *J* 5.6 Hz, 2H), 2.78 (t, *J* 6.5 Hz, 2H), 2.54 (br s, 1H), 2.01-1.92 (m, 2H), 1.86 (tt, *J* 6.6 6.2 Hz, 2H); ^{13}C NMR (101 MHz; $CDCl_3$): δ 151.2, 140.2, 124.7, 115.2, 113.2, 112.5, 61.6, 55.8, 50.2, 49.5, 29.2, 28.3, 22.3; LRMS *m/z* (ES+) 222 ([M+H]$^+$, 100%); HRMS *m/z* (ES+) [Found: (M+H)$^+$ 222.1483. $C_{13}H_{20}NO_2^+$ requires 222.1489].

3-(2,3-Dihydro-4*H*-1,4-benzoxazin-4-yl)propan-1-ol (69)

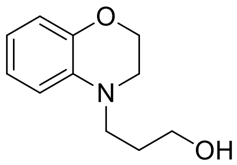

A solution of 3,4-dihydro-2*H*-1,4-benzoxazine (179 mg, 1.32 mmol, 1.0 eq) was reacted according to general procedure 4. Purification *via* silica gel chromatography (gradient elution 8 to 66% EtOAc in petroleum ether)

yielded **69** (131 mg, 0.483 mmol, 51%) as a brown oil. R_f 0.20 (1:2 EtOAc:petroleum ether); ν_{max}/cm^{-1} 3359 (br), 2935 (m), 2877 (m), 1503 (s), 1056 (s); ^{1}H NMR (400 MHz; $CDCl_3$): δ 6.87-6.81 (m, 1H), 6.79 (dd, *J* 7.7 1.5 Hz, 1H), 6.74 (dd, *J* 8.1 1.3 Hz, 1H), 6.63 (ddd, *J* 7.7 7.5 1.3 Hz, 1H), 4.24 (t, *J* 4.4 Hz, 2H), 3.76 (t, *J* 6.2 Hz, 2H), 3.39 (t, *J* 6.7 Hz, 2H), 3.34 (t, *J* 4.4 Hz, 2H), 1.86 (tt, *J* 6.7 6.2 Hz, 2H), 1.82 (br s, 1H); ^{13}C NMR (101 MHz; $CDCl_3$): δ 144.2, 135.3, 121.6, 117.7, 116.4, 112.6, 64.4, 60.9, 48.4, 47.2, 29.1; LRMS *m/z* (ES+) 194 ($[M+H]^+$, 100%); HRMS *m/z* (ES+) [Found: $(M+H)^+$ 194.11780. $C_{11}H_{16}NO_2^+$ requires 194.1176].

1-(3-azidopropyl)-7-methoxy-1,2,3,4-tetrahydroquinoline (70)

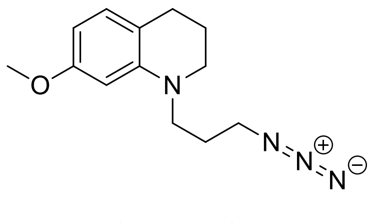

To a solution of **66** (265 mg, 1.20 mmol, 1.0 eq) in CH_2Cl_2 (7 mL) at 0 °C was added Et_3N (242 mg, 334 µL, 2.39 mmol, 2.0 eq) and stirred for 5 min. MsCl (164 mg, 111 µL, 1.44 mmol, 1.2 eq) was added and the reaction mixture was stirred at rt for 1 h before being concentrated *in vacuo*. The resulting residue was passed through a plug of silica gel (eluent EtOAc) and concentrated *in vacuo* to give 3-(7-methoxy-3,4-dihydroquinolin-1(2*H*)-yl)propyl methanesulfonate (348 mg, 1.16 mmol, 97%) as a brown oil. A solution of the sulfonate (348 mg, 1.16 mmol, 1.0 eq) and NaN_3 (302 mg, 4.65 mmol, 4.0 eq) in DMF (10 mL) was heated at 60 °C for 75 min. The reaction mixture was cooled to rt, then diluted with EtOAc (40 mL), washed with H_2O (2 × 40 mL) and brine (40 mL), dried ($MgSO_4$), filtered and concentrated *in vacuo*. The crude material was purified *via* silica gel chromatography (gradient elution 1 to 12% EtOAc in petroleum ether) to give **70** (213 mg, 0.946 mmol, 82%) as an brown oil. R_f 0.77 (1:2 EtOAc:petroleum ether); ν_{max}/cm^{-1} 2933 (m), 2091 (s), 1609 (s), 1507 (s), 1263 (s); ^{1}H NMR (400 MHz; $CDCl_3$): δ 6.91-6.87 (m, 1H), 6.21-6.17 (m, 2H), 3.80 (s, 3H), 3.41 (t, *J* 6.5 Hz, 2H), 3.36 (t, *J* 7.1 Hz, 2H), 3.28 (t, *J* 5.6 Hz, 2H), 2.72 (t, *J* 6.3 Hz, 2H), 1.99-1.87 (m, 4H); ^{13}C NMR (101 MHz; $CDCl_3$): δ 159.3, 145.9, 129.6, 115.3, 100.0, 97.4, 55.2, 49.7, 49.3, 48.6, 27.4, 25.8, 22.4; LRMS *m/z* (ES+) 247 ($[M+H]^+$, 100%); HRMS *m/z* (ES+) [Found: $(M+H)^+$ 247.1556. $C_{13}H_{19}N_4O^+$ requires 247.1553].

1-(3-Azidopropyl)-1,2,3,4-tetrahydroquinoline (71)

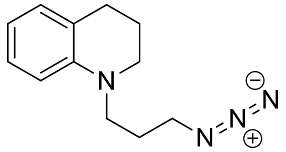

Compound **67** (82 mg, 0.43 mmol, 1.0 eq) was reacted according to general procedure 5. The crude material was passed through a pad of silica (eluent EtOAc) to give **71** (43 mg, 0.95 mmol, 46%) as a brown oil. R_f 0.88

(1:2 EtOAc:petroleum ether); ν_{max}/cm^{-1} 2929 (m), 2090 (s), 1601 (s), 1503 (s); ^{1}H NMR (400 MHz; $CDCl_3$): δ 7.10 (dd, *J* 7.8 7.8 Hz, 1H), 6.99 (d, *J* 7.5 Hz, 1H), 6.66-6.58 (m, 2H), 3.46-3.35 (m, 4H), 3.31 (t, *J* 5.8 Hz, 2H), 2.80 (t, *J* 6.2 Hz, 2H), 1.99 (tt, *J* 6.2 5.8 Hz, 2H), 1.91 (tt, *J* 6.8 6.8 Hz, 2H); ^{13}C NMR (101 MHz; $CDCl_3$): δ 145.1, 129.3, 127.2, 122.5, 115.8, 110.5, 49.8, 49.3, 48.5, 28.1, 25.9, 22.2; LRMS *m/z* (ES+) 217 ($[M+H]^+$, 100%); HRMS *m/z* (ES+) [Found: $(M+H)^+$ 217.1444. $C_{12}H_{17}N_4^+$ requires 217.1448].

1-(3-Azidopropyl)-6-methoxy-1,2,3,4-tetrahydroquinoline (72)

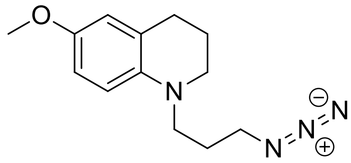

Compound **68** (107 mg, 0.484 mmol, 1.0 eq) was reacted according to general procedure 5. The crude material was passed through a pad of silica (eluent EtOAc) to give **72** (104 mg, 0.422 mmol, 87%) as a red oil. R_f 0.80 (1:2 EtOAc:petroleum ether); ν_{max}/cm^{-1} 2951 (m), 2092 (s), 1504 (s), 1240 (s); ^{1}H NMR (400 MHz; $CDCl_3$): δ 6.66 (dd, *J* 8.8 3.0 Hz, 1H), 6.59 (d, *J* 3.0 Hz, 1H), 6.53 (d, *J* 8.8 Hz, 1H), 3.73 (s, 3H), 3.38 (t, *J* 6.7 Hz, 2H), 3.30 (t, *J* 6.9 Hz, 2H), 3.20 (t, *J* 5.6 Hz, 2H), 2.74 (t, *J* 6.4 Hz, 2H), 1.99-1.90 (m, 2H), 1.85 (tt, *J* 6.9 6.7 Hz, 2H); ^{13}C NMR (101 MHz; $CDCl_3$): δ 150.9, 139.8, 124.1, 115.4, 112.5, 112.0, 55.8, 49.7, 49.4, 49.2, 28.3, 25.9, 22.4; LRMS *m/z* (ES+) 247 ($[M+H]^+$, 100%); HRMS *m/z* (ES+) [Found: $(M+Na)^+$ 247.1560. $C_{13}H_{19}N_4O^+$ requires 247.1553].

4-(3-Azidopropyl)-3,4-dihydro-2*H*-1,4-benzoxazine (73)

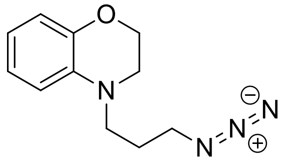

Compound **69** (105 mg, 0.543 mmol, 1.0 eq) was reacted according to general procedure 5. The crude material was passed through a pad of silica (eluent EtOAc) to give **73** (109 mg, 0.499 mmol, 92%) as a brown oil. R_f 0.77 (1:2 EtOAc:petroleum ether); ν_{max}/cm^{-1} 2933 (m), 2878 (m), 2092 (s), 1502 (s), 1057 (s); ^{1}H NMR (500 MHz; $CDCl_3$): δ 6.87-6.81 (m, 1H), 6.79 (dd, J 7.7 1.5 Hz, 1H), 6.67 (dd, *J* 8.1 1.3 , 1H), 6.63 (ddd, J 7.7 7.6 1.3 Hz, 1H), 4.24 (t, *J* 4.4 Hz, 2H), 3.41 (t, *J* 6. 6 Hz, 2H), 3.38-3.30 (m, 4H), 1.88 (tt, *J* 6.8 6.6 Hz, 2H); ^{13}C NMR (126 MHz; $CDCl_3$): δ 144.2, 135.1, 121.8, 117.8, 116.6, 112.2, 64.6, 49.3, 48.3, 47.5, 26.0; LRMS *m/z* (ES+) 193 ($[M+H]^+$, 100%); HRMS *m/z* (ES+) [Found: $(M+H)^+$ 193.1329. $C_{11}H_{17}N_2O^+$ requires 193.1335].

3-(7-methoxy-3,4-dihydroquinolin-1(2*H*)-yl)propan-1-amine (74)

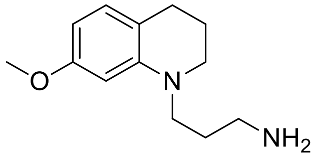

Compound **70** (213 mg, 0.865 mmol, 1.0 eq) was reacted according to general procedure 6 to give **74** (190 mg, 0.862 mmol, 99%) as a grey oil without need for further purifiaction. R_f 0.18 (1:9 MeOH:$CHCl_3$); ν_{max}/cm^{-1} 3353 (br), 2931 (m), 1611 (s), 1508 (s), 1166 (s); ^{1}H NMR (500 MHz; CD_3OD): δ 6.76 (d, *J* 8.1 Hz, 1H), 6.14 (d, *J* 2.3 Hz, 1H), 6.09 (dd, *J* 8.1 2.3 Hz, 1H), 3.71 (s, 3H), 3.31-3.26 (m, 2H), 3.25 (t, *J* 5.6 Hz, 2H), 2.71 (t, *J* 7.3 Hz, 2H), 2.64 (t, *J* 6.3 Hz, 2H), 1.92-1.86 (m, 2H), 1.80-1.72 (m, 2H); ^{13}C NMR (126 MHz; CD_3OD): δ 161.0, 147.7, 130.9, 116.9, 101.8, 99.0, 55.9, 50.9, 50.5, 41.0, 30.7, 28.9, 24.1; LRMS *m/z* (ES+) 221 ($[M+H]^+$, 100%); HRMS *m/z* (ES+) [Found: $(M+H)^+$ 221.1644. $C_{13}H_{21}N_2O^+$ requires 221.1648].

3-(3,4-Dihydroquinolin-1(2*H*)-yl)propan-1-amine (75)

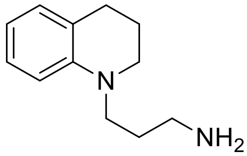

Compound **71** (43 mg, 0.20 mmol, 1.0 eq) was reacted according to general procedure 6 to give **75** (37 mg, 0.20 mmol, 99%) as a grey oil without need for further purification. R_f 0.16 (1:9 MeOH:$CHCl_3$); ν_{max}/cm^{-1} 3344 (br), 2928 (m), 1600 (s), 1500 (s); ^{1}H NMR (500 MHz; CD_3OD): δ 6.96 (dd, *J* 7.9 7.4 Hz, 1H), 6.85 (d, *J* 7.3 Hz, 1H), 6.59 (d, *J* 7.9 Hz, 1H), 6.48 (dd, *J* 7.4 7.3 Hz, 1H), 3.30 (t, *J* 7.4 Hz, 2H), 3.25 (t, *J* 5.6 Hz, 2H), 2.77-2.67 (m, 4H), 1.95-1.86 (m, 2H), 1.77 (tt, *J* 7.4 7.3 Hz, 2H); ^{13}C NMR (126 MHz; CD_3OD): δ 146.9, 130.5, 128.4, 124.2, 117.3, 112.5, 51.0, 50.4, 40.8, 30.1, 29.7, 23.9; LRMS *m/z* (ES+) 191 ($[M+H]^+$, 100%); HRMS *m/z* (ES+) [Found: $(M+H)^+$ 191.1547. $C_{12}H_{19}N_2^+$ requires 191.1543].

3-(6-Methoxy-3,4-dihydroquinolin-1(2*H*)-yl)propan-1-amine (76)

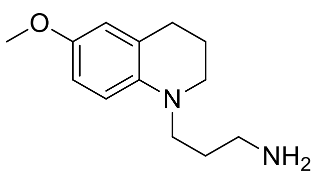

Compound **72** (90 mg, 0.37 mmol, 1.0 eq) was reacted according to general procedure 6 to give **76** (69 mg, 0.31 mmol, 86%) as a yellow oil without need for further purification. R_f 0.11 (1:9 MeOH:$CHCl_3$); ν_{max}/cm^{-1} 3289 (br), 2931 (m), 1503 (s), 1238 (s); ^{1}H NMR (500 MHz; CD_3OD): δ 6.62 (dd, *J* 8.9 2.8 Hz, 1H), 6.59 (d, *J* 8.9 Hz, 1H), 6.55 (d, *J* 2.8 Hz, 1H), 3.67 (s, 3H), 3.24 (t, *J* 7.3 Hz, 2H), 3.18 (t, *J* 5.6 Hz, 2H), 2.73-2.66 (m, 4H), 1.95-1.88 (m, 2H), 1.72 (tt, *J* 7.3 7.3 Hz, 2H); ^{13}C NMR (126 MHz; CD_3OD): δ 152.8, 141.7, 126.0,

116.6, 114.3, 113.9, 56.6, 51.3, 50.9, 41.2, 30.8, 29.8, 24.0; LRMS *m/z* (ES+) 221 ($[M+H]^+$, 100%); HRMS *m/z* (ES+) [Found: $(M+H)^+$ 221.1644. $C_{13}H_{21}N_2O^+$ requires 221.1648].

3-(2,3-Dihydro-4*H*-1,4-benzoxazin-4-yl)propan-1-amine (77)

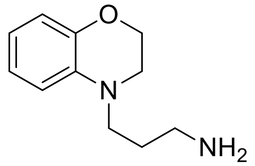

Compound **73** (90 mg, 0.41 mmol, 1.0 eq) was reacted according to general procedure 6 to give **77** (76 mg, 0.40 mmol, 96%) as a colorless oil without need for further purification. R_f 0.08 (1:9 MeOH:$CHCl_3$); ν_{max}/cm^{-1} 3302 (br), 2933 (m), 2874 (m), 1502 (s), 1056 (s); ^1H NMR (400 MHz; CD_3OD): δ 6.79-6.69 (m, 2H), 6.67 (dd, *J* 7.9 1.3 Hz, 1H), 6.55 (m, 1H), 4.19 (t, *J* 4.4 Hz, 2H), 3.37-3.30 (m, 4H), 2.83 (t, *J* 7.4 Hz, 2H), 1.84 (tt, *J* 7.4 7.4 Hz, 2H); ^{13}C NMR (126 MHz; CD_3OD): δ 145.6, 136.6, 122.5, 118.3, 117.1, 113.5, 65.6, 49.6, 48.1, 40.5, 30.2; LRMS *m/z* (ES+) 193 ($[M+H]^+$, 100%); HRMS *m/z* (ES+) [Found: $(M+H)^+$ 193.1329. $C_{11}H_{17}N_2O^+$ requires 193.1335].

References

[1] Flack, H. D. On enantiomorph-polarity estimation. *Acta Cryst.* **1983**, *A39*, 876-881.
[2] Flack, H. D.; Bernardinelli, G. Reporting and evaluating absolute-structure and absolute-configuration determinations. *J. Appl. Cryst.* **2000**, *33*, 1143-1148.
[3] Thompson, A. L.; Watkin, D. J. *CRYSTALS* enhancements: absolute structure determination. *J. Appl. Cryst.* **2011**, *44*, 1017-1022
[4] Steiner, S.; Magno, A.; Huang, D.; Caflisch A. Does bromodomain flexibility influence histone recognition? *FEBS Lett*. **2013**, *587*, 2158-2163.
[5] Gallivan, J.P.; Dougherty, D.A. Cation-π interactions in structural biology. *Proc. Natl. Acad. Sci. U.S.A*. **1999**, *96*, 9459-9464.
[6] Khandelia H.; Kaznessis, Y. N. Cation-pi interactions stabilize the structure of the antimicrobial peptide indolicidin near membranes: molecular dynamics simulations. *J Phys Chem B.* **2007**, *111*, 242-50.
[7] Frisch, M. J.; Trucks, G. W.; Schlegel, H. B.; Scuseria, G. E.; Robb, M. A.; Cheeseman, J. R.; Scalmani, G.; Barone, V.; Mennucci, B.; Petersson, G. A.; Nakatsuji, H.; Caricato, M.; Li, X.; Hratchian, H. P.; Izmaylov, A. F.; Bloino, J.; Zheng, G.; Sonnenberg, J. L.; Hada, M.; Ehara, M.; Toyota, K.; Fukuda, R.; Hasegawa, J.; Ishida, M.; Nakajima, T.; Honda, Y.; Kitao, O.; Nakai, H.; Vreven, T.; Montgomery Jr., J. A.; Peralta, J. E.; Ogliaro, F.; Bearpark, M.; Heyd, J. J.; Brothers, E.; Kudin, K. N.; Staroverov, V. N.; Kobayashi, R.; Normand, J.; Raghavachari, K.; Rendell, A.; Burant, J. C.; Iyengar, S. S.; Tomasi, J.; Cossi, M.; Rega, N.; Millam, J. M.; Klene, M.; Knox, J. E.; Cross, J. B.; Bakken, V.; Adamo, C.; Jaramillo, J.; Gomperts, R.; Stratmann, R. E.; Yazyev, O.; Austin, A. J.; Cammi, R.; Pomelli, C.; Ochterski, J. W.; Martin, R. L.; Morokuma, K.; Zakrzewski, V. G.; Voth, G. A.; Salvador, P.; Dannenberg, J. J.; Dapprich, S.; Daniels, A. D.; Farkas, Ö.; Foresman, J. B.; Ortiz, J. V.; Cioslowski, J.; Fox. D. J. *Gaussian 09, Revision A.1*; Gaussian, Inc., Wallingford, CT, **2009**.
[8] Barone V.; Cossi, M. Quantum Calculation of Molecular Energies and Energy Gradients in Solution by a Conductor Solvent Model. *J. Phys. Chem. A,* **1998**, *102*, 1995; Cossi, M.; Rega, N.; Scalmani, G.; Barone, V. Energies, structures, and electronic properties of molecules in solution with the C-PCM solvation model. *J. Comp. Chem.* **2003**, *24*, 669.
[9] Chai, J.-D.; Head-Gordon, M. Long-range corrected hybrid density functionals with damped atom-atom dispersion corrections. *Phys. Chem. Chem. Phys*. **2008**, *10*, 6615.
[10] Schaefer, A.; Huber, C.; Ahlrichs, R. Fully optimized contracted Gaussian basis sets of triple zeta valence quality for atoms Li to Kr. *J. Chem. Phys.* **1994**, *100*, 5829.
[11] Philpott, M.; Yang, J.; Tumber, T.; Fedorov, O.; Uttarkar, S.; Filippakopoulos, P.; Picaud, S.; Keates, T.; Felletar, I.; Ciulli, A.; Knapp, S.; Heightman, T. D. Bromodomain-peptide displacement assays for interactome mapping and inhibitor discovery. *Mol. BioSyst.* **2011**, *7*, 2899-2908.
[12] Hopkins, A. L.; Groom, C. R.; Alex, A.; Ligand efficiency: a useful metric for lead selection. *Drug Discov. Today* **2004**, *9*, 430-431.
[13] Vanquelef, E.; Simon, S.; Marquant, G.; Garcia, E; Klimerak, G.; Delepine, J.C.; Cieplak, P.; Dupradeau, F.Y. R.E.D. Server: a web service for deriving RESP and ESP charges and building force field libraries for new molecules and molecular fragments. *Nucl. Acids Res*. **2011**, *39*, W511-W517.
[14] Duan, Y.; Wu, C.; Chowdhury, S.; Lee, M.C.; Xiong, G.; Zhang, W.; Yang, R.; Cieplak, P.; Luo, R.; Lee, T.; Caldwell, J.; Wang, J.; Kollman, P. A Point-Charge Force Field for Molecular Mechanics Simulations of Proteins Based on Condensed-Phase Quantum Mechanical Calculations. *J. Comp. Chem*. **2003**, *24*, 1999-2012.
[15] Dupradeau, F.Y.; Pigache, A.; Zaffran, T.; Savineau, C.; Lelong, R.; Grivel, N.; Lelong, D.; Rosanski, W.; Cieplak, P. The R.E.D. Tools: Advances in RESP and ESP charge derivation and force field library building. *Phys. Chem. Chem. Phys*. **2010**, *12*, 7821–7839.
[16] Trott, O.; Olson, A.J. AutoDock Vina: improving the speed and accuracy of docking with a new scoring function, efficient optimization and multithreading. *J. Comput. Chem*. **2010**, *31*, 455-461.
[17] Leach, A.R.; Shoichet, B.K.; Peishoff, C.E. Prediction of protein-ligand interactions. Docking and scoring: successes and gaps. *J. Med. Chem.* **2006**, *49*, 5851–5855; (b) Kontoyianni, M.; McClellan, L.M., Sokol G.S. Evaluation of docking performance: comparative data on docking algorithms. *J. Med. Chem*. **2004**, *47*, 558–565; (c) Warren, G.L.; Andrews, C.W.; Capelli, A.M.; Clarke, B.; LaLonde, J.; Lambert, M.H.; Lindvall, M.; Nevins, N.; Semus, S.F.; Senger, S.; Tedesco, G.; Wall, I.D.; Woolven J.M.; Peishoff C.E.; Head M.S. A critical assessment of docking programs and scoring functions. *J. Med. Chem*. **2006**, *49*, 5912–5931.
[18] (a) Wang, J.; Wang, W.; Kollman, P.A.; Case, D.A. Automatic atom type and bond type perception in molecular mechanical calculations. *J. Mol. Graphics Modell*. **2006**, *25*, 247260; (b) Wang, J.; Wolf, R.M.; Caldwell, J.W.; Kollman, P.A.; Case, D.A. Development and testing of a general AMBER force field. *J. Comput. Chem*. **2004**, *25*, 1157-1174.
[19] Chen, V.B.; Arendall, W.B.; Headd, J.J.; Keedy, D.A.; Immormino, R.M.; Kapral, G.J.; Murray, L.W.; Richardson, J.S.; Richardson, D.C. MolProbity: all-atom structure validation for macromolecular crystallography. *Acta Crystallogr*. **2010**, *D66*:12-21.
[20] (a) Case, D. A.; Darden, T. A.; Cheatham, T. E.; Simmerling, C. L.; Wang, J.; Duke R. E.; Luo, R.; Walker, R. C.; Zhang, W.; Merz, K. M.; Roberts, B.; Hayik, S.; Roitberg, A.; Seabra, G.; Swails, J.; Goetz, A. W.; Kolossváry, I.; Wong, K. F.; Paesani, F.; Vanicek, J.; Wolf, R. M.; Liu, J.; Wu, X.; Brozell, S. R.; Steinbrecher, T.; Gohlke, H.; Cai, Q.; Ye, X.; Wang, J.; Hsieh, M. J.; Cui, G.; Roe, D. R.; Mathews, D. H.; Seetin, M. G.; Salomon-Ferrer, R.; Sagui, C.; Babin, V.; Luchko, T.; Gusarov, S.; Kovalenko, A.; Kollman, P. A. AMBER 12. **2012**. University of California, San Francisco.; (b) Salomon-Ferrer, R.; Götz, A. W.; Poole, D.; Le Grand, S.; Walker, R. C. Routine Microsecond Molecular Dynamics Simulations with AMBER on GPUs. 2. Explicit Solvent Particle Mesh Ewald. *J. Chem. Theory Comput.* **2013***, 9*, 3878-3888; (c) Götz, A. W., Williamson; M. J.; Xu, D.; Poole, D.; Le

Grand, S.; Walker, R. C. Routine Microsecond Molecular Dynamics Simulations with AMBER on GPUs. 1. Generalized Born. *J. Chem. Theory Comput.* **2012**, *8*, 1542-1555.

[21] Jorgensen, W.L.; Madura, J.D. Solvation and conformation of methanol in water. *J. Am. Chem. Soc.* **1983**, *105*, 1407–1413.

[22] Berendsen, H.J.C.; Postma, J.P.M.; van Gunsteren, W.F.; DiNola, A.; Haak, J.R. Molecular-dynamics with coupling to an external bath. *J. Chem. Phys*. **1984**, *81*, 3684–3690.

[23] Kräutler, V.; van Gunsteren, W.F.; Hünenberger, P.H. A Fast SHAKE Algorithm to solve distance constraint equations for small molecules in molecular dynamics simulations. *J. Comput. Chem*. **2001**, *22*, 501-508.

[24] Humphrey, W.; Dalke, A.; Schulten, K.VMD: visual molecular dynamics. *J. Mol. Graph*. **2006**, *14*, 33–38.

[25] C, N, O atoms were restrained at their crystallographic coordinates, while the positions of the added H atoms were optimized at the B3LYP/6-31G(d,p) level of theory.

[26] (a) Becke, A.D. Density-functional thermochemistry. III. The role of exact exchange. *J. Chem. Phys.* **1993**, *98*, 5648-52. (b) Lee, C.; Yang, W.; Parr, R. G. Development of the Colle-Salvetti correlation-energy formula into a functional of the electron density. *Phys. Rev. B*. **1988**, *37*, 785-89.

[27] Marenich, A.V.; Cramer, C.J.; Truhlar, D.G. Universal solvation model based on solute electron density and a continuum model of the solvent defined by the bulk dielectric constant and atomic surface tensions. *J. Phys. Chem. B*. **2009**, *113*, 6378-96.

[28] (a) Grimme, S.; Antony, J.; Ehrlich, S.; Krieg, H. A consistent and accurate ab initio parameterization of density functional dispersion correction (DFT-D) for the 94 elements H-Pu. *J. Chem. Phys*. **2010**, *132*, 154104. (b) Grimme, S.; Ehrlich, S.; Goerigk, L. The BJ-damping used in version 4. *J. Comput. Chem.* **2011**, *32*, 1456-1465.

[29] Zhao, Y.; Truhlar, D.G. The M06 suite of density functionals for main group thermochemistry, thermochemical kinetics, noncovalent interactions, excited states, and transition elements: two new functionals and systematic testing of four M06-class functionals and 12 other functionals. *Theor. Chem. Acc.* **2008**, *120*, 215-41.

[30] Cosier, J.; Glazer, A. M. A nitrogen-gas-stream cryostat for general X-ray diffraction studies. *J. Appl. Cryst.* **1986**, *19*, 105-107.

[31] Altomare, A.; Cascarano, G.; Giacovazzo, C.; Guagliardi, A.; Burla, M. C.; Polidori, G.; Camalli, M. *SIR*92 - a program for automatic solution of crystal structures by direct methods. *J. Appl. Cryst.* **1994**, *27*, 435.

[32] Betteridge, P. W.; Carruthers, J. R.; Cooper, R. I.; Prout, K.; Watkin, D. J. *CRYSTALS* version 12: software for guided crystal structure analysis. *J. Appl. Cryst.* **2003**, *36*, 1487.

[33] Cooper, R. I.; Thompson, A. L.; Watkin, D. J. *CRYSTALS* enhancements: dealing with hydrogen atoms in refinement. *J. Appl. Cryst.* **2010**, *43*, 1100-1107.

[34] Hewings, D. S.; Wang, M.; Philpott, M.; Fedorov, O.; Uttarkar, S.; Filippakopoulos, P.; Picaud, S.; Vuppusetty, C.; Marsden, B.; Knapp, S.; Conway, S. J.; Heightman, T. D. 3,5-Dimethylisoxazoles Act As Acetyl-lysine-mimetic Bromodomain Ligands. *J. Med. Chem.* **2011**, *54*, 6761-6770.

[35] Wiseman, T.; Williston, S.; Brandts, J. F.; Lin L. N. Rapid measurement of binding constants and heats of binding using a new titration calorimeter. *Anal. Biochem.* **1989**, *179*, 131-137.

[36] Filippakopoulos, P.; Picaud, S.; Mangos, M.; Keates, T.; Lambert, J. P.; Barsyte-Lovejoy, D.; Felletar, I.; Volkmer, R.; Muller, S.; Pawson, T.; Gingras, A. C.; Arrowsmith, C. H.; Knapp, S. Histone recognition and large-scale structural analysis of the human bromodomain family. *Cell* **2012,** *149*, 214-31.

[37] Kabsch, W. Evaluation of Single-Crystal X-Ray-Diffraction Data from a Position-Sensitive Detector. *J. Appl. Cryst.* **1988,** *21*, 916-924.

[38] Kabsch, W. Automatic-Indexing of Rotation Diffraction Patterns. *J Appl Cryst* **1988,** *21*, 67-71.

[39] Evans, P. *SCALA - scale together multiple observations of reflections*, 3.3.0; MRC Laboratory of Molecular Biology: Cambridge, **2007**.

[40] McCoy, A. J.; Grosse-Kunstleve, R. W.; Storoni, L. C.; Read, R. J. Likelihood-enhanced fast translation functions. *Acta. Crystallogr. D. Biol. Crystallogr.* **2005,** *61*, 458-464.

[41] Perrakis, A.; Morris, R.; Lamzin, V. S. Automated protein model building combined with iterative structure refinement. *Nat. Struct. Biol.* **1999,** *6*, 458-63.

[42] Emsley, P.; Cowtan, K. Coot: model-building tools for molecular graphics. *Acta. Crystallogr. D. Biol. Crystallogr.* **2004,** *60*, 2126-32.

[43] Murshudov, G. N.; Vagin, A. A.; Dodson, E. J. Refinement of macromolecular structures by the maximum-likelihood method. *Acta. Crystallogr. D. Biol. Crystallogr.* **1997,** *53*, 240-255.

[44] Painter, J.; Merritt, E. A. Optimal description of a protein structure in terms of multiple groups undergoing TLS motion. *Acta. Crystallogr. D. Biol. Crystallogr.* **2006,** *62*, 439-50.

[45] Fedorov, O.; Lingard, H.; Wells, C.; Monteiro, O. P.; Picaud, S.; Keates, T.; Yapp, C.; Philpott, M.; Martin, S. J.; Felletar, I.; Marsden, B. D.; Filippakopoulos, P.; Muller, S.; Knapp, S.; Brennan, P. E. [1,2,4]Triazolo[4,3-*a*]phthalazines: Inhibitors of Diverse Bromodomains. *J. Med. Chem.* **2014**, *54*, dx.doi.org/10.1021/jm401568s.

[46] Pangborn, A. B.; Giardello, M. A.; Grubbs, R. H.; Rosen, R. K.; Timmers, F. J. Safe and Convenient Procedure for Solvent Purification. *Organomet.*, **1996**, *15*, 1518-1520.

[47] Dai, W.-M.; Wang, X.; Ma C. Microwave-assisted one-pot regioselective synthesis of 2-alkyl-3,4-dihydro-3-oxo-2H-1,4-benzoxazines. *Tetrahedron* **2005**, *61*, 6879-6885.

[48] Tsuritani, T.; Yamamoto, Y.; Kawasaki, M.; Mase T. Novel Approach to 3,4-Dihydro-2(^{1}H)-quinolinone Derivatives via Cyclopropane Ring Expansion. *Org. Lett.* **2009**, *11*, 1043–1045.

[49] Pauff S. M.; Miller S. C.; Synthesis of Near-IR Fluorescent Oxazine Dyes with Esterase-Labile Sulfonate Esters. *Org. Lett.* **2011**, *13*, 6196–6199.

Benzyl 2-fluoro-3-nitrobenzoate **12** ^{1}H NMR

```
NAME                tr4066
EXPNO                    1
PROCNO                   1
Date_             20130405
Time                 16.53
INSTRUM             avc500
PROBHD   5 mm CPDUL 13C
PULPROG               zg30
TD                   65536
SOLVENT              CDCl3
NS                      16
DS                       4
SWH              10330.578 Hz
FIDRES            0.157632 Hz
AQ               3.1719923 sec
RG                     2.8
DW                  48.400 usec
DE                    6.00 usec
TE                   298.0 K
D1              1.00000000 sec
TD0                      1

======== CHANNEL f1 ========
NUC1                    1H
P1                   10.60 usec
PL1                   6.00 dB
PL1W            0.95905519 W
SFO1           500.3030896 MHz
SI                   32768
SF             500.3000240 MHz
WDW                     EM
SSB                      0
LB                    0.30 Hz
GB                       0
PC                    1.00
```

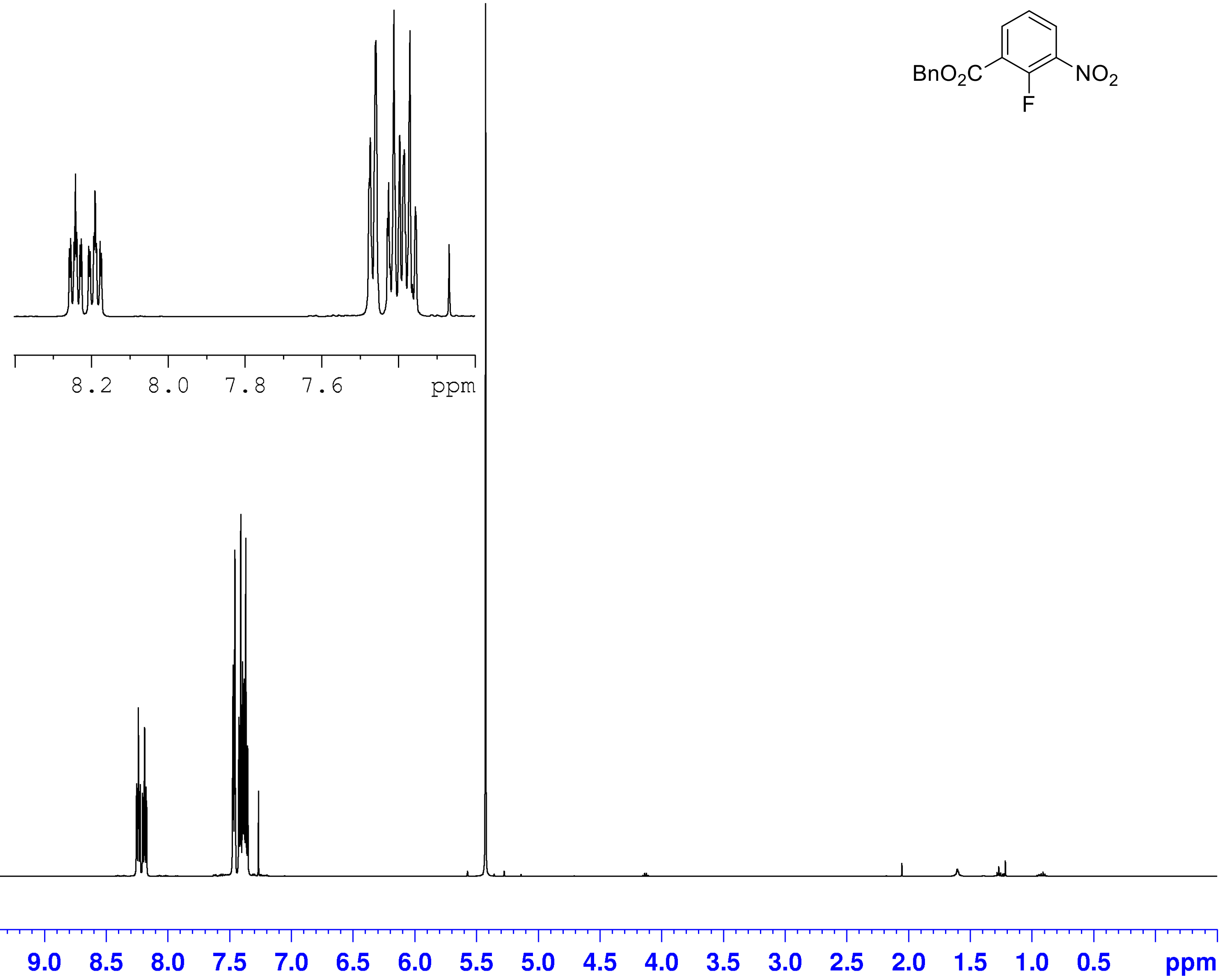

Benzyl 2-fluoro-3-nitrobenzoate **12** ^{13}C NMR

NAME	tr4066
EXPNO	3
PROCNO	1
Date_	20130405
Time	17.18
INSTRUM	avc500
PROBHD	5 mm CPDUL 13C
PULPROG	zgpg30
TD	65536
SOLVENT	CDCl3
NS	256
DS	2
SWH	31250.000 Hz
FIDRES	0.476837 Hz
AQ	1.0486259 sec
RG	912
DW	16.000 usec
DE	20.00 usec
TE	298.0 K
D1	2.00000000 sec
D11	0.03000000 sec
TD0	1

======== CHANNEL f1 ========

NUC1	13C
P1	10.25 usec
PL1	8.00 dB
PL1W	1.62029624 W
SFO1	125.8131151 MHz

======== CHANNEL f2 ========

CPDPRG2	waltz16
NUC2	1H
PCPD2	80.00 usec
PL2	6.00 dB
PL12	23.56 dB
PL13	29.56 dB
PL2W	0.95905519 W
PL12W	0.01682068 W
PL13W	0.00422516 W
SFO2	500.3020012 MHz
SI	32768
SF	125.8005438 MHz
WDW	EM
SSB	0
LB	1.00 Hz
GB	0
PC	1.40

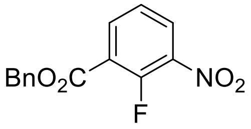

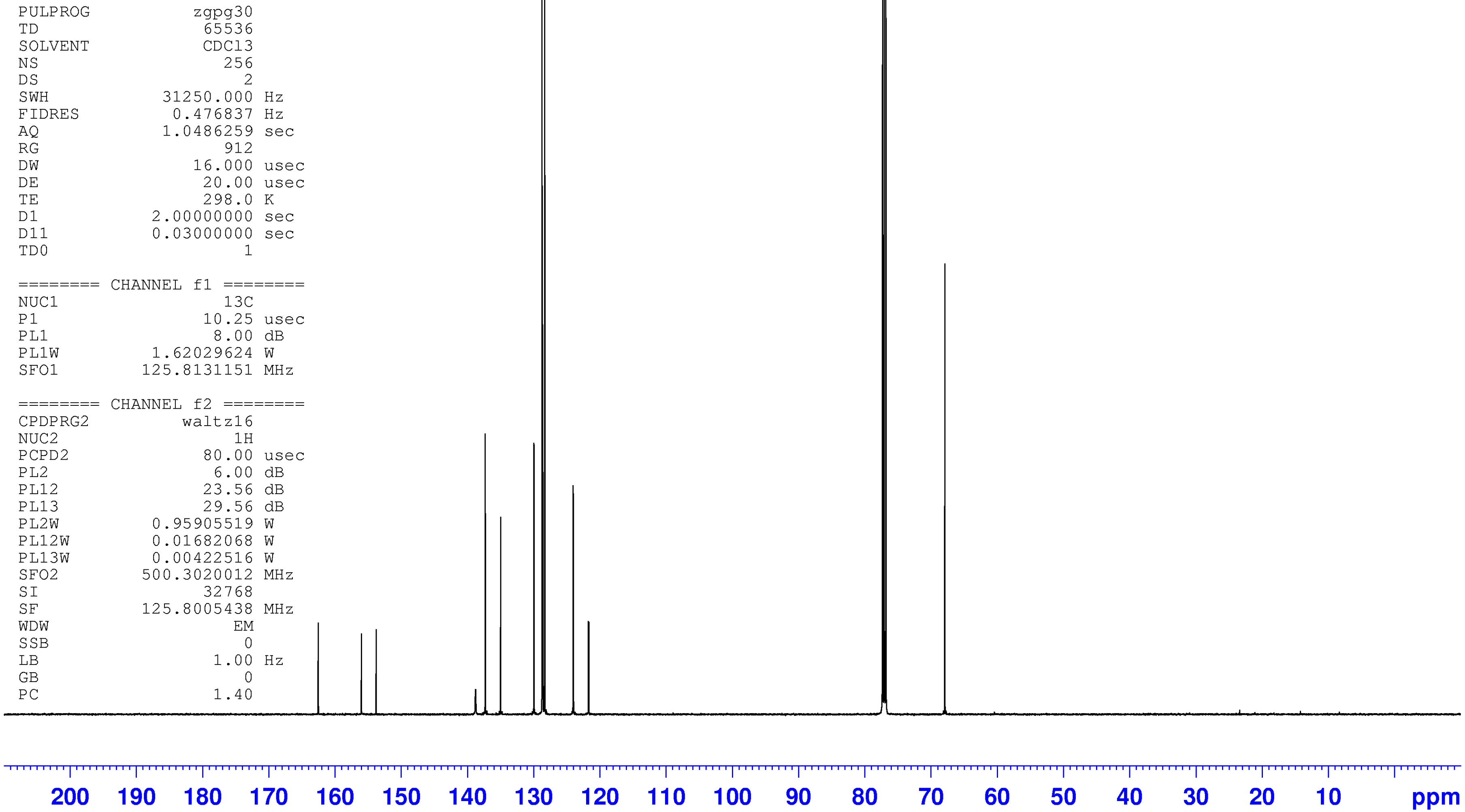

Benzyl 2-fluoro-3-nitrobenzoate **12** ^{19}F NMR

```
NAME       FULL-tr4066-extra
EXPNO                     25
PROCNO                     1
Date_               20130404
Time                   10.17
INSTRUM               avn400
PROBHD     5 mm PABBO BB/
PULPROG               zgflqn
TD                    131072
SOLVENT                CDCl3
NS                        16
DS                         4
SWH                75000.000 Hz
FIDRES              0.572205 Hz
AQ                 0.8738633 sec
RG                    205.43
DW                     6.667 usec
DE                      6.50 usec
TE                     296.0 K
D1                1.00000000 sec
TD0                        1

======== CHANNEL f1 ========
SFO1             376.5547873 MHz
NUC1                     19F
P1                     13.50 usec
SI                     65536
SF               376.6112790 MHz
WDW                       EM
SSB                        0
LB                      0.30 Hz
GB                         0
PC                      1.00
```

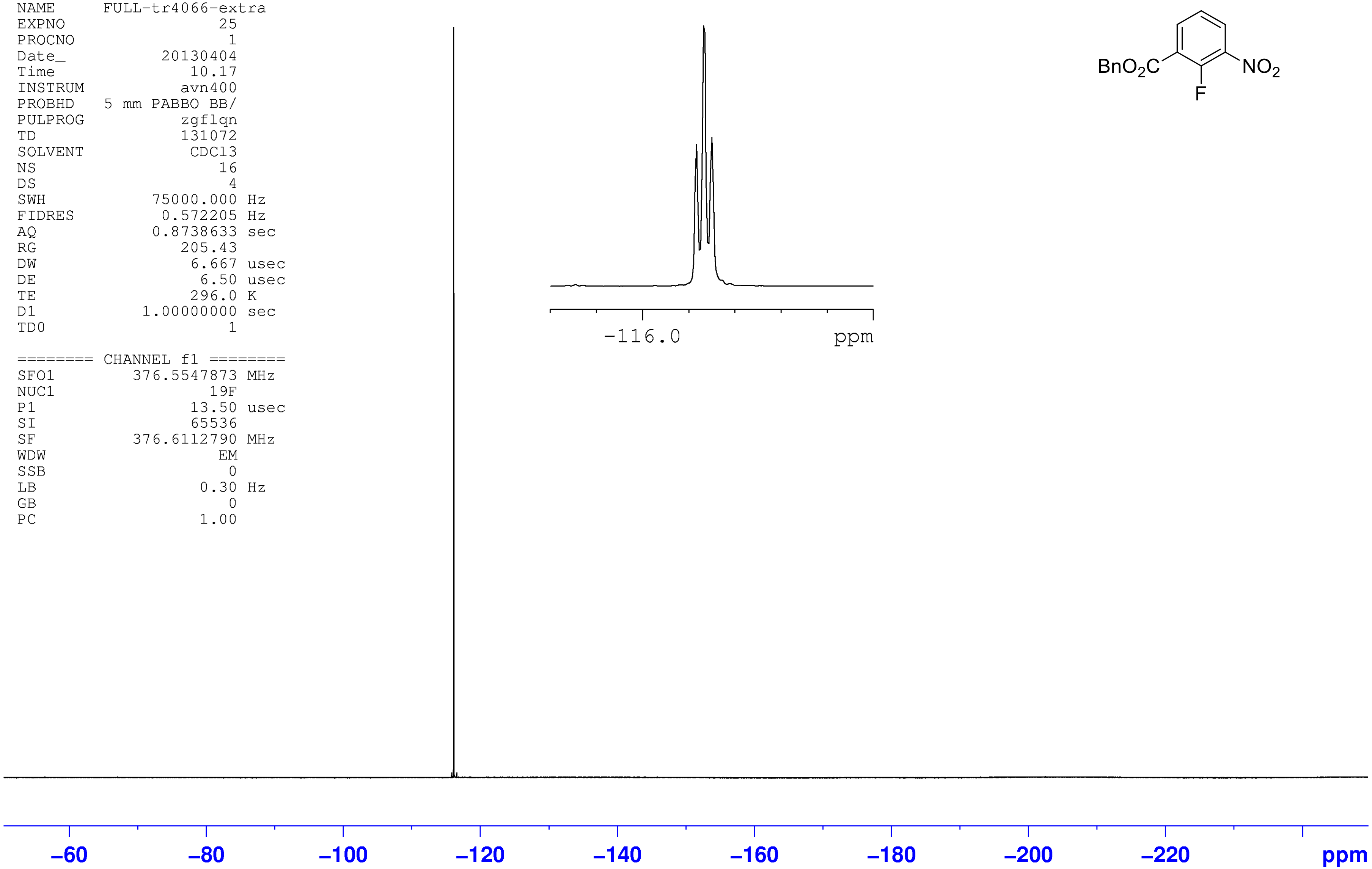

Benzyl 2-{[(2’*R*)-1’-methoxy-1’-oxopropan-2’-yl]amino}-3-nitrobenzoate (*R*)-**13** ^{1}H NMR

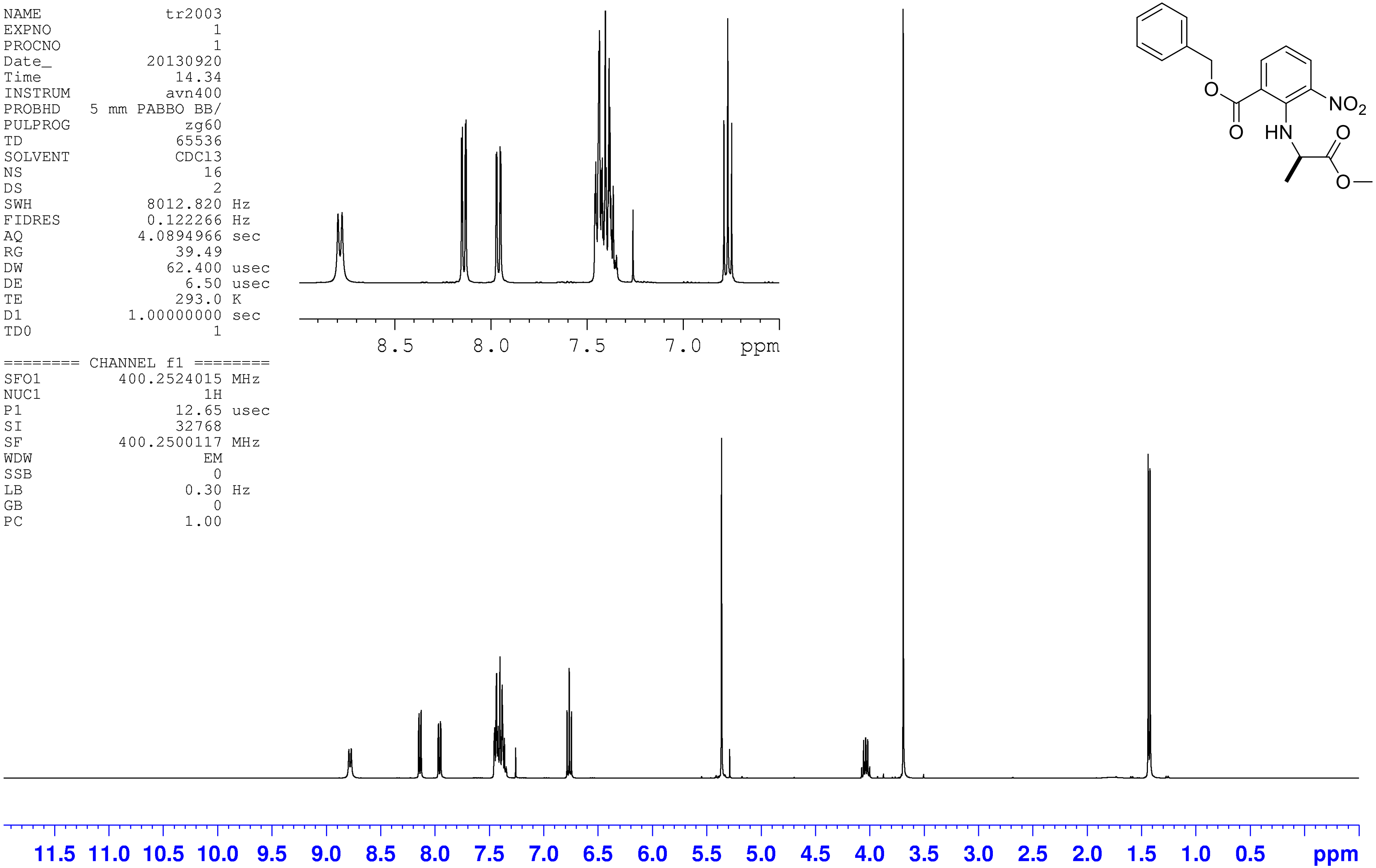

Benzyl 2-{[(2'*R*)-1'-methoxy-1'-oxopropan-2'-yl]amino}-3-nitrobenzoate (*R*)-**13** ^{13}C NMR

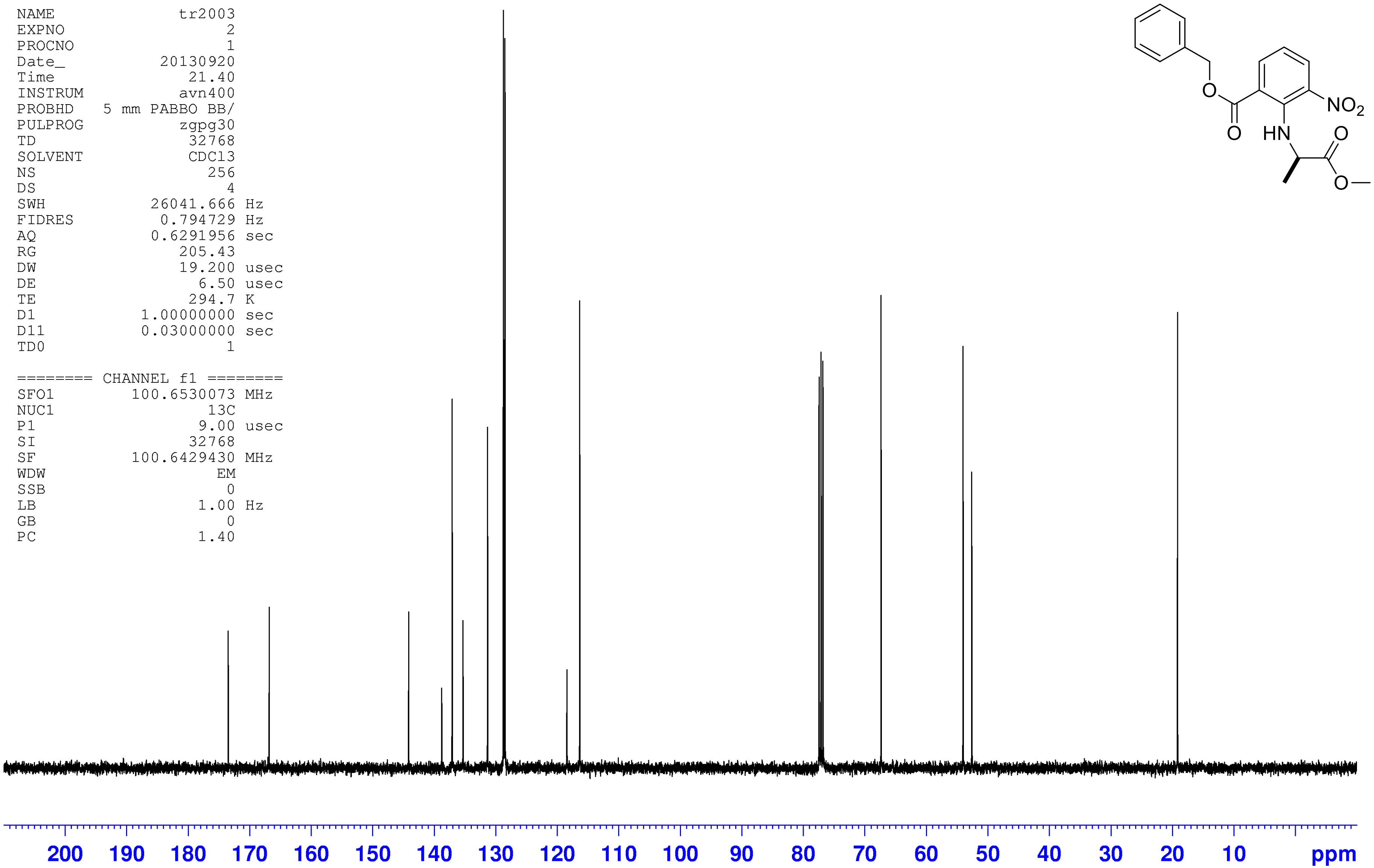

Benzyl (3*R*)-3-methyl-2-oxo-1,2,3,4-tetrahydroquinoxaline-5-carboxylate (*R*)-**14** ^{1}H NMR

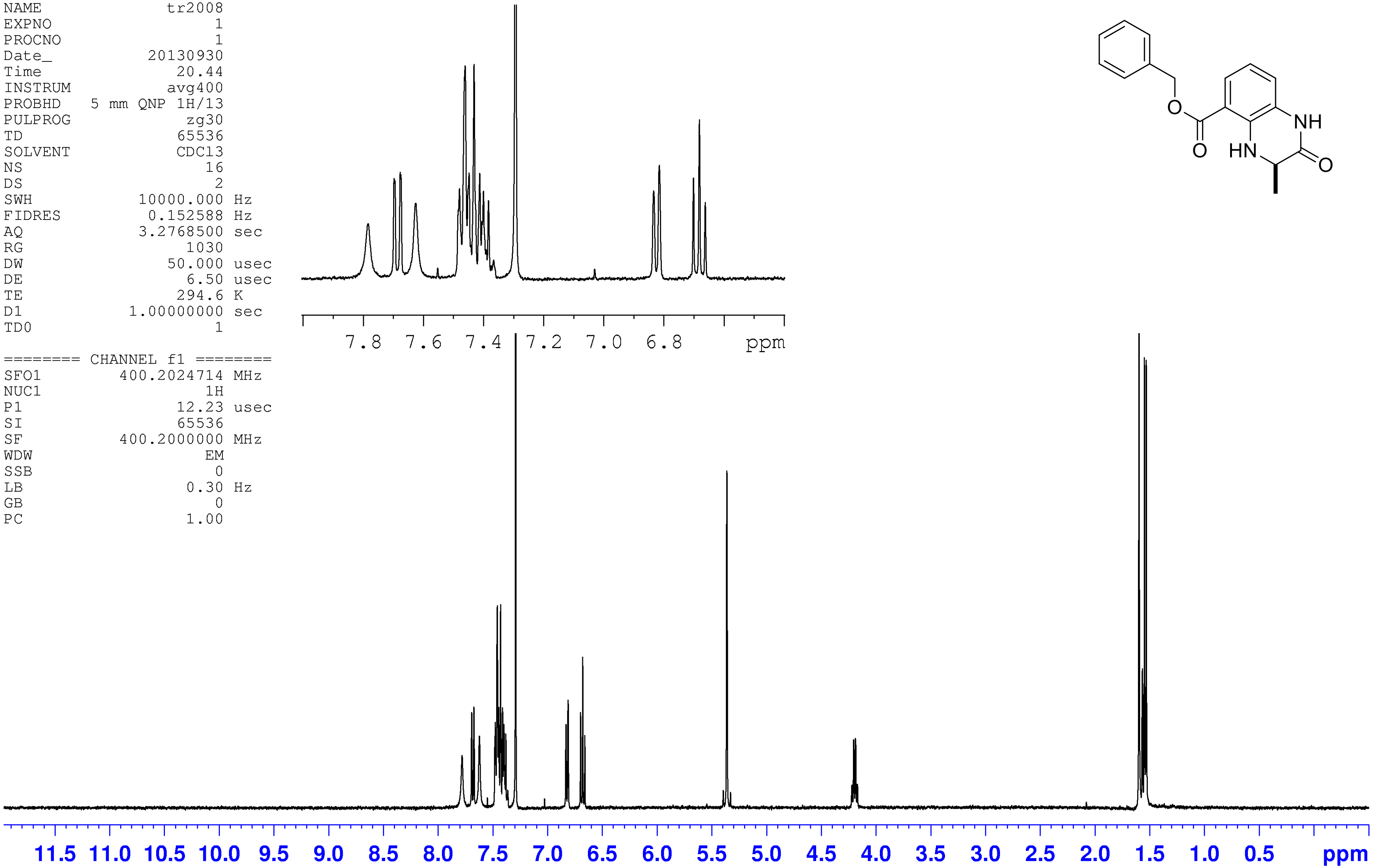

Benzyl (3*R*)-3-methyl-2-oxo-1,2,3,4-tetrahydroquinoxaline-5-carboxylate (*R*)-**14** ^{13}C NMR

```
NAME              tr2008
EXPNO                  4
PROCNO                 1
Date_           20120130
Time               14.57
INSTRUM           avc500
PROBHD   5 mm CPDUL 13C
PULPROG          zgpg30
TD                 65536
SOLVENT            CDCl3
NS                   485
DS                     2
SWH            31250.000 Hz
FIDRES          0.476837 Hz
AQ             1.0486259 sec
RG                  1820
DW                16.000 usec
DE                 20.00 usec
TE                 298.0 K
D1            2.00000000 sec
D11           0.03000000 sec
TD0                    1

======== CHANNEL f1 ========
NUC1                 13C
P1                 10.00 usec
PL1                -4.40 dB
PL1W         28.15752029 W
SFO1         125.8131151 MHz

======== CHANNEL f2 ========
CPDPRG2          waltz16
NUC2                  1H
PCPD2              80.00 usec
PL2                -6.00 dB
PL12               12.42 dB
PL13               18.42 dB
PL2W         15.19999981 W
PL12W         0.21869738 W
PL13W         0.05493430 W
SFO2         500.3020012 MHz
SI                 32768
SF           125.8005438 MHz
WDW                   EM
SSB                    0
LB                  1.00 Hz
GB                     0
PC                  1.40
```

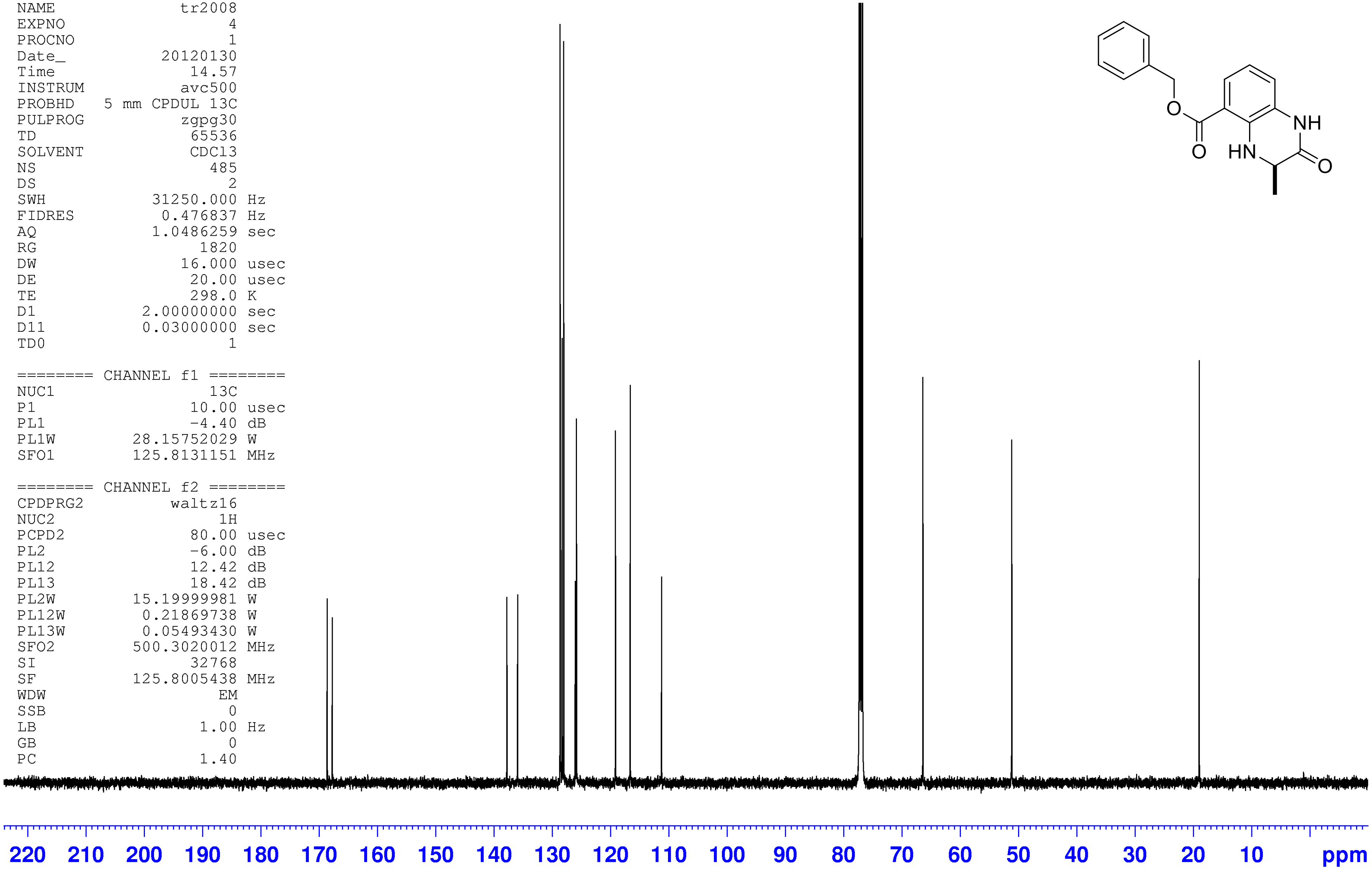

(*R*)-3-Methyl-2-oxo-1,2,3,4-tetrahydroquinoxaline-5-carboxylic acid (*R*)-**15** ^{1}H NMR

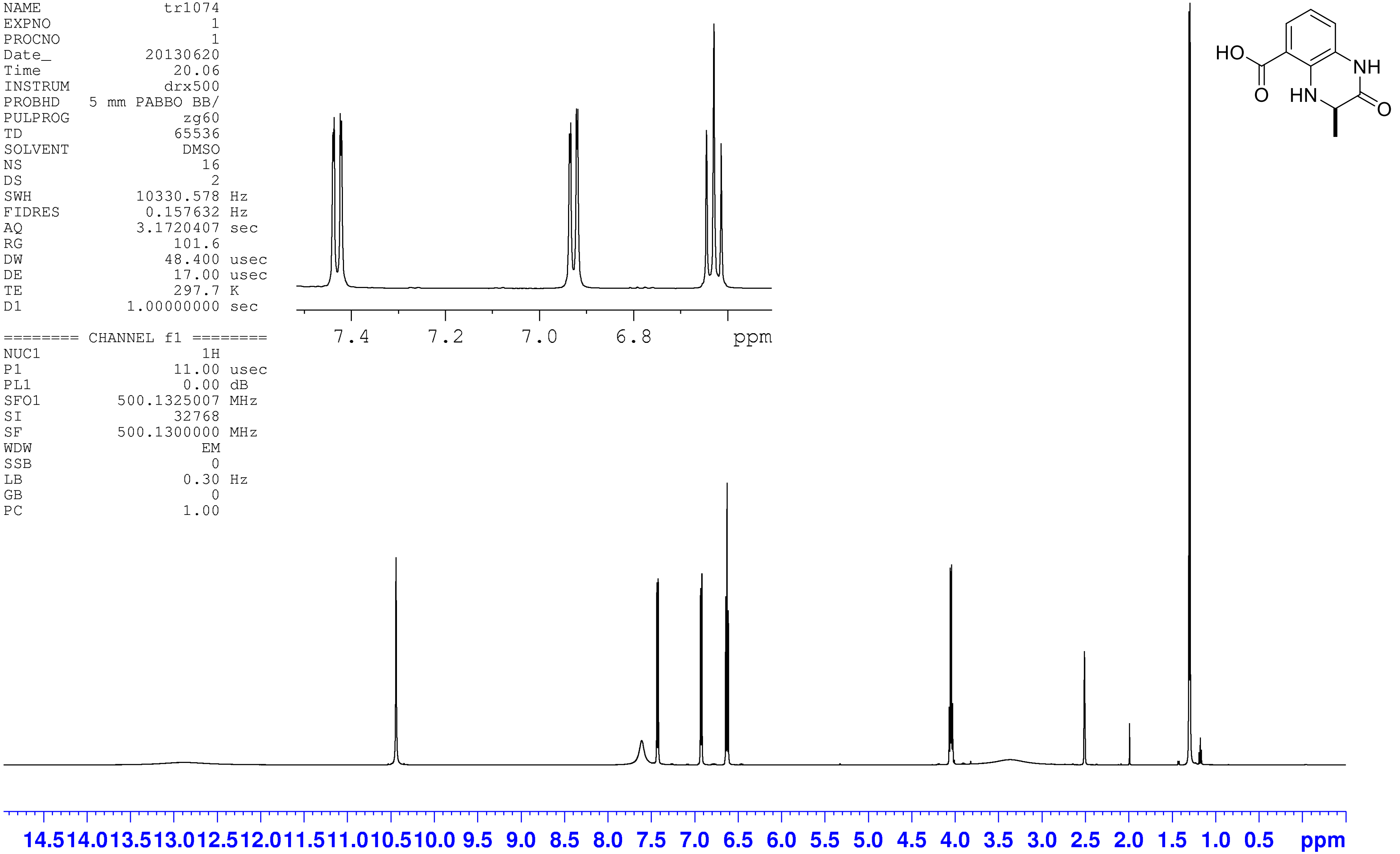

(*R*)-3-Methyl-2-oxo-1,2,3,4-tetrahydroquinoxaline-5-carboxylic acid (*R*)-**15** ^{13}C NMR

```
NAME             tr1074
EXPNO                 2
PROCNO                1
Date_          20130620
Time              21.55
INSTRUM          drx500
PROBHD   5 mm PABBO BB/
PULPROG         zgpg30
TD                65536
SOLVENT            DMSO
NS                 2048
DS                    4
SWH           30303.031 Hz
FIDRES         0.462388 Hz
AQ            1.0814105 sec
RG               9195.2
DW               16.500 usec
DE                11.00 usec
TE                297.7 K
D1           2.00000000 sec
d11          0.03000000 sec
DELTA        1.89999998 sec
TD0                   1

======== CHANNEL f1 ========
NUC1                13C
P1                 6.80 usec
PL1                5.00 dB
SFO1        125.7703148 MHz

======== CHANNEL f2 ========
CPDPRG2         waltz16
NUC2                 1H
PCPD2            100.00 usec
PL2                0.00 dB
PL12              19.00 dB
PL13              23.00 dB
SFO2        500.1320005 MHz
SI                32768
SF          125.7578019 MHz
WDW                  EM
SSB                   0
LB                 1.00 Hz
GB                    0
PC                 1.40
```

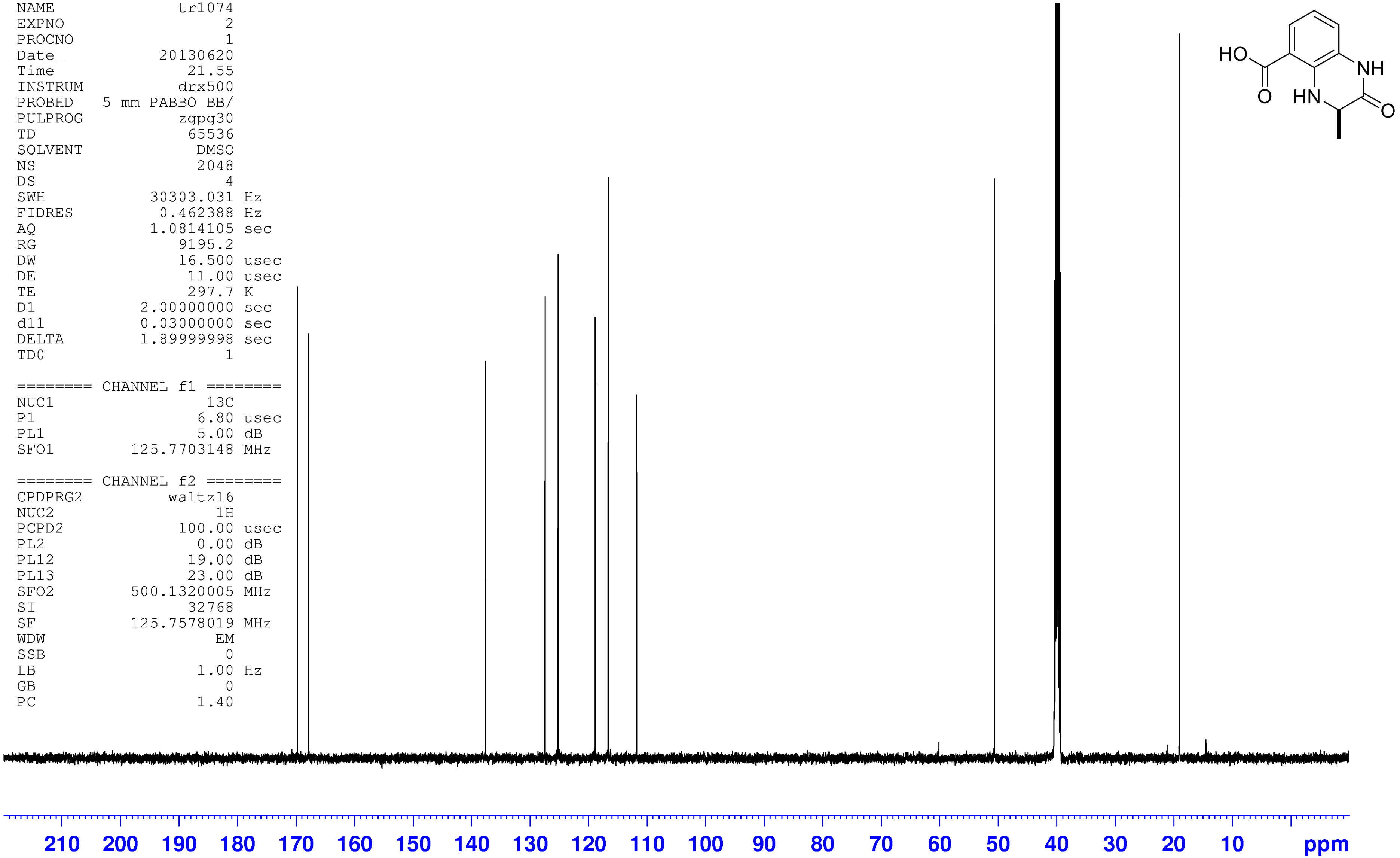

Benzyl 2,6-difluoro-3-nitrobenzoate **17** ^{1}H NMR

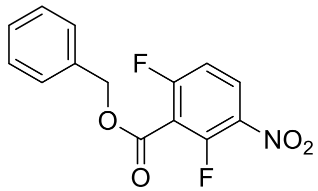


```
NAME              tr4014
EXPNO                  1
PROCNO                 1
Date_           20121122
Time               18.01
INSTRUM            av400
PROBHD    5 mm QNP 1H/13
PULPROG             zg60
TD                 65536
SOLVENT            CDCl3
NS                    16
DS                     2
SWH             8278.146 Hz
FIDRES          0.126314 Hz
AQ             3.9584243 sec
RG                 645.1
DW                60.400 usec
DE                  7.50 usec
TE                 300.0 K
D1            1.00000000 sec

======== CHANNEL f1 ========
NUC1                  1H
P1                 10.10 usec
PL1                 0.00 dB
SFO1         400.2024714 MHz
SI                 32768
SF           400.2000028 MHz
WDW                   EM
SSB                    0
LB                  0.30 Hz
GB                     0
PC                  1.00
```

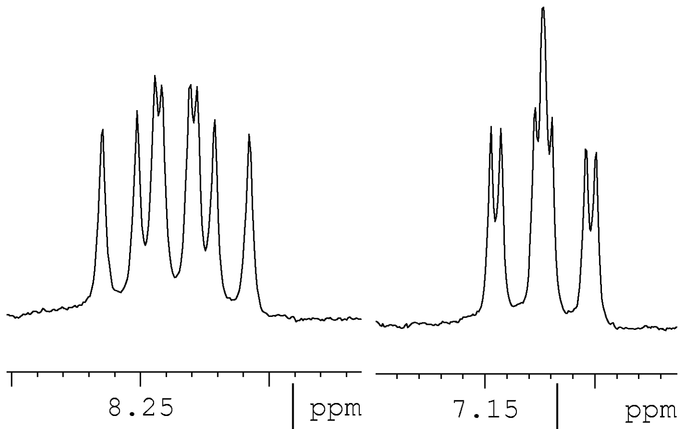
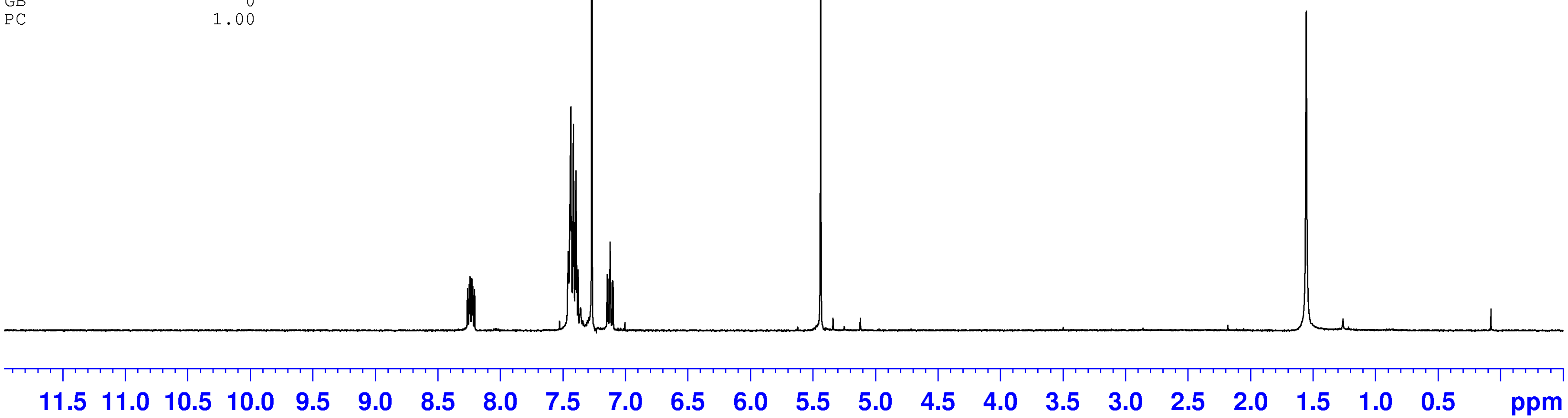

Benzyl 2,6-difluoro-3-nitrobenzoate **17** ^{13}C NMR

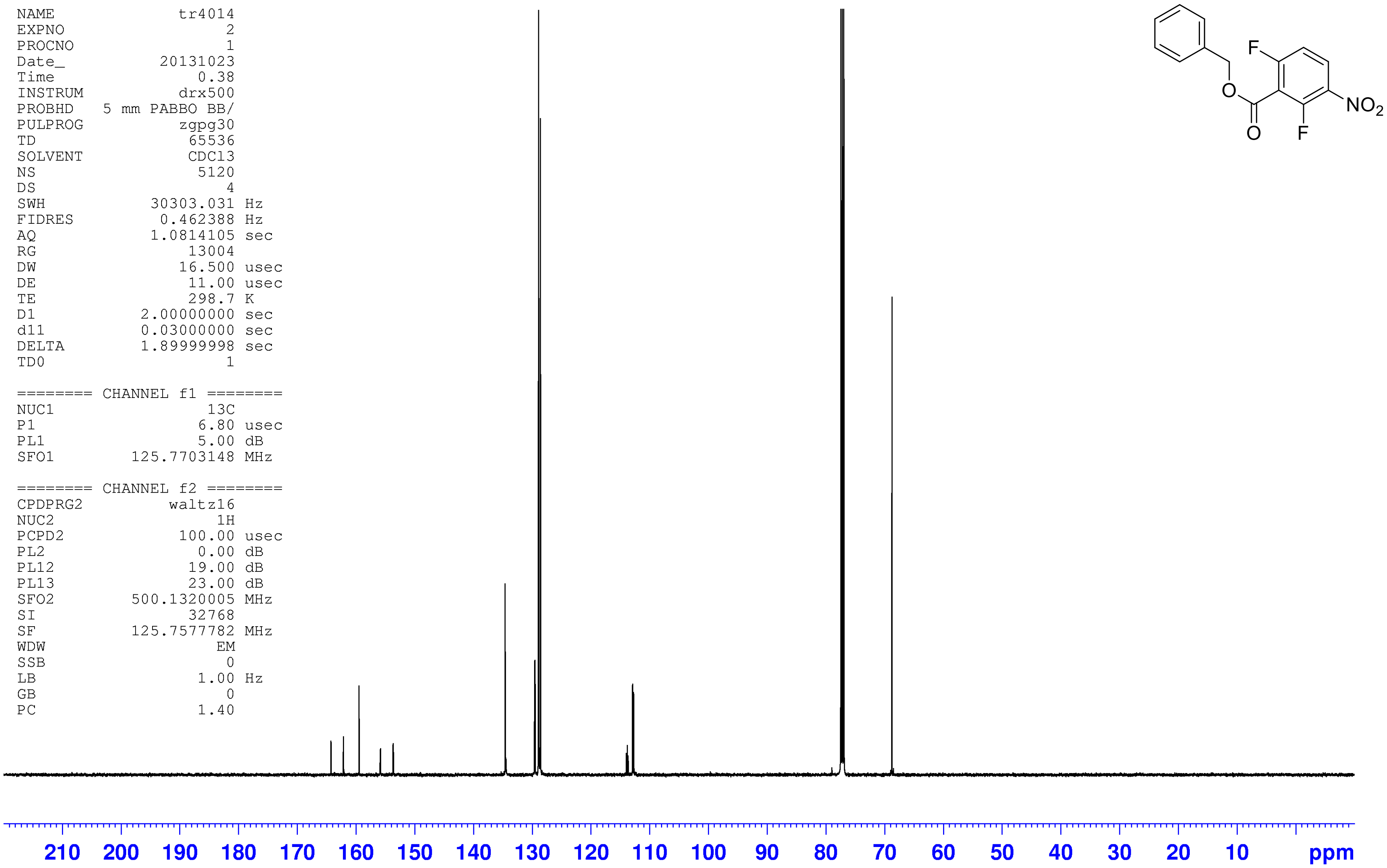

Benzyl 2,6-difluoro-3-nitrobenzoate **17** ^{19}F NMR

NAME	tr4014
EXPNO	3
PROCNO	1
Date_	20121118
Time	22.41
INSTRUM	avn400
PROBHD	5 mm PABBO BB/
PULPROG	zgflqn
TD	131072
SOLVENT	CDCl3
NS	256
DS	4
SWH	75000.000 Hz
FIDRES	0.572205 Hz
AQ	0.8738633 sec
RG	205.43
DW	6.667 usec
DE	6.50 usec
TE	293.8 K
D1	1.00000000 sec
TD0	1

======== CHANNEL f1 ========

SFO1	376.5547873 MHz
NUC1	19F
P1	13.50 usec
SI	65536
SF	376.6112790 MHz
WDW	EM
SSB	0
LB	0.30 Hz
GB	0
PC	1.00

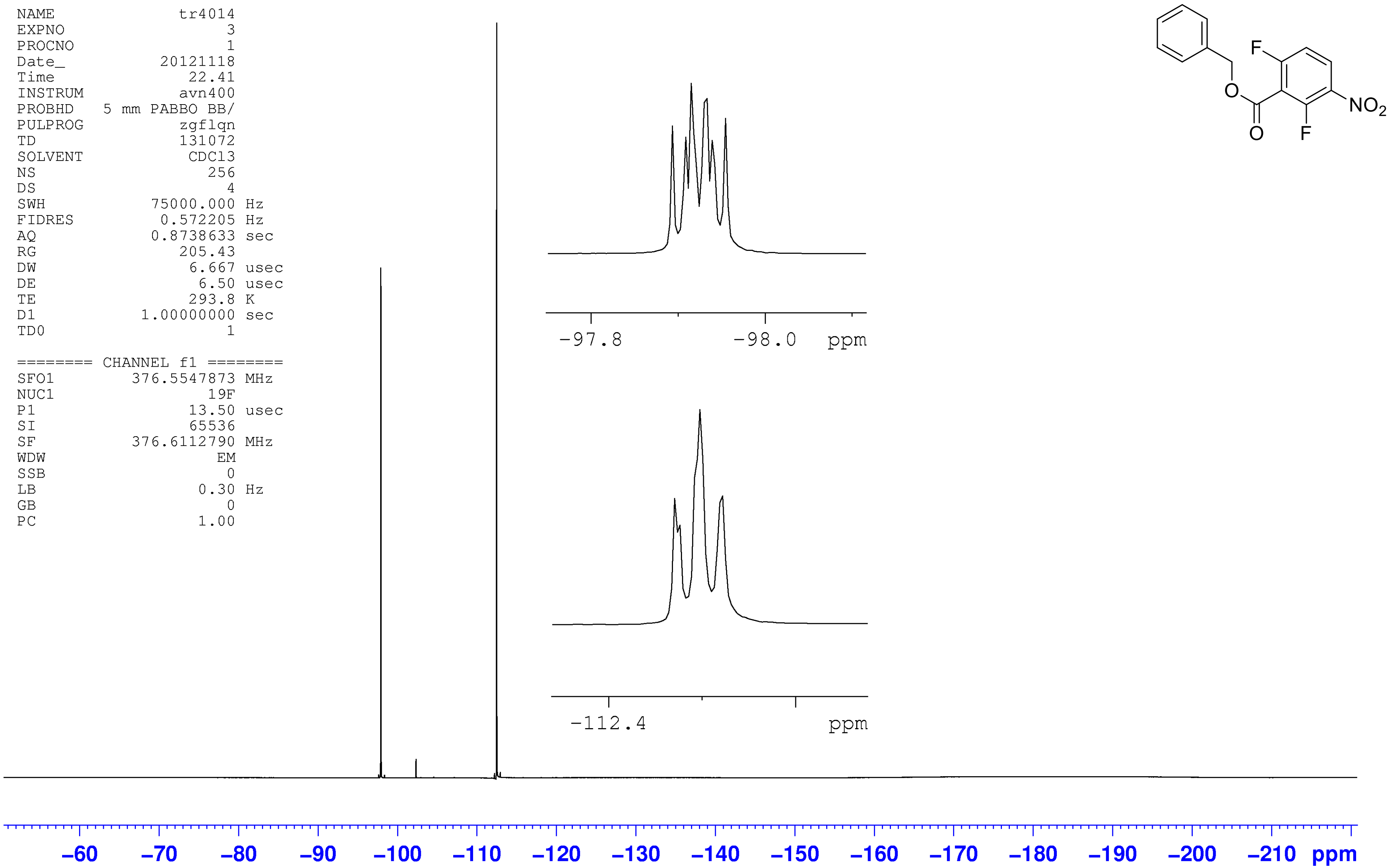

Benzyl 6-fluoro-2-{[(2*R*)-1-methoxy-1-oxopropan-2-yl]amino}-3-nitrobenzoate (*R*)-**18** ^{1}H NMR

```
NAME             tr4021
EXPNO                 1
PROCNO                1
Date_          20121130
Time               3.28
INSTRUM          avn400
PROBHD   5 mm PABBO BB/
PULPROG            zg60
TD                65536
SOLVENT           CDCl3
NS                   16
DS                    2
SWH            8012.820 Hz
FIDRES         0.122266 Hz
AQ            4.0894966 sec
RG                52.03
DW               62.400 usec
DE                 6.50 usec
TE                294.4 K
D1           1.00000000 sec
TD0                   1

======== CHANNEL f1 ========
SFO1        400.2524015 MHz
NUC1                 1H
P1                12.65 usec
SI                32768
SF          400.2500113 MHz
WDW                  EM
SSB                   0
LB                 0.30 Hz
GB                    0
PC                 1.00
```

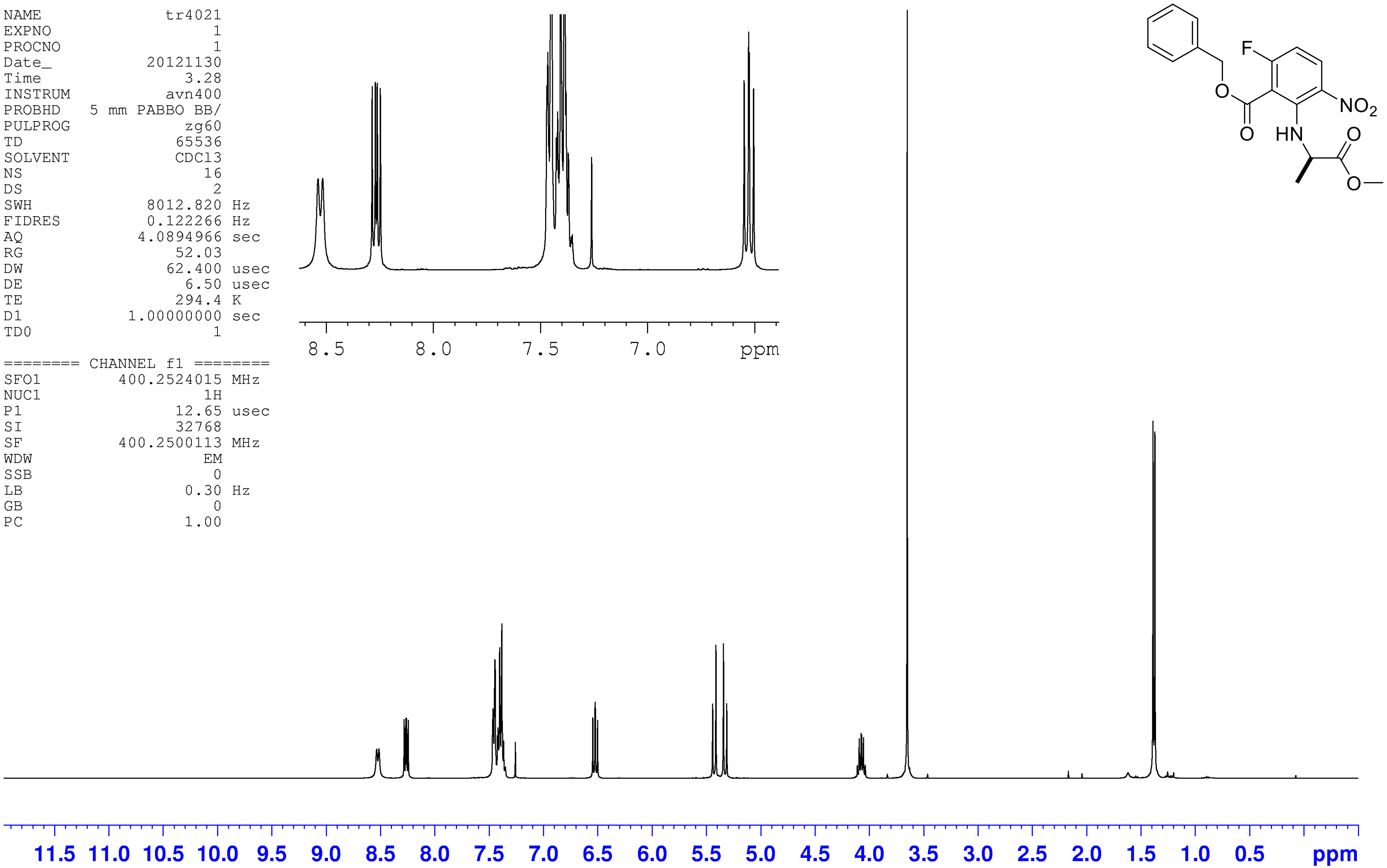

Benzyl 6-fluoro-2-{[(2*R*)-1-methoxy-1-oxopropan-2-yl]amino}-3-nitrobenzoate (*R*)-**18** ^{13}C NMR

```
NAME               tr4021
EXPNO                   2
PROCNO                  1
Date_            20121130
Time                 3.36
INSTRUM            avn400
PROBHD   5 mm PABBO BB/
PULPROG           zgpg30
TD                  32768
SOLVENT             CDCl3
NS                    256
DS                      4
SWH             26041.666 Hz
FIDRES           0.794729 Hz
AQ              0.6291956 sec
RG                 205.43
DW                 19.200 usec
DE                   6.50 usec
TE                  295.5 K
D1             1.00000000 sec
D11            0.03000000 sec
TD0                     1

======== CHANNEL f1 ========
SFO1          100.6530073 MHz
NUC1                  13C
P1                   9.00 usec
SI                  32768
SF            100.6429430 MHz
WDW                    EM
SSB                     0
LB                   1.00 Hz
GB                      0
PC                   1.40
```

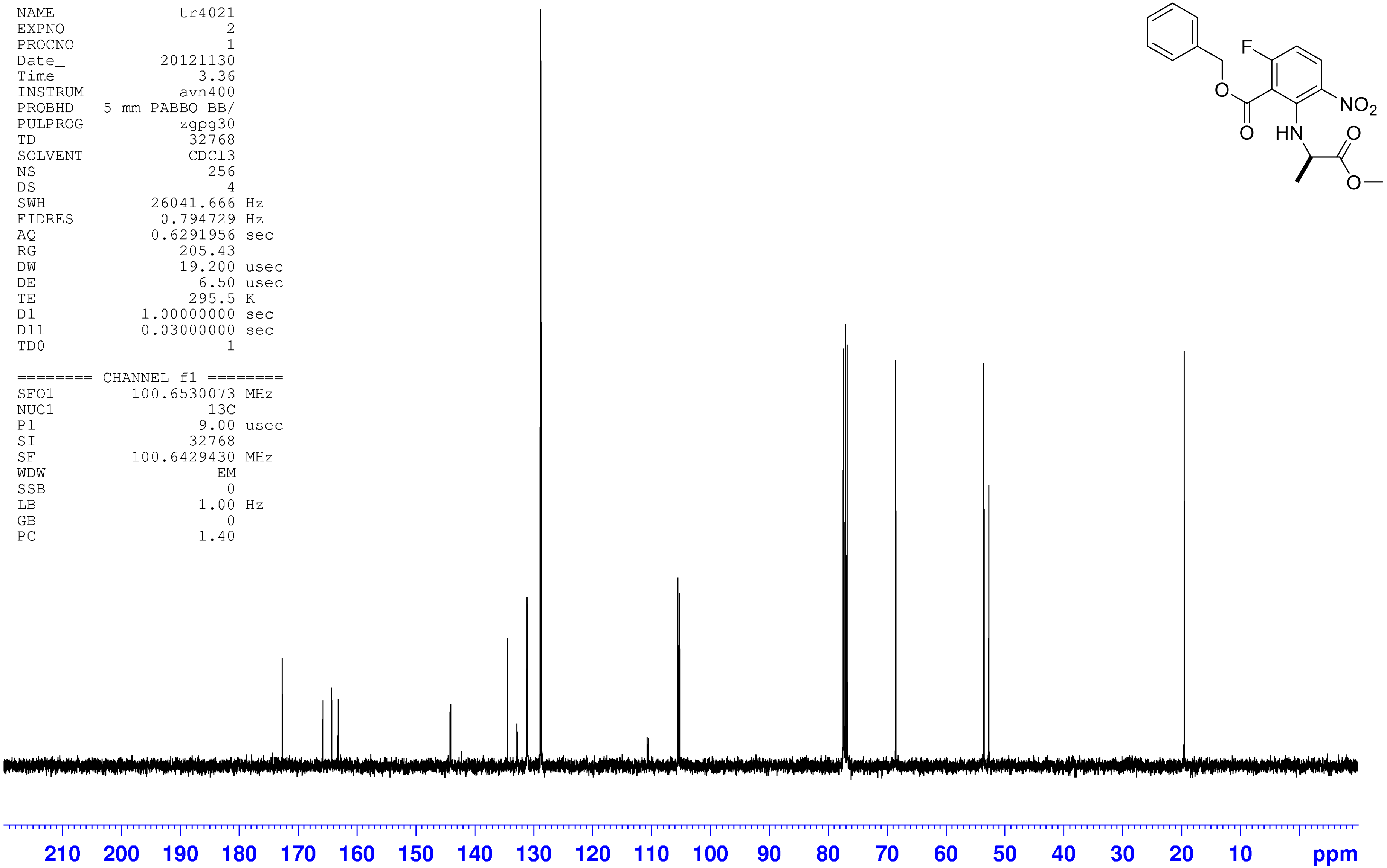

Benzyl 6-fluoro-2-{[(2*R*)-1-methoxy-1-oxopropan-2-yl]amino}-3-nitrobenzoate (*R*)-**18** ^{19}F NMR

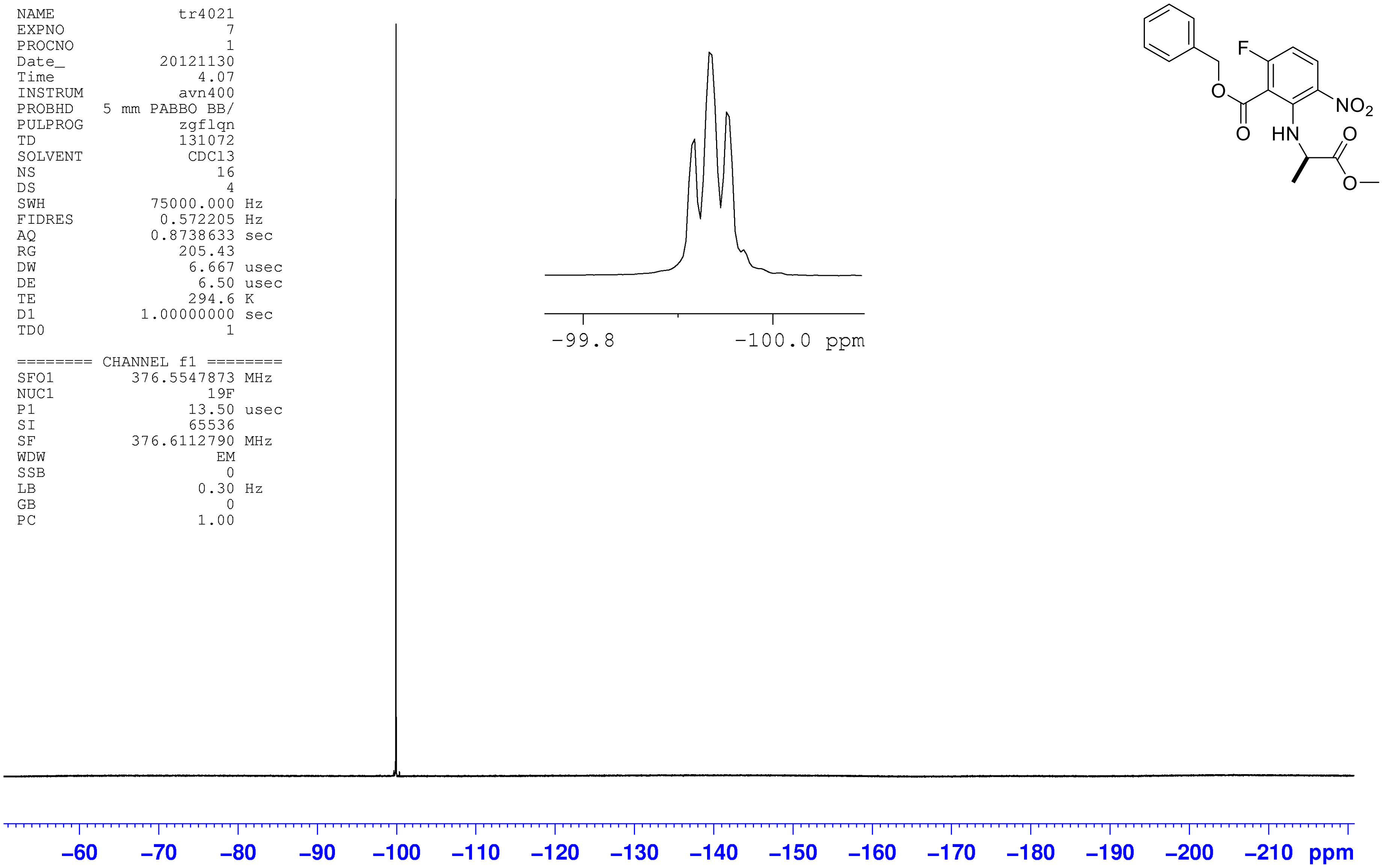

Benzyl (3*R*)-6-fluoro-3-methyl-2-oxo-1,2,3,4-tetrahydroquinoxaline-5-carboxylate (*R*)-**19** ^{1}H NMR

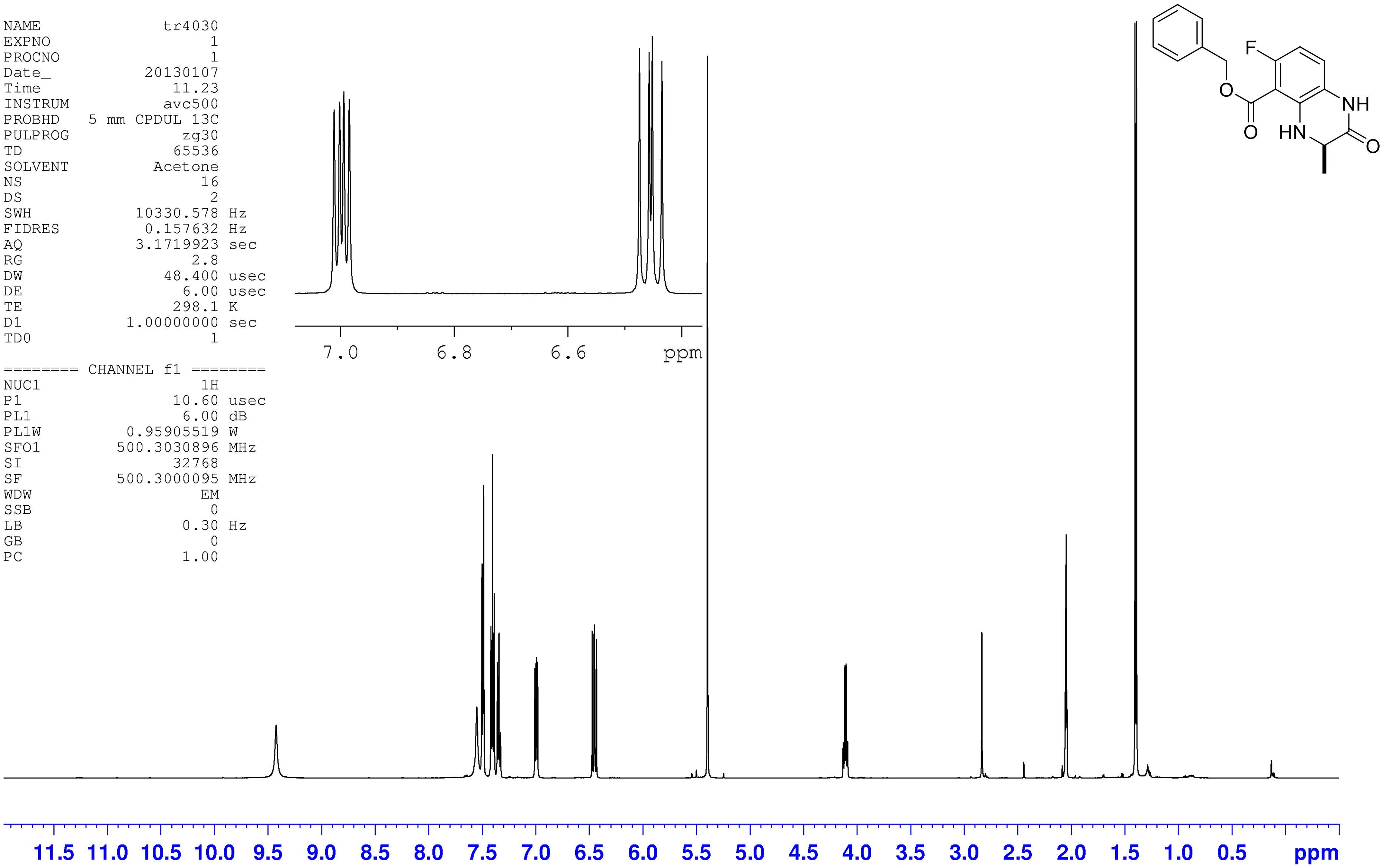

Benzyl (3*R*)-6-fluoro-3-methyl-2-oxo-1,2,3,4-tetrahydroquinoxaline-5-carboxylate (*R*)-**19** ^{13}C NMR

```
NAME                tr4030
EXPNO                    4
PROCNO                   1
Date_             20130107
Time                 11.50
INSTRUM             avc500
PROBHD   5 mm CPDUL 13C
PULPROG            zgpg30
TD                   65536
SOLVENT            Acetone
NS                     512
DS                       2
SWH            31250.000 Hz
FIDRES          0.476837 Hz
AQ             1.0486259 sec
RG                     912
DW                  16.000 usec
DE                  20.00 usec
TE                  298.0 K
D1             2.00000000 sec
D11            0.03000000 sec
TD0                      1

======== CHANNEL f1 ========
NUC1                   13C
P1                  10.25 usec
PL1                  8.00 dB
PL1W           1.62029624 W
SFO1          125.8131151 MHz

======== CHANNEL f2 ========
CPDPRG2            waltz16
NUC2                    1H
PCPD2               80.00 usec
PL2                  6.00 dB
PL12                23.56 dB
PL13                29.56 dB
PL2W           0.95905519 W
PL12W          0.01682068 W
PL13W          0.00422516 W
SFO2          500.3020012 MHz
SI                   32768
SF            125.8004258 MHz
WDW                     EM
SSB                      0
LB                   1.00 Hz
GB                       0
PC                    1.40
```

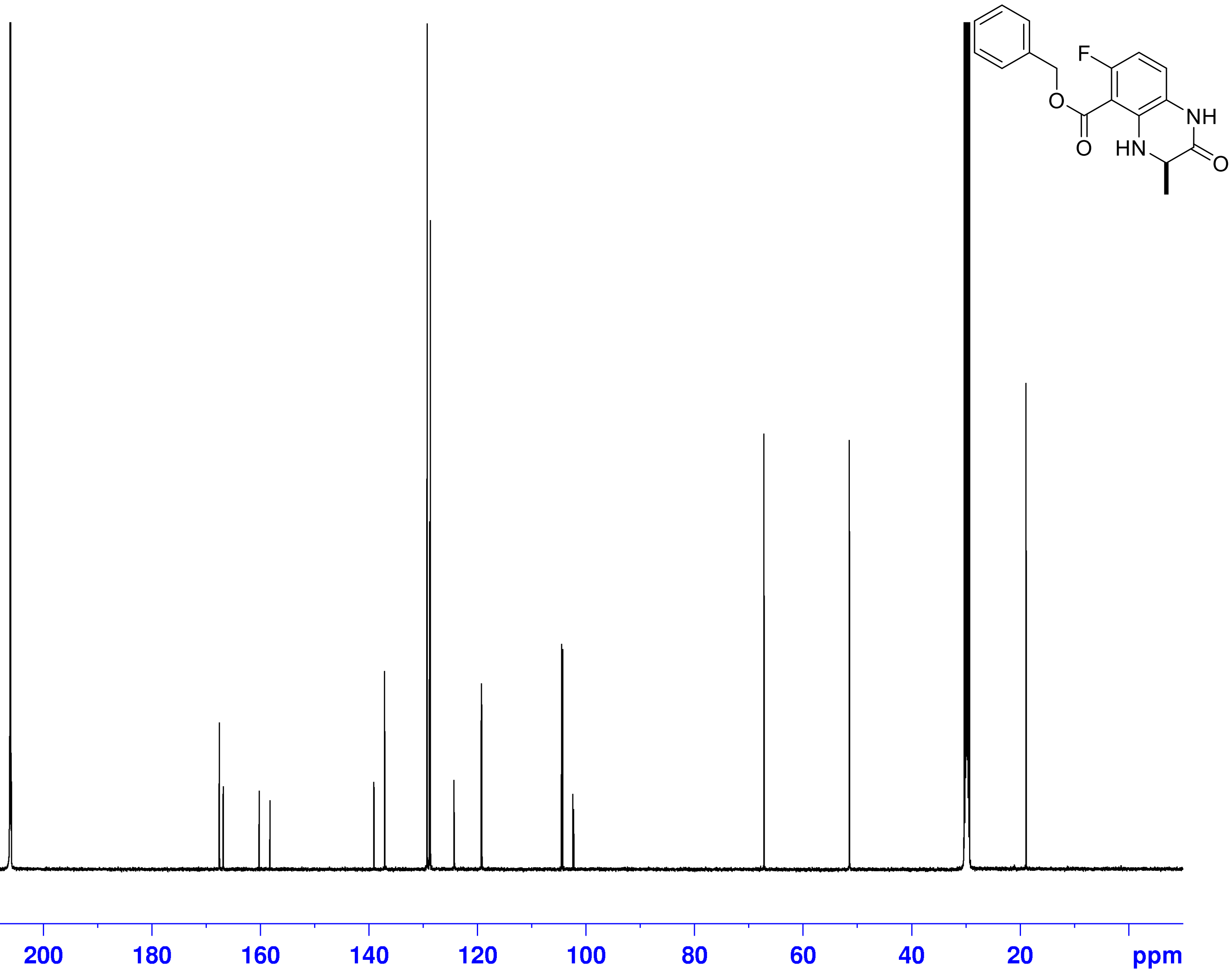

Benzyl (3*R*)-6-fluoro-3-methyl-2-oxo-1,2,3,4-tetrahydroquinoxaline-5-carboxylate (*R*)-**19** ^{19}F NMR

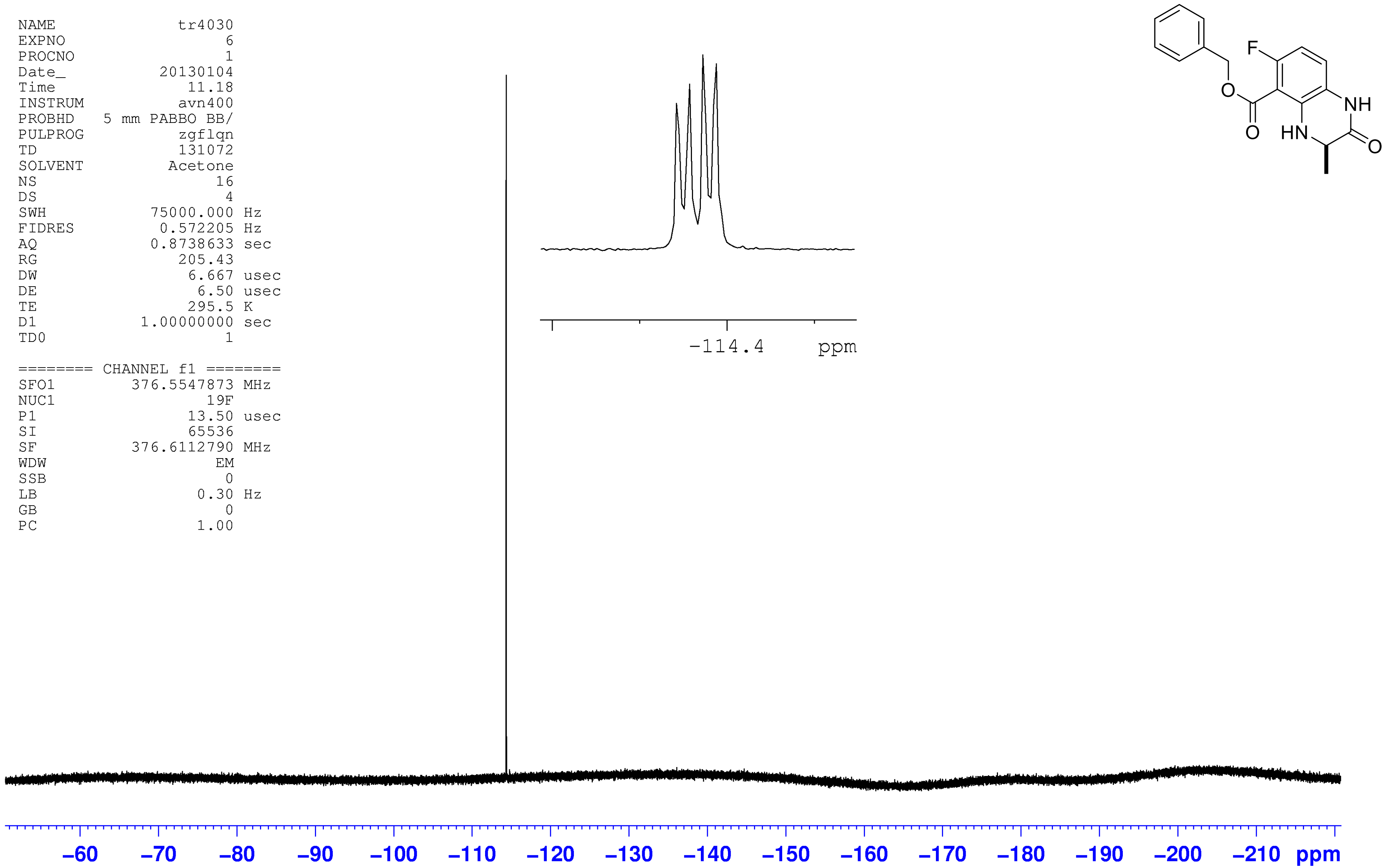


```
NAME              tr4030
EXPNO                  6
PROCNO                 1
Date_           20130104
Time               11.18
INSTRUM           avn400
PROBHD   5 mm PABBO BB/
PULPROG          zgflqn
TD                131072
SOLVENT          Acetone
NS                    16
DS                     4
SWH            75000.000 Hz
FIDRES          0.572205 Hz
AQ             0.8738633 sec
RG                205.43
DW                 6.667 usec
DE                  6.50 usec
TE                 295.5 K
D1            1.00000000 sec
TD0                    1

======== CHANNEL f1 ========
SFO1         376.5547873 MHz
NUC1                 19F
P1                 13.50 usec
SI                 65536
SF           376.6112790 MHz
WDW                   EM
SSB                    0
LB                  0.30 Hz
GB                     0
PC                  1.00
```

(3*R*)-6-Fluoro-3-methyl-2-oxo-1,2,3,4-tetrahydroquinoxaline-5-carboxylic acid (*R*)-**20** ^{1}H NMR

```
NAME              tr4037
EXPNO                  1
PROCNO                 1
Date_           20131020
Time               16.59
INSTRUM           drx500
PROBHD   5 mm PABBO BB/
PULPROG             zg60
TD                 65536
SOLVENT             DMSO
NS                    16
DS                     2
SWH            10330.578 Hz
FIDRES          0.157632 Hz
AQ             3.1720407 sec
RG                 161.3
DW                48.400 usec
DE                 17.00 usec
TE                 298.7 K
D1            1.00000000 sec

======== CHANNEL f1 ========
NUC1                  1H
P1                 11.00 usec
PL1                 0.00 dB
SFO1         500.1325007 MHz
SI                 32768
SF           500.1300055 MHz
WDW                   EM
SSB                    0
LB                  0.30 Hz
GB                     0
PC                  1.00
```

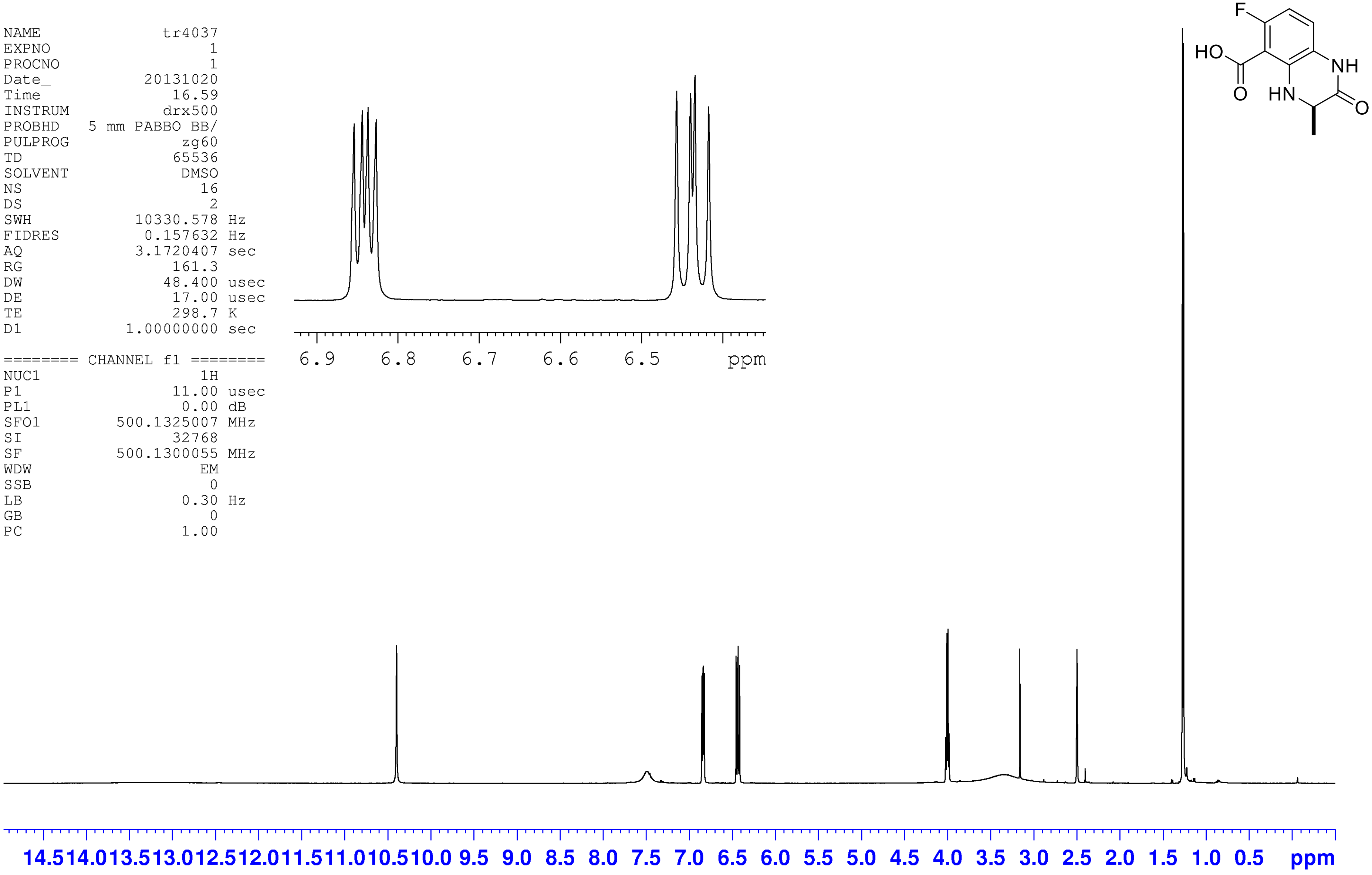

(3*R*)-6-Fluoro-3-methyl-2-oxo-1,2,3,4-tetrahydroquinoxaline-5-carboxylic acid (*R*)-**20** ^{13}C NMR

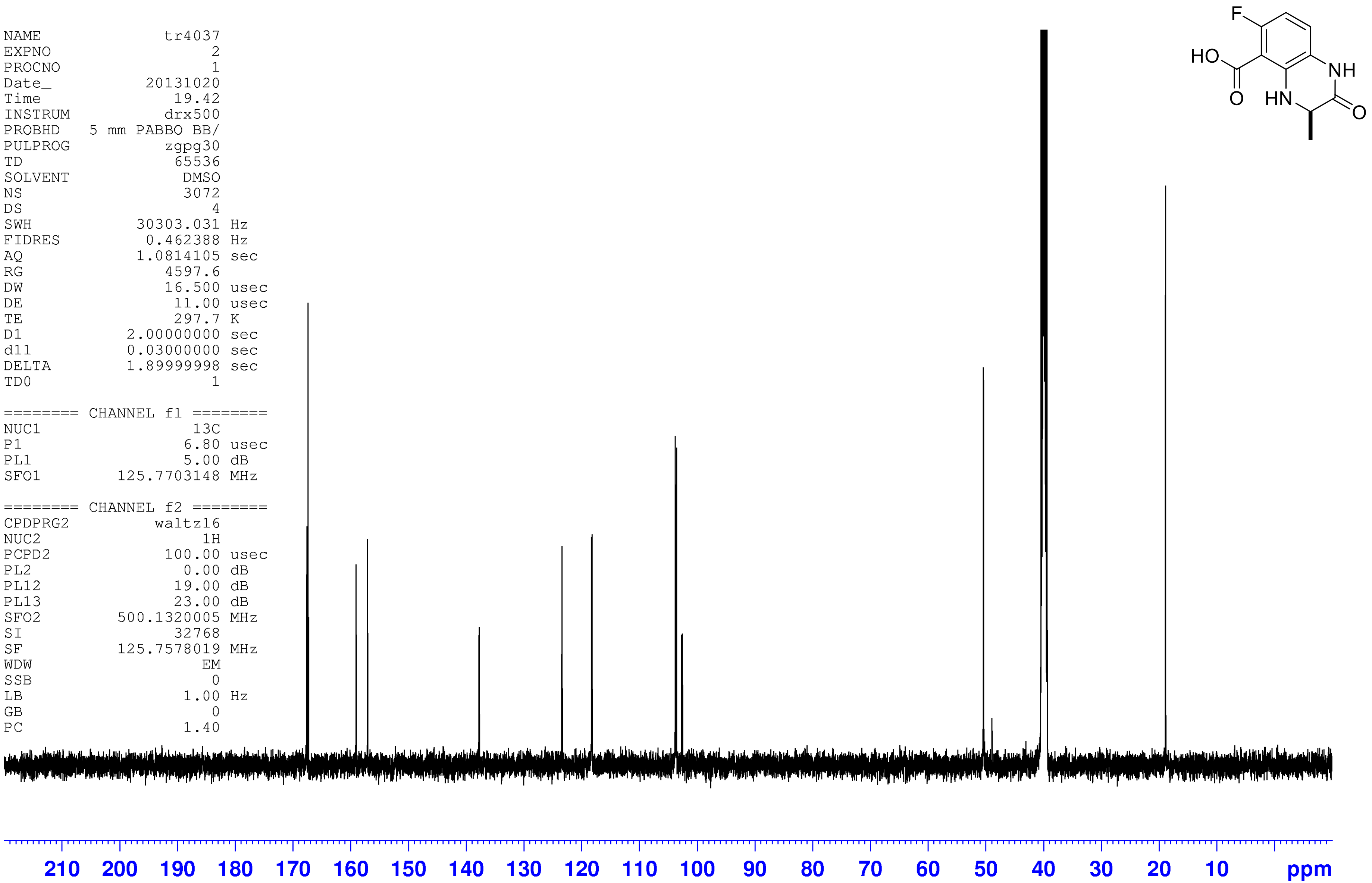

(3*R*)-6-Fluoro-3-methyl-2-oxo-1,2,3,4-tetrahydroquinoxaline-5-carboxylic acid (*R*)-**20** ^{19}F NMR

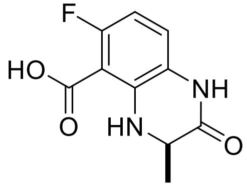


```
NAME            tr4037-2
EXPNO                  2
PROCNO                 1
Date_           20131121
Time               20.27
INSTRUM           avb500
PROBHD   5 mm PATXI 1H/
PULPROG          zgflqn
TD                131072
SOLVENT             DMSO
NS                   128
DS                     4
SWH           113636.367 Hz
FIDRES          0.866977 Hz
AQ             0.5767668 sec
RG                  2050
DW                 4.400 usec
DE                  6.50 usec
TE                 298.0 K
D1            1.00000000 sec
TD0                    1

======== CHANNEL f1 ========
SFO1         470.4041911 MHz
NUC1                 19F
P1                 10.00 usec
SI                 65536
SF           470.4512360 MHz
WDW                   EM
SSB                    0
LB                  0.30 Hz
GB                     0
PC                  1.00
```

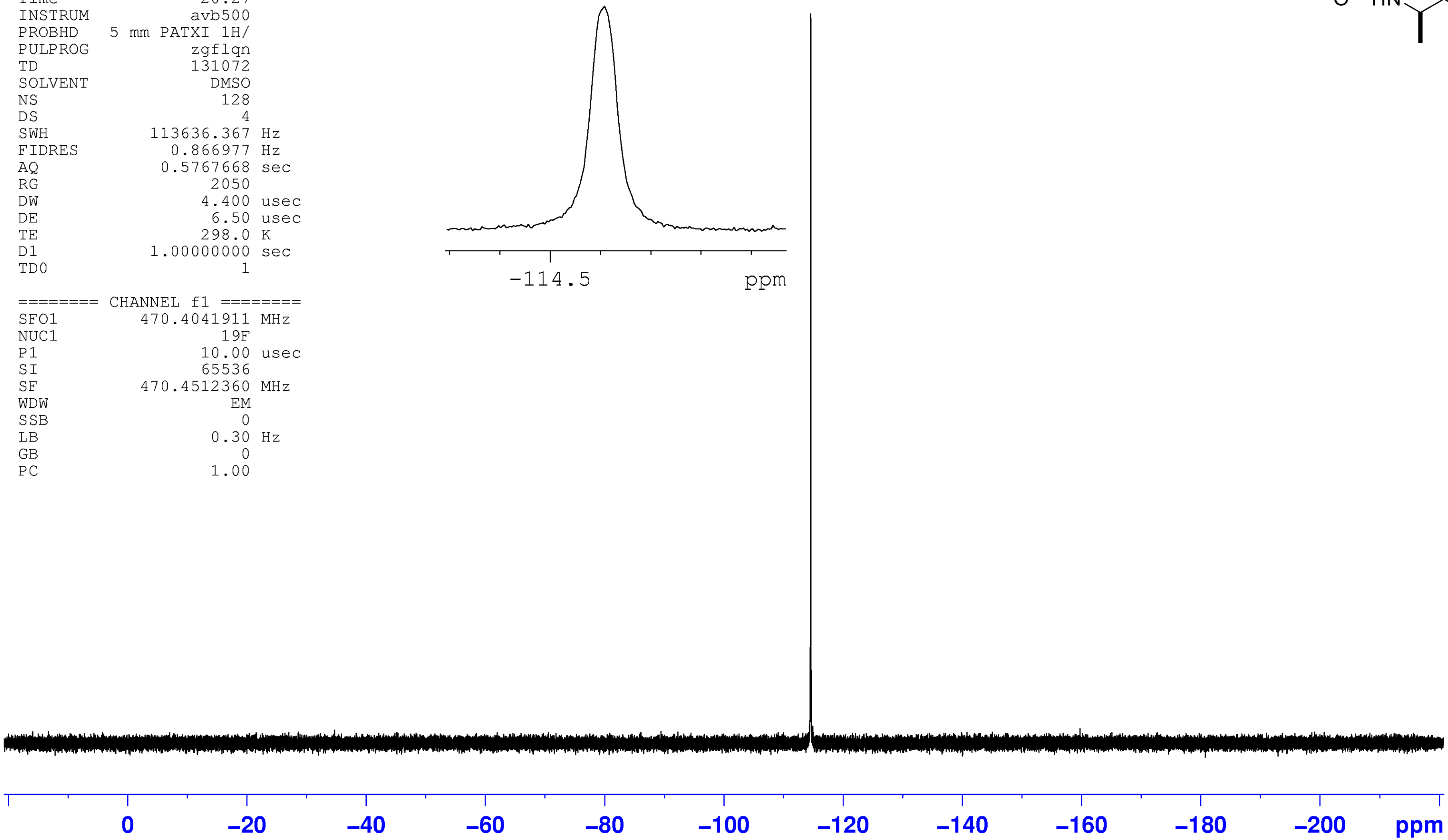

Benzyl 2-(benzyloxy)-5-fluoro-3-nitrobenzoate **22** ^{1}H NMR

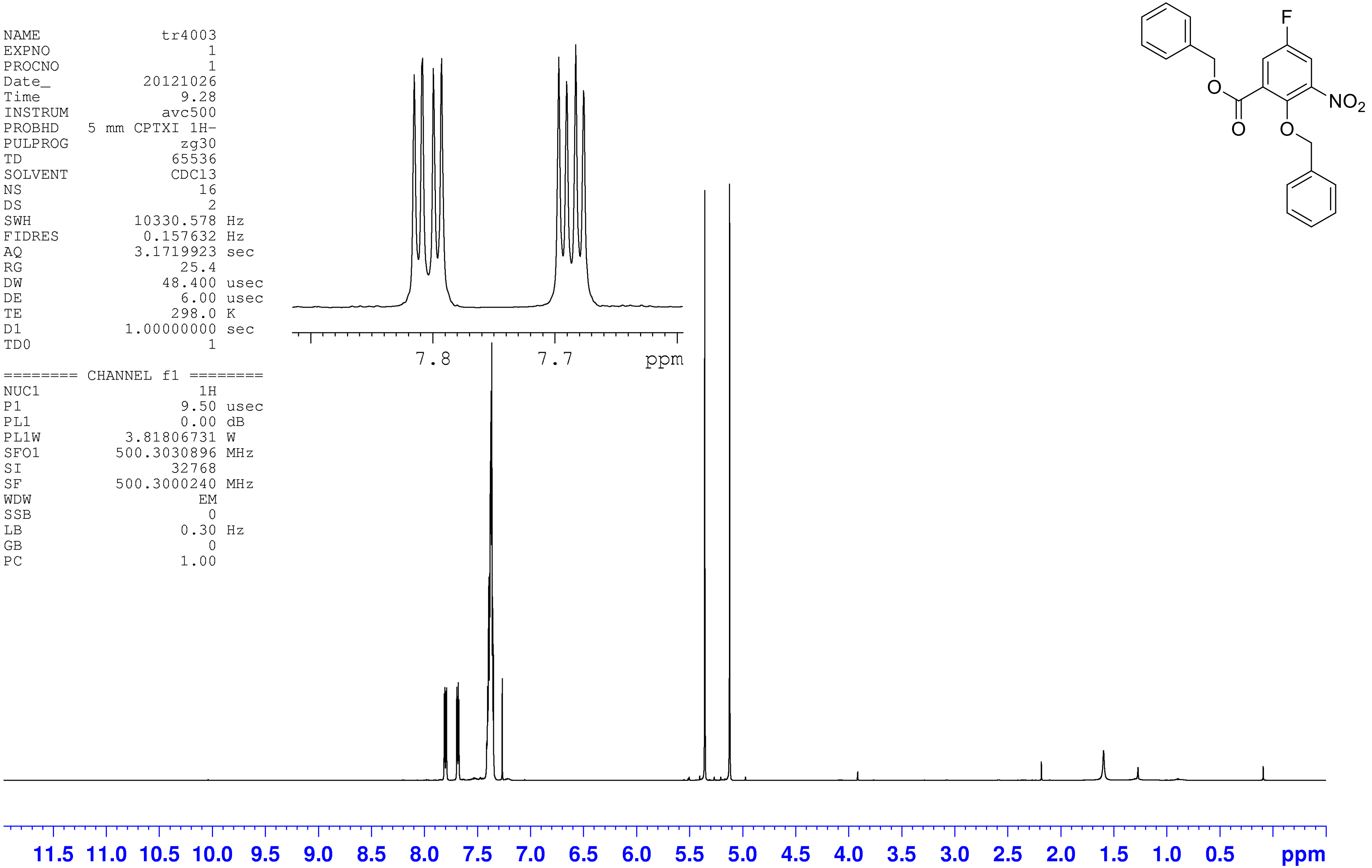

Benzyl 2-(benzyloxy)-5-fluoro-3-nitrobenzoate **22** ^{13}C NMR

```
NAME               tr4003
EXPNO                   2
PROCNO                  1
Date_            20121026
Time                10.43
INSTRUM            avc500
PROBHD   5 mm CPTXI 1H-
PULPROG           zgpg30
TD                  65536
SOLVENT             CDCl3
NS                   1024
DS                      2
SWH             31250.000 Hz
FIDRES           0.476837 Hz
AQ              1.0486259 sec
RG                    228
DW                 16.000 usec
DE                  20.00 usec
TE                  298.0 K
D1             2.00000000 sec
D11            0.03000000 sec
TD0                     1

======== CHANNEL f1 ========
NUC1                  13C
P1                  17.00 usec
PL1                 -6.00 dB
PL1W          40.70000076 W
SFO1          125.8131151 MHz

======== CHANNEL f2 ========
CPDPRG2           waltz16
NUC2                   1H
PCPD2               80.00 usec
PL2                  0.00 dB
PL12                18.51 dB
PL13                24.50 dB
PL2W           3.81806731 W
PL12W          0.05380759 W
PL13W          0.01354701 W
SFO2          500.3020012 MHz
SI                  32768
SF            125.8005438 MHz
WDW                    EM
SSB                     0
LB                   1.00 Hz
GB                      0
PC                   1.40
```

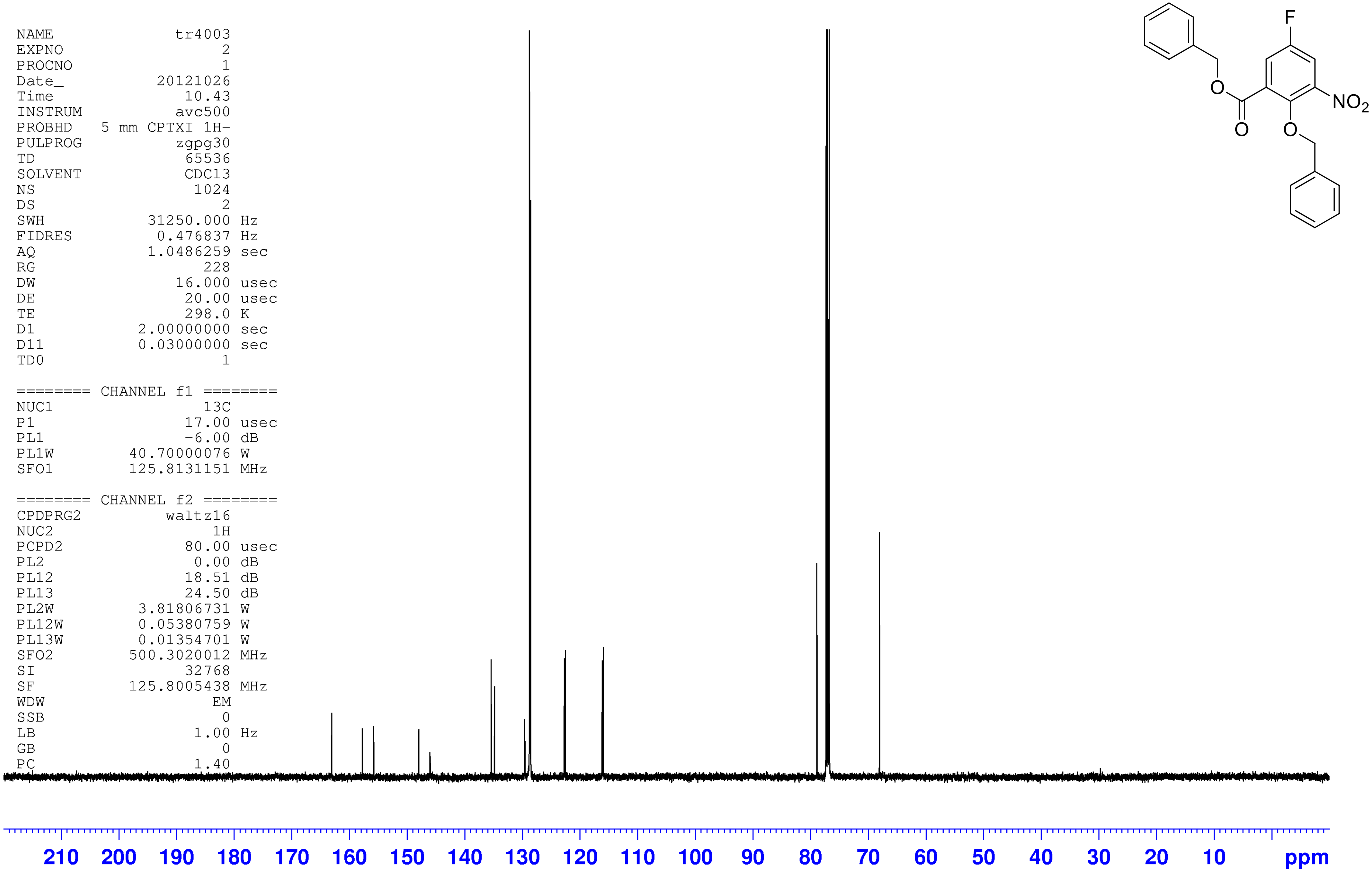

Benzyl 2-(benzyloxy)-5-fluoro-3-nitrobenzoate **22** ^{19}F NMR

```
NAME              tr4003
EXPNO                  3
PROCNO                 1
Date_           20121030
Time                3.14
INSTRUM           avn400
PROBHD   5 mm PABBO BB/
PULPROG          zgflqn
TD                131072
SOLVENT            CDCl3
NS                   256
DS                     4
SWH            75000.000 Hz
FIDRES          0.572205 Hz
AQ             0.8738633 sec
RG                205.43
DW                 6.667 usec
DE                  6.50 usec
TE                 293.3 K
D1            1.00000000 sec
TD0                    1

======== CHANNEL f1 ========
SFO1         376.5547873 MHz
NUC1                 19F
P1                 13.50 usec
SI                 65536
SF           376.6112790 MHz
WDW                   EM
SSB                    0
LB                  0.30 Hz
GB                     0
PC                  1.00
```

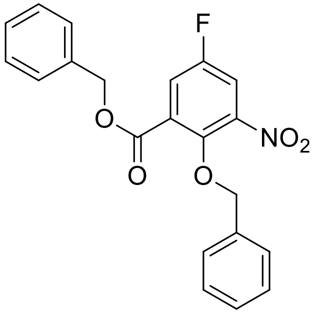

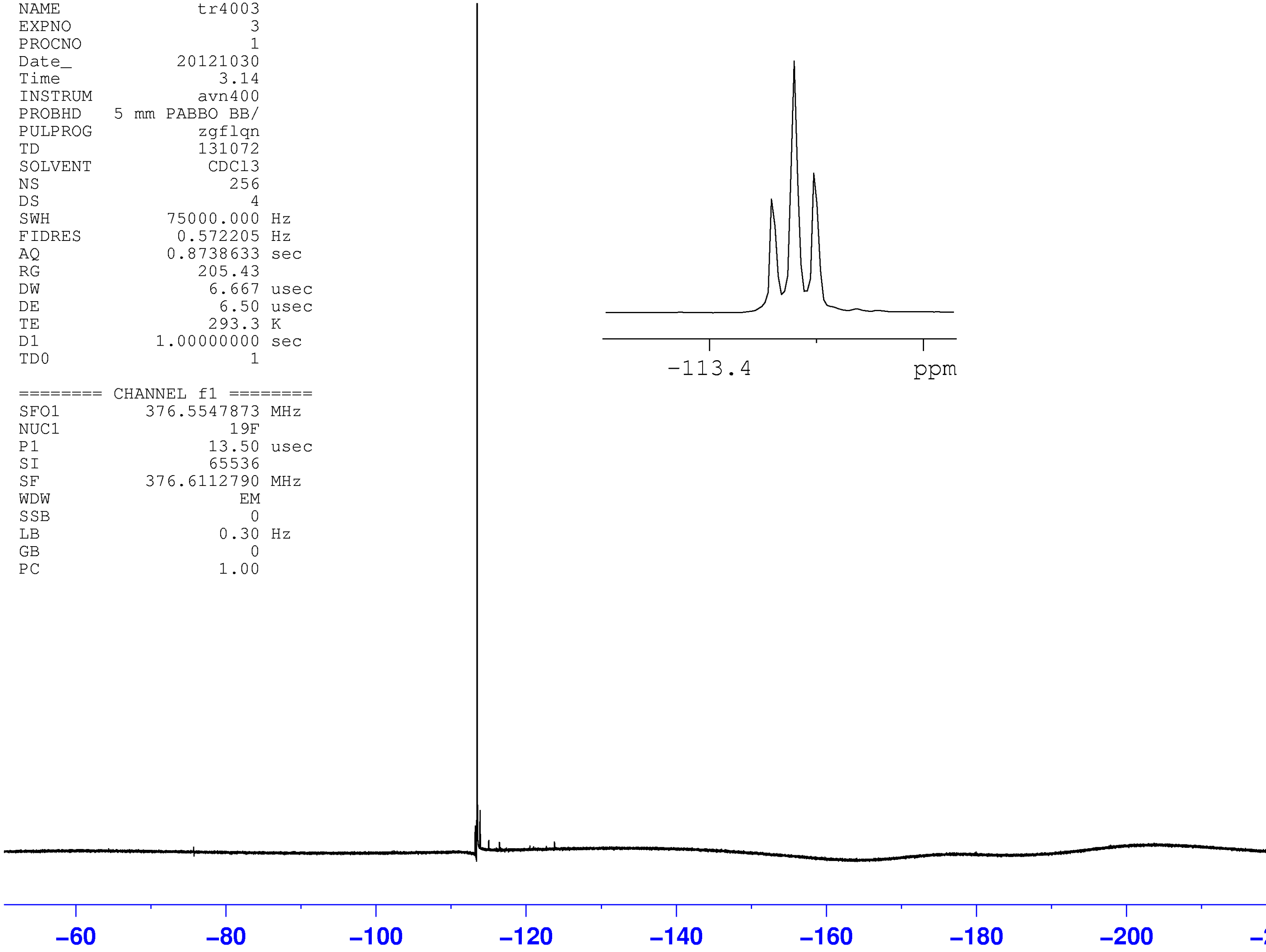

Benzyl 5-fluoro-2-hydroxy-3-nitrobenzoate **23** ^{1}H NMR

```
NAME             tr4002
EXPNO                 1
PROCNO                1
Date_          20121207
Time               7.40
INSTRUM          avc500
PROBHD   5 mm CPDUL 13C
PULPROG            zg30
TD                65536
SOLVENT           CDCl3
NS                   16
DS                    2
SWH           10330.578 Hz
FIDRES         0.157632 Hz
AQ            3.1719923 sec
RG                  2.8
DW               48.400 usec
DE                 6.00 usec
TE                298.0 K
D1           1.00000000 sec
TD0                   1

======== CHANNEL f1 ========
NUC1                 1H
P1                10.60 usec
PL1                6.00 dB
PL1W         0.95905519 W
SFO1        500.3030896 MHz
SI                32768
SF          500.3000240 MHz
WDW                  EM
SSB                   0
LB                 0.30 Hz
GB                    0
PC                 1.00
```

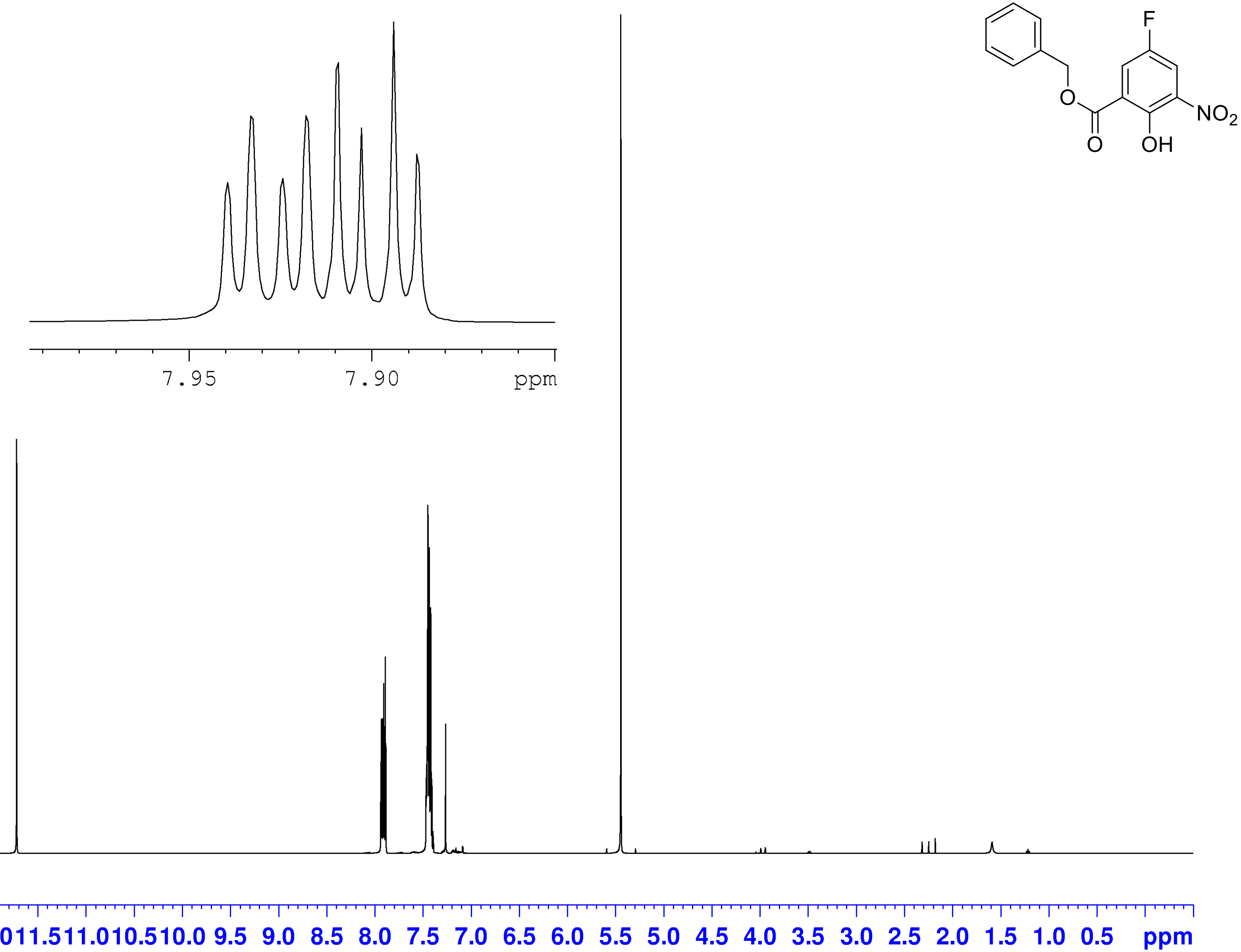

Benzyl 5-fluoro-2-hydroxy-3-nitrobenzoate **23** ^{13}C NMR

```
NAME               tr4002
EXPNO                   3
PROCNO                  1
Date_            20121207
Time                 8.08
INSTRUM            avc500
PROBHD   5 mm CPDUL 13C
PULPROG           zgpg30
TD                  65536
SOLVENT             CDCl3
NS                    256
DS                      2
SWH             31250.000 Hz
FIDRES           0.476837 Hz
AQ              1.0486259 sec
RG                    912
DW                 16.000 usec
DE                  20.00 usec
TE                  298.0 K
D1             2.00000000 sec
D11            0.03000000 sec
TD0                     1

======== CHANNEL f1 ========
NUC1                  13C
P1                  10.25 usec
PL1                  8.00 dB
PL1W           1.62029624 W
SFO1          125.8131151 MHz

======== CHANNEL f2 ========
CPDPRG2           waltz16
NUC2                   1H
PCPD2               80.00 usec
PL2                  6.00 dB
PL12                23.56 dB
PL13                29.56 dB
PL2W           0.95905519 W
PL12W          0.01682068 W
PL13W          0.00422516 W
SFO2          500.3020012 MHz
SI                  32768
SF            125.8005244 MHz
WDW                    EM
SSB                     0
LB                   1.00 Hz
GB                      0
PC                   1.40
```

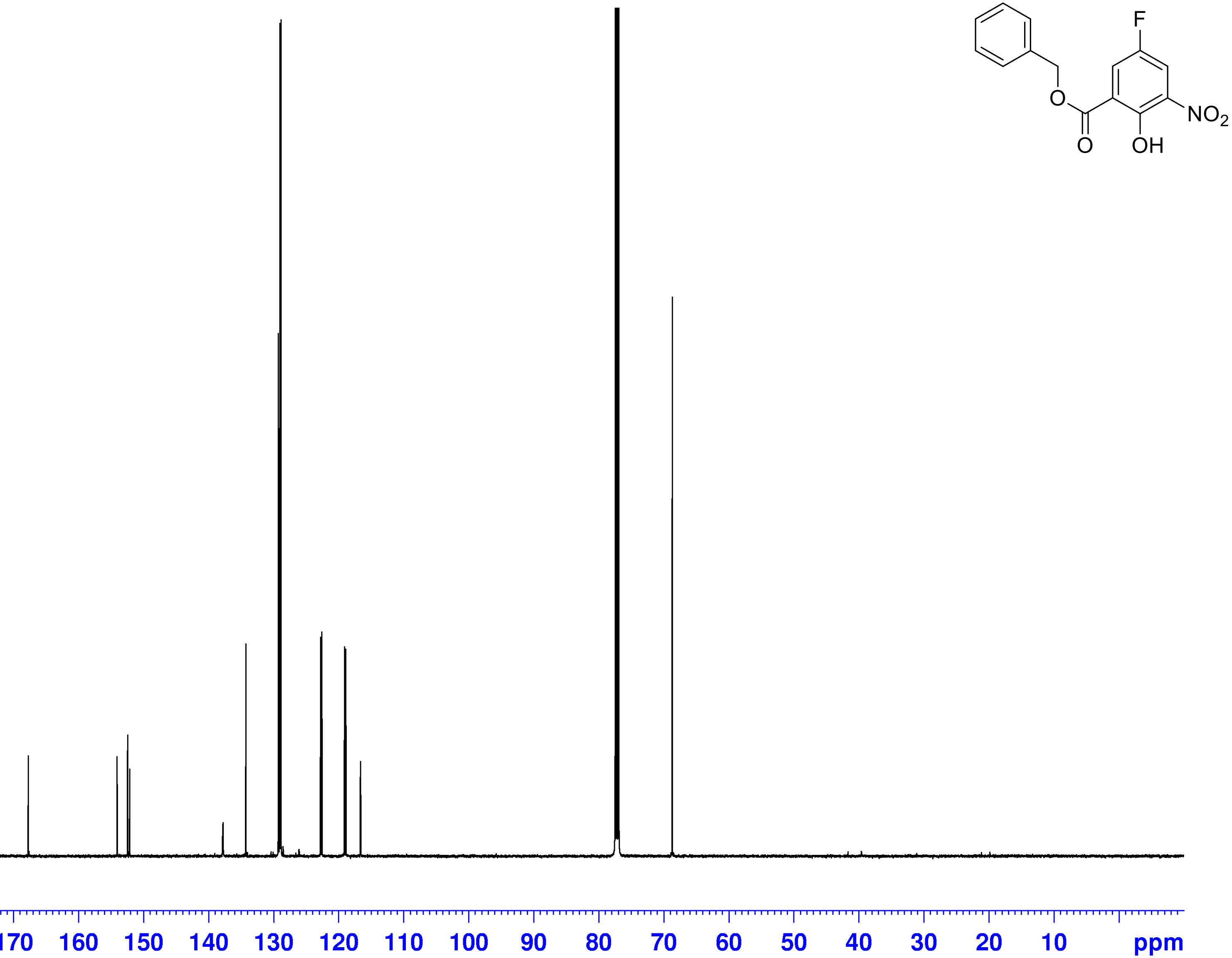

Benzyl 5-fluoro-2-hydroxy-3-nitrobenzoate **23** ^{19}F NMR

```
NAME                tr4002
EXPNO                    6
PROCNO                   1
Date_             20121204
Time                 13.14
INSTRUM             avn400
PROBHD   5 mm PABBO BB/
PULPROG             zgflqn
TD                  131072
SOLVENT              CDCl3
NS                      16
DS                       4
SWH              75000.000 Hz
FIDRES            0.572205 Hz
AQ               0.8738633 sec
RG                  205.43
DW                   6.667 usec
DE                    6.50 usec
TE                   294.2 K
D1              1.00000000 sec
TD0                      1

======== CHANNEL f1 ========
SFO1           376.5547873 MHz
NUC1                   19F
P1                   13.50 usec
SI                   65536
SF             376.6112790 MHz
WDW                     EM
SSB                      0
LB                    0.30 Hz
GB                       0
PC                    1.00
```

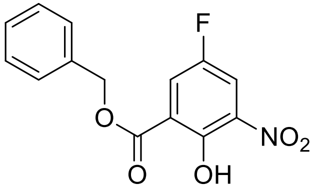

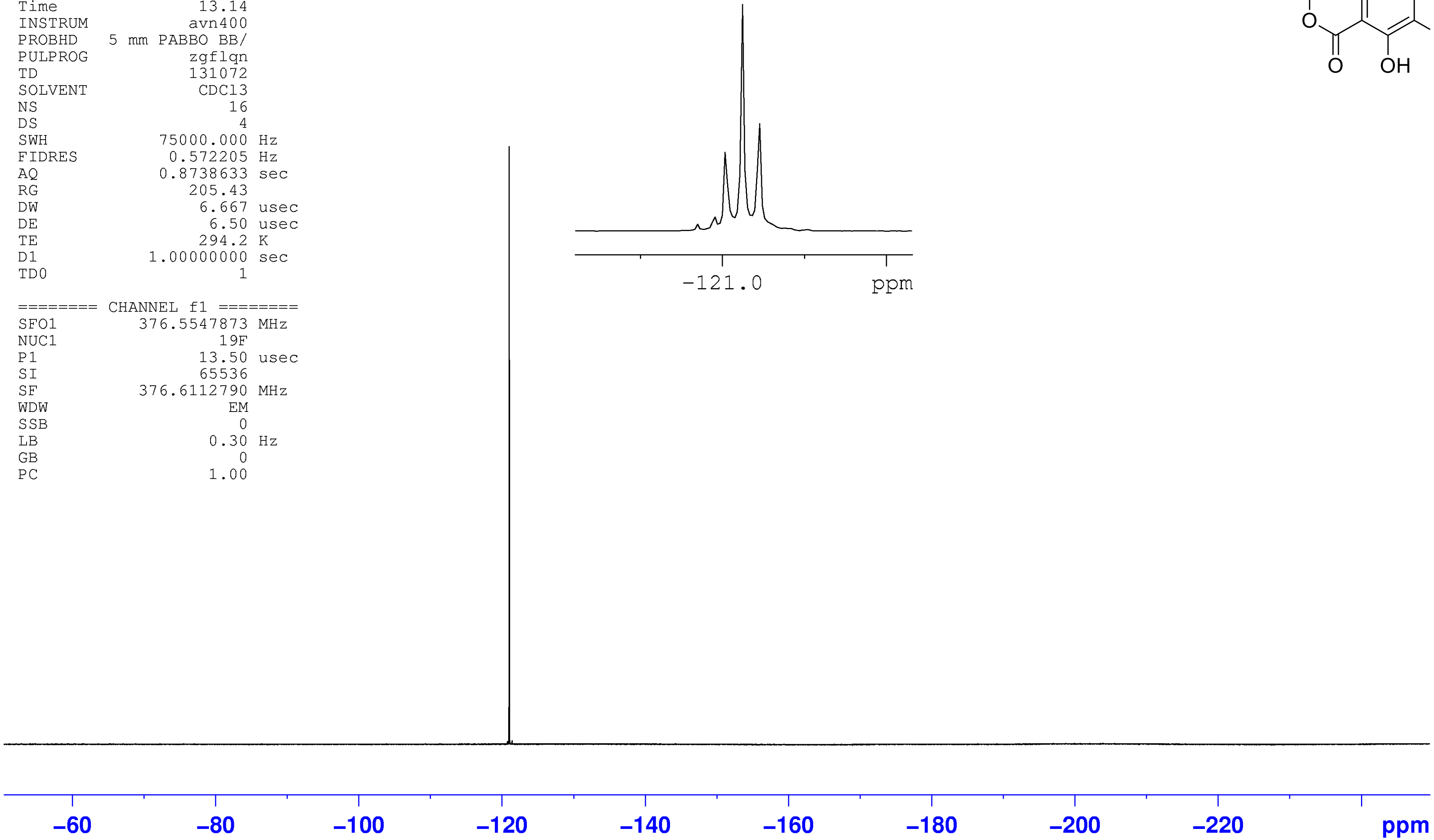

Benzyl 5-fluoro-3-nitro-2-{[(trifluoromethyl)sulfonyl]oxy}benzoate **24** ^{1}H NMR

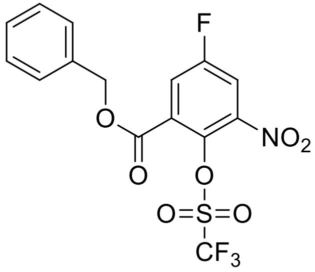

NAME	tr4009
EXPNO	1
PROCNO	1
Date_	20131031
Time	1.42
INSTRUM	avc500
PROBHD	5 mm CPDUL 13C
PULPROG	zg30
TD	65536
SOLVENT	CDCl3
NS	16
DS	4
SWH	10330.578 Hz
FIDRES	0.157632 Hz
AQ	3.1719923 sec
RG	4
DW	48.400 usec
DE	10.00 usec
TE	298.1 K
D1	1.00000000 sec
TD0	1

======== CHANNEL f1 ========

SFO1	500.3030896 MHz
NUC1	1H
P1	15.00 usec
SI	65536
SF	500.3000138 MHz
WDW	EM
SSB	0
LB	0.30 Hz
GB	0
PC	1.00

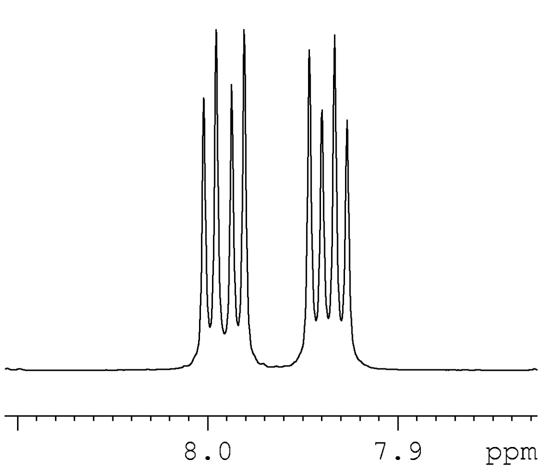

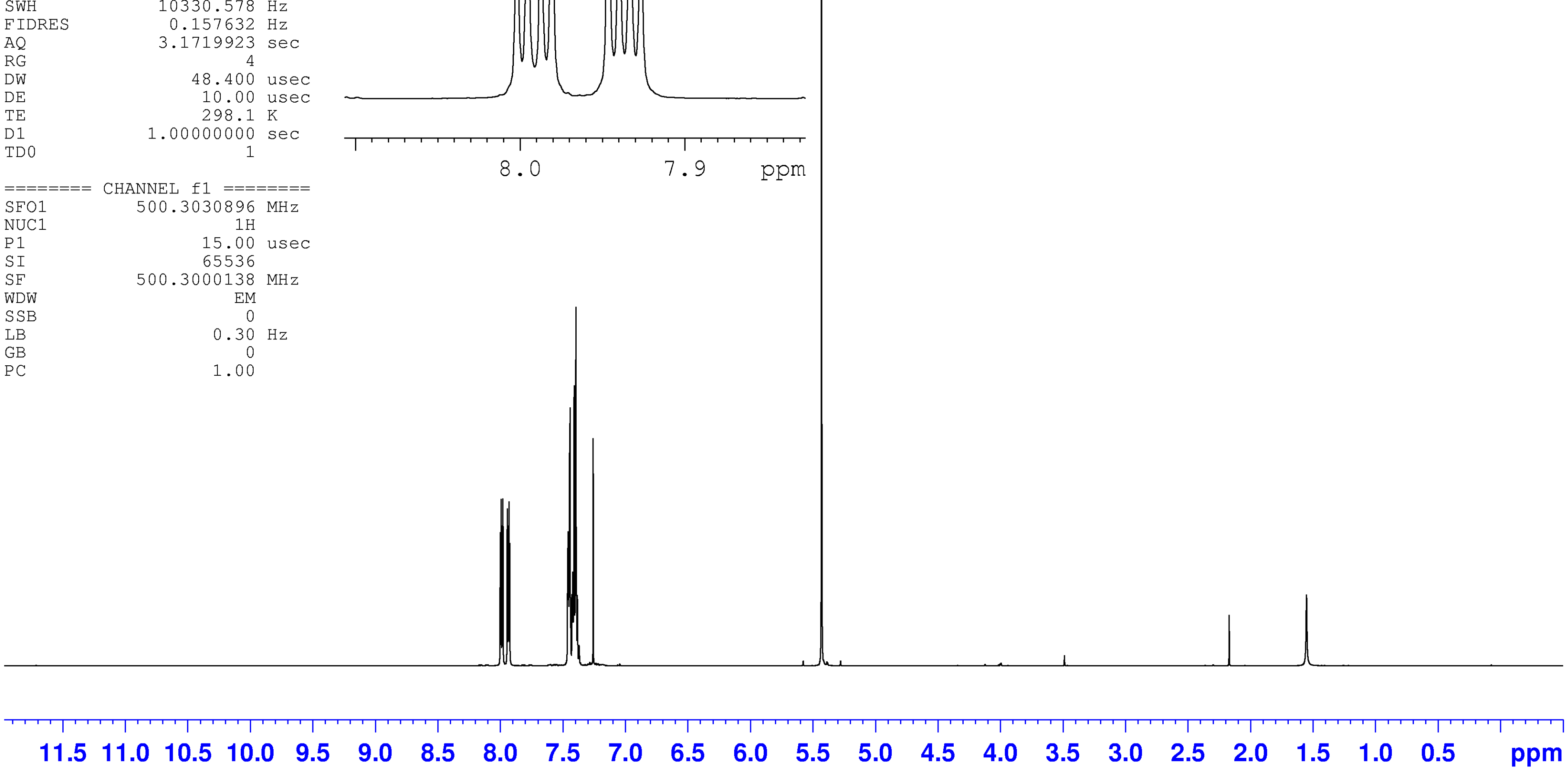

Benzyl 5-fluoro-3-nitro-2-{[(trifluoromethyl)sulfonyl]oxy}benzoate **24** ^{13}C NMR

NAME	tr4009
EXPNO	2
PROCNO	1
Date_	20131031
Time	2.36
INSTRUM	avc500
PROBHD	5 mm CPDUL 13C
PULPROG	zgpg30
TD	65536
SOLVENT	CDCl3
NS	1024
DS	2
SWH	31250.000 Hz
FIDRES	0.476837 Hz
AQ	1.0486259 sec
RG	912
DW	16.000 usec
DE	18.00 usec
TE	298.0 K
D1	2.00000000 sec
D11	0.03000000 sec
TD0	1
======== CHANNEL f1 ========	
SFO1	125.8131151 MHz
NUC1	13C
P1	10.00 usec
SI	32768
SF	125.8005187 MHz
WDW	EM
SSB	0
LB	1.00 Hz
GB	0
PC	1.40

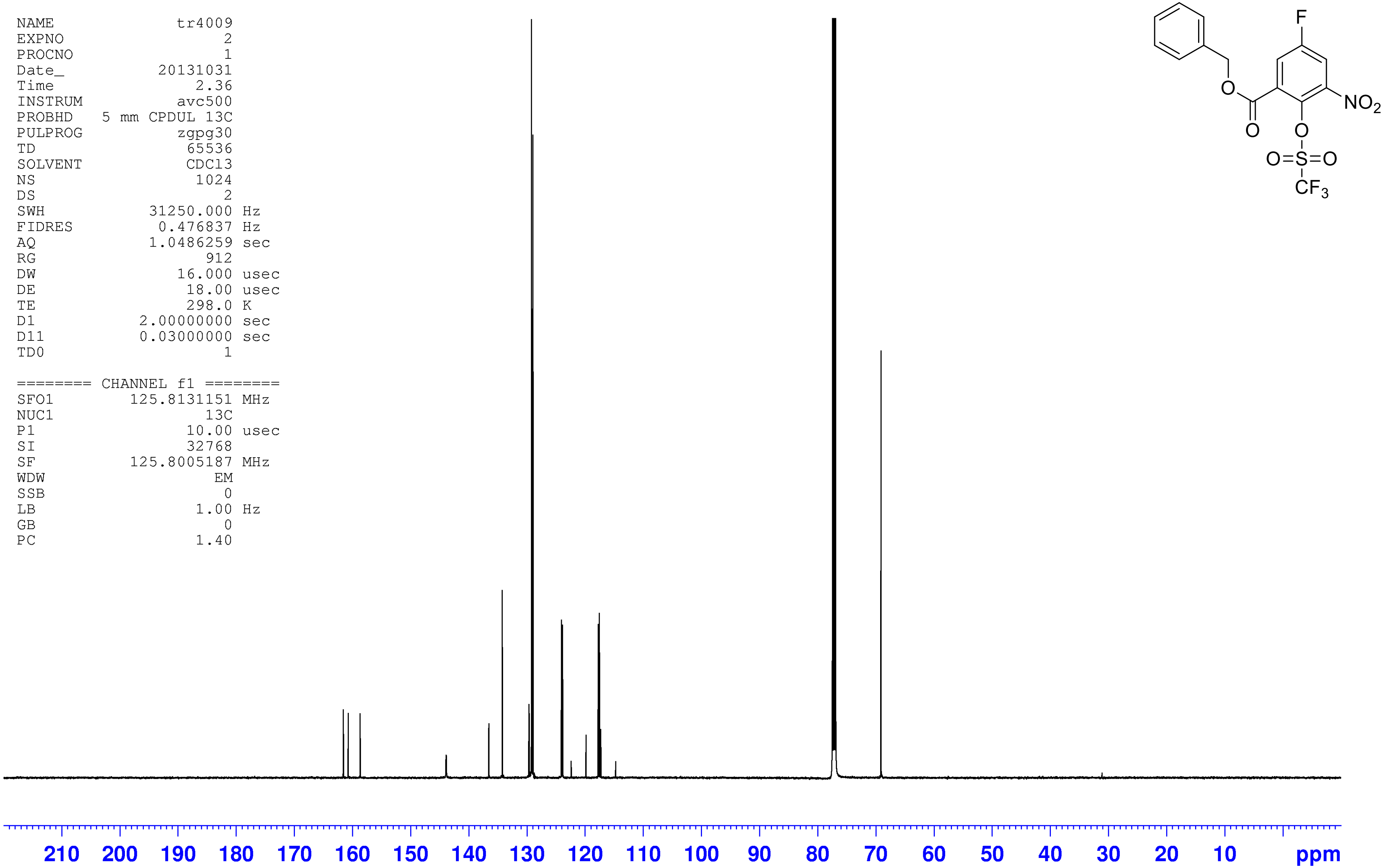

Benzyl 5-fluoro-3-nitro-2-{[(trifluoromethyl)sulfonyl]oxy}benzoate **24** ^{19}F NMR

```
NAME               tr4009
EXPNO                   8
PROCNO                  1
Date_            20130201
Time                16.13
INSTRUM            avn400
PROBHD   5 mm PABBO BB/
PULPROG            zgflqn
TD                 131072
SOLVENT             CDCl3
NS                    256
DS                      4
SWH             75000.000 Hz
FIDRES           0.572205 Hz
AQ              0.8738633 sec
RG                 205.43
DW                  6.667 usec
DE                   6.50 usec
TE                  293.8 K
D1             1.00000000 sec
TD0                     1

======== CHANNEL f1 ========
SFO1          376.5547873 MHz
NUC1                  19F
P1                  13.50 usec
SI                  65536
SF            376.6112790 MHz
WDW                    EM
SSB                     0
LB                   0.30 Hz
GB                      0
PC                   1.00
```

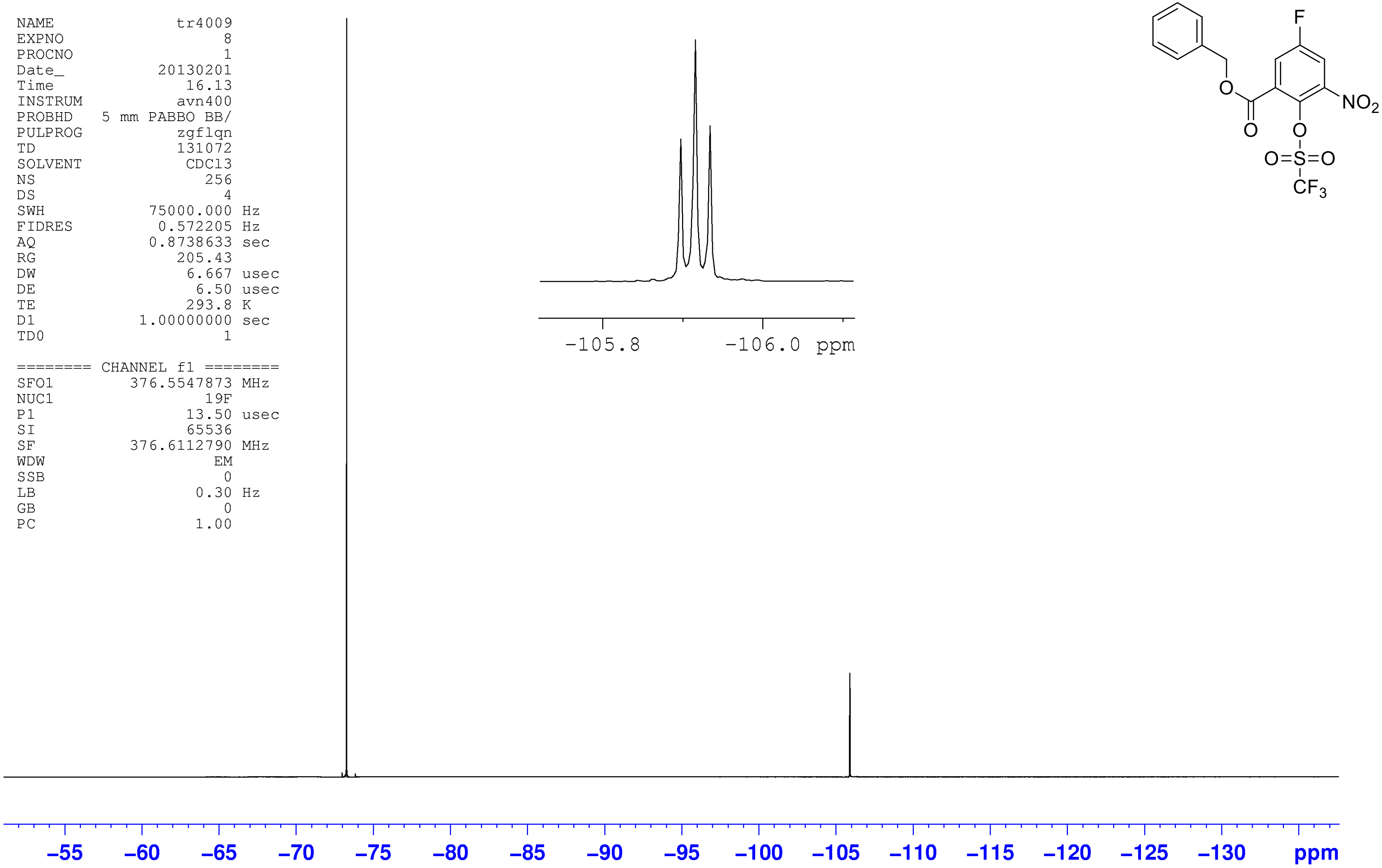

Benzyl 5-fluoro-2-{[(2*R*)-1-methoxy-1-oxopropan-2-yl]amino}-3-nitrobenzoate (*R*)-**25** ^{1}H NMR

```
NAME             tr4028
EXPNO                 1
PROCNO                1
Date_          20130107
Time               9.17
INSTRUM          avc500
PROBHD   5 mm CPDUL 13C
PULPROG            zg30
TD                65536
SOLVENT           CDCl3
NS                   16
DS                    2
SWH          10330.578 Hz
FIDRES        0.157632 Hz
AQ           3.1719923 sec
RG                 1.78
DW              48.400 usec
DE                6.00 usec
TE               298.0 K
D1          1.00000000 sec
TD0                   1

======== CHANNEL f1 ========
NUC1                 1H
P1               10.60 usec
PL1               6.00 dB
PL1W        0.95905519 W
SFO1       500.3030896 MHz
SI                32768
SF         500.3000231 MHz
WDW                  EM
SSB                   0
LB                0.30 Hz
GB                    0
PC                 1.00
```

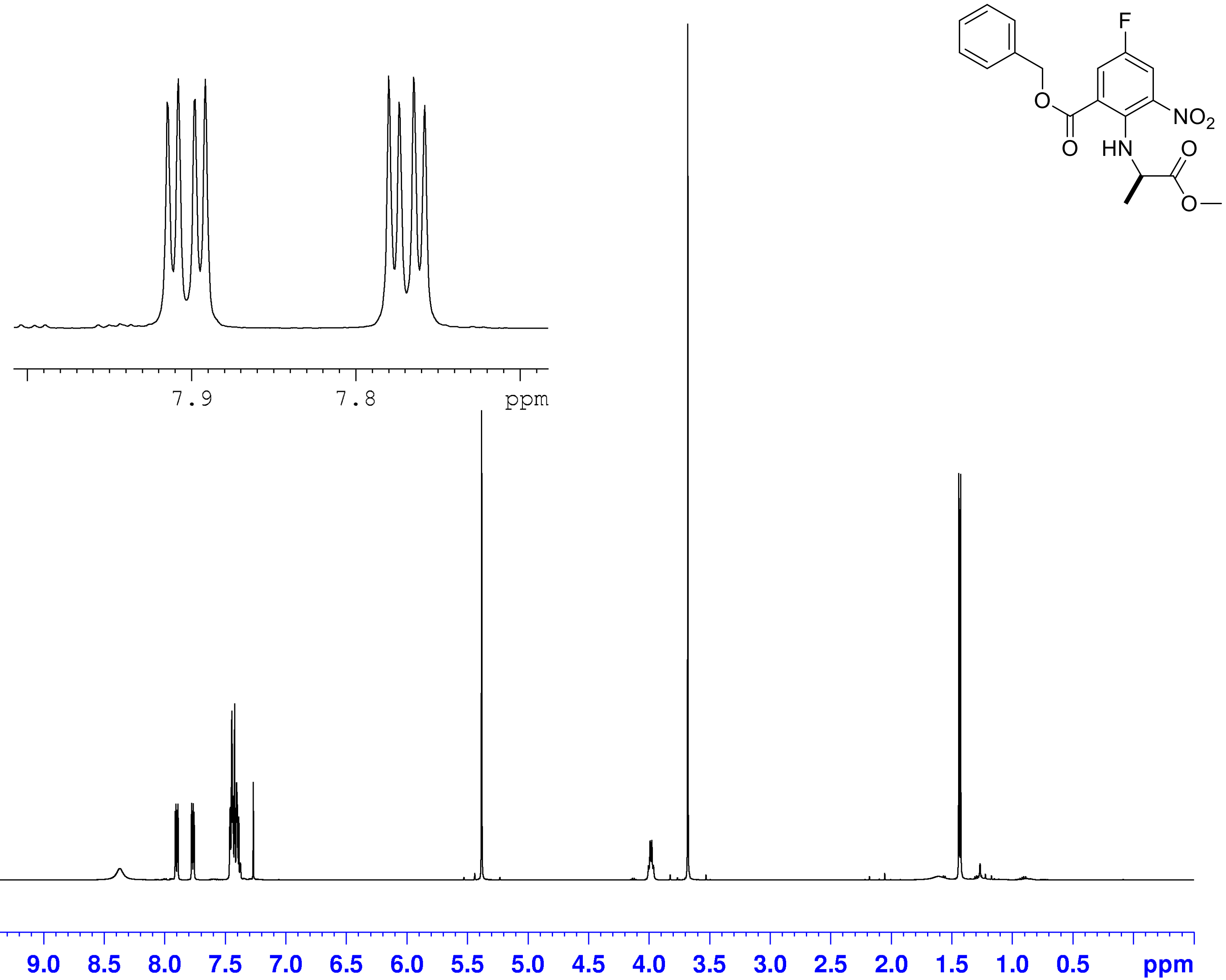

Benzyl 5-fluoro-2-{[(2*R*)-1-methoxy-1-oxopropan-2-yl]amino}-3-nitrobenzoate (*R*)-**25** ^{13}C NMR

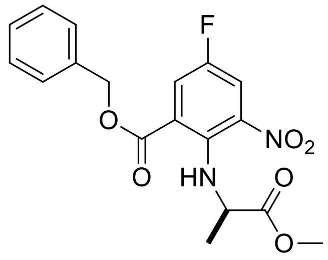


```
NAME              tr4028
EXPNO                  4
PROCNO                 1
Date_           20130107
Time                9.43
INSTRUM           avc500
PROBHD   5 mm CPDUL 13C
PULPROG          zgpg30
TD                 65536
SOLVENT            CDCl3
NS                   411
DS                     2
SWH            31250.000 Hz
FIDRES          0.476837 Hz
AQ             1.0486259 sec
RG                   912
DW                16.000 usec
DE                 20.00 usec
TE                 298.0 K
D1            2.00000000 sec
D11           0.03000000 sec
TD0                    1

======== CHANNEL f1 ========
NUC1                 13C
P1                 10.25 usec
PL1                 8.00 dB
PL1W          1.62029624 W
SFO1         125.8131151 MHz

======== CHANNEL f2 ========
CPDPRG2          waltz16
NUC2                  1H
PCPD2              80.00 usec
PL2                 6.00 dB
PL12               23.56 dB
PL13               29.56 dB
PL2W          0.95905519 W
PL12W         0.01682068 W
PL13W         0.00422516 W
SFO2         500.3020012 MHz
SI                 32768
SF           125.8005438 MHz
WDW                   EM
SSB                    0
LB                  1.00 Hz
GB                     0
PC                  1.40
```

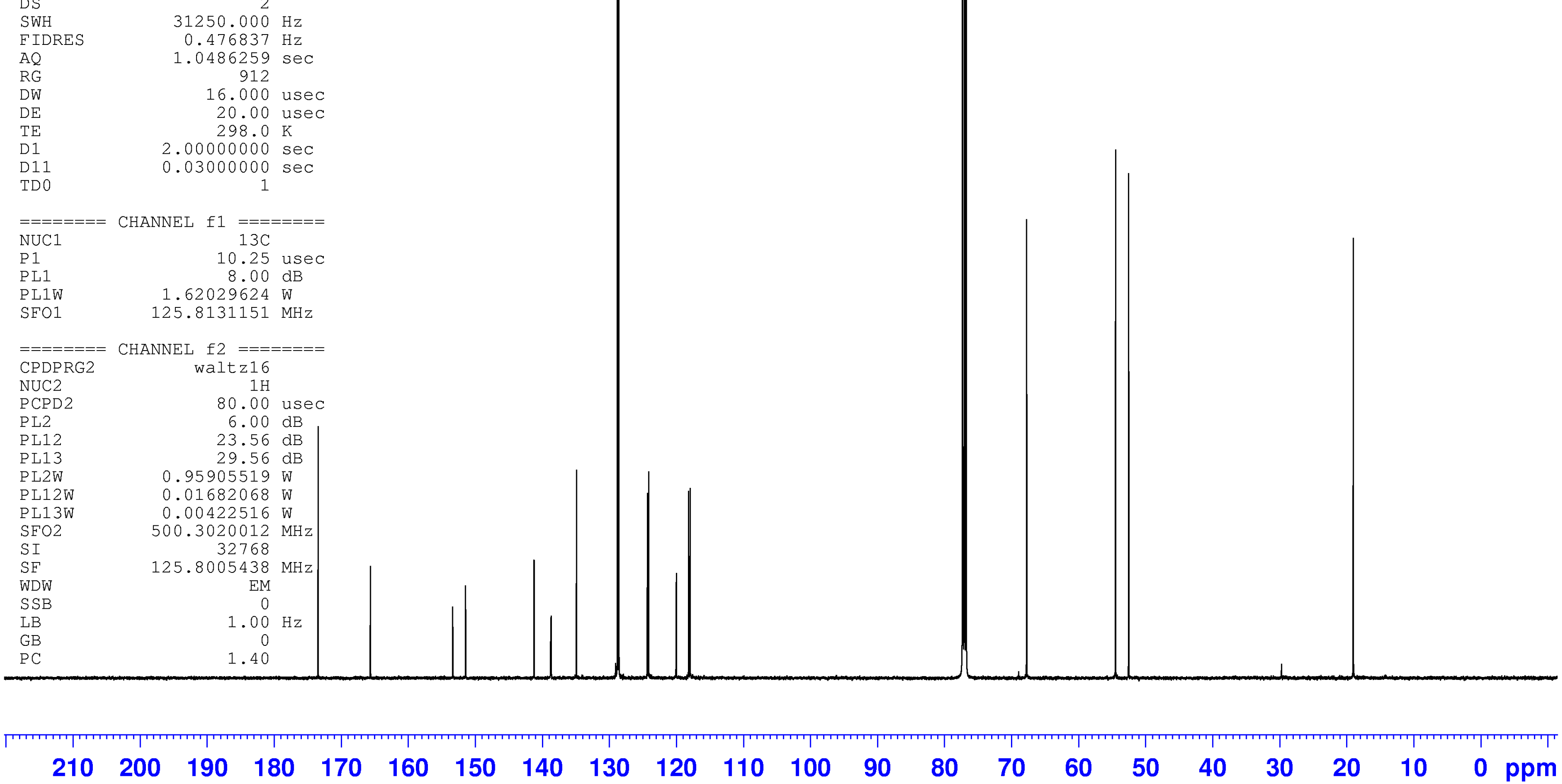

Benzyl 5-fluoro-2-{[(2*R*)-1-methoxy-1-oxopropan-2-yl]amino}-3-nitrobenzoate (*R*)-**25** ^{19}F NMR

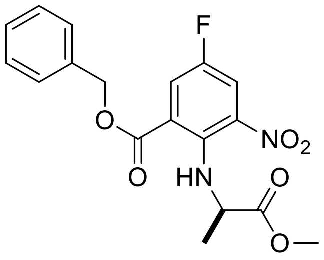

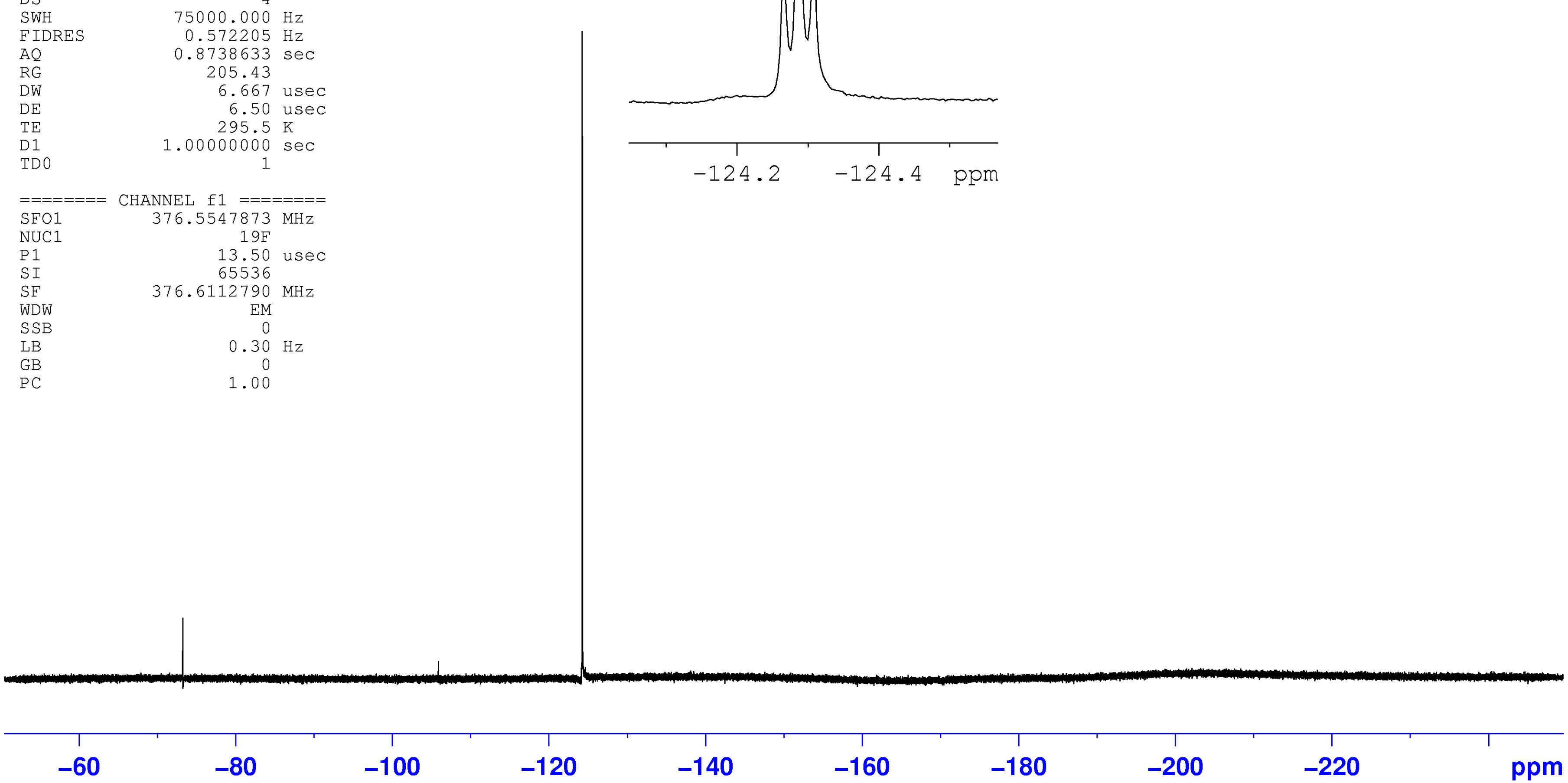

Benzyl (3*R*)-7-fluoro-3-methyl-2-oxo-1,2,3,4-tetrahydroquinoxaline-5-carboxylate (*R*)-**26** ^{1}H NMR

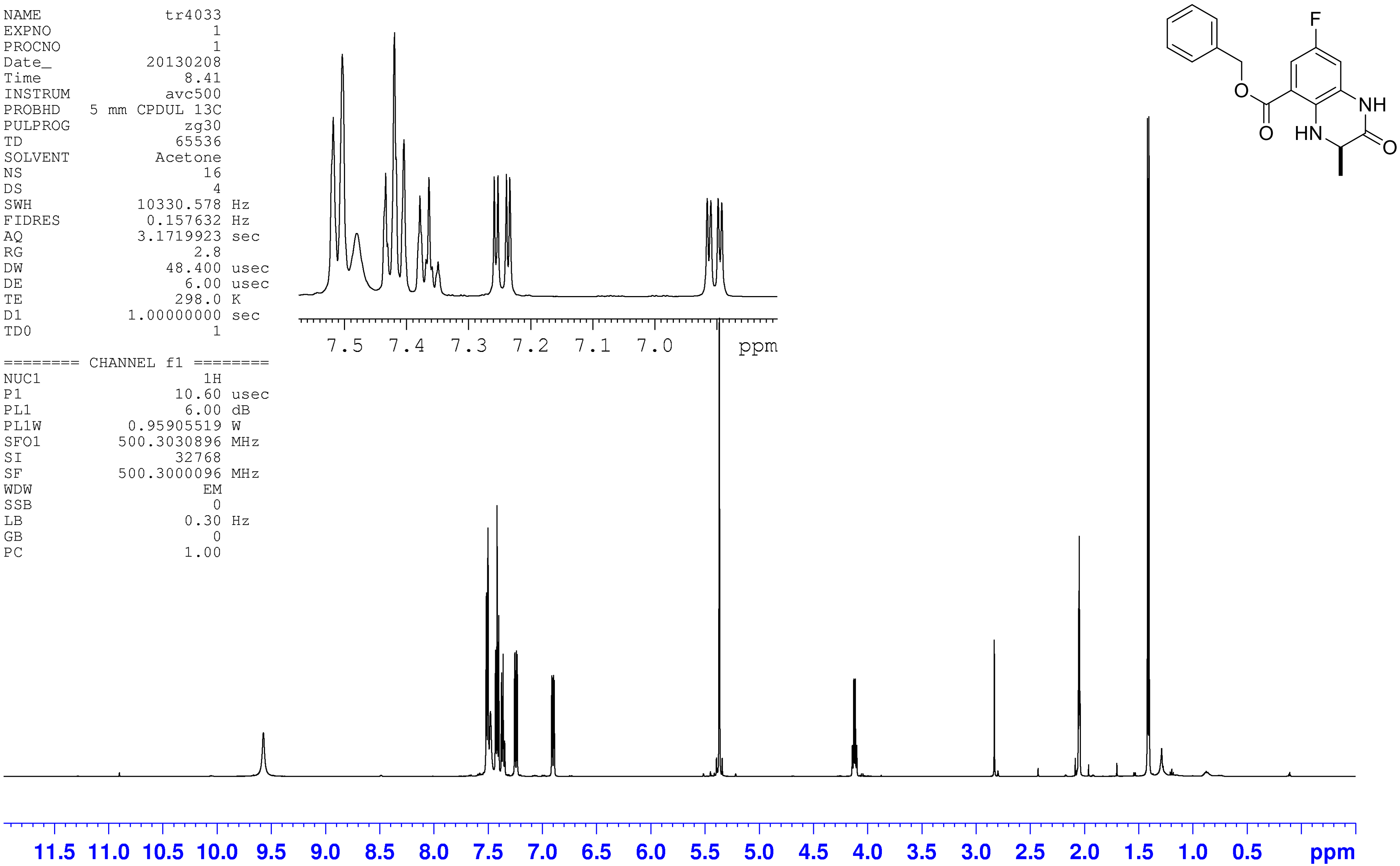

Benzyl (3*R*)-7-fluoro-3-methyl-2-oxo-1,2,3,4-tetrahydroquinoxaline-5-carboxylate (*R*)-**26** ^{13}C NMR

NAME	tr4033
EXPNO	4
PROCNO	1
Date_	20130208
Time	9.11
INSTRUM	avc500
PROBHD	5 mm CPDUL 13C
PULPROG	zgpg30
TD	65536
SOLVENT	Acetone
NS	256
DS	2
SWH	31250.000 Hz
FIDRES	0.476837 Hz
AQ	1.0486259 sec
RG	912
DW	16.000 usec
DE	20.00 usec
TE	298.0 K
D1	2.00000000 sec
D11	0.03000000 sec
TD0	1
======== CHANNEL f1 ========	
NUC1	13C
P1	10.25 usec
PL1	8.00 dB
PL1W	1.62029624 W
SFO1	125.8131151 MHz
======== CHANNEL f2 ========	
CPDPRG2	waltz16
NUC2	1H
PCPD2	80.00 usec
PL2	6.00 dB
PL12	23.56 dB
PL13	29.56 dB
PL2W	0.95905519 W
PL12W	0.01682068 W
PL13W	0.00422516 W
SFO2	500.3020012 MHz
SI	32768
SF	125.8004218 MHz
WDW	EM
SSB	0
LB	1.00 Hz
GB	0
PC	1.40

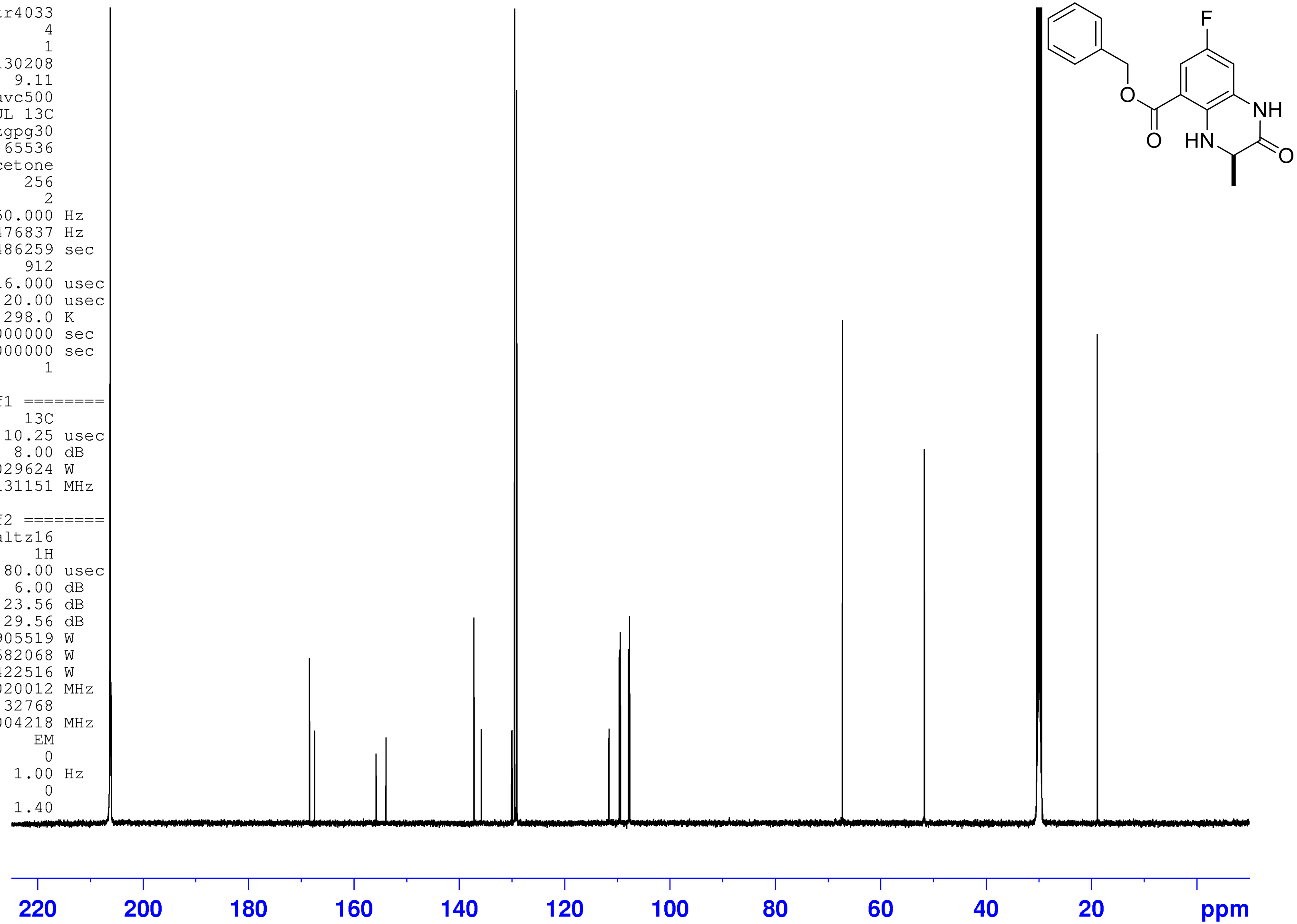

Benzyl (3*R*)-7-fluoro-3-methyl-2-oxo-1,2,3,4-tetrahydroquinoxaline-5-carboxylate (*R*)-**26** ^{19}F NMR

```
NAME              tr4033
EXPNO                  6
PROCNO                 1
Date_           20130208
Time               10.12
INSTRUM           avb500
PROBHD   5 mm PATXI 1H/
PULPROG           zgflqn
TD                131072
SOLVENT          Acetone
NS                    16
DS                     4
SWH           113636.367 Hz
FIDRES          0.866977 Hz
AQ             0.5767668 sec
RG                  2050
DW                 4.400 usec
DE                  6.50 usec
TE                 298.0 K
D1            1.00000000 sec
TD0                    1

======== CHANNEL f1 ========
NUC1                 19F
P1                 10.00 usec
PL1                 0.90 dB
SFO1         470.4041911 MHz
SI                 65536
SF           470.4512552 MHz
WDW                   EM
SSB                    0
LB                  0.30 Hz
GB                     0
PC                  1.00
```

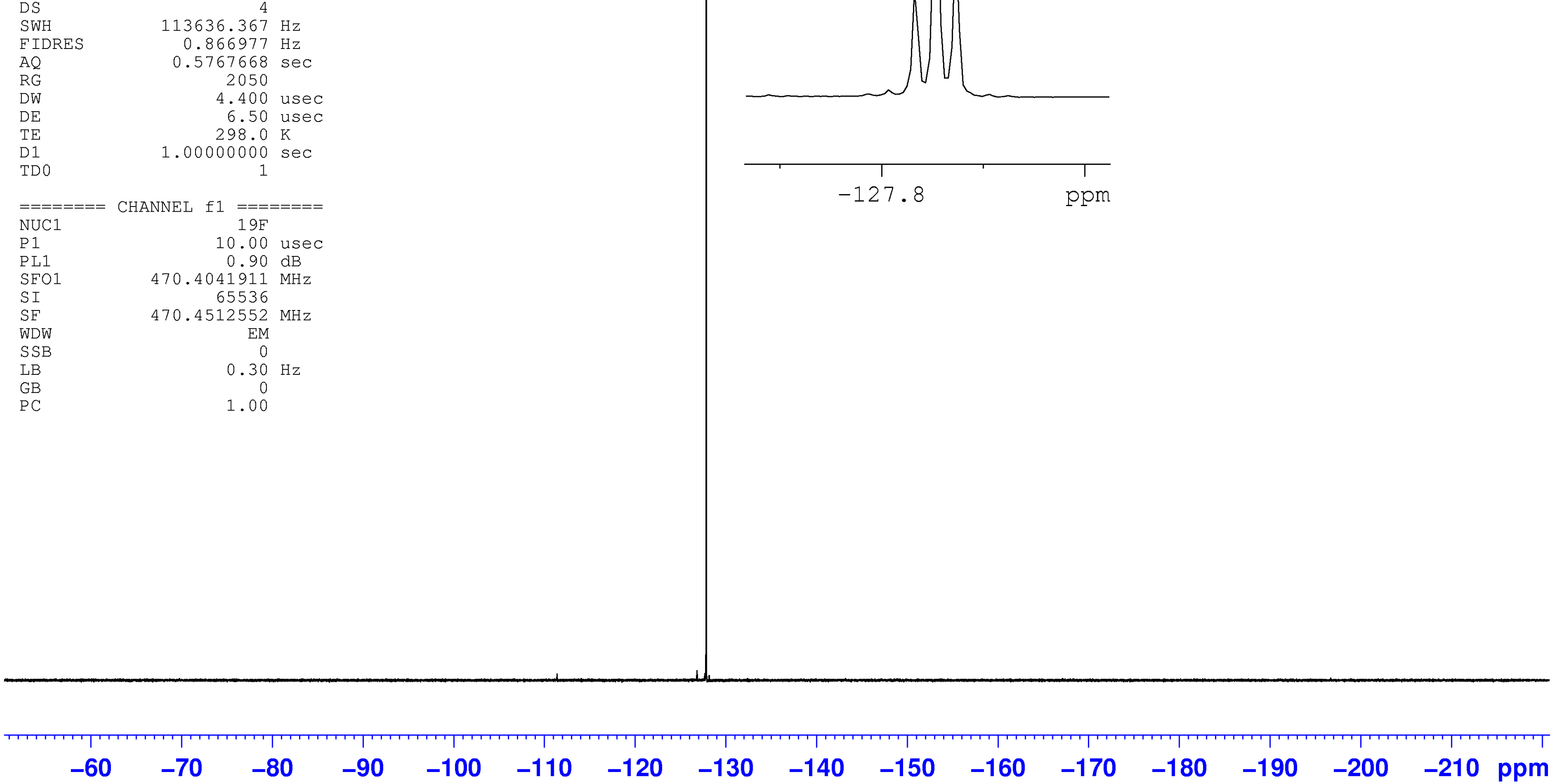

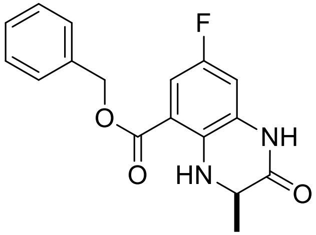

(3*R*)-7-Fluoro-3-methyl-2-oxo-1,2,3,4-tetrahydroquinoxaline-5-carboxylic acid (*R*)-**27** ^{1}H NMR

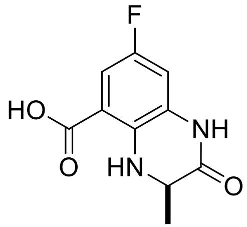


```
NAME          tr4038-2
EXPNO                1
PROCNO               1
Date_         20131121
Time             20.32
INSTRUM         avb500
PROBHD   5 mm PATXI 1H/
PULPROG           zg60
TD               65536
SOLVENT           DMSO
NS                  16
DS                   2
SWH          10330.578 Hz
FIDRES        0.157632 Hz
AQ           3.1719923 sec
RG                 362
DW              48.400 usec
DE                6.50 usec
TE               298.0 K
D1          1.00000000 sec
TD0                  1

======== CHANNEL f1 ========
SFO1       499.9825000 MHz
NUC1                1H
P1                9.00 usec
SI               32768
SF         499.9800000 MHz
WDW                 EM
SSB                  0
LB                0.30 Hz
GB                   0
PC                1.00
```

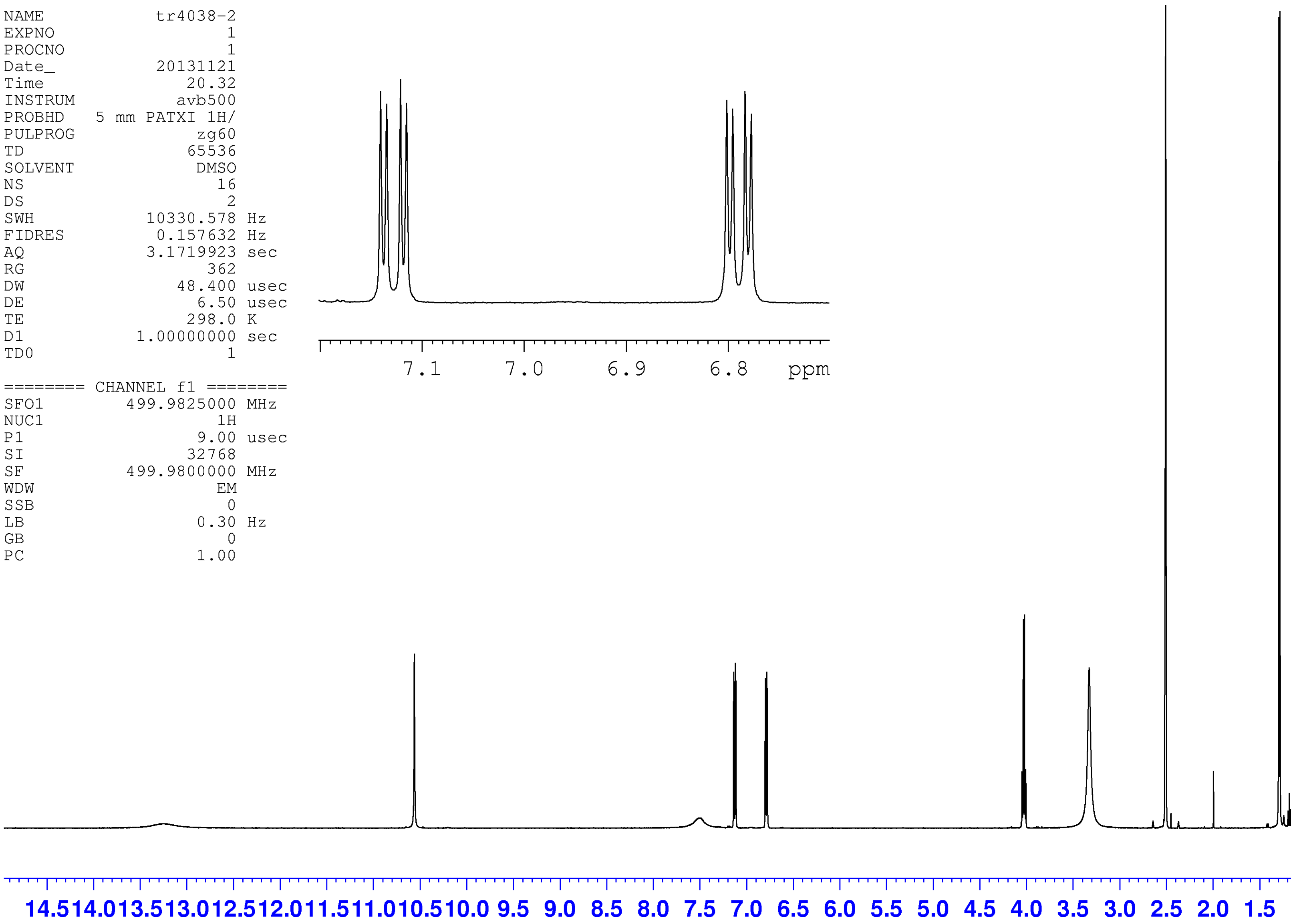

(3*R*)-7-Fluoro-3-methyl-2-oxo-1,2,3,4-tetrahydroquinoxaline-5-carboxylic acid (*R*)-**27** ^{13}C NMR

```
NAME              tr4038
EXPNO                  2
PROCNO                 1
Date_           20131021
Time                0.20
INSTRUM           drx500
PROBHD   5 mm PABBO BB/
PULPROG          zgpg30
TD                65536
SOLVENT             DMSO
NS                  3072
DS                     4
SWH            30303.031 Hz
FIDRES          0.462388 Hz
AQ             1.0814105 sec
RG                5160.6
DW                16.500 usec
DE                 11.00 usec
TE                 298.7 K
D1            2.00000000 sec
d11           0.03000000 sec
DELTA         1.89999998 sec
TD0                    1

======== CHANNEL f1 ========
NUC1                 13C
P1                  6.80 usec
PL1                 5.00 dB
SFO1         125.7703148 MHz

======== CHANNEL f2 ========
CPDPRG2          waltz16
NUC2                  1H
PCPD2             100.00 usec
PL2                 0.00 dB
PL12               19.00 dB
PL13               23.00 dB
SFO2         500.1320005 MHz
SI                 32768
SF           125.7578458 MHz
WDW                   EM
SSB                    0
LB                  1.00 Hz
GB                     0
PC                  1.40
```

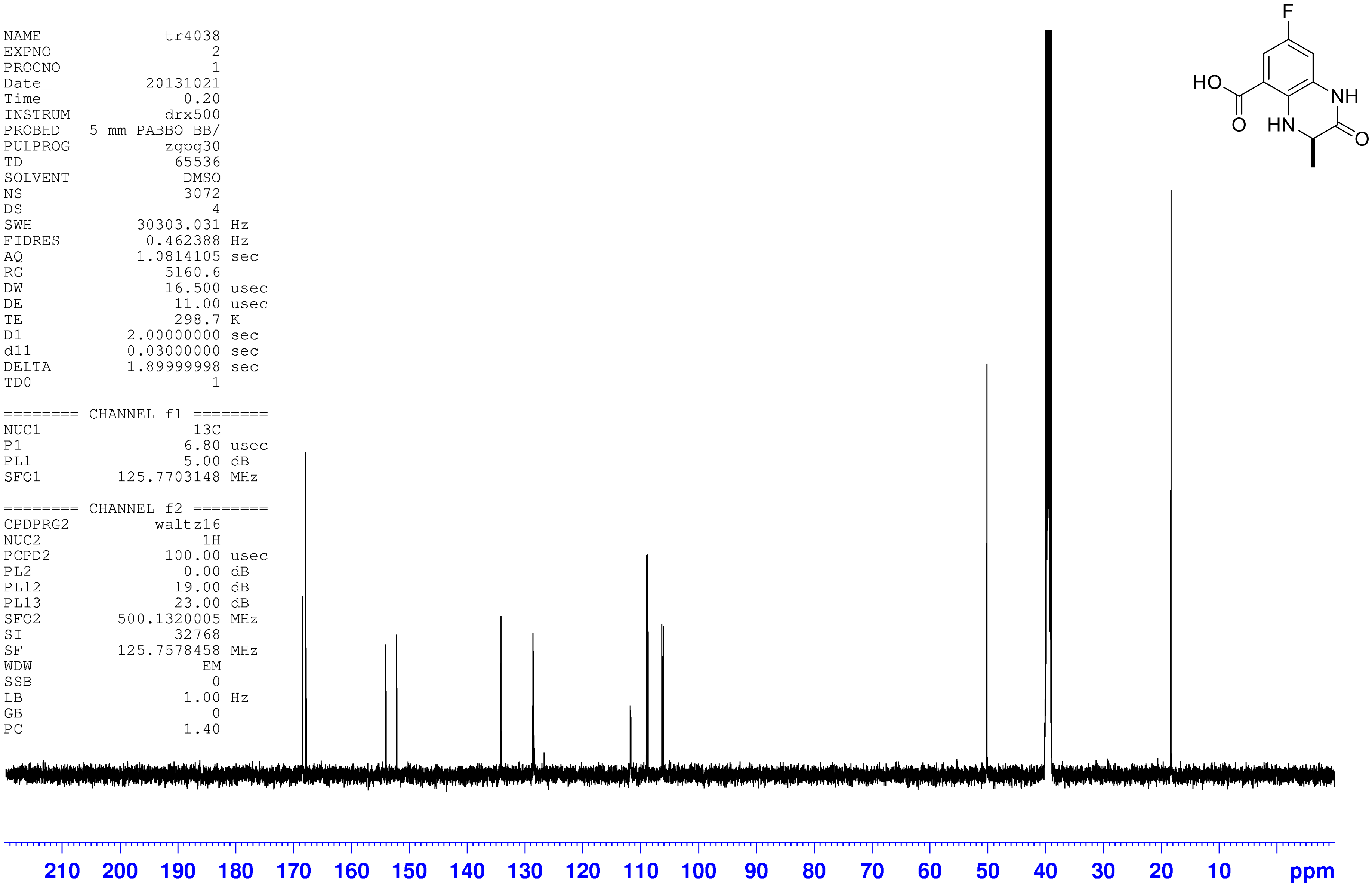

(3*R*)-7-Fluoro-3-methyl-2-oxo-1,2,3,4-tetrahydroquinoxaline-5-carboxylic acid (*R*)-**27** ^{19}F NMR

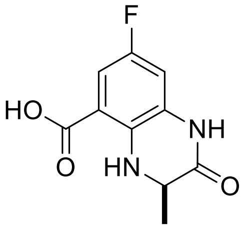


```
NAME              tr4038-2
EXPNO                    2
PROCNO                   1
Date_             20131121
Time                 20.37
INSTRUM             avb500
PROBHD    5 mm PATXI 1H/
PULPROG            zgflqn
TD                  131072
SOLVENT               DMSO
NS                     128
DS                       4
SWH             113636.367 Hz
FIDRES            0.866977 Hz
AQ               0.5767668 sec
RG                    2050
DW                   4.400 usec
DE                    6.50 usec
TE                   298.0 K
D1              1.00000000 sec
TD0                      1

======== CHANNEL f1 ========
SFO1           470.4041911 MHz
NUC1                   19F
P1                   10.00 usec
SI                   65536
SF             470.4512360 MHz
WDW                     EM
SSB                      0
LB                    0.30 Hz
GB                       0
PC                    1.00
```

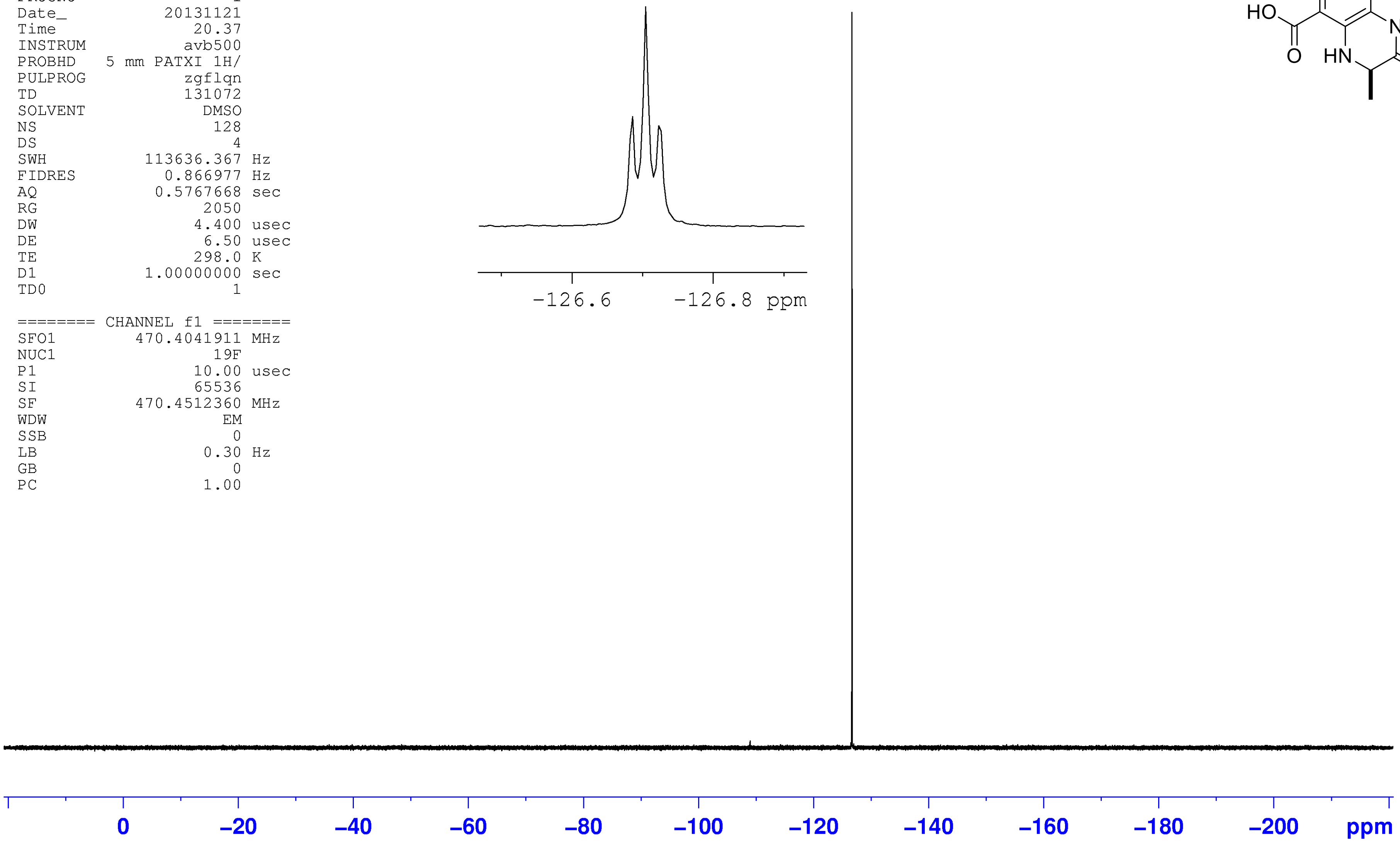

(3*R*)-*N*-[3-(3,4-Dihydroquinolin-1(2*H*)-yl)propyl]-3-methyl-2-oxo-1,2,3,4-tetrahydroquinoxaline-5-carboxamide (*R*)-**1** ^{1}H NMR

NAME	tr1080
EXPNO	1
PROCNO	1
Date_	20130902
Time	10.50
INSTRUM	av400
PROBHD	5 mm QNP 1H/13
PULPROG	zg60
TD	65536
SOLVENT	Acetone
NS	16
DS	2
SWH	8278.146 Hz
FIDRES	0.126314 Hz
AQ	3.9584243 sec
RG	362
DW	60.400 usec
DE	7.50 usec
TE	300.0 K
D1	1.00000000 sec
======== CHANNEL f1 ========	
NUC1	1H
P1	10.10 usec
PL1	0.00 dB
SFO1	400.2024714 MHz
SI	32768
SF	400.2000000 MHz
WDW	EM
SSB	0
LB	0.30 Hz
GB	0
PC	1.00

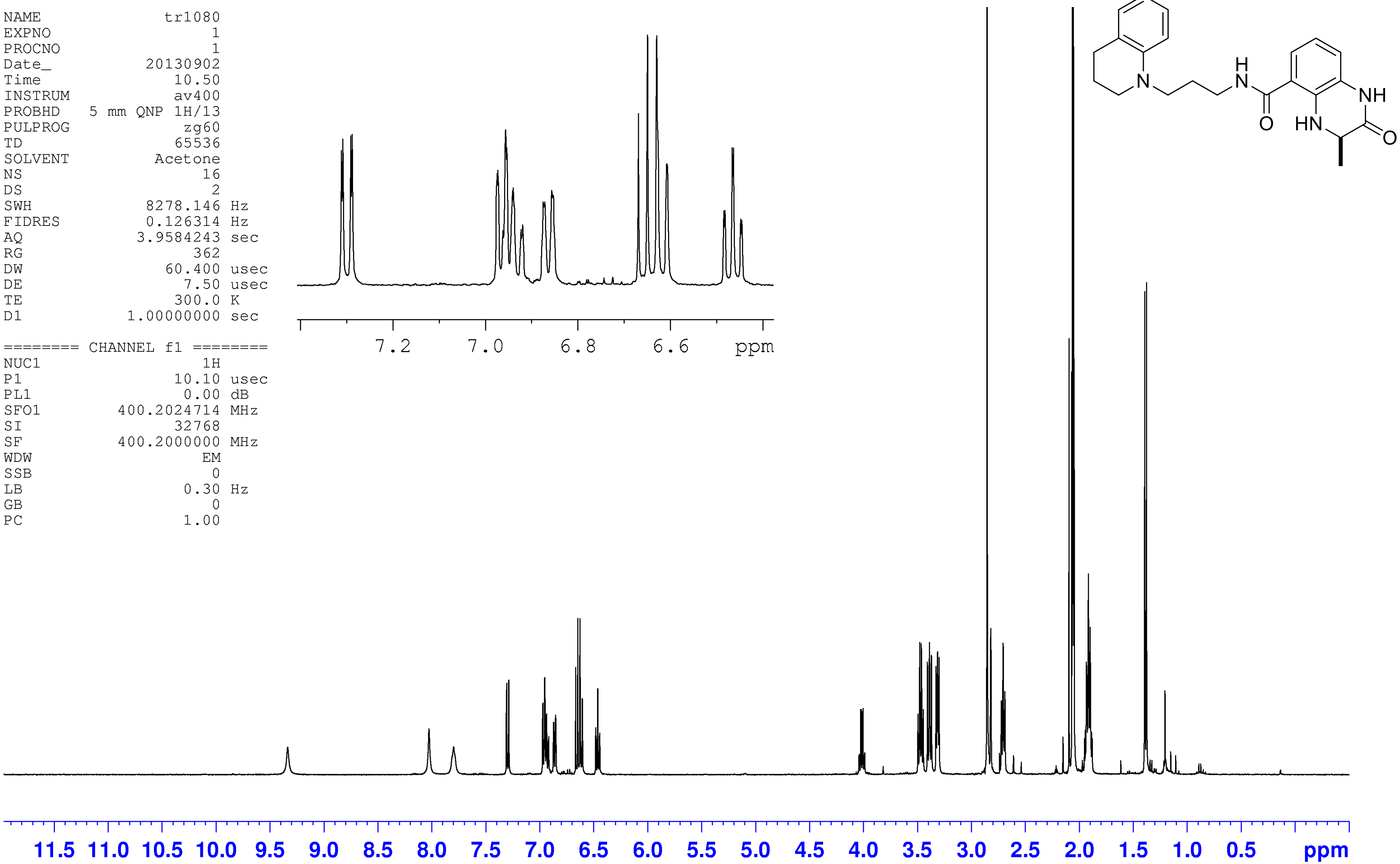

(3*R*)-*N*-[3-(3,4-Dihydroquinolin-1(2*H*)-yl)propyl]-3-methyl-2-oxo-1,2,3,4-tetrahydroquinoxaline-5-carboxamide (*R*)-**1** ^{13}C NMR

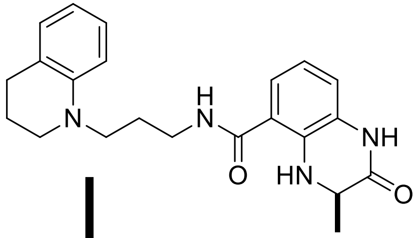


```
NAME              tr1080
EXPNO                  4
PROCNO                 1
Date_           20130904
Time               16.08
INSTRUM           avc500
PROBHD   5 mm CPDUL 13C
PULPROG          zgpg30
TD                 65536
SOLVENT          Acetone
NS                  1024
DS                     2
SWH            31250.000 Hz
FIDRES          0.476837 Hz
AQ             1.0486259 sec
RG                   912
DW                16.000 usec
DE                 20.00 usec
TE                 298.0 K
D1            2.00000000 sec
D11           0.03000000 sec
TD0                    1

======== CHANNEL f1 ========
NUC1                 13C
P1                 10.25 usec
PL1                 8.00 dB
PL1W          1.62029624 W
SFO1         125.8131151 MHz

======== CHANNEL f2 ========
CPDPRG2          waltz16
NUC2                  1H
PCPD2              80.00 usec
PL2                 6.00 dB
PL12               23.56 dB
PL13               29.56 dB
PL2W          0.95905519 W
PL12W         0.01682068 W
PL13W         0.00422516 W
SFO2         500.3020012 MHz
SI                 32768
SF           125.8004218 MHz
WDW                   EM
SSB                    0
LB                  1.00 Hz
GB                     0
PC                  1.40
```

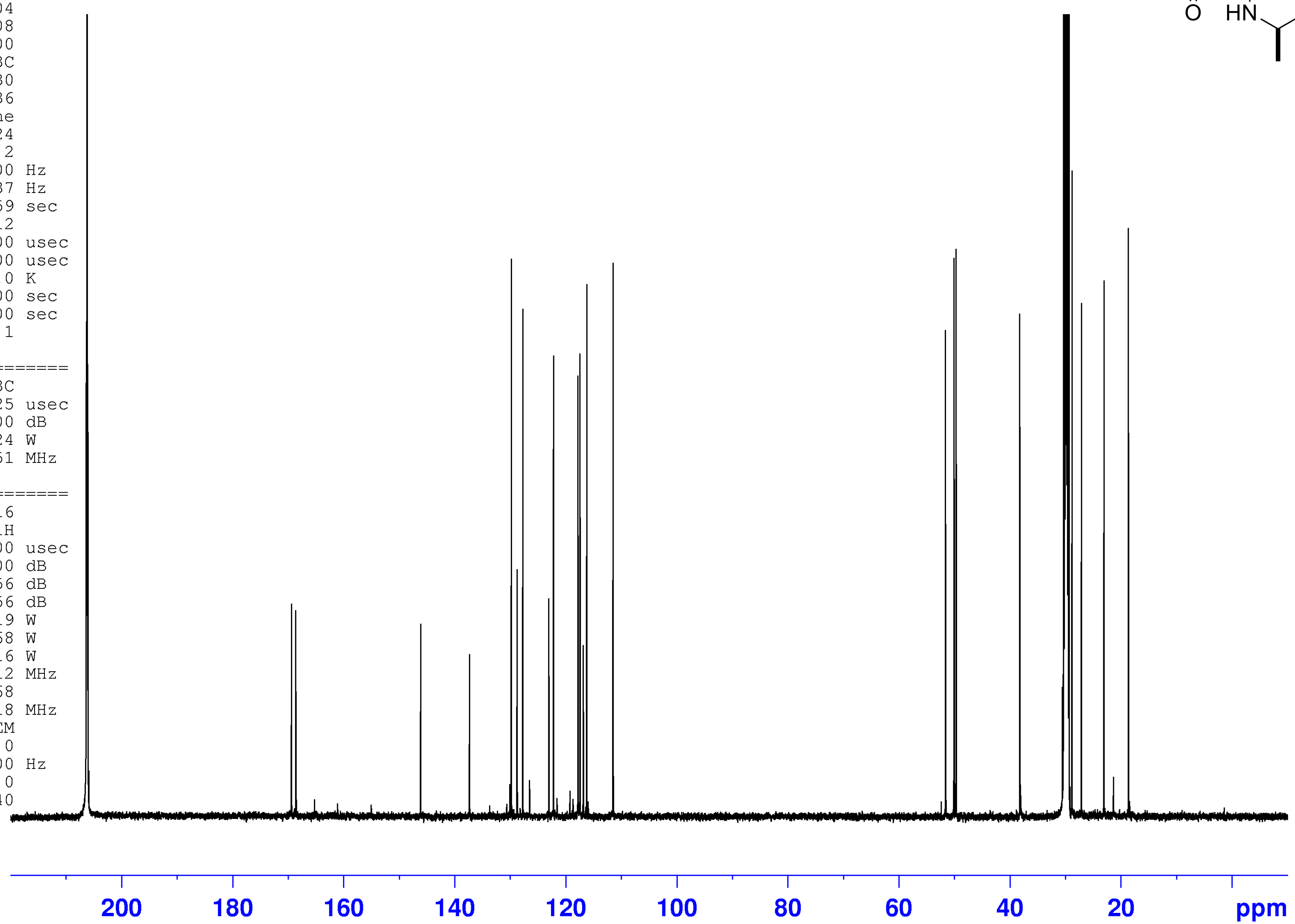

(3*R*)-*N*-[3-(6-Methoxy-3,4-dihydroquinolin-1(2*H*)-yl)propyl]-3-methyl-2-oxo-1,2,3,4-tetrahydroquinoxaline-5-carboxamide (*R*)-**6** ^{1}H-NMR

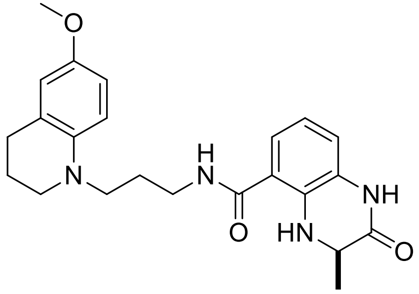


```
NAME             tr3029
EXPNO                 1
PROCNO                1
Date_          20130821
Time              12.15
INSTRUM           av400
PROBHD   5 mm QNP 1H/13
PULPROG            zg60
TD                65536
SOLVENT         Acetone
NS                   16
DS                    2
SWH         8278.146 Hz
FIDRES      0.126314 Hz
AQ         3.9584243 sec
RG                  512
DW           60.400 usec
DE             7.50 usec
TE             300.0 K
D1        1.00000000 sec

======== CHANNEL f1 ========
NUC1                 1H
P1            10.10 usec
PL1            0.00 dB
SFO1     400.2024714 MHz
SI                32768
SF       400.2000000 MHz
WDW                  EM
SSB                   0
LB              0.30 Hz
GB                    0
PC                 1.00
```

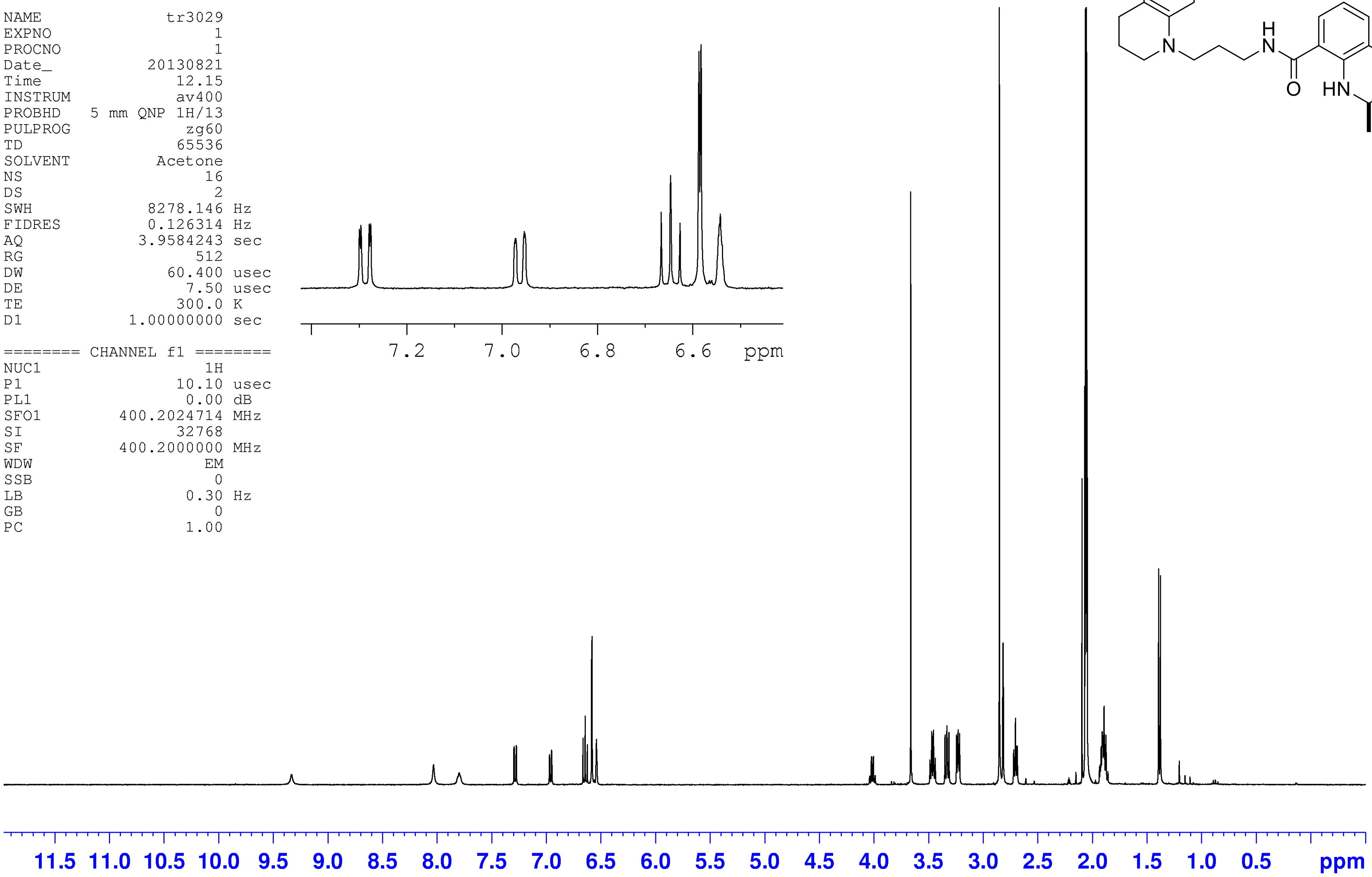

(3*R*)-*N*-[3-(6-Methoxy-3,4-dihydroquinolin-1(2*H*)-yl)propyl]-3-methyl-2-oxo-1,2,3,4-tetrahydroquinoxaline-5-carboxamide (*R*)-**6** ^{13}C-NMR

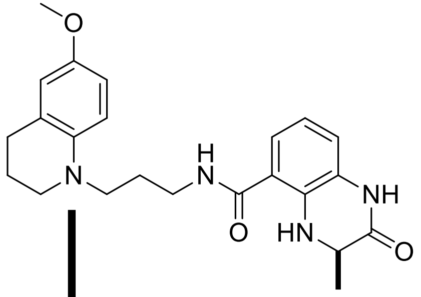


```
NAME               tr3029
EXPNO                   2
PROCNO                  1
Date_            20130824
Time                16.40
INSTRUM            drx500
PROBHD   5 mm PABBO BB/
PULPROG           zgpg30
TD                  65536
SOLVENT           Acetone
NS                   1536
DS                      4
SWH             30303.031 Hz
FIDRES           0.462388 Hz
AQ              1.0814105 sec
RG                 3649.1
DW                 16.500 usec
DE                  11.00 usec
TE                  297.7 K
D1             2.00000000 sec
d11            0.03000000 sec
DELTA          1.89999998 sec
TD0                     1

======== CHANNEL f1 ========
NUC1                  13C
P1                   6.80 usec
PL1                  5.00 dB
SFO1          125.7703148 MHz

======== CHANNEL f2 ========
CPDPRG2           waltz16
NUC2                   1H
PCPD2              100.00 usec
PL2                  0.00 dB
PL12                19.00 dB
PL13                23.00 dB
SFO2          500.1320005 MHz
SI                  32768
SF            125.7576258 MHz
WDW                    EM
SSB                     0
LB                   1.00 Hz
GB                      0
PC                   1.40
```

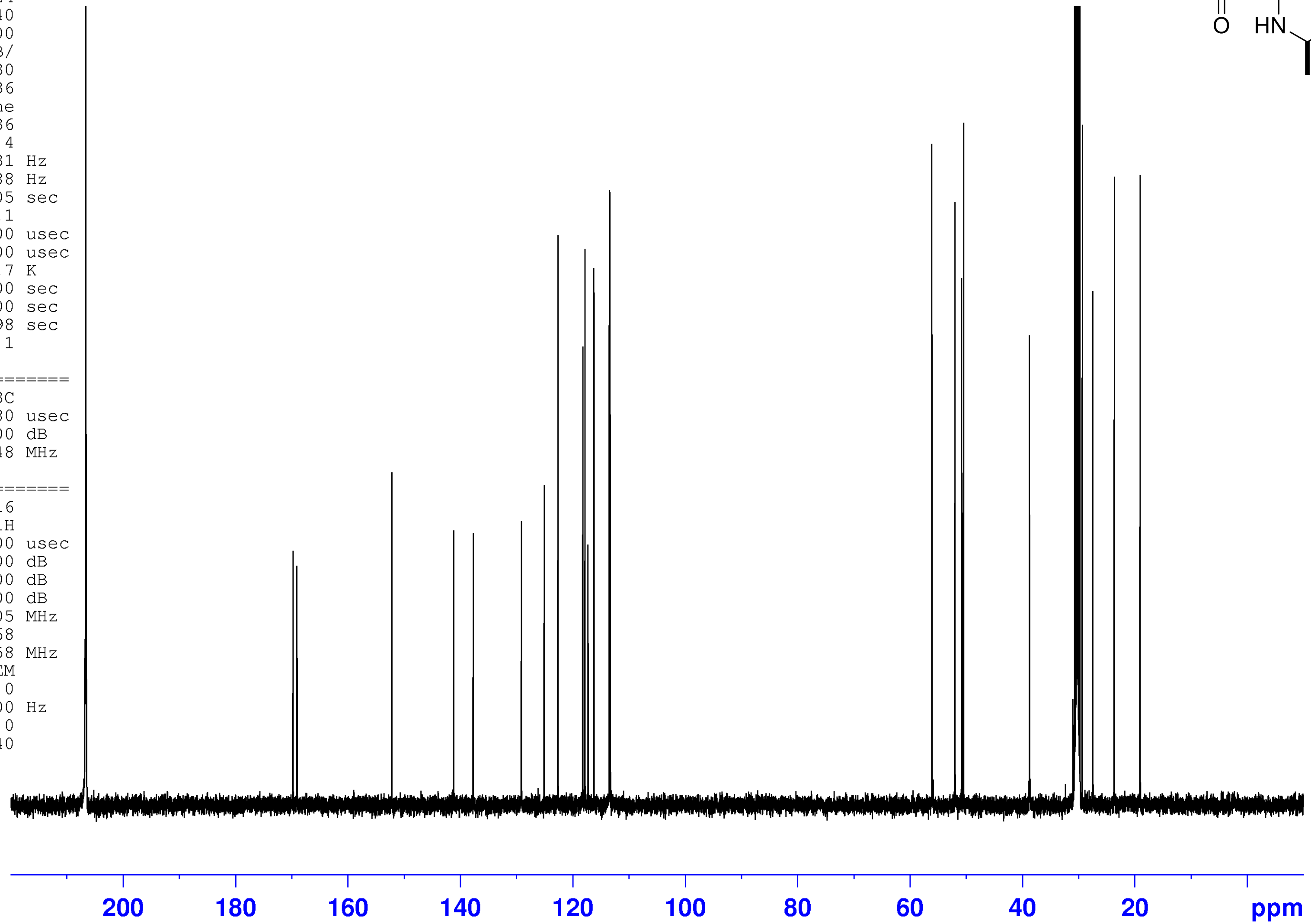

(3*R*)-*N*-[3-(7-Methoxy-3,4-dihydroquinolin-1(2*H*)-yl)propyl]-3-methyl-2-oxo-1,2,3,4-tetrahydroquinoxaline-5-carboxamide (*R*)-**2** ^{1}H NMR

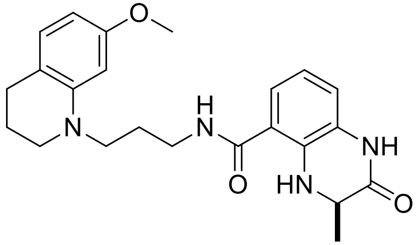

NAME	tr3030	
EXPNO	1	
PROCNO	1	
Date_	20130904	
Time	14.17	
INSTRUM	avc500	
PROBHD	5 mm CPDUL 13C	
PULPROG	zg30	
TD	65536	
SOLVENT	Acetone	
NS	16	
DS	4	
SWH	10330.578	Hz
FIDRES	0.157632	Hz
AQ	3.1719923	sec
RG	2.8	
DW	48.400	usec
DE	6.00	usec
TE	298.0	K
D1	1.00000000	sec
TD0	1	

======== CHANNEL f1 ========

NUC1	1H	
P1	10.60	usec
PL1	6.00	dB
PL1W	0.95905519	W
SFO1	500.3030896	MHz
SI	32768	
SF	500.3000096	MHz
WDW	EM	
SSB	0	
LB	0.30	Hz
GB	0	
PC	1.00	

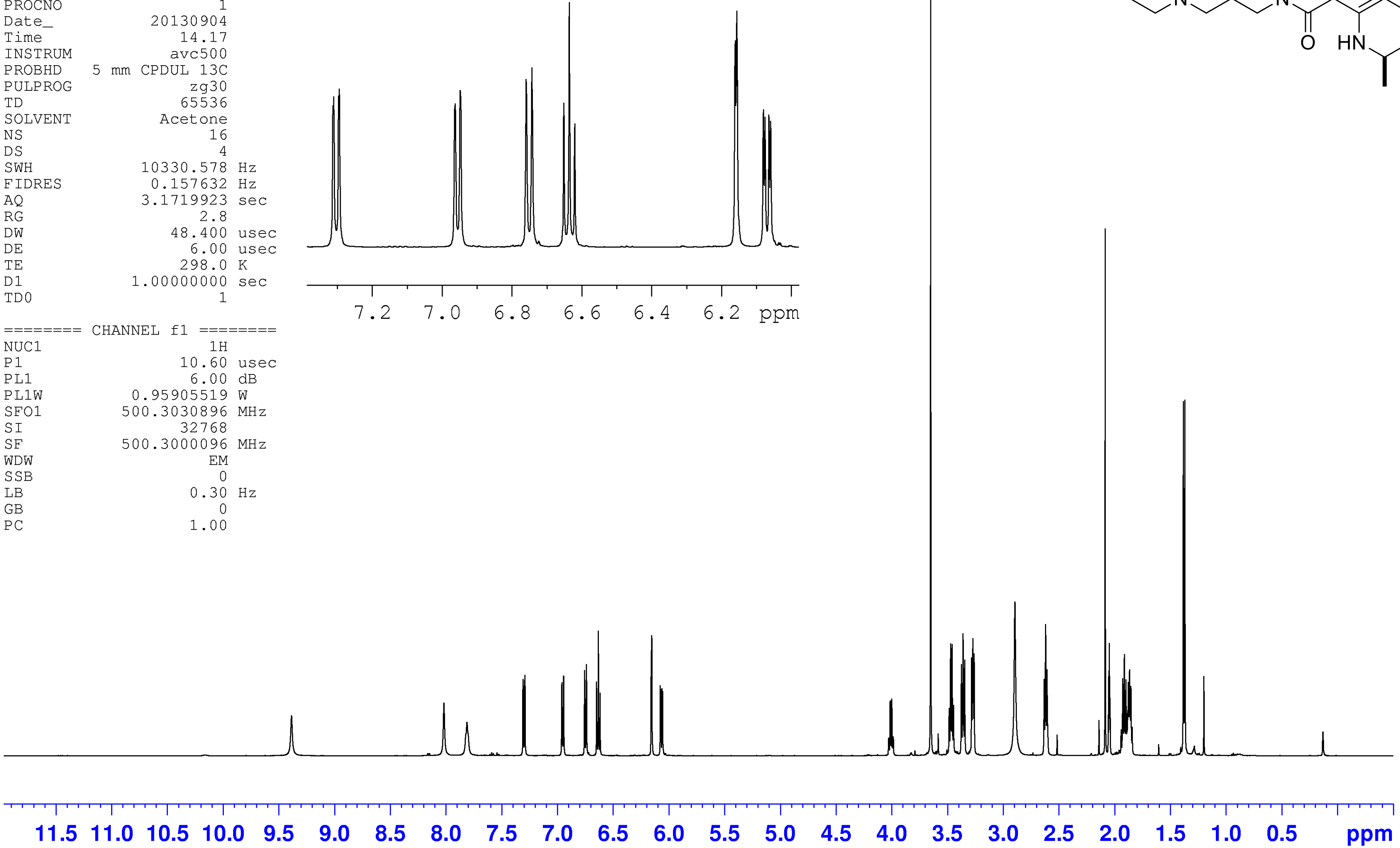

(3*R*)-*N*-[3-(7-Methoxy-3,4-dihydroquinolin-1(2*H*)-yl)propyl]-3-methyl-2-oxo-1,2,3,4-tetrahydroquinoxaline-5-carboxamide (*R*)-**2** ^{13}C NMR

NAME	tr3030
EXPNO	4
PROCNO	1
Date_	20130904
Time	14.55
INSTRUM	avc500
PROBHD	5 mm CPDUL 13C
PULPROG	zgpg30
TD	65536
SOLVENT	Acetone
NS	347
DS	2
SWH	31250.000 Hz
FIDRES	0.476837 Hz
AQ	1.0486259 sec
RG	912
DW	16.000 usec
DE	20.00 usec
TE	298.0 K
D1	2.00000000 sec
D11	0.03000000 sec
TD0	1

======== CHANNEL f1 ========

NUC1	13C
P1	10.25 usec
PL1	8.00 dB
PL1W	1.62029624 W
SFO1	125.8131151 MHz

======== CHANNEL f2 ========

CPDPRG2	waltz16
NUC2	1H
PCPD2	80.00 usec
PL2	6.00 dB
PL12	23.56 dB
PL13	29.56 dB
PL2W	0.95905519 W
PL12W	0.01682068 W
PL13W	0.00422516 W
SFO2	500.3020012 MHz
SI	32768
SF	125.8004218 MHz
WDW	EM
SSB	0
LB	1.00 Hz
GB	0
PC	1.40

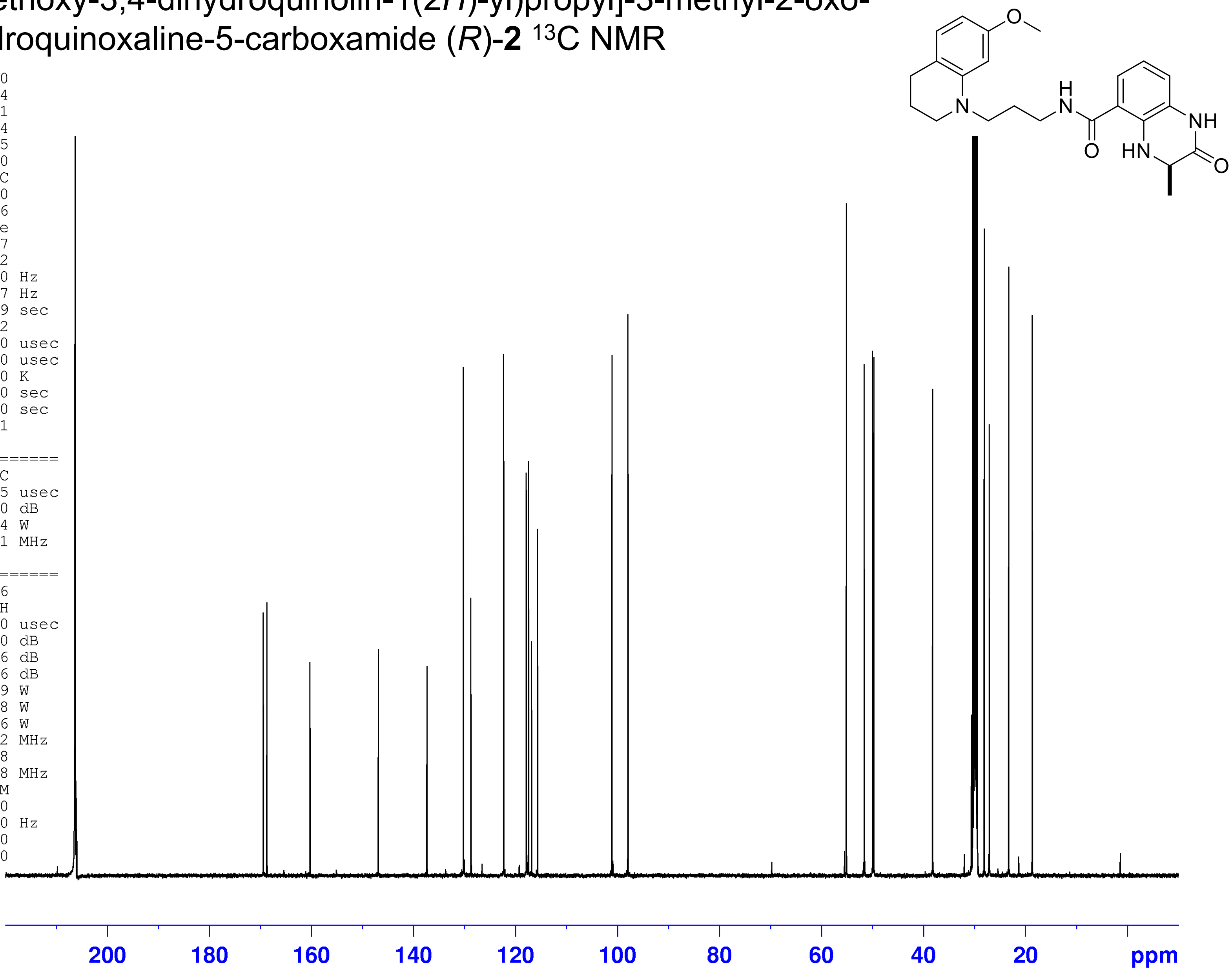

(3*R*)-*N*-[3-(2,3-Dihydro-4*H*-1,4-benzoxazin-4-yl)propyl]-3-methyl-2-oxo-1,2,3,4-tetrahydroquinoxaline-5-carboxamide (*R*)-**5** ^{1}H NMR

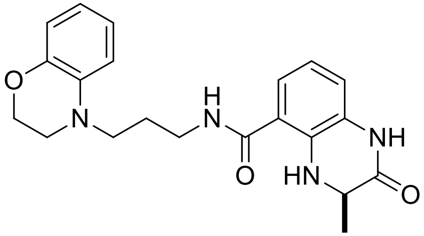

NAME	tr3031
EXPNO	1
PROCNO	1
Date_	20130923
Time	18.49
INSTRUM	spect
PROBHD	5 mm QNP 1H/13
PULPROG	zg30
TD	65536
SOLVENT	Acetone
NS	16
DS	2
SWH	10000.000 Hz
FIDRES	0.152588 Hz
AQ	3.2768500 sec
RG	568.78
DW	50.000 usec
DE	6.50 usec
TE	294.1 K
D1	1.00000000 sec
TD0	1

======== CHANNEL f1 ========

SFO1	400.2024714 MHz
NUC1	1H
P1	12.23 usec
SI	65536
SF	400.2000102 MHz
WDW	EM
SSB	0
LB	0.30 Hz
GB	0
PC	1.00

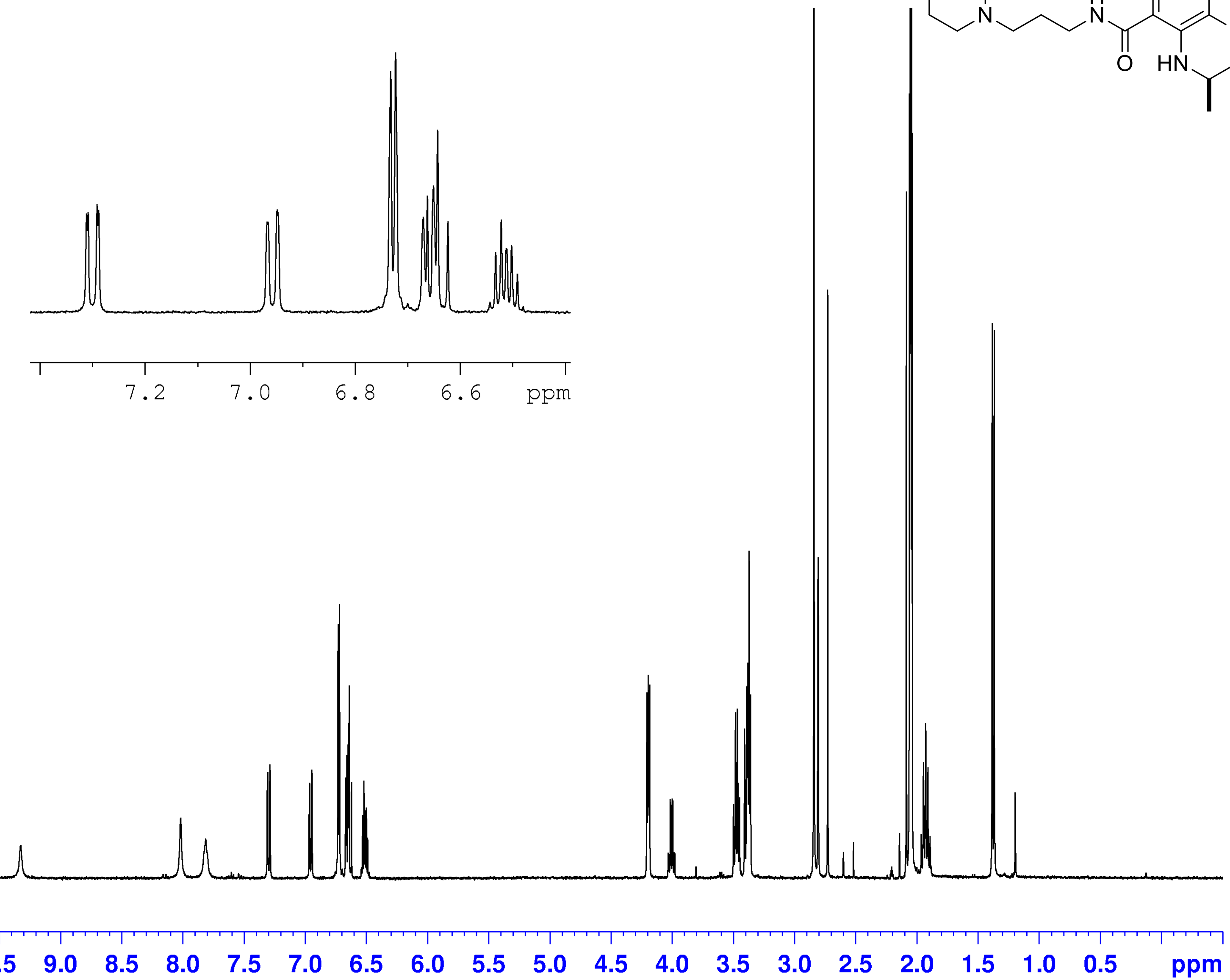

(3*R*)-*N*-[3-(2,3-Dihydro-4*H*-1,4-benzoxazin-4-yl)propyl]-3-methyl-2-oxo-1,2,3,4-tetrahydroquinoxaline-5-carboxamide (*R*)-**5** ^{13}C NMR

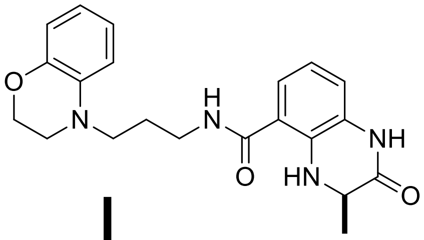


```
NAME              tr3031
EXPNO                  4
PROCNO                 1
Date_           20130925
Time               15.34
INSTRUM           avc500
PROBHD   5 mm CPDUL 13C
PULPROG          zgpg30
TD                 65536
SOLVENT          Acetone
NS                   512
DS                     2
SWH            31250.000 Hz
FIDRES          0.476837 Hz
AQ             1.0486259 sec
RG                   912
DW                16.000 usec
DE                 18.00 usec
TE                 298.0 K
D1            2.00000000 sec
D11           0.03000000 sec
TD0                    1

======== CHANNEL f1 ========
SFO1         125.8131151 MHz
NUC1                 13C
P1                 10.00 usec
SI                 32768
SF           125.8005350 MHz
WDW                   EM
SSB                    0
LB                  1.00 Hz
GB                     0
PC                  1.40
```

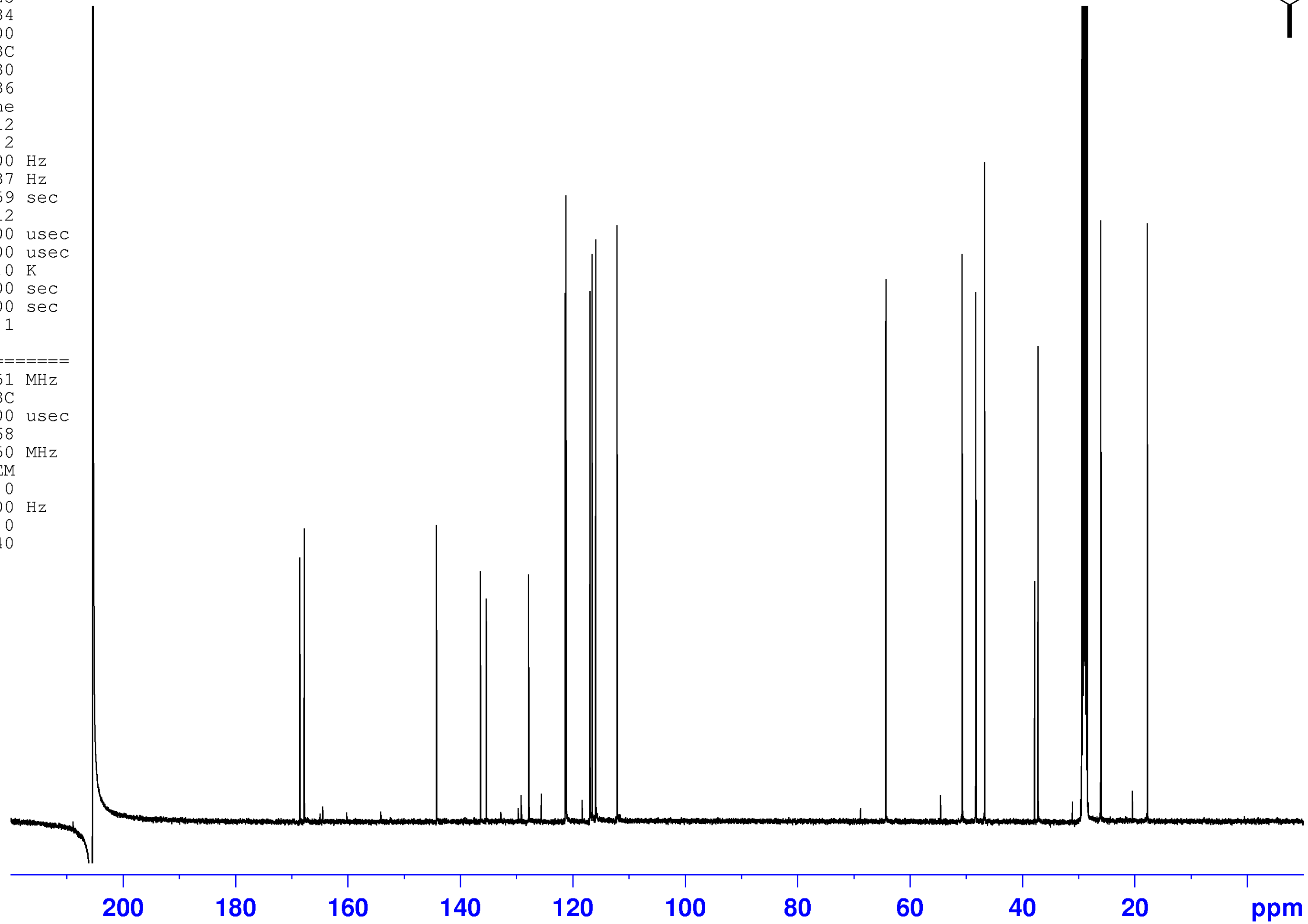

(3*R*)-*N*-[3-(7-methoxy-3,4-dihydroquinolin-1(2*H*)-yl)propyl]-6-fluoro-3-methyl-2-oxo-1,2,3,4-tetrahydroquinoxaline-5-carboxamide (*R*)-**8** ^{1}H NMR

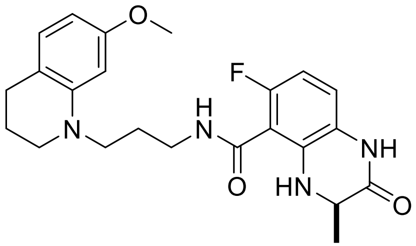


```
NAME              tr4039
EXPNO                  1
PROCNO                 1
Date_           20130114
Time               16.48
INSTRUM           avc500
PROBHD   5 mm CPDUL 13C
PULPROG             zg30
TD                 65536
SOLVENT          Acetone
NS                    16
DS                     2
SWH            10330.578 Hz
FIDRES          0.157632 Hz
AQ             3.1719923 sec
RG                   2.8
DW                48.400 usec
DE                  6.00 usec
TE                 298.0 K
D1            1.00000000 sec
TD0                    1

======== CHANNEL f1 ========
NUC1                  1H
P1                 10.60 usec
PL1                 6.00 dB
PL1W          0.95905519 W
SFO1         500.3030896 MHz
SI                 32768
SF           500.3000000 MHz
WDW                   EM
SSB                    0
LB                  0.30 Hz
GB                     0
PC                  1.00
```

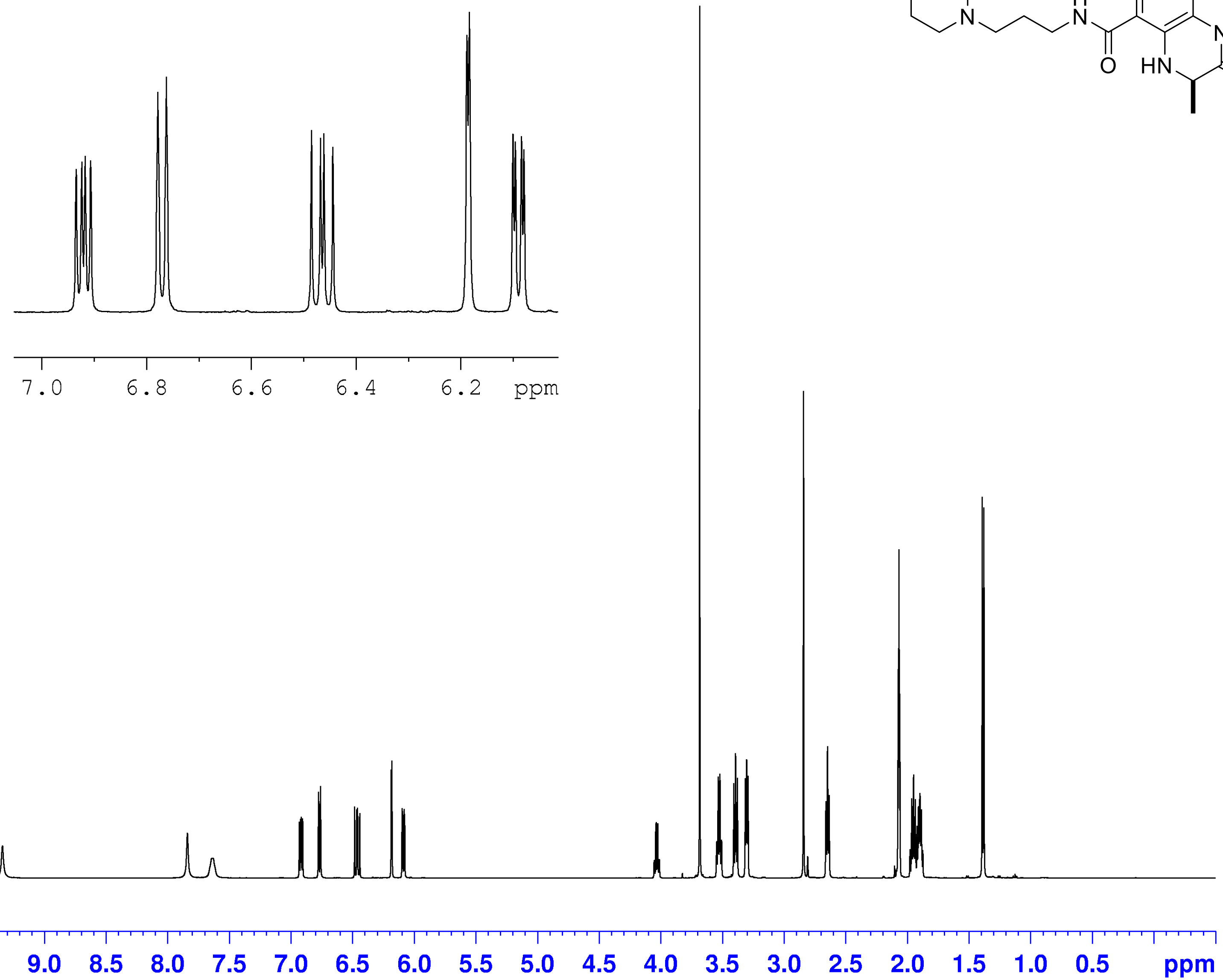

(3*R*)-*N*-[3-(7-methoxy-3,4-dihydroquinolin-1(2*H*)-yl)propyl]-6-fluoro-3-methyl-2-oxo-1,2,3,4-tetrahydroquinoxaline-5-carboxamide (*R*)-**8** ^{13}C NMR

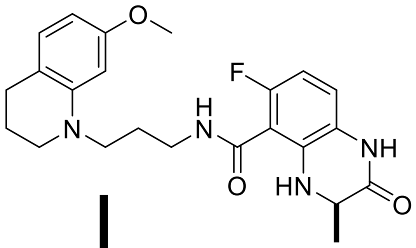


```
NAME              tr4039
EXPNO                  4
PROCNO                 1
Date_           20130114
Time               17.25
INSTRUM           avc500
PROBHD   5 mm CPDUL 13C
PULPROG          zgpg30
TD                 65536
SOLVENT          Acetone
NS                   280
DS                     2
SWH            31250.000 Hz
FIDRES          0.476837 Hz
AQ             1.0486259 sec
RG                   912
DW                16.000 usec
DE                 20.00 usec
TE                 298.0 K
D1            2.00000000 sec
D11           0.03000000 sec
TD0                    1

======== CHANNEL f1 ========
NUC1                 13C
P1                 10.25 usec
PL1                 8.00 dB
PL1W          1.62029624 W
SFO1         125.8131151 MHz

======== CHANNEL f2 ========
CPDPRG2          waltz16
NUC2                  1H
PCPD2              80.00 usec
PL2                 6.00 dB
PL12               23.56 dB
PL13               29.56 dB
PL2W          0.95905519 W
PL12W         0.01682068 W
PL13W         0.00422516 W
SFO2         500.3020012 MHz
SI                 32768
SF           125.8004218 MHz
WDW                   EM
SSB                    0
LB                  1.00 Hz
GB                     0
PC                  1.40
```

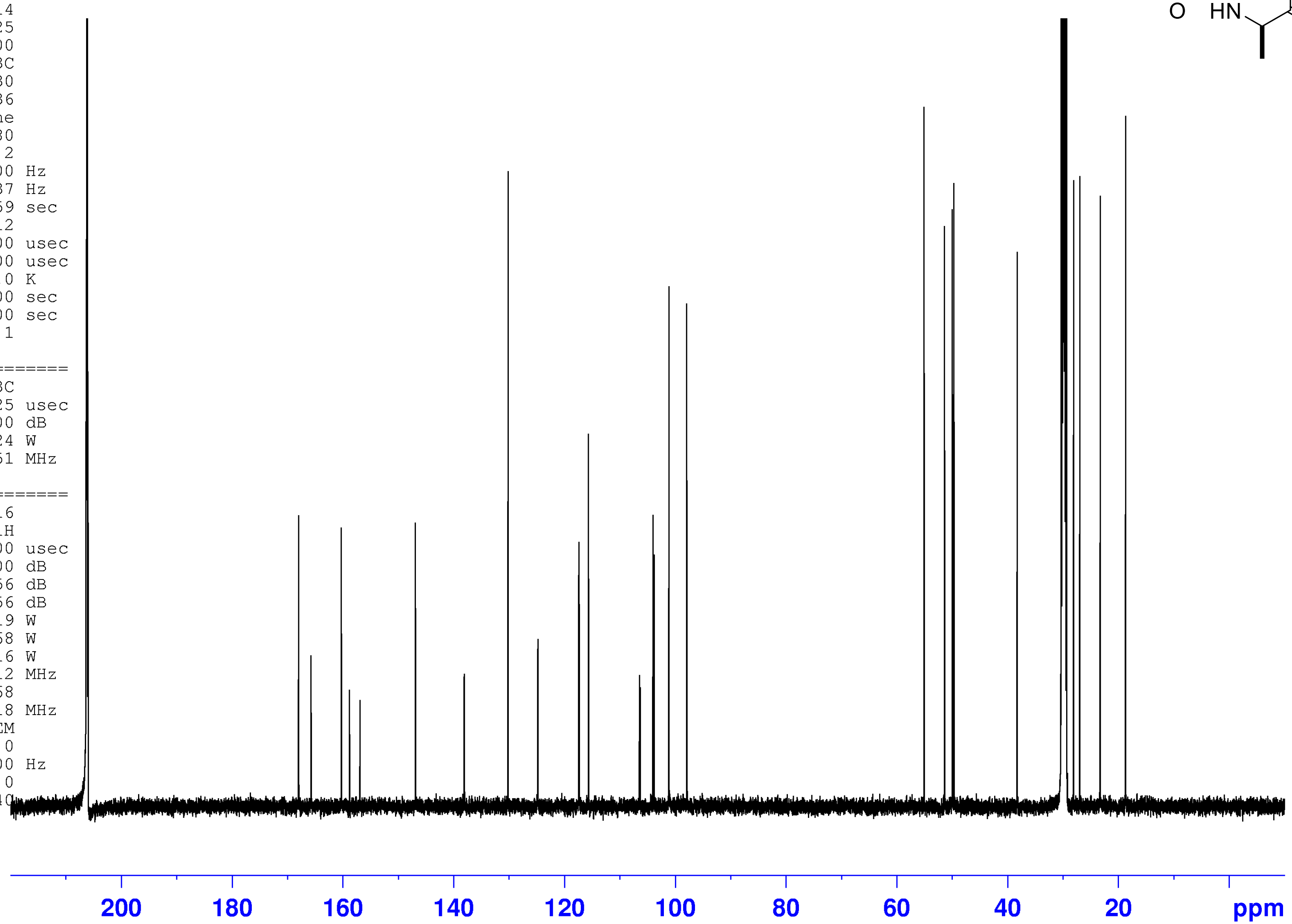

(3*R*)-*N*-[3-(7-methoxy-3,4-dihydroquinolin-1(2*H*)-yl)propyl]-6-fluoro-3-methyl-2-oxo-1,2,3,4-tetrahydroquinoxaline-5-carboxamide (*R*)-**8** ^{19}F NMR

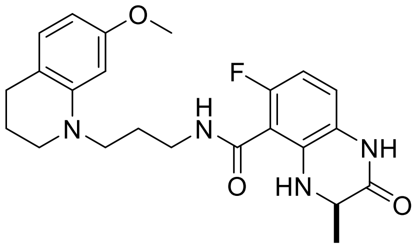

NAME	tr4039	
EXPNO	6	
PROCNO	1	
Date_	20130114	
Time	15.24	
INSTRUM	avb500	
PROBHD	5 mm PATXI 1H/	
PULPROG	zgflqn	
TD	131072	
SOLVENT	Acetone	
NS	16	
DS	4	
SWH	23437.500	Hz
FIDRES	0.178814	Hz
AQ	2.7962527	sec
RG	2050	
DW	21.333	usec
DE	6.50	usec
TE	298.0	K
D1	1.00000000	sec
TD0	1	
======== CHANNEL f1 ========		
NUC1	19F	
P1	10.00	usec
PL1	0.90	dB
SFO1	470.3947819	MHz
SI	131072	
SF	470.4512360	MHz
WDW	EM	
SSB	0	
LB	0.30	Hz
GB	0	
PC	1.00	

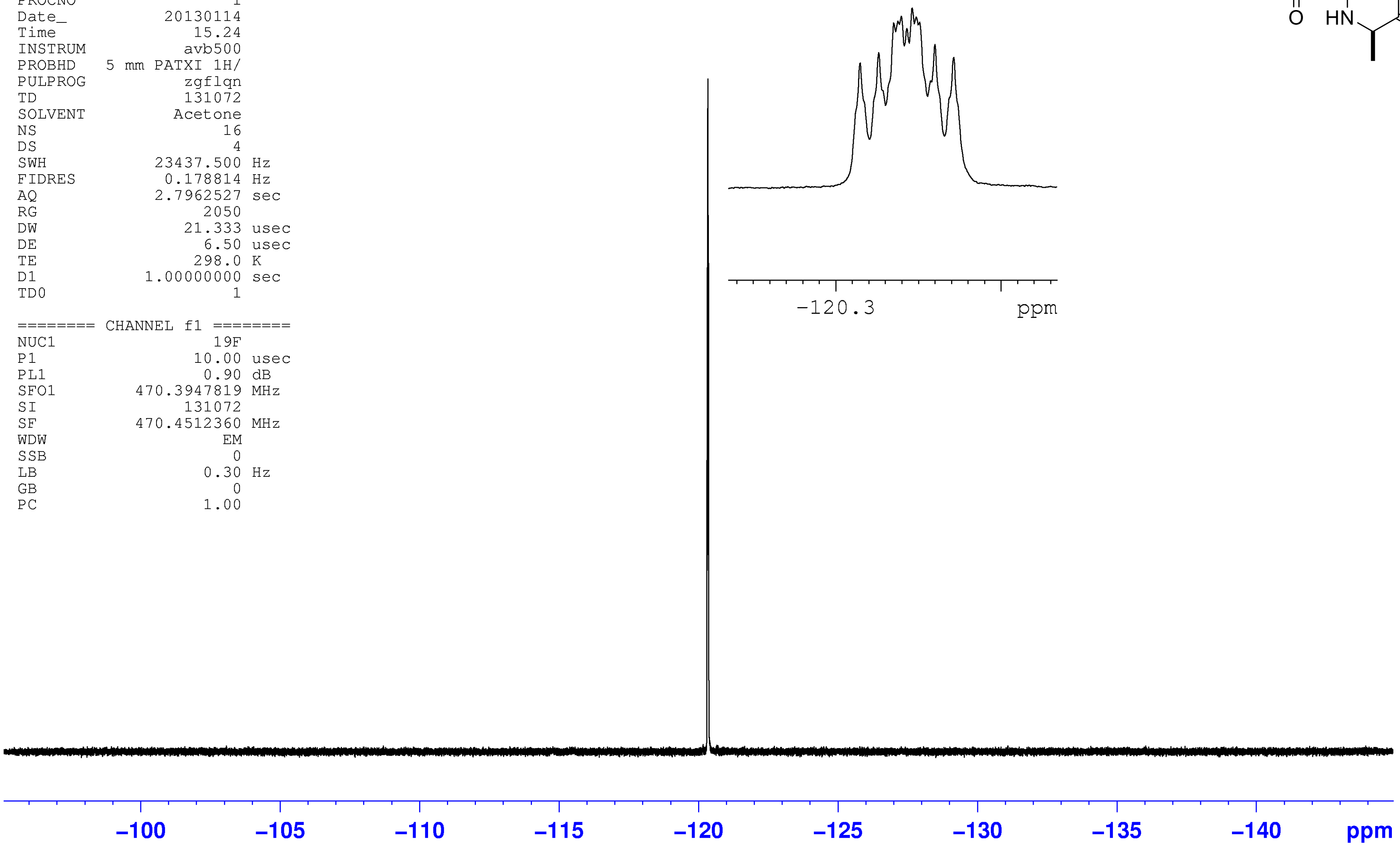

(3*R*)-*N*-[3-(7-methoxy-3,4-dihydroquinolin-1(2*H*)-yl)propyl]-7-fluoro-3-methyl-2-oxo-1,2,3,4-tetrahydroquinoxaline-5-carboxamide (*R*)-**7** ^{1}H NMR

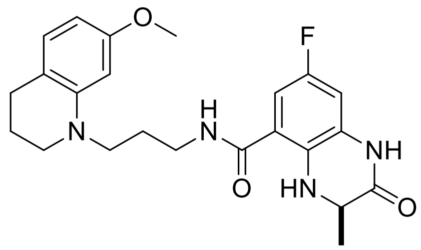


```
NAME                tr4040
EXPNO                   11
PROCNO                   1
Date_             20130114
Time                 18.33
INSTRUM             avc500
PROBHD    5 mm CPDUL 13C
PULPROG               zg30
TD                   65536
SOLVENT            Acetone
NS                      16
DS                       2
SWH             10330.578 Hz
FIDRES           0.157632 Hz
AQ              3.1719923 sec
RG                     2.8
DW                  48.400 usec
DE                    6.00 usec
TE                   298.0 K
D1              1.00000000 sec
TD0                      1

======== CHANNEL f1 ========
NUC1                    1H
P1                   10.60 usec
PL1                   6.00 dB
PL1W            0.95905519 W
SFO1           500.3030896 MHz
SI                   32768
SF             500.3000096 MHz
WDW                     EM
SSB                      0
LB                    0.30 Hz
GB                       0
PC                    1.00
```

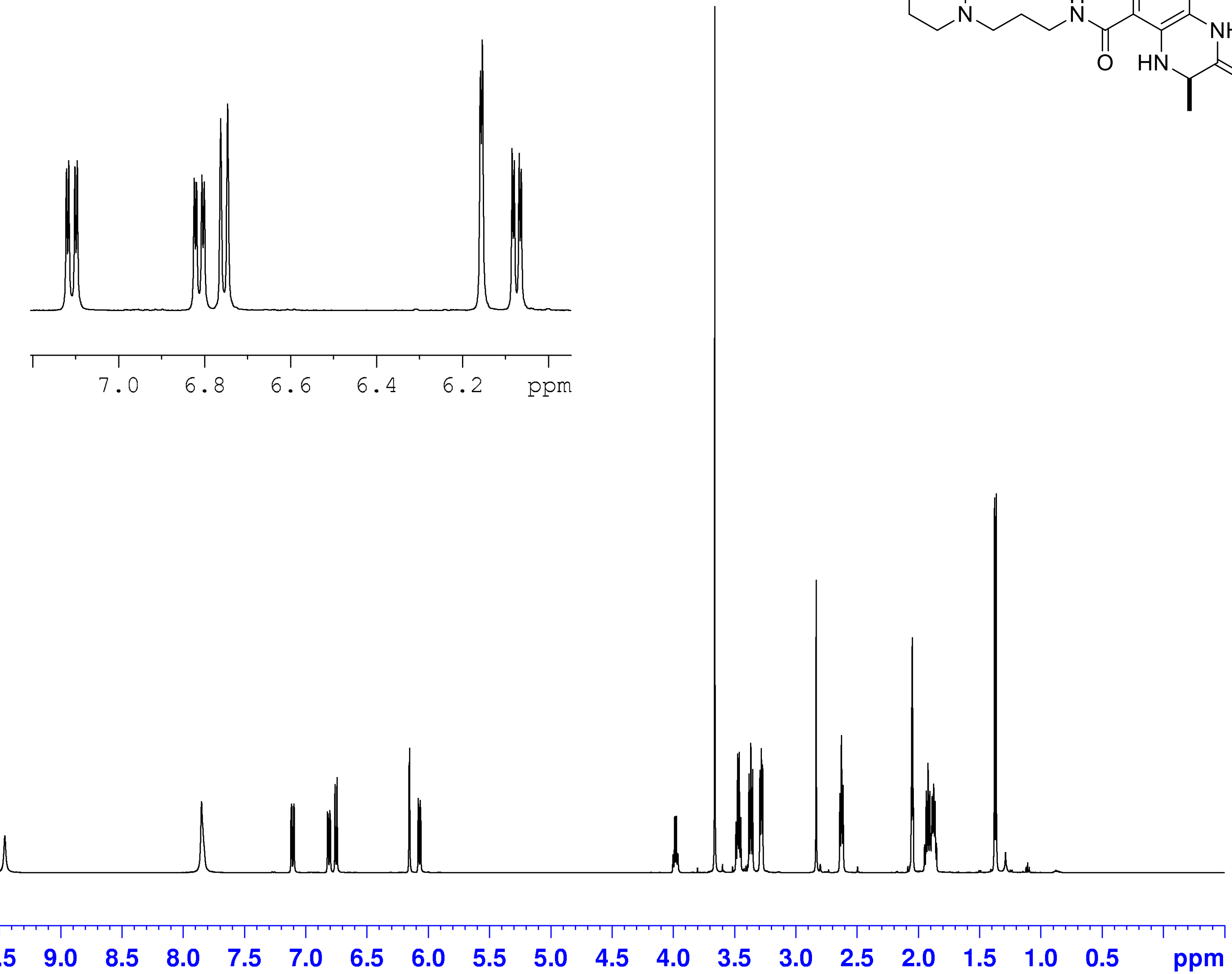

(3*R*)-*N*-[3-(7-methoxy-3,4-dihydroquinolin-1(2*H*)-yl)propyl]-7-fluoro-3-methyl-2-oxo-1,2,3,4-tetrahydroquinoxaline-5-carboxamide (*R*)-**7** ^{13}C NMR

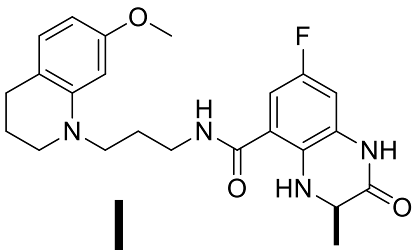

NAME	tr4040
EXPNO	14
PROCNO	1
Date_	20130114
Time	19.21
INSTRUM	avc500
PROBHD	5 mm CPDUL 13C
PULPROG	zgpg30
TD	65536
SOLVENT	Acetone
NS	512
DS	2
SWH	31250.000 Hz
FIDRES	0.476837 Hz
AQ	1.0486259 sec
RG	912
DW	16.000 usec
DE	20.00 usec
TE	298.0 K
D1	2.00000000 sec
D11	0.03000000 sec
TD0	1
======== CHANNEL f1 ========	
NUC1	13C
P1	10.25 usec
PL1	8.00 dB
PL1W	1.62029624 W
SFO1	125.8131151 MHz
======== CHANNEL f2 ========	
CPDPRG2	waltz16
NUC2	1H
PCPD2	80.00 usec
PL2	6.00 dB
PL12	23.56 dB
PL13	29.56 dB
PL2W	0.95905519 W
PL12W	0.01682068 W
PL13W	0.00422516 W
SFO2	500.3020012 MHz
SI	32768
SF	125.8004218 MHz
WDW	EM
SSB	0
LB	1.00 Hz
GB	0
PC	1.40

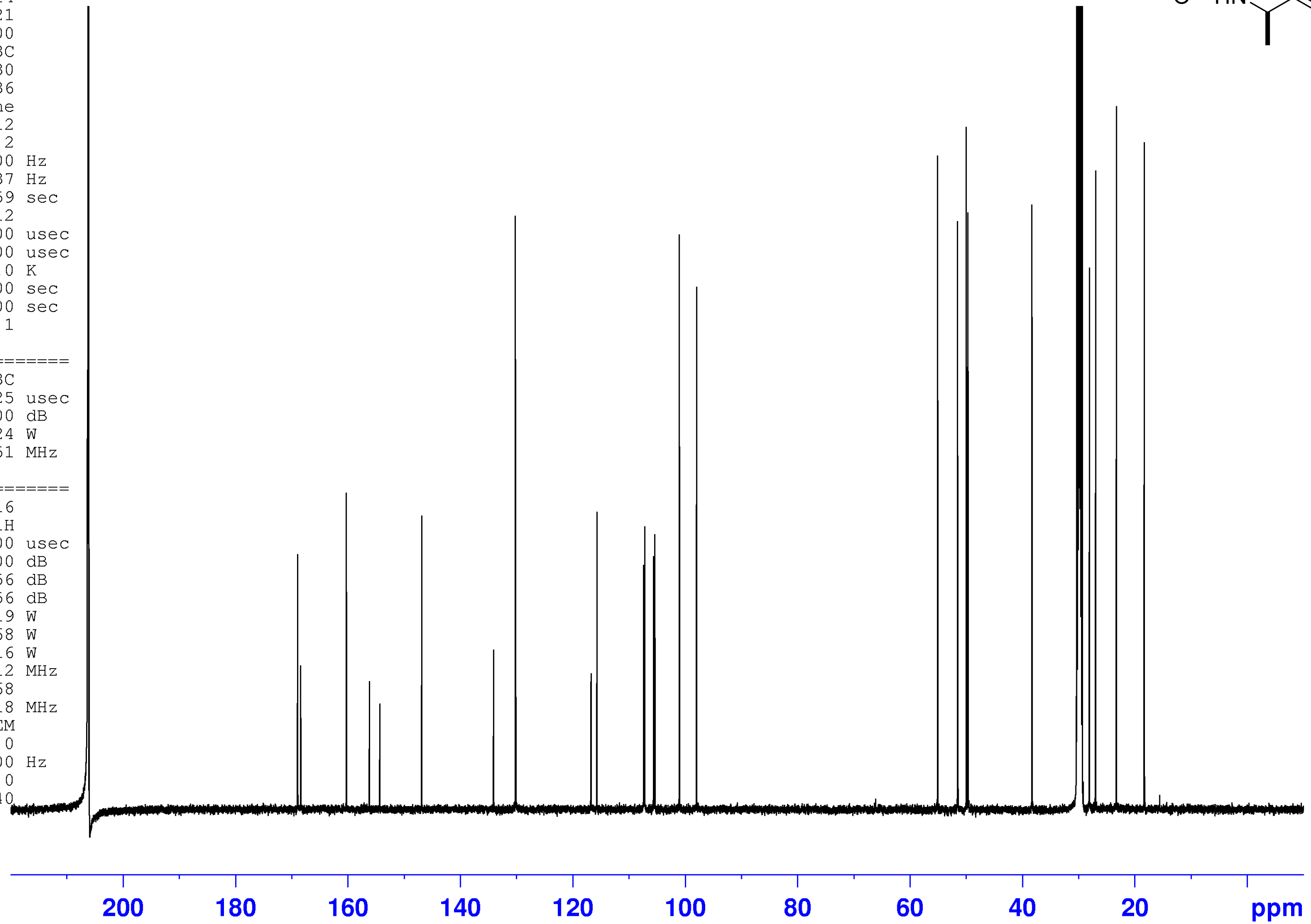

(3*R*)-*N*-[3-(7-methoxy-3,4-dihydroquinolin-1(2*H*)-yl)propyl]-7-fluoro-3-methyl-2-oxo-1,2,3,4-tetrahydroquinoxaline-5-carboxamide (*R*)-**7** ^{19}F NMR

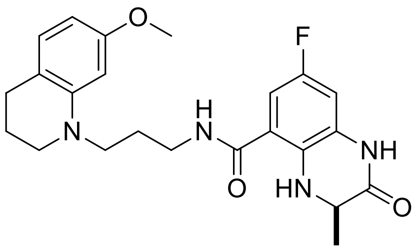


```
NAME              tr4040
EXPNO                 16
PROCNO                 1
Date_           20130114
Time               16.17
INSTRUM           avb500
PROBHD   5 mm PATXI 1H/
PULPROG           zgflqn
TD                131072
SOLVENT          Acetone
NS                    16
DS                     4
SWH           46875.000 Hz
FIDRES         0.357628 Hz
AQ            1.3981513 sec
RG                  2050
DW               10.667 usec
DE                 6.50 usec
TE                298.0 K
D1           1.00000000 sec
TD0                    1

======== CHANNEL f1 ========
NUC1                 19F
P1                 10.00 usec
PL1                 0.90 dB
SFO1         470.3919591 MHz
SI                 65536
SF           470.4503009 MHz
WDW                   EM
SSB                    0
LB                  0.30 Hz
GB                     0
PC                  1.00
```

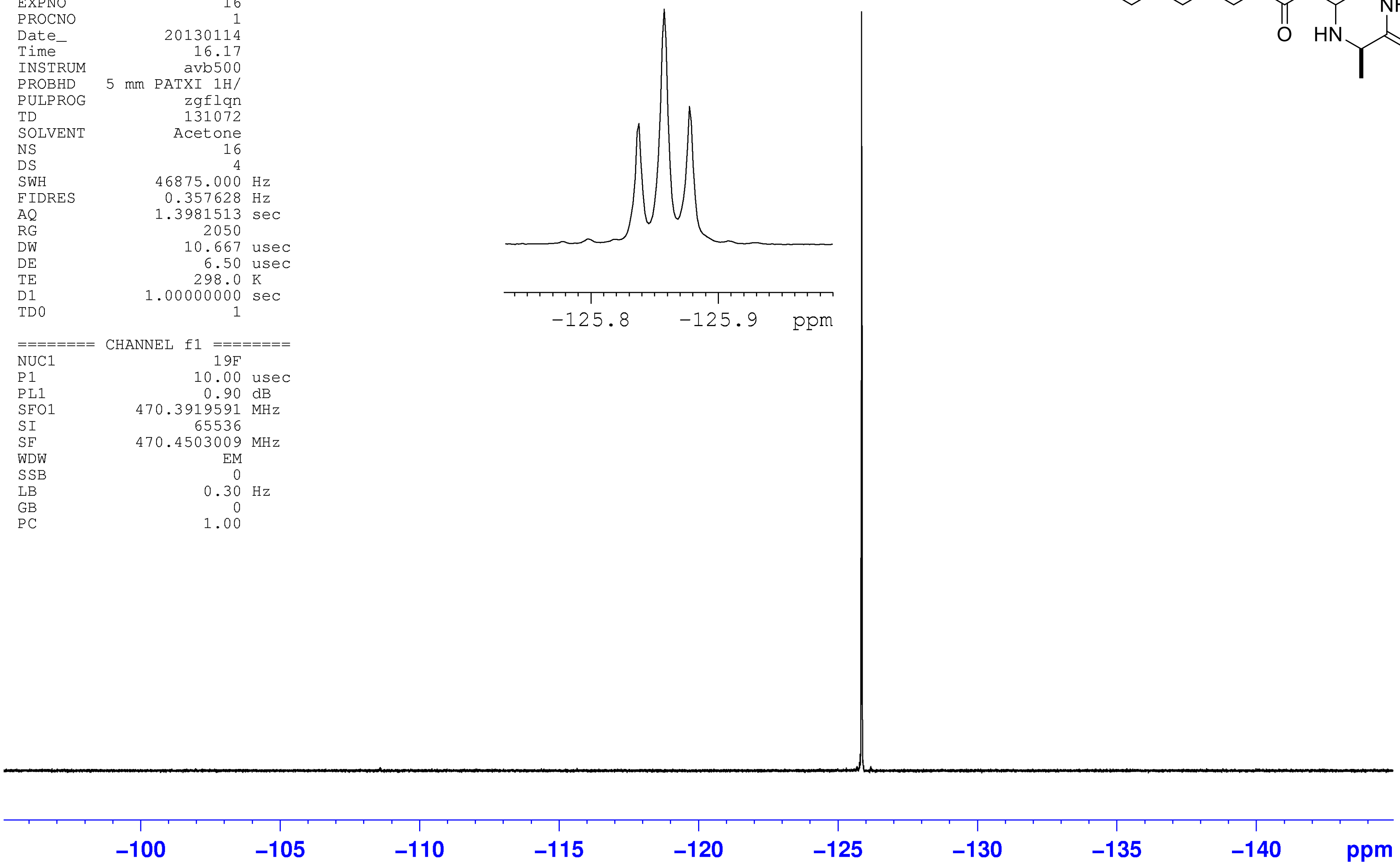

7-Bromo-2-methyl-2*H*-1,4-benzoxazin-3(4H)-one **33** ^{1}H NMR

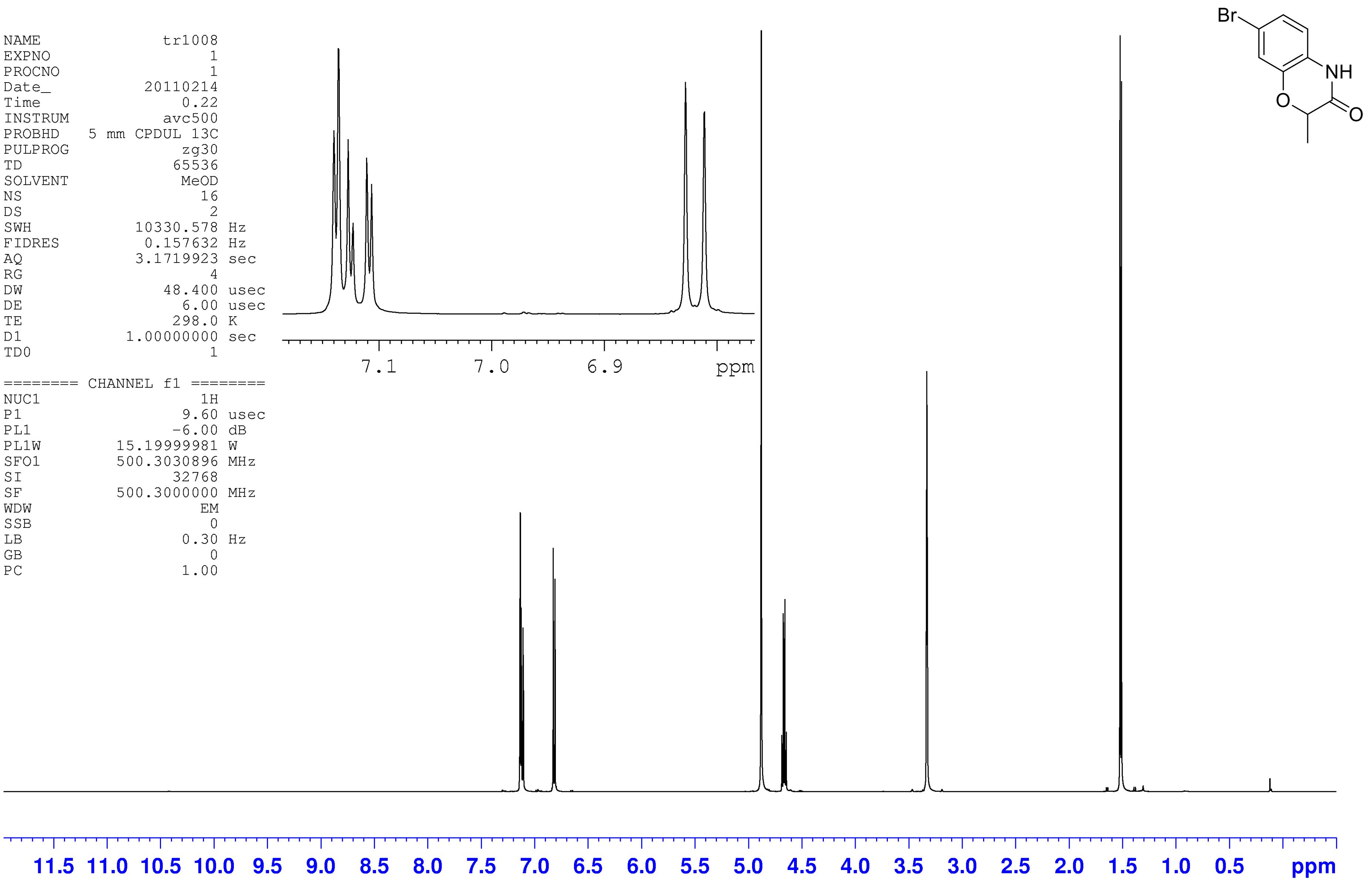

7-Bromo-2-methyl-2*H*-1,4-benzoxazin-3(4H)-one **33** ^{13}C NMR

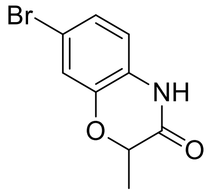

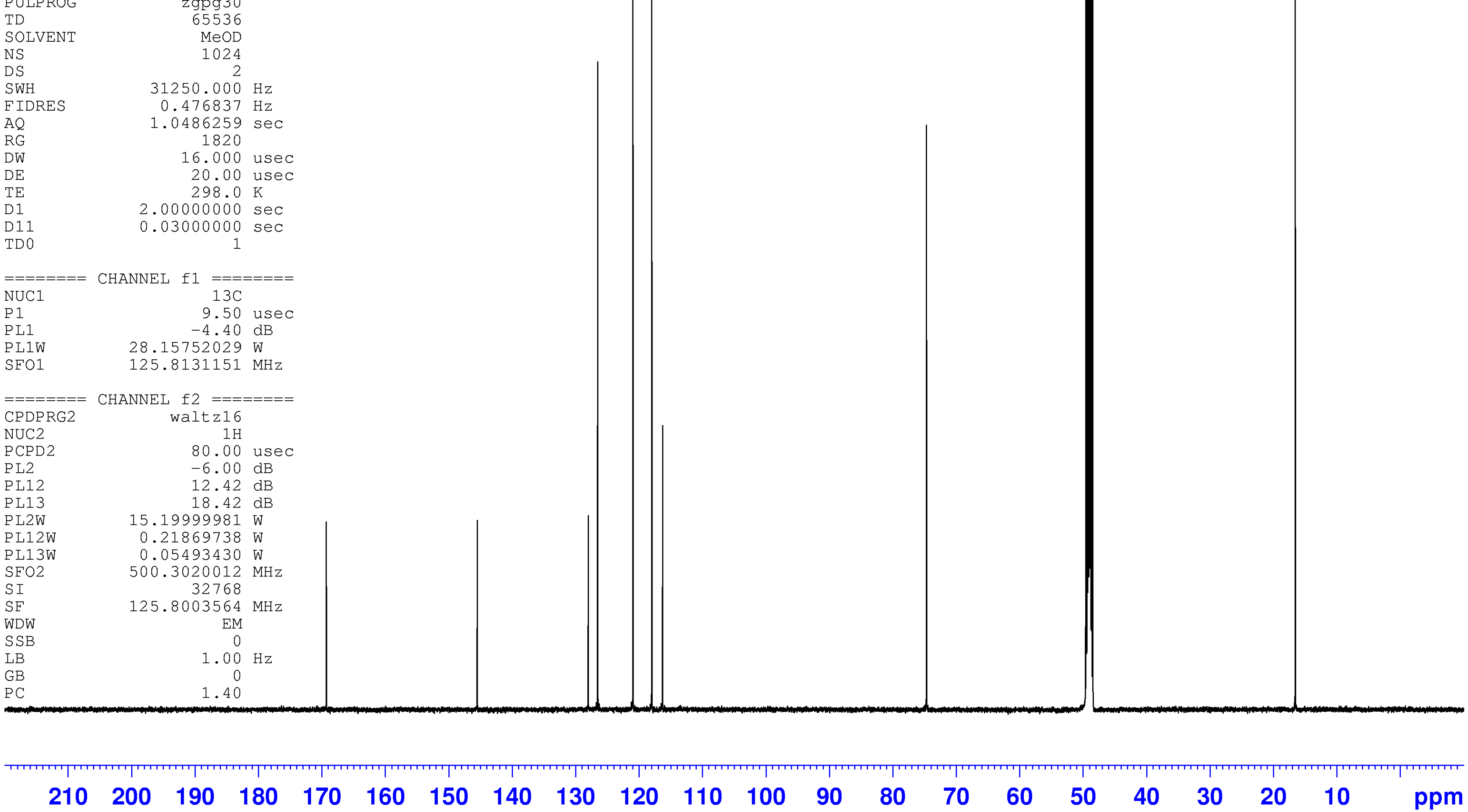

8-Bromo-2-methyl-2*H*-1,4-benzoxazin-3(4H)-one **34** ^{1}H NMR

NAME	tr1003
EXPNO	1
PROCNO	1
Date_	20110127
Time	21.22
INSTRUM	dpx400
PROBHD	5 mm Dual 1H/1
PULPROG	zg60
TD	32768
SOLVENT	CDCl3
NS	16
DS	2
SWH	5592.841 Hz
FIDRES	0.170680 Hz
AQ	2.9295092 sec
RG	203.2
DW	89.400 usec
DE	17.00 usec
TE	300.0 K
D1	1.00000000 sec

======== CHANNEL f1 ========

NUC1	1H
P1	7.30 usec
PL1	0.00 dB
SFO1	400.1320007 MHz
SI	32768
SF	400.1300182 MHz
WDW	EM
SSB	0
LB	0.30 Hz
GB	0
PC	0.60

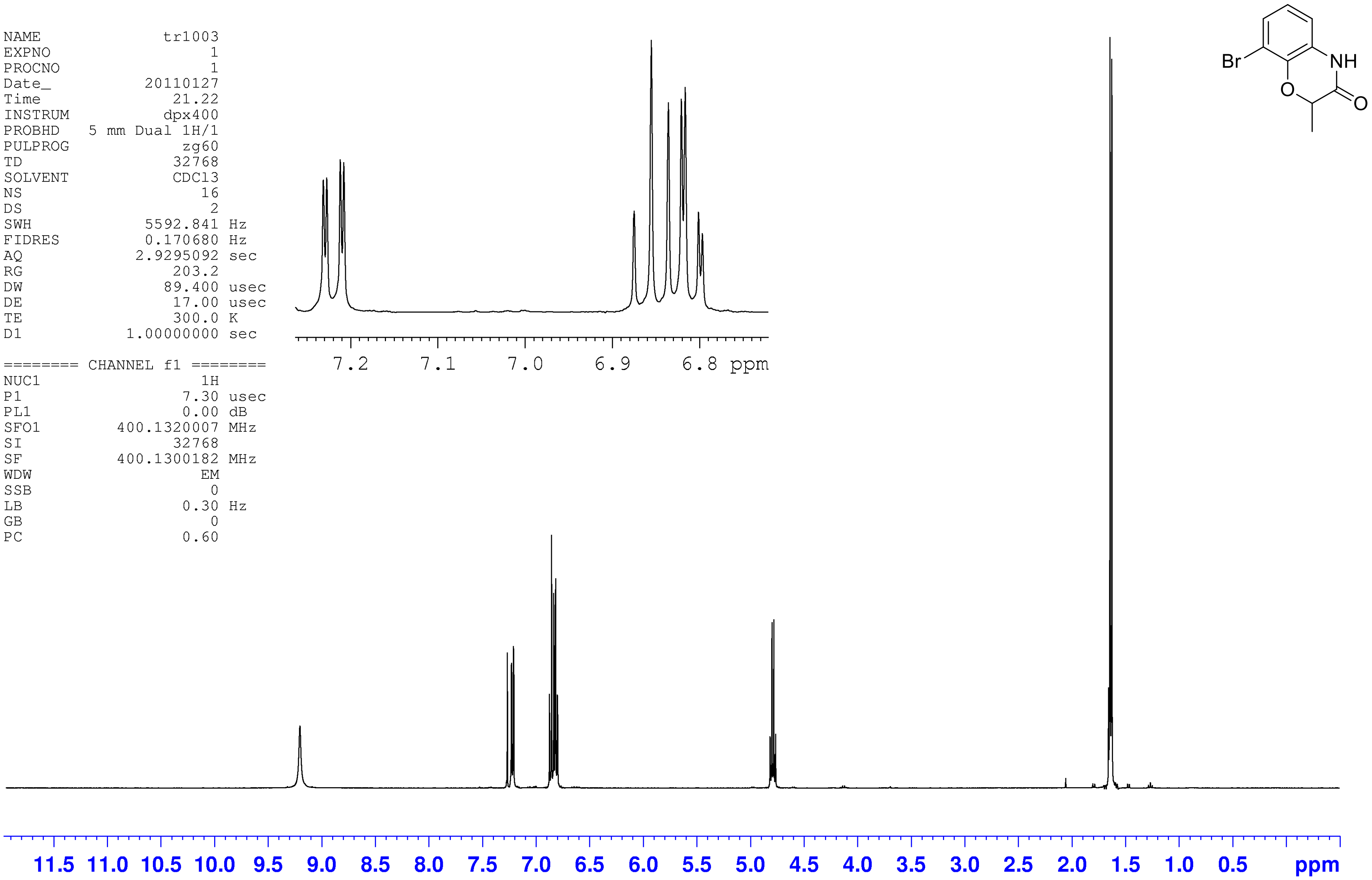

8-Bromo-2-methyl-2*H*-1,4-benzoxazin-3(4H)-one **34** ^{13}C NMR

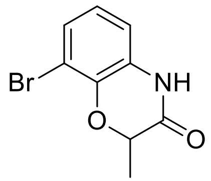

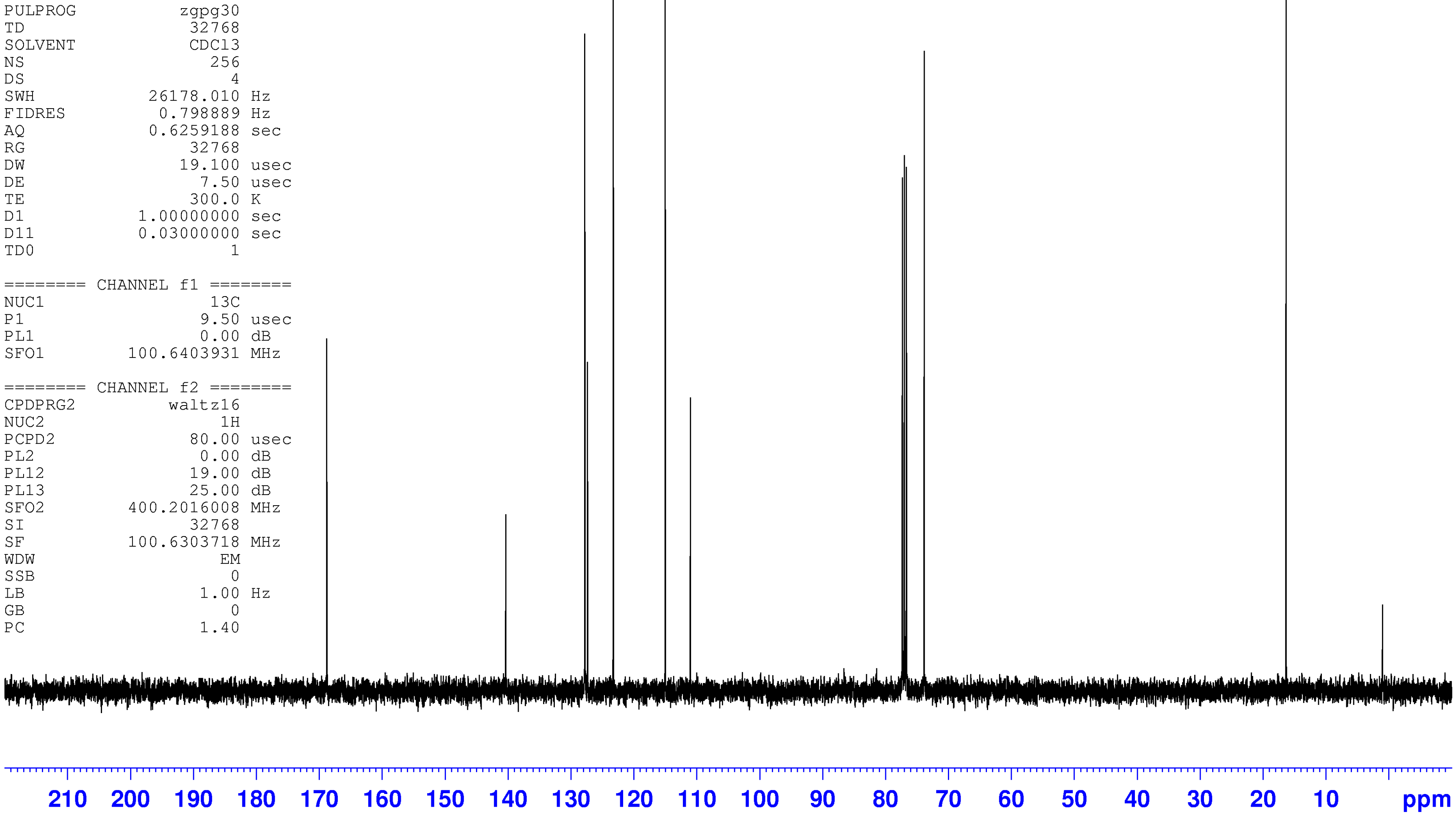

Methyl 2-methyl-3-oxo-3,4-dihydro-2*H*-1,4benzoxazine-8-carboxylate **35** ^{1}H NMR

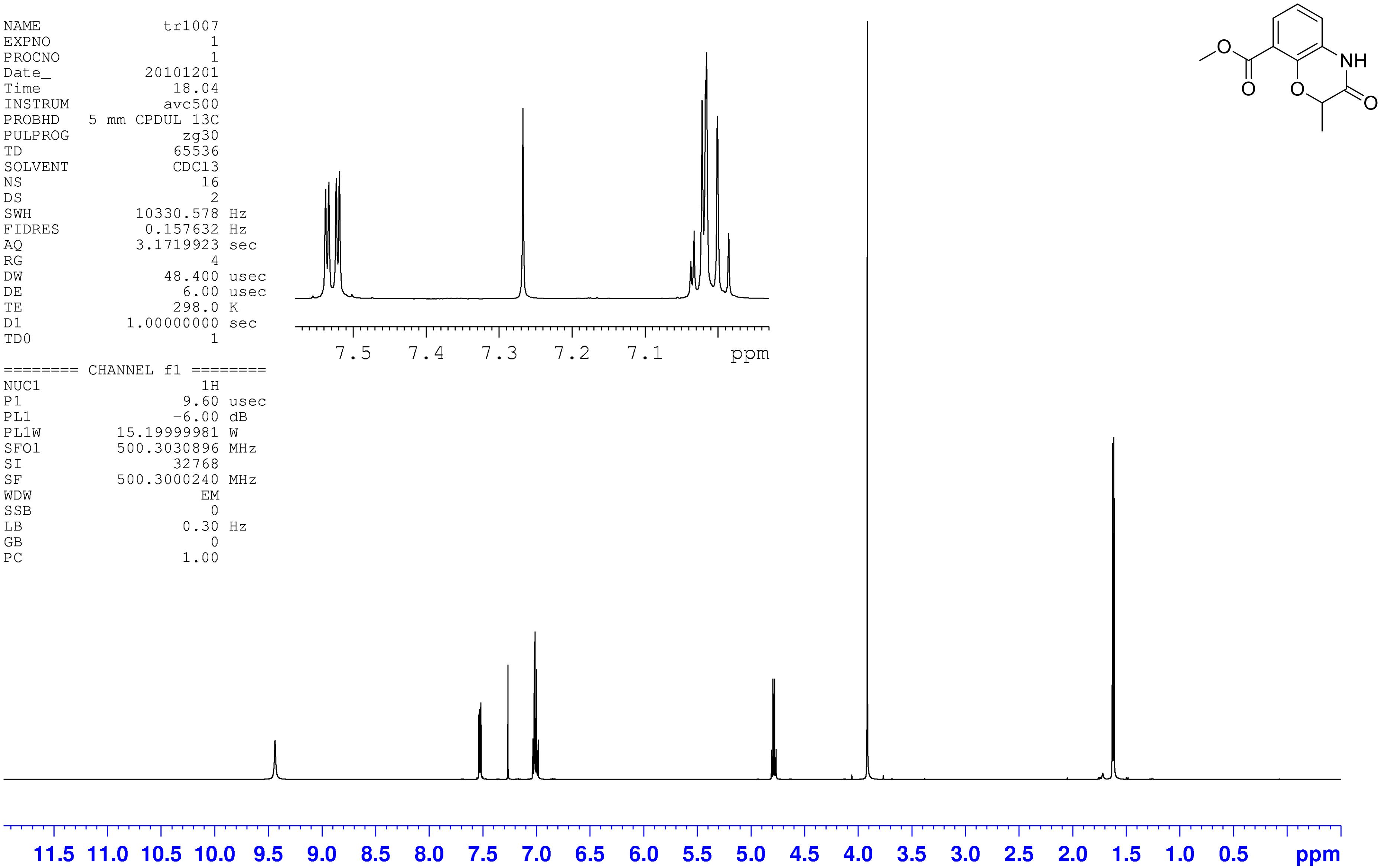

Methyl 2-methyl-3-oxo-3,4-dihydro-2*H*-1,4benzoxazine-8-carboxylate **35** ^{13}C NMR

```
NAME              tr1007
EXPNO                  4
PROCNO                 1
Date_           20101201
Time               19.14
INSTRUM           avc500
PROBHD    5 mm CPDUL 13C
PULPROG          zgpg30
TD                 65536
SOLVENT            CDCl3
NS                  1024
DS                     2
SWH            31250.000 Hz
FIDRES          0.476837 Hz
AQ             1.0486259 sec
RG                  1820
DW                16.000 usec
DE                 20.00 usec
TE                 298.0 K
D1            2.00000000 sec
D11           0.03000000 sec
TD0                    1

======== CHANNEL f1 ========
NUC1                 13C
P1                  9.50 usec
PL1                -4.40 dB
PL1W         28.15752029 W
SFO1         125.8131151 MHz

======== CHANNEL f2 ========
CPDPRG2          waltz16
NUC2                  1H
PCPD2              80.00 usec
PL2                -6.00 dB
PL12               12.42 dB
PL13               18.42 dB
PL2W         15.19999981 W
PL12W         0.21869738 W
PL13W         0.05493430 W
SFO2         500.3020012 MHz
SI                 32768
SF           125.8005438 MHz
WDW                   EM
SSB                    0
LB                  1.00 Hz
GB                     0
PC                  1.40
```

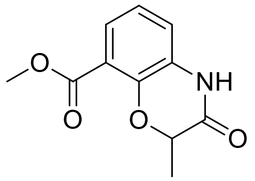

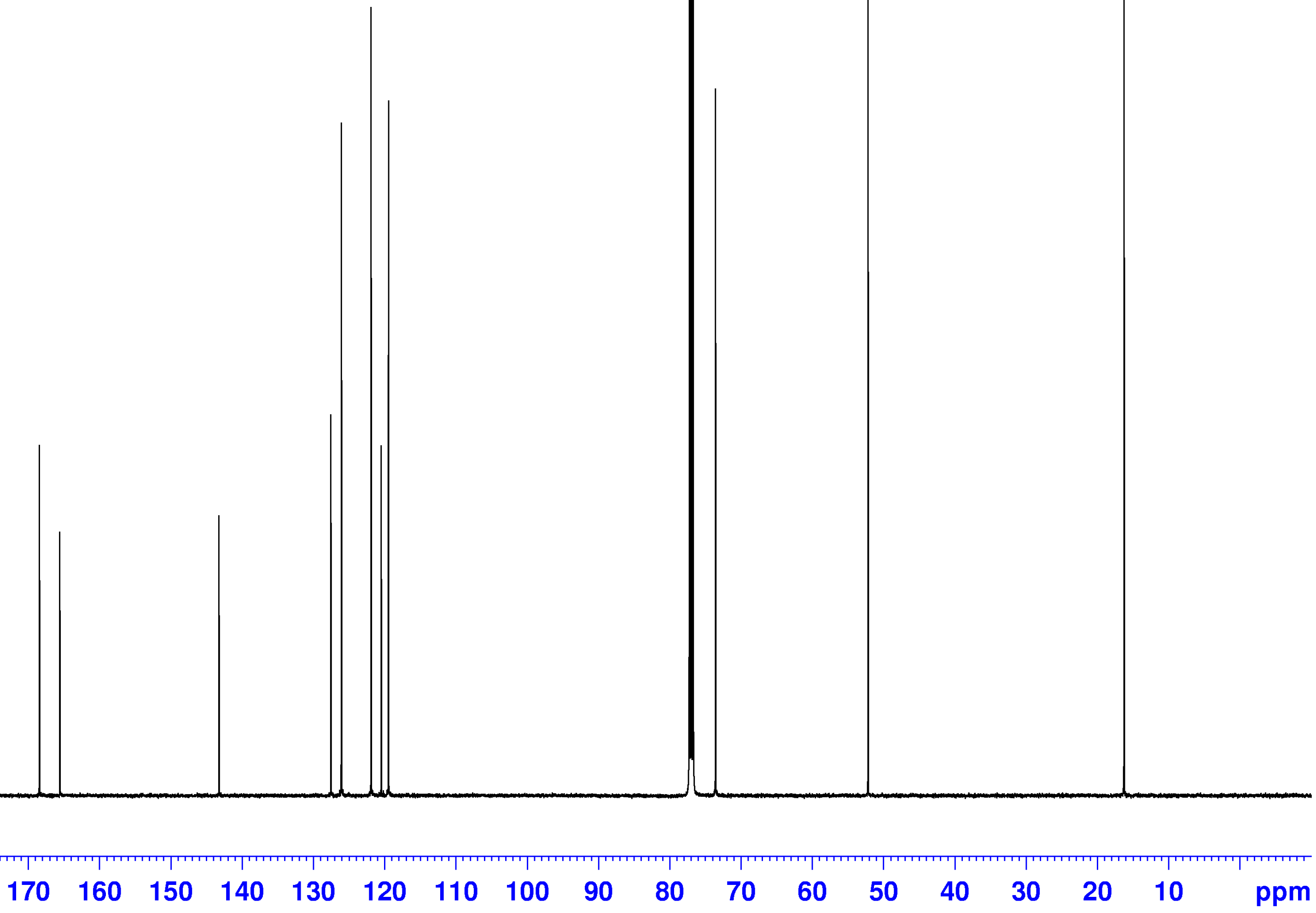

2-Methyl-3-oxo-3,4-dihydro-2*H*-1,4benzoxazine-8-carboxylic acid **36** ^{1}H NMR

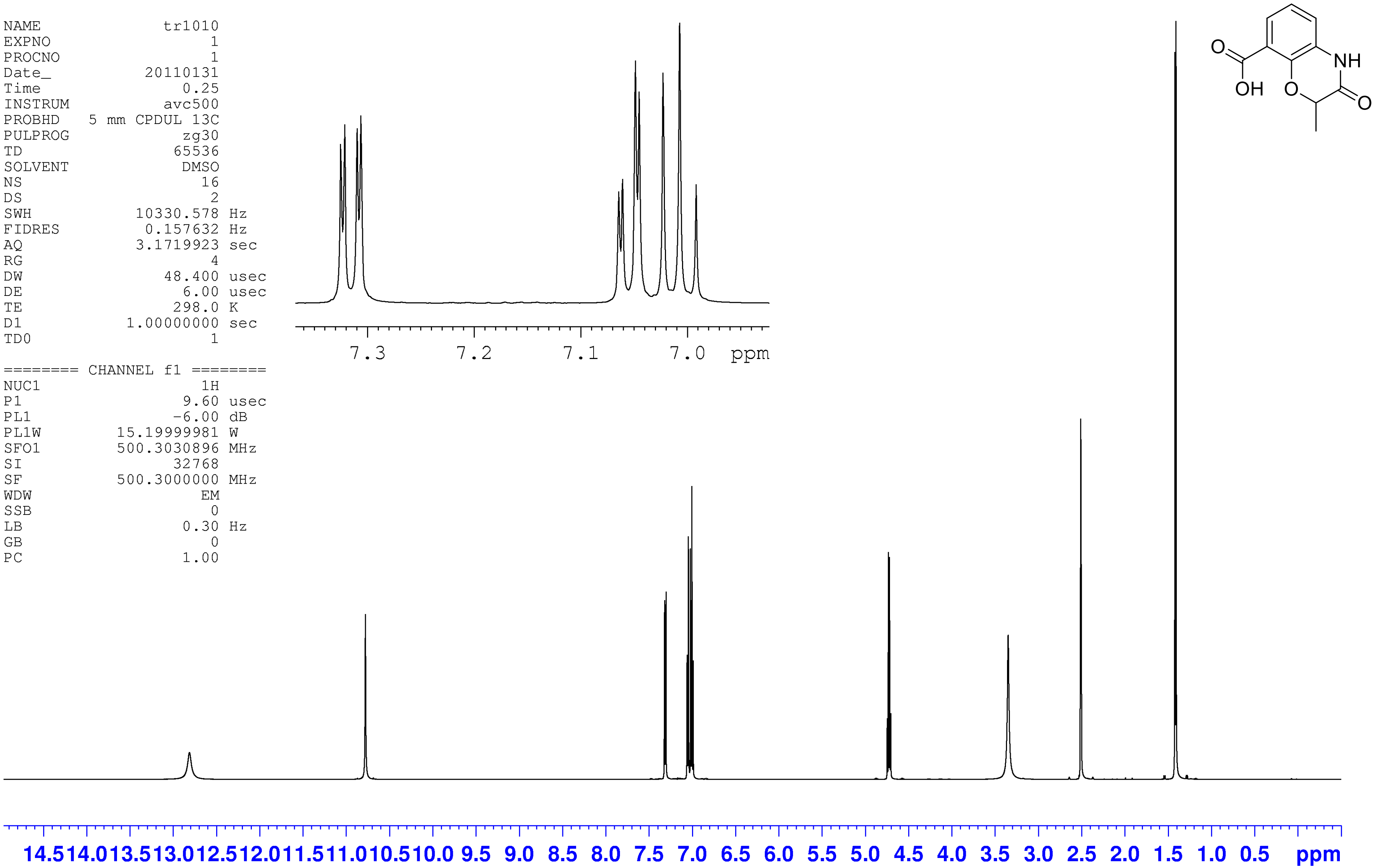


```
NAME              tr1010
EXPNO                  1
PROCNO                 1
Date_           20110131
Time                0.25
INSTRUM           avc500
PROBHD    5 mm CPDUL 13C
PULPROG             zg30
TD                 65536
SOLVENT             DMSO
NS                    16
DS                     2
SWH            10330.578 Hz
FIDRES          0.157632 Hz
AQ             3.1719923 sec
RG                     4
DW                48.400 usec
DE                  6.00 usec
TE                 298.0 K
D1            1.00000000 sec
TD0                    1

======== CHANNEL f1 ========
NUC1                  1H
P1                  9.60 usec
PL1                -6.00 dB
PL1W         15.19999981 W
SFO1         500.3030896 MHz
SI                 32768
SF           500.3000000 MHz
WDW                   EM
SSB                    0
LB                  0.30 Hz
GB                     0
PC                  1.00
```

2-Methyl-3-oxo-3,4-dihydro-2*H*-1,4benzoxazine-8-carboxylic acid **36** ^{13}C NMR

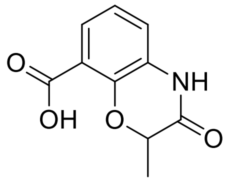


```
NAME              tr1010
EXPNO                  4
PROCNO                 1
Date_           20110131
Time                1.09
INSTRUM           avc500
PROBHD   5 mm CPDUL 13C
PULPROG          zgpg30
TD                 65536
SOLVENT             DMSO
NS                   512
DS                     2
SWH            31250.000 Hz
FIDRES          0.476837 Hz
AQ             1.0486259 sec
RG                  1440
DW                16.000 usec
DE                 20.00 usec
TE                 298.0 K
D1            2.00000000 sec
D11           0.03000000 sec
TD0                    1

======== CHANNEL f1 ========
NUC1                 13C
P1                  9.50 usec
PL1                -4.40 dB
PL1W         28.15752029 W
SFO1         125.8131151 MHz

======== CHANNEL f2 ========
CPDPRG2          waltz16
NUC2                  1H
PCPD2              80.00 usec
PL2                -6.00 dB
PL12               12.42 dB
PL13               18.42 dB
PL2W         15.19999981 W
PL12W         0.21869738 W
PL13W         0.05493430 W
SFO2         500.3020012 MHz
SI                 32768
SF           125.8005954 MHz
WDW                   EM
SSB                    0
LB                  1.00 Hz
GB                     0
PC                  1.40
```

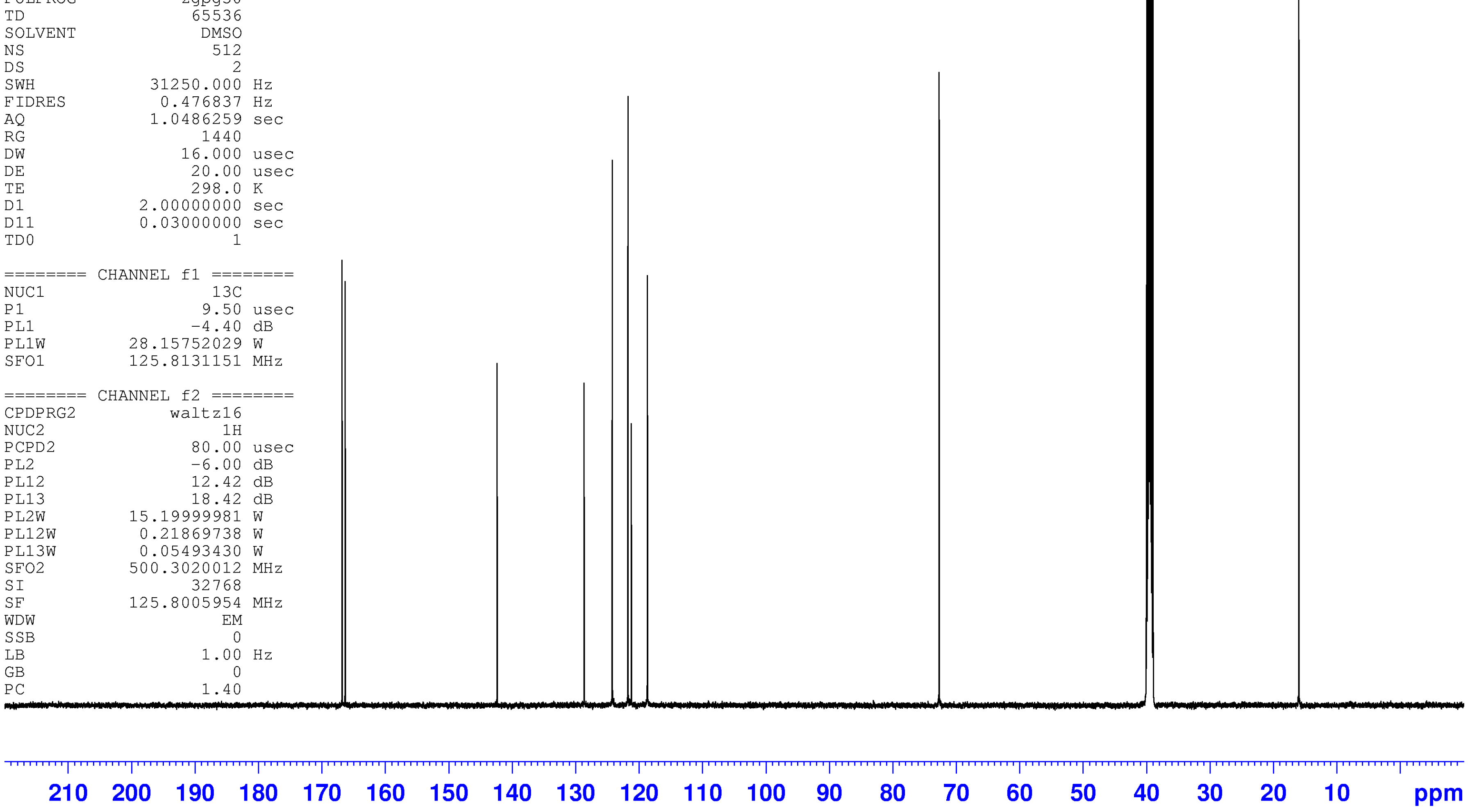

N-[2-(2,3-Dihydro-1*H*-indol-1-yl)ethyl]-2-methyl-3-oxo-3,4-dihydro-2*H*-1,4-benzoxazine-8-carboxamide **3** ^{1}H NMR

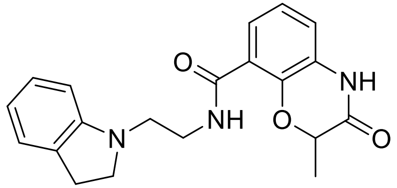


```
NAME              tr1078
EXPNO                  1
PROCNO                 1
Date_           20110705
Time               14.06
INSTRUM           avc500
PROBHD   5 mm CPDUL 13C
PULPROG             zg30
TD                 65536
SOLVENT            CDCl3
NS                    16
DS                     2
SWH           10330.578 Hz
FIDRES         0.157632 Hz
AQ            3.1719923 sec
RG                     4
DW                48.400 usec
DE                  6.00 usec
TE                 298.0 K
D1            1.00000000 sec
TD0                    1

======== CHANNEL f1 ========
NUC1                  1H
P1                  9.60 usec
PL1                -6.00 dB
PL1W         15.19999981 W
SFO1         500.3030896 MHz
SI                 32768
SF           500.3000240 MHz
WDW                   EM
SSB                    0
LB                  0.30 Hz
GB                     0
PC                  1.00
```

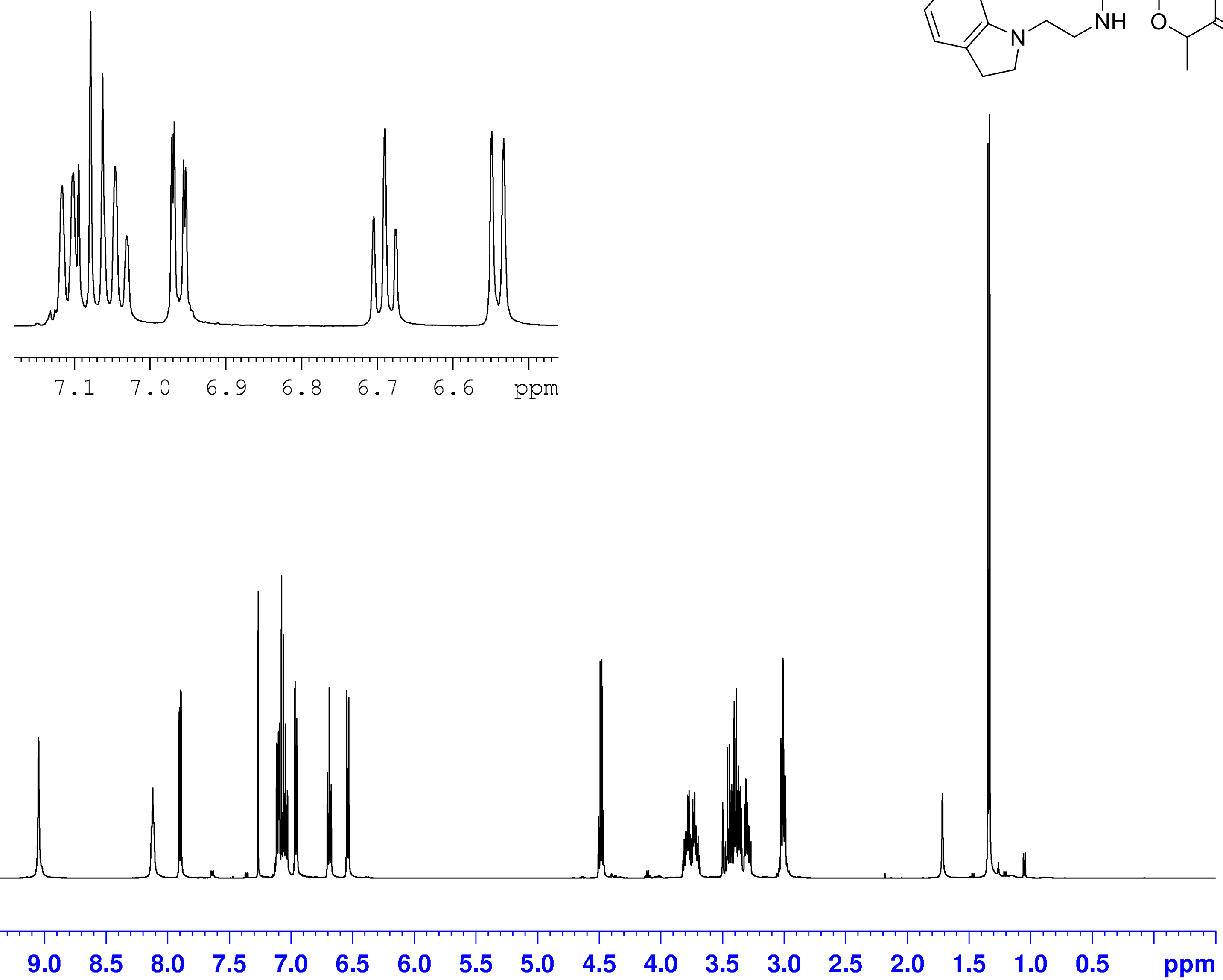

N-[2-(2,3-Dihydro-1*H*-indol-1-yl)ethyl]-2-methyl-3-oxo-3,4-dihydro-2*H*-1,4-benzoxazine-8-carboxamide **3** ^{13}C NMR

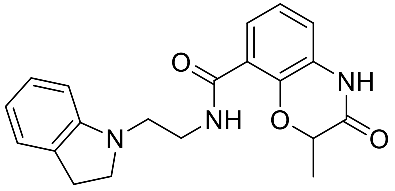

NAME	tr1078
EXPNO	4
PROCNO	1
Date_	20110705
Time	12.26
INSTRUM	avc500
PROBHD	5 mm CPDUL 13C
PULPROG	zgpg30
TD	65536
SOLVENT	CDCl3
NS	256
DS	2
SWH	31250.000 Hz
FIDRES	0.476837 Hz
AQ	1.0486259 sec
RG	1820
DW	16.000 usec
DE	20.00 usec
TE	298.0 K
D1	2.00000000 sec
D11	0.03000000 sec
TD0	1
======== CHANNEL f1 ========	
NUC1	13C
P1	9.10 usec
PL1	-4.40 dB
PL1W	28.15752029 W
SFO1	125.8131151 MHz
======== CHANNEL f2 ========	
CPDPRG2	waltz16
NUC2	1H
PCPD2	80.00 usec
PL2	-6.00 dB
PL12	12.42 dB
PL13	18.42 dB
PL2W	15.19999981 W
PL12W	0.21869738 W
PL13W	0.05493430 W
SFO2	500.3020012 MHz
SI	32768
SF	125.8005438 MHz
WDW	EM
SSB	0
LB	1.00 Hz
GB	0
PC	1.40

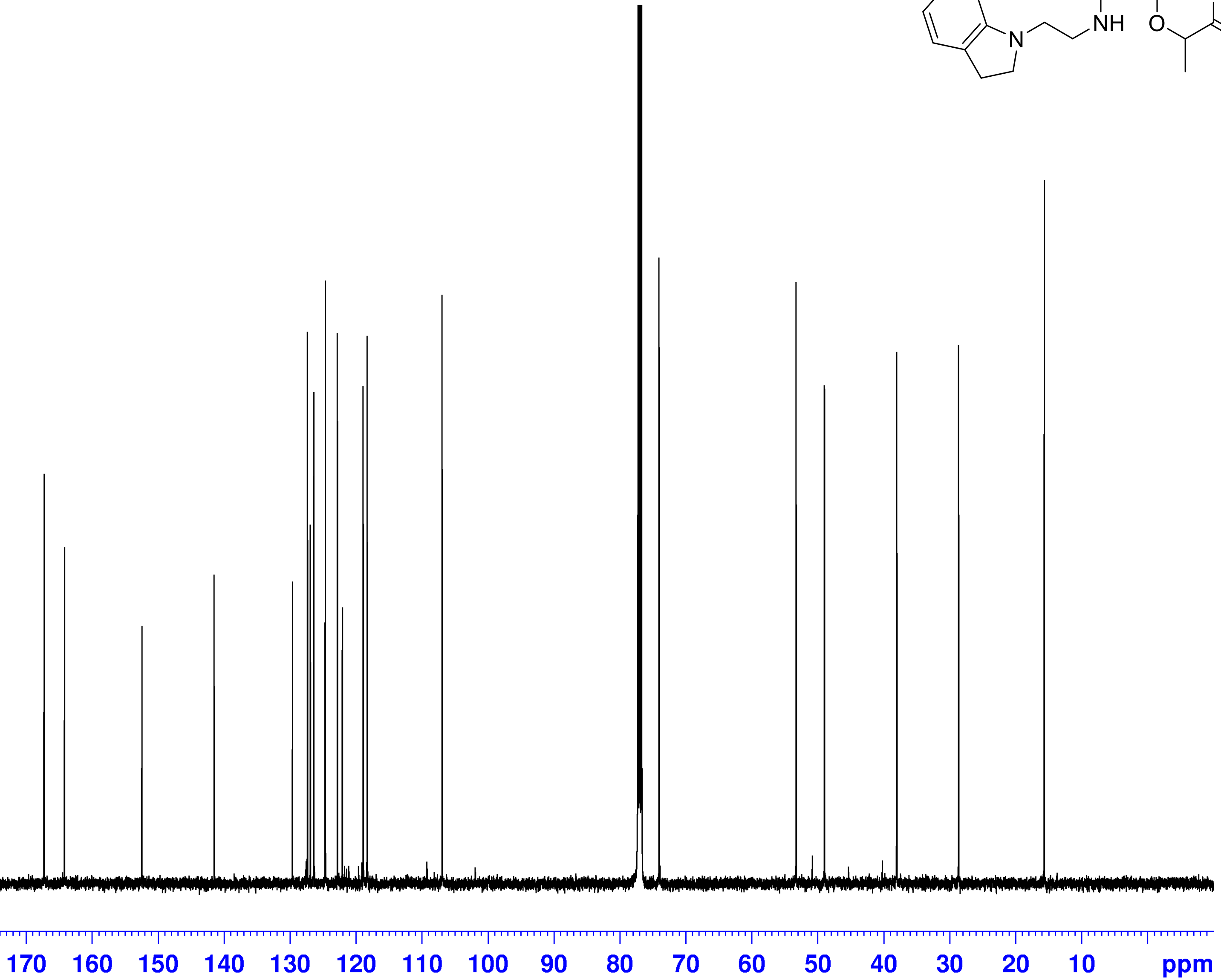

N-[3-(2,3-Dihydro-1*H*-indol-1-yl)propyl]-2-methyl-3-oxo-3,4-dihydro-2*H*-1,4-benzoxazine-8-carboxamide **37** ^{1}H-NMR

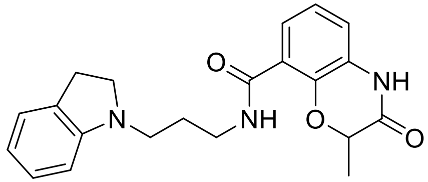

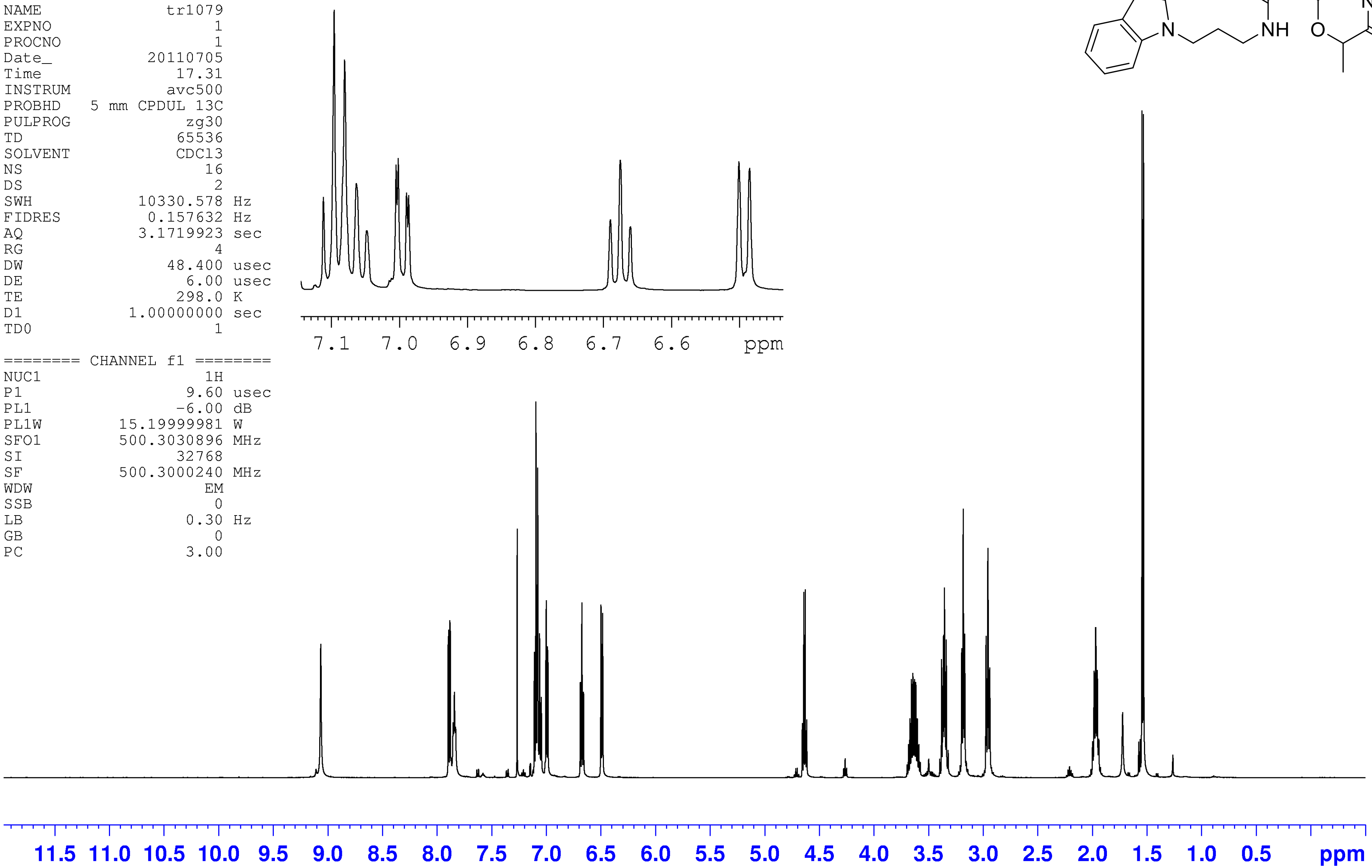

N-[3-(2,3-Dihydro-1*H*-indol-1-yl)propyl]-2-methyl-3-oxo-3,4-dihydro-2*H*-1,4-benzoxazine-8-carboxamide **37** ^{13}C-NMR

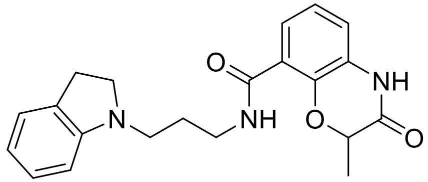


```
NAME              tr1079
EXPNO                  4
PROCNO                 1
Date_           20110705
Time               13.33
INSTRUM           avc500
PROBHD   5 mm CPDUL 13C
PULPROG          zgpg30
TD                 65536
SOLVENT            CDCl3
NS                   256
DS                     2
SWH            31250.000 Hz
FIDRES          0.476837 Hz
AQ             1.0486259 sec
RG                  1820
DW                16.000 usec
DE                 20.00 usec
TE                 298.0 K
D1            2.00000000 sec
D11           0.03000000 sec
TD0                    1

======== CHANNEL f1 ========
NUC1                 13C
P1                  9.10 usec
PL1                -4.40 dB
PL1W         28.15752029 W
SFO1         125.8131151 MHz

======== CHANNEL f2 ========
CPDPRG2          waltz16
NUC2                  1H
PCPD2              80.00 usec
PL2                -6.00 dB
PL12               12.42 dB
PL13               18.42 dB
PL2W         15.19999981 W
PL12W         0.21869738 W
PL13W         0.05493430 W
SFO2         500.3020012 MHz
SI                 32768
SF           125.8005438 MHz
WDW                   EM
SSB                    0
LB                  1.00 Hz
GB                     0
PC                  1.40
```

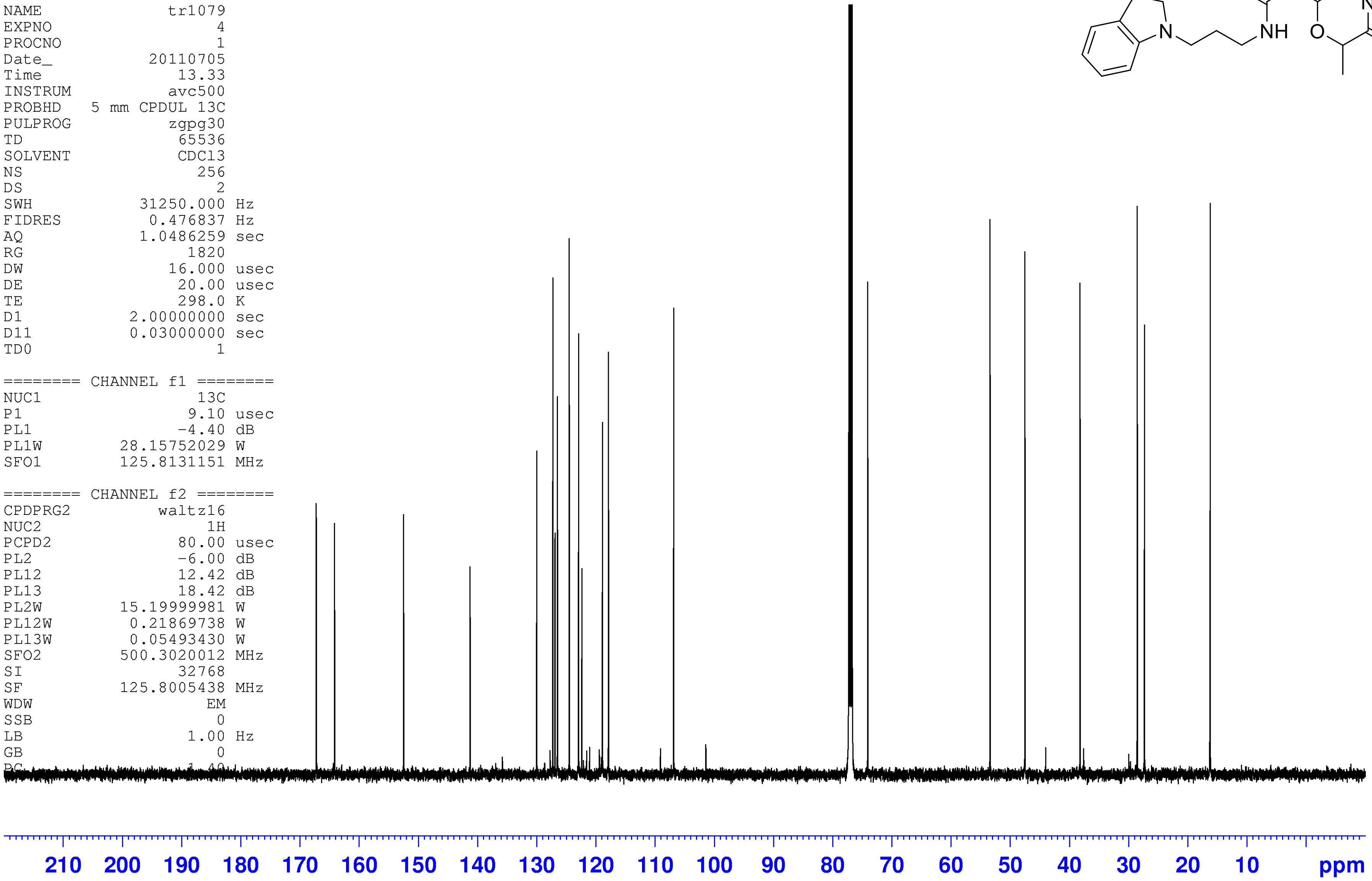

N-[2-(3,4-Dihydroquinolin-1(2*H*)-yl)propyl]-2-methyl-3-oxo-3,4-dihydro -2*H*-1,4-benzoxazine-8-carboxamide **4** ^{1}H-NMR

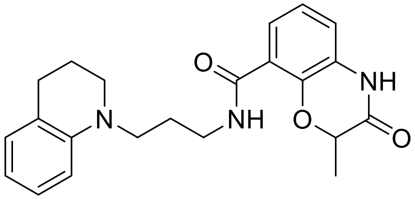

NAME	tr1062
EXPNO	1
PROCNO	1
Date_	20131113
Time	10.00
INSTRUM	avc500
PROBHD	5 mm CPDUL 13C
PULPROG	zg30
TD	65536
SOLVENT	CDCl3
NS	16
DS	4
SWH	10330.578 Hz
FIDRES	0.157632 Hz
AQ	3.1719923 sec
RG	4
DW	48.400 usec
DE	10.00 usec
TE	298.0 K
D1	1.00000000 sec
TD0	1

======== CHANNEL f1 ========

SFO1	500.3030896 MHz
NUC1	1H
P1	15.00 usec
SI	65536
SF	500.3000133 MHz
WDW	EM
SSB	0
LB	0.30 Hz
GB	0
PC	1.00

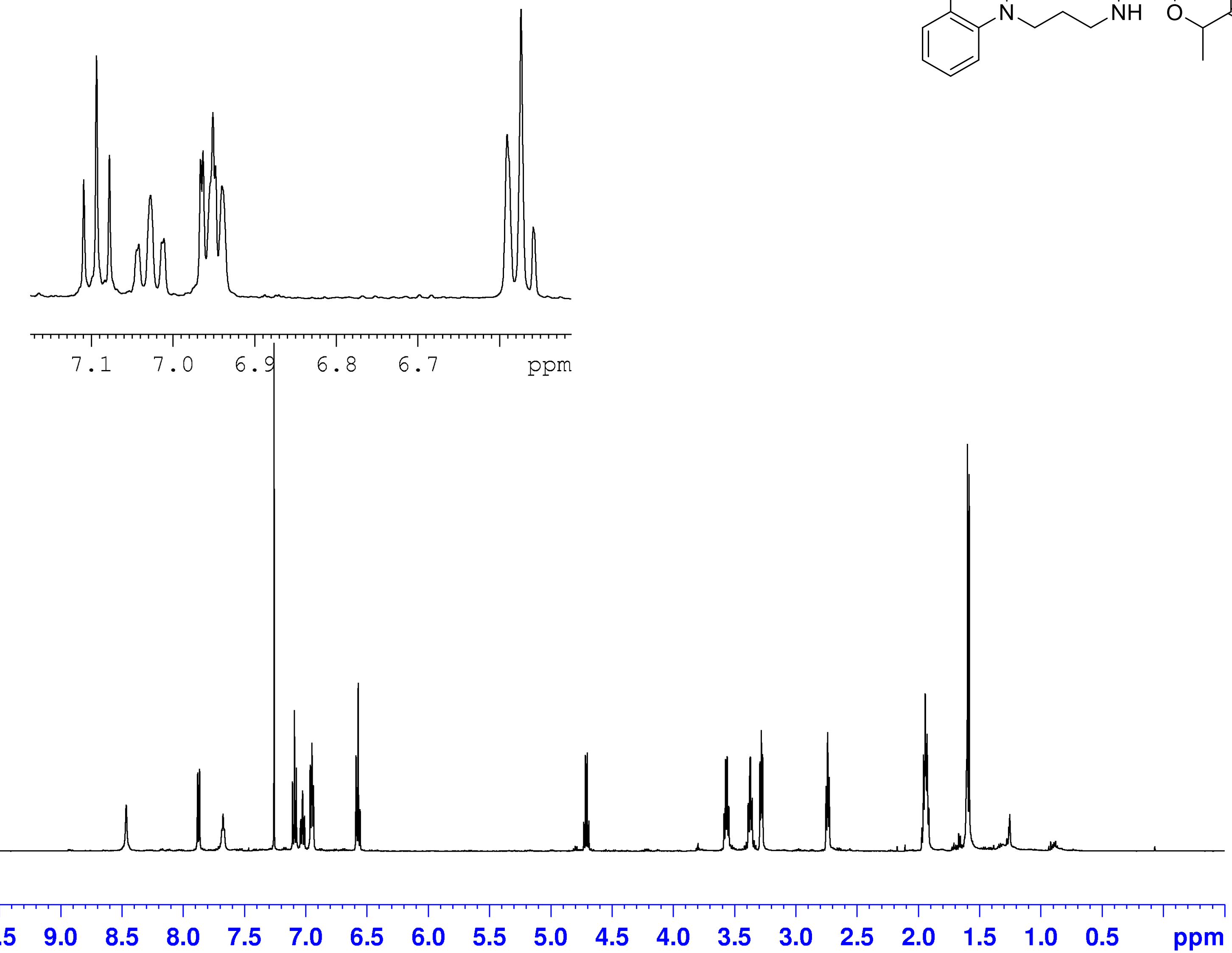

N-[2-(3,4-Dihydroquinolin-1(2*H*)-yl)propyl]-2-methyl-3-oxo-3,4-dihydro-2*H*-1,4-benzoxazine-8-carboxamide **4** ^{13}C-NMR

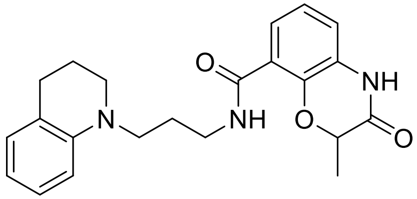

NAME	tr1062	
EXPNO	4	
PROCNO	1	
Date_	20131113	
Time	10.48	
INSTRUM	avc500	
PROBHD	5 mm CPDUL 13C	
PULPROG	zgpg30	
TD	65536	
SOLVENT	CDCl3	
NS	512	
DS	2	
SWH	31250.000	Hz
FIDRES	0.476837	Hz
AQ	1.0486259	sec
RG	912	
DW	16.000	usec
DE	18.00	usec
TE	298.0	K
D1	2.00000000	sec
D11	0.03000000	sec
TD0	1	
======== CHANNEL f1 ========		
SFO1	125.8131151	MHz
NUC1	13C	
P1	10.00	usec
SI	32768	
SF	125.8005192	MHz
WDW	EM	
SSB	0	
LB	1.00	Hz
GB	0	
PC	1.40	

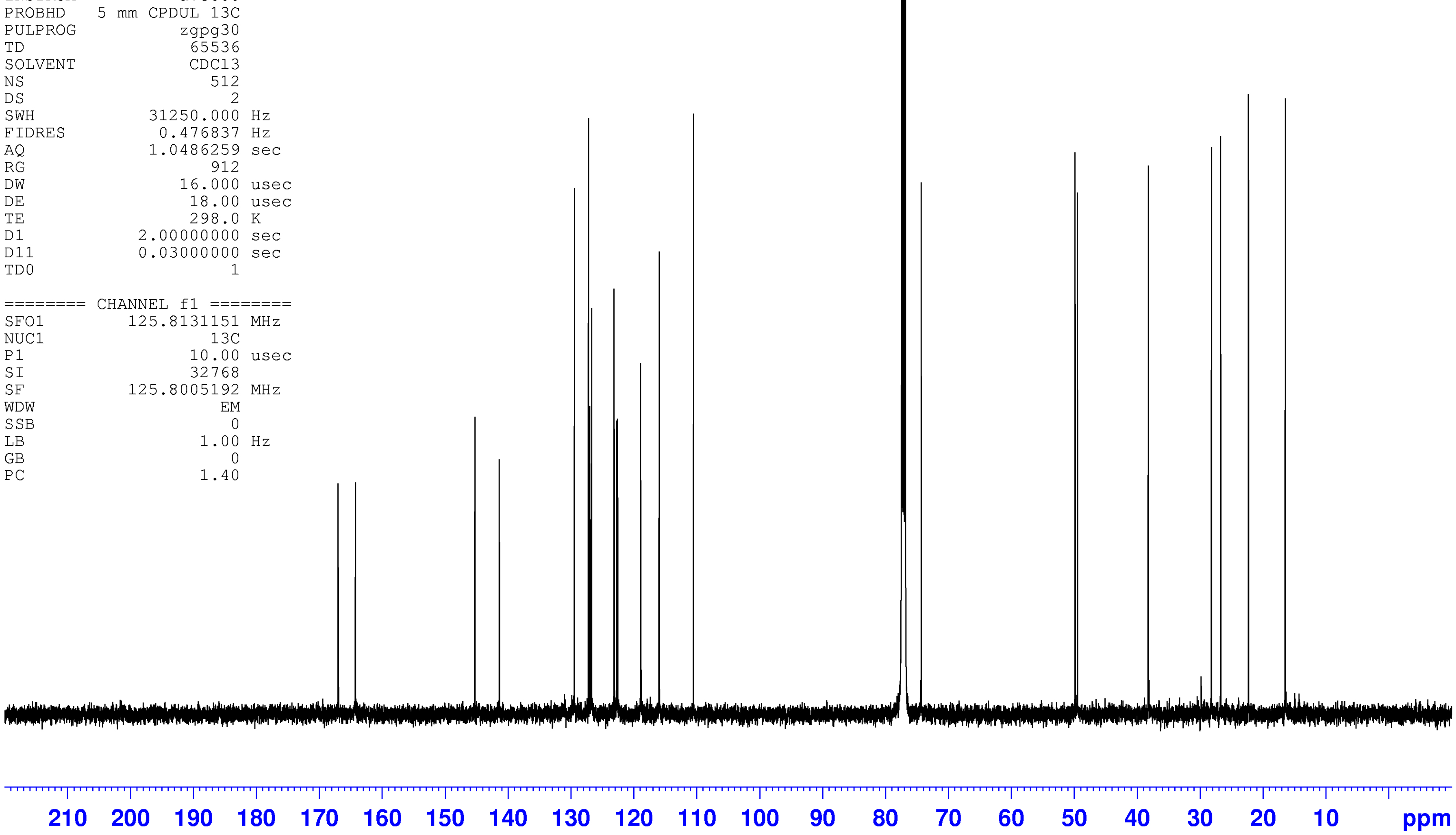

N-benzyl-2-methyl-3-oxo-3,4-dihydro-2*H*-1,4-benzoxazine-8-carboxamide **9** ^{1}H NMR

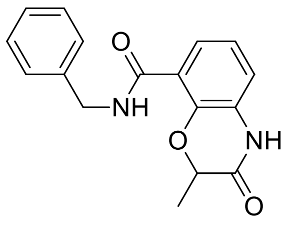


```
NAME              tr1020
EXPNO                  1
PROCNO                 1
Date_           20131124
Time               19.43
INSTRUM           avg400
PROBHD   5 mm QNP 1H/13
PULPROG             zg30
TD                 65536
SOLVENT             DMSO
NS                    16
DS                     2
SWH            10000.000 Hz
FIDRES          0.152588 Hz
AQ             3.2768500 sec
RG                359.87
DW                50.000 usec
DE                  6.50 usec
TE                 294.9 K
D1            1.00000000 sec
TD0                    1

======== CHANNEL f1 ========
SFO1         400.2024714 MHz
NUC1                  1H
P1                 12.23 usec
SI                 65536
SF           400.2000067 MHz
WDW                   EM
SSB                    0
LB                  0.30 Hz
GB                     0
PC                  1.00
```

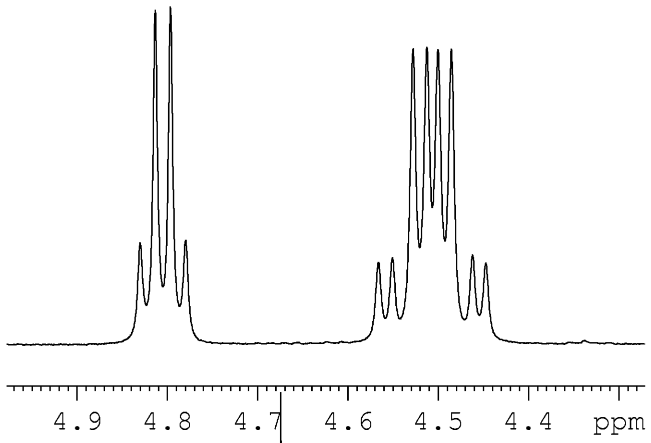

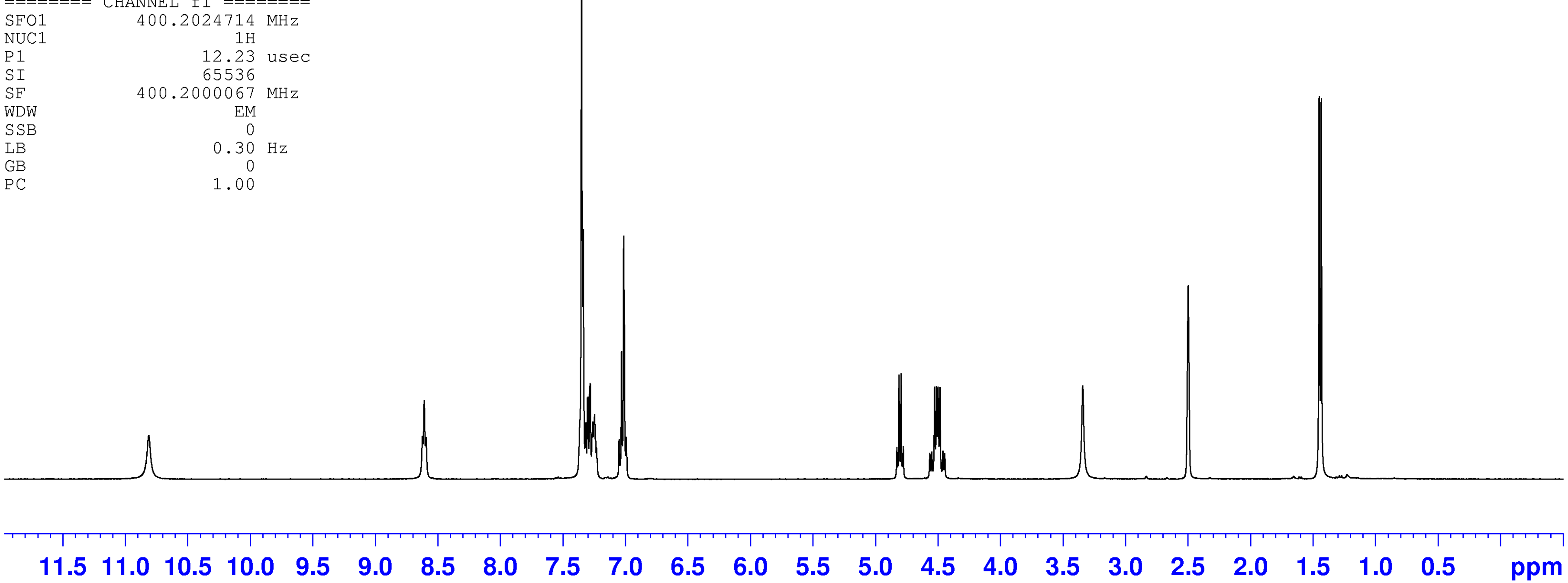

N-benzyl-2-methyl-3-oxo-3,4-dihydro-2*H*-1,4-benzoxazine-8-carboxamide **9** ^{13}C NMR

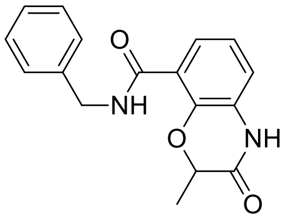

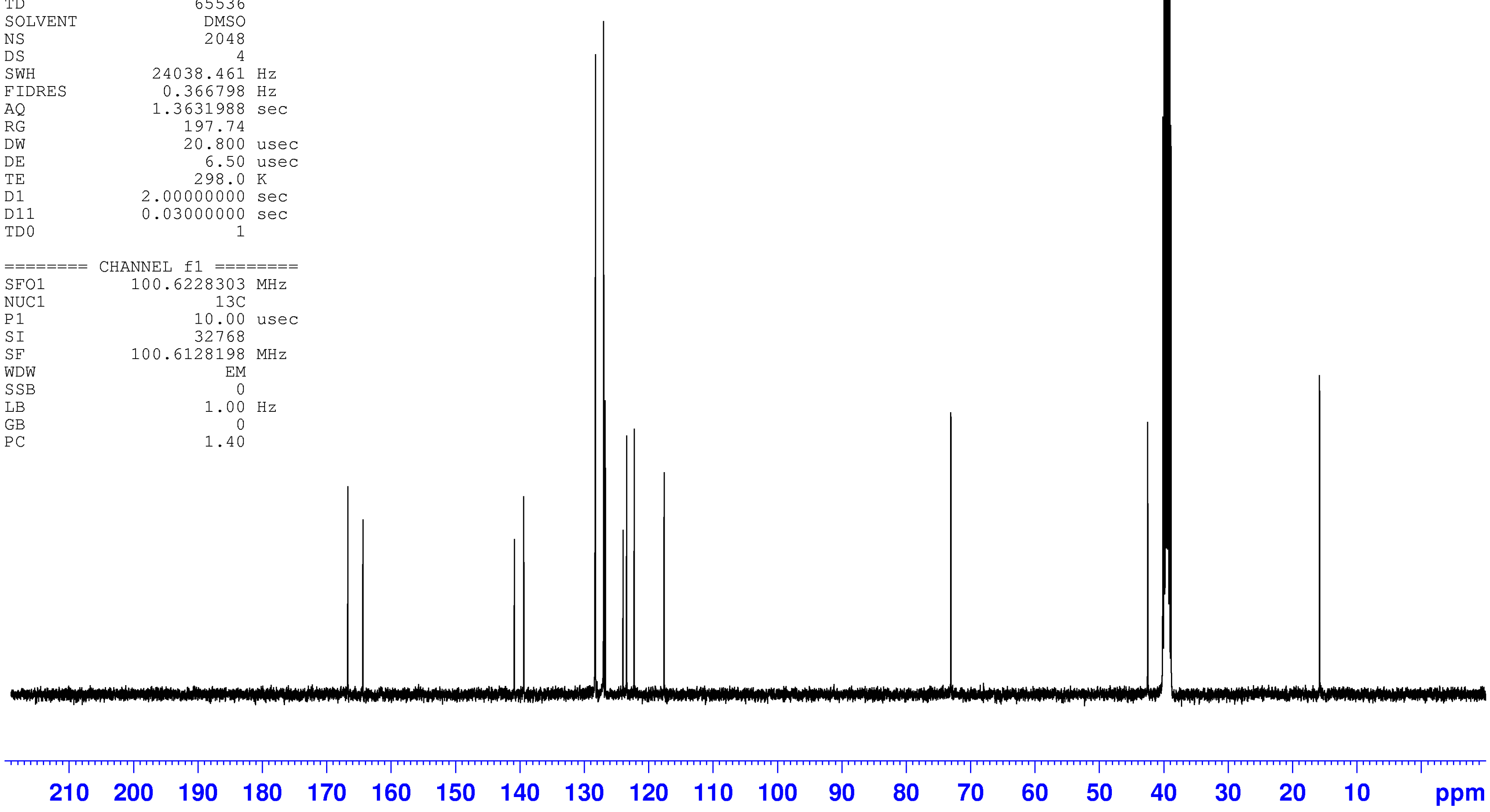

Methyl 2-{[(2*S*)-1-methoxy-1-oxopropan-2-yl]oxy}-3-nitrobenzoate (*S*)-**51** ^{1}H NMR

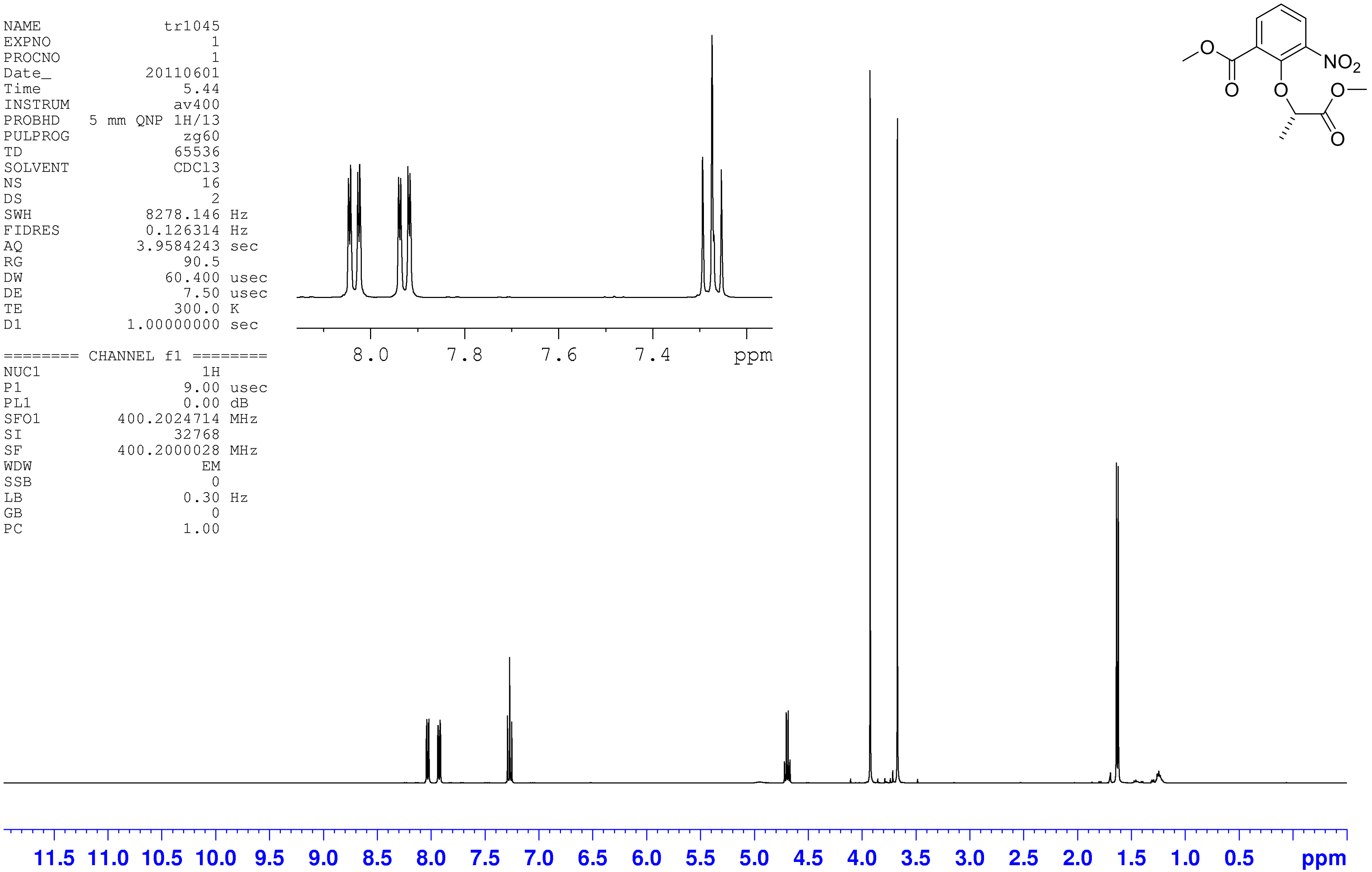

Methyl 2-{[(2*S*)-1-methoxy-1-oxopropan-2-yl]oxy}-3-nitrobenzoate (*S*)-**51** ^{13}C NMR

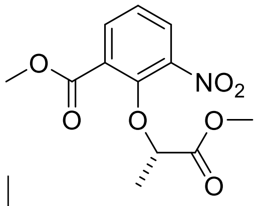


```
NAME              tr1045
EXPNO                  2
PROCNO                 1
Date_           20110601
Time                5.52
INSTRUM            av400
PROBHD   5 mm QNP 1H/13
PULPROG          zgpg30
TD                 32768
SOLVENT            CDCl3
NS                   256
DS                     4
SWH            26178.010 Hz
FIDRES          0.798889 Hz
AQ             0.6259188 sec
RG                 32768
DW                19.100 usec
DE                  7.50 usec
TE                 300.0 K
D1            1.00000000 sec
D11           0.03000000 sec
TD0                    1

======== CHANNEL f1 ========
NUC1                 13C
P1                  9.50 usec
PL1                 0.00 dB
SFO1         100.6403931 MHz

======== CHANNEL f2 ========
CPDPRG2          waltz16
NUC2                  1H
PCPD2              80.00 usec
PL2                 0.00 dB
PL12               19.00 dB
PL13               25.00 dB
SFO2         400.2016008 MHz
SI                 32768
SF           100.6303718 MHz
WDW                   EM
SSB                    0
LB                  1.00 Hz
GB                     0
PC                  1.40
```

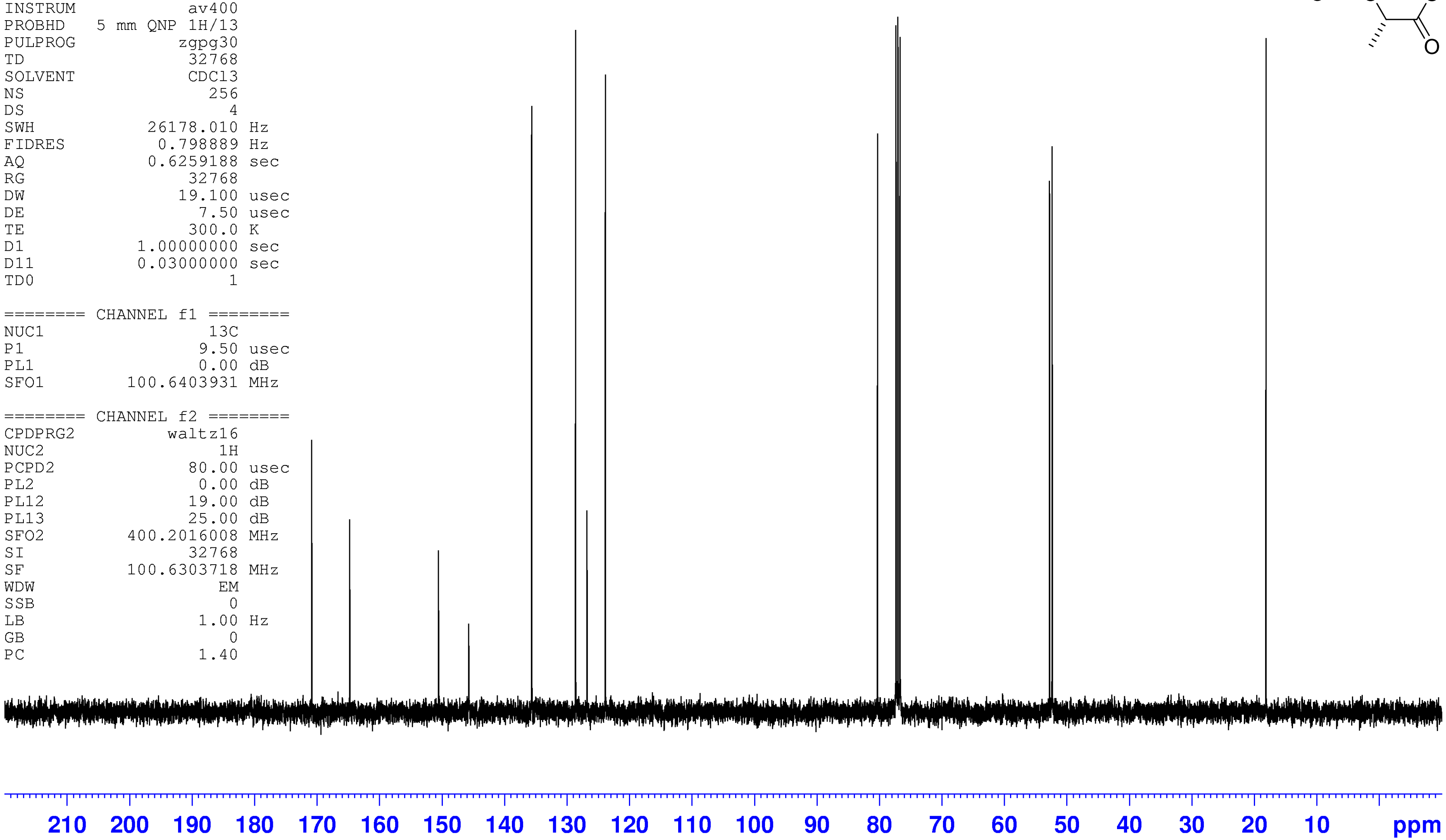

Benzyl 2-benzyloxy-3-nitrobenzoate **53** ^{1}H NMR

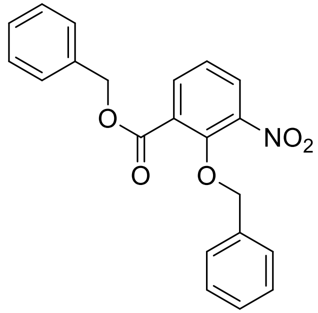

NAME	tr3050
EXPNO	1
PROCNO	1
Date_	20120805
Time	17.18
INSTRUM	av400
PROBHD	5 mm QNP 1H/13
PULPROG	zg60
TD	65536
SOLVENT	CDCl3
NS	16
DS	2
SWH	8278.146 Hz
FIDRES	0.126314 Hz
AQ	3.9584243 sec
RG	35.9
DW	60.400 usec
DE	7.50 usec
TE	300.0 K
D1	1.00000000 sec
======== CHANNEL f1 ========	
NUC1	1H
P1	9.00 usec
PL1	0.00 dB
SFO1	400.2024714 MHz
SI	32768
SF	400.2000028 MHz
WDW	EM
SSB	0
LB	0.30 Hz
GB	0
PC	1.00

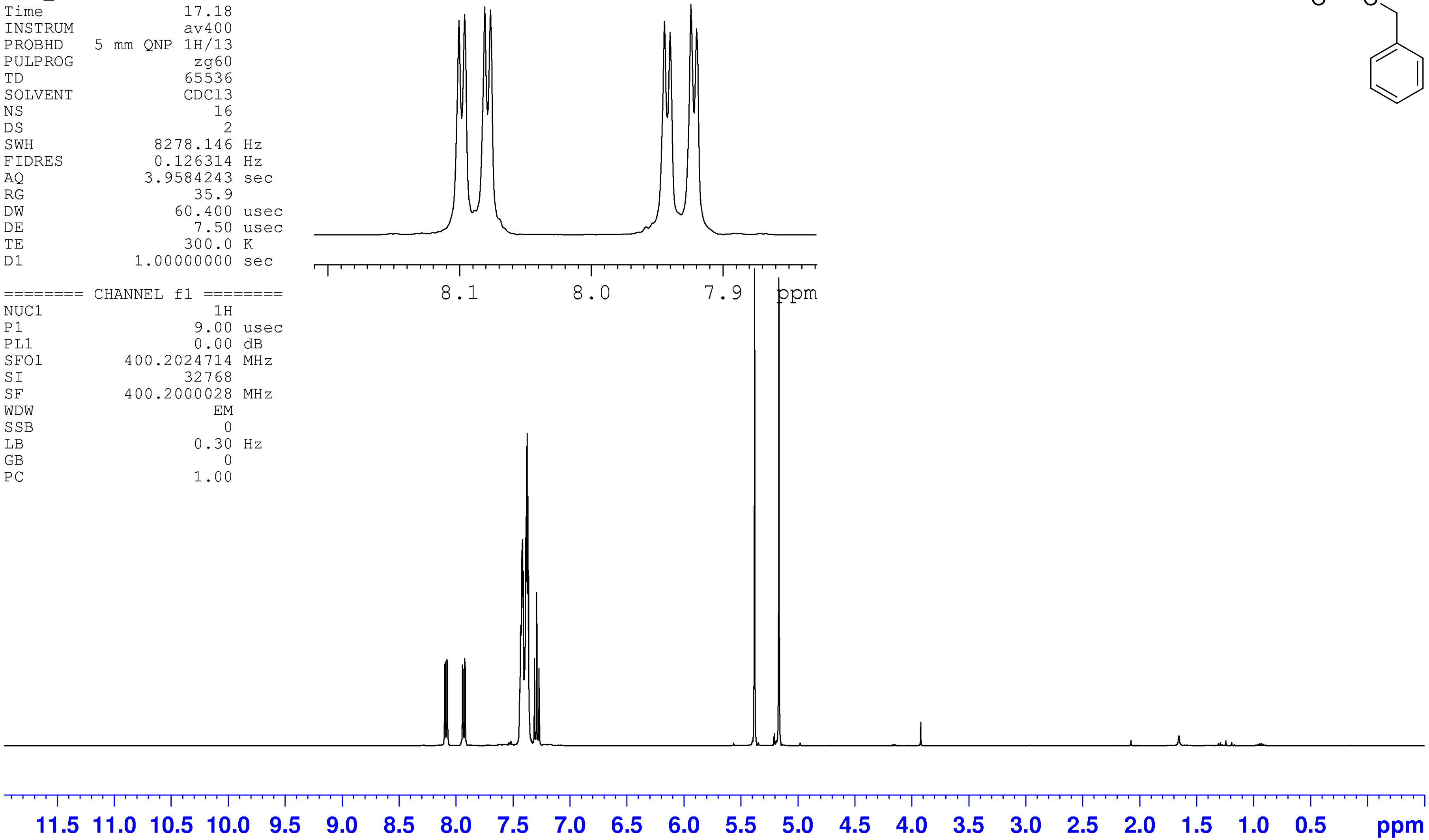

Benzyl 2-benzyloxy-3-nitrobenzoate **53** ^{13}C NMR

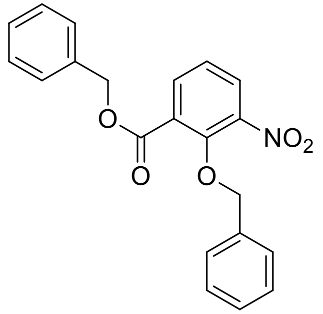

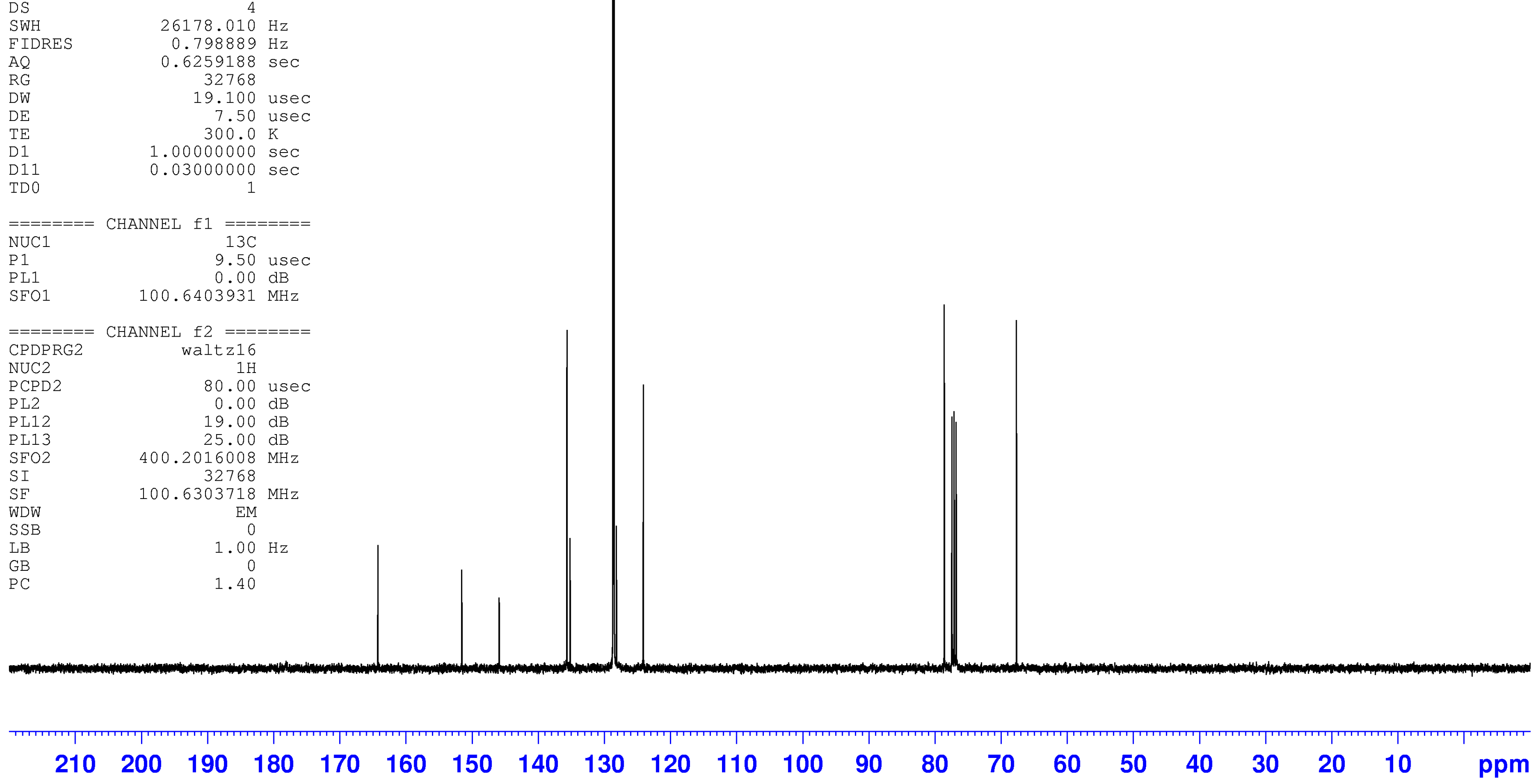

Benzyl 3-nitro-2-hydroxybenzoate **54** ^{1}H NMR

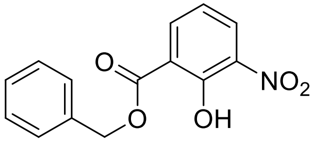

NAME	tr1072
EXPNO	1
PROCNO	1
Date_	20110709
Time	16.46
INSTRUM	avc500
PROBHD	5 mm CPDUL 13C
PULPROG	zg30
TD	65536
SOLVENT	CDCl3
NS	16
DS	2
SWH	10330.578 Hz
FIDRES	0.157632 Hz
AQ	3.1719923 sec
RG	4
DW	48.400 usec
DE	6.00 usec
TE	298.0 K
D1	1.00000000 sec
TD0	1

======== CHANNEL f1 ========

NUC1	1H
P1	9.60 usec
PL1	-6.00 dB
PL1W	15.19999981 W
SFO1	500.3030896 MHz
SI	32768
SF	500.3000240 MHz
WDW	EM
SSB	0
LB	0.30 Hz
GB	0
PC	1.00

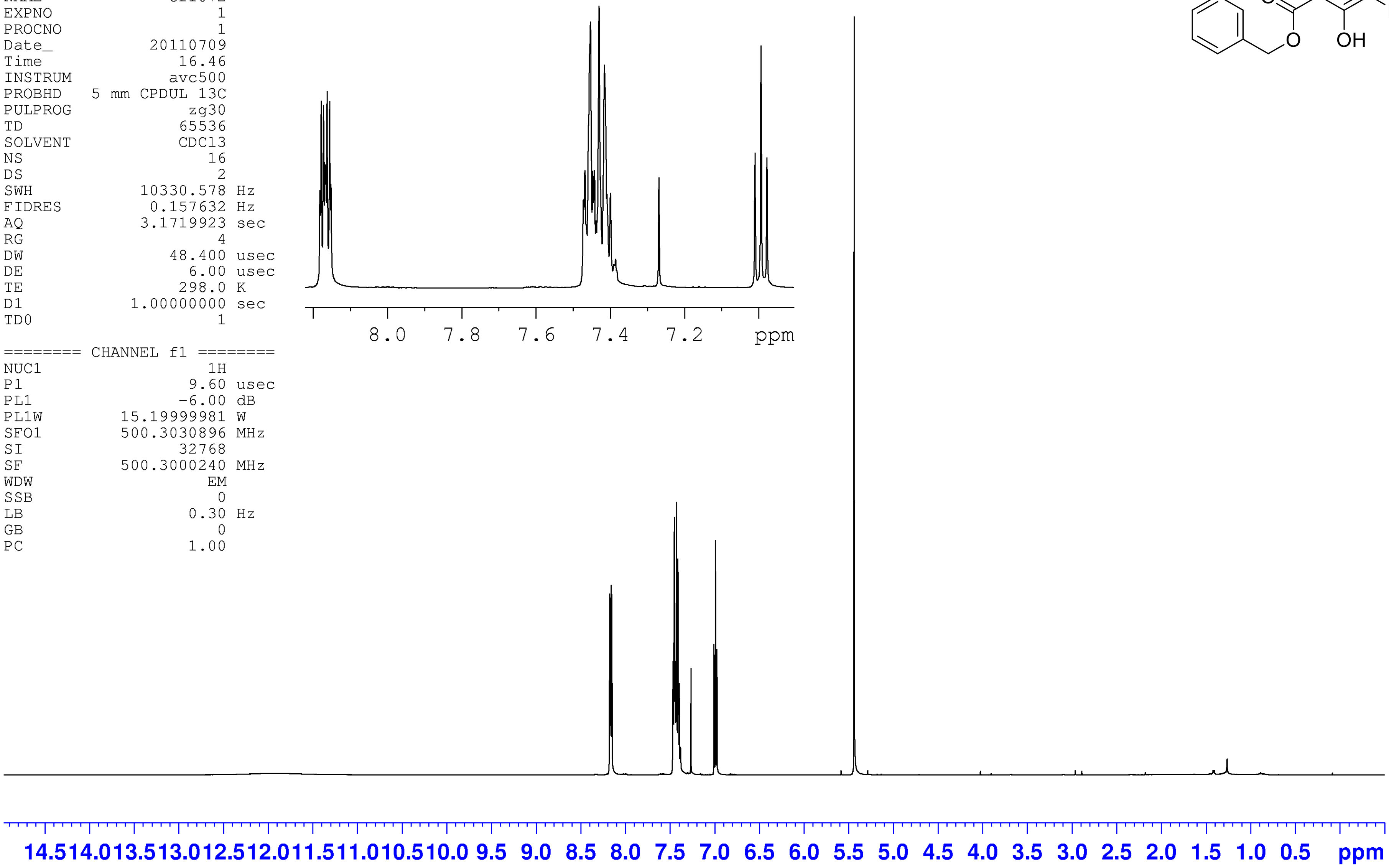

Benzyl 3-nitro-2-hydroxybenzoate **54** ^{13}C NMR

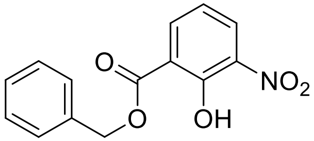


```
NAME              tr1072
EXPNO                  4
PROCNO                 1
Date_           20110709
Time               17.30
INSTRUM           avc500
PROBHD   5 mm CPDUL 13C
PULPROG          zgpg30
TD                 65536
SOLVENT            CDCl3
NS                   512
DS                     2
SWH            31250.000 Hz
FIDRES          0.476837 Hz
AQ             1.0486259 sec
RG                  1820
DW                16.000 usec
DE                 20.00 usec
TE                 298.0 K
D1            2.00000000 sec
D11           0.03000000 sec
TD0                    1

======== CHANNEL f1 ========
NUC1                 13C
P1                  9.10 usec
PL1                -4.40 dB
PL1W         28.15752029 W
SFO1         125.8131151 MHz

======== CHANNEL f2 ========
CPDPRG2          waltz16
NUC2                  1H
PCPD2              80.00 usec
PL2                -6.00 dB
PL12               12.42 dB
PL13               18.42 dB
PL2W         15.19999981 W
PL12W         0.21869738 W
PL13W         0.05493430 W
SFO2         500.3020012 MHz
SI                 32768
SF           125.8005438 MHz
WDW                   EM
SSB                    0
LB                  1.00 Hz
GB                     0
PC                  1.40
```

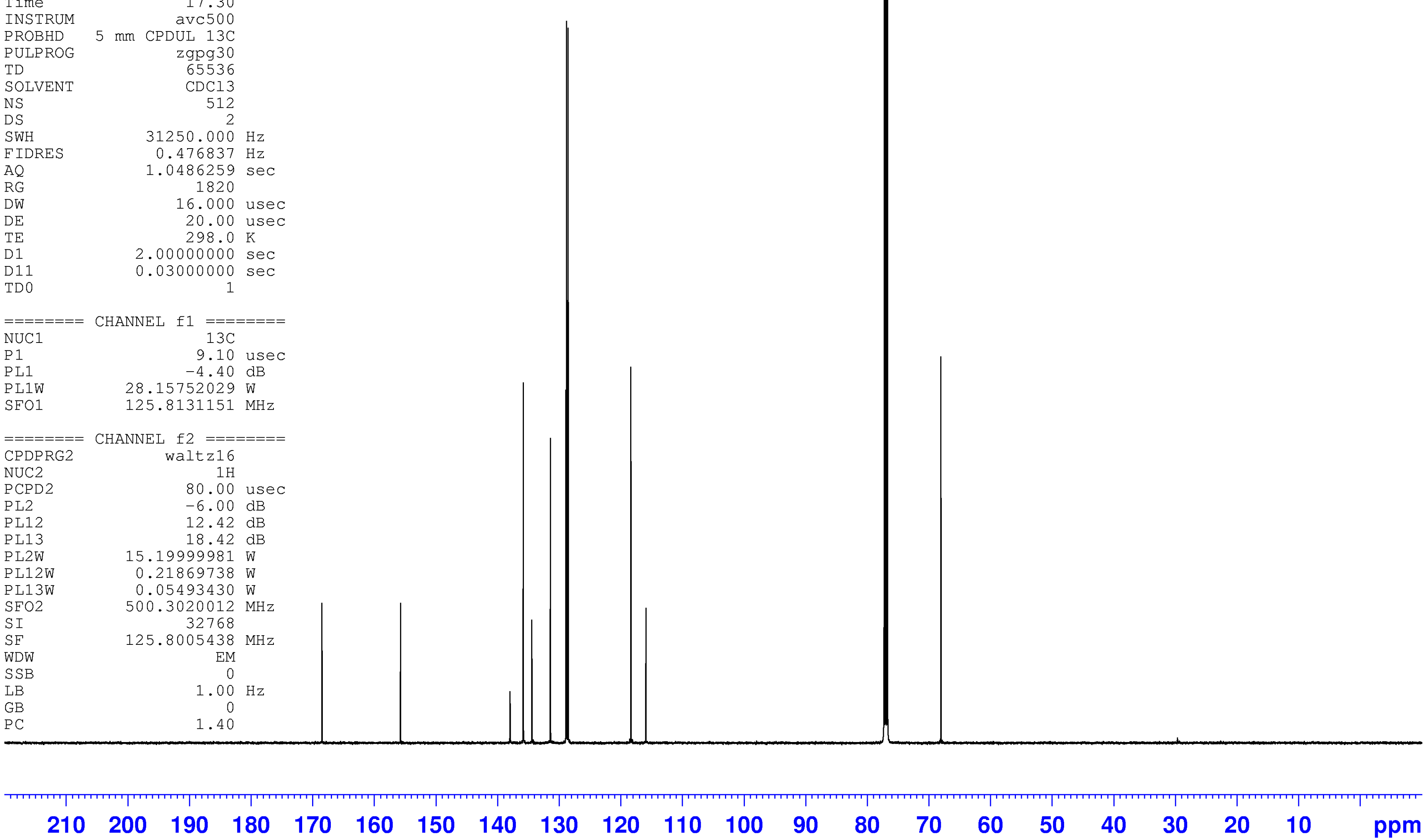

Benzyl 3-nitro-2-{[(trifluoromethyl)sulfonyl]oxy}benzoate **55** ^{1}H NMR

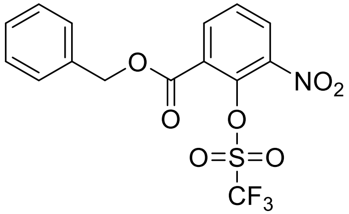

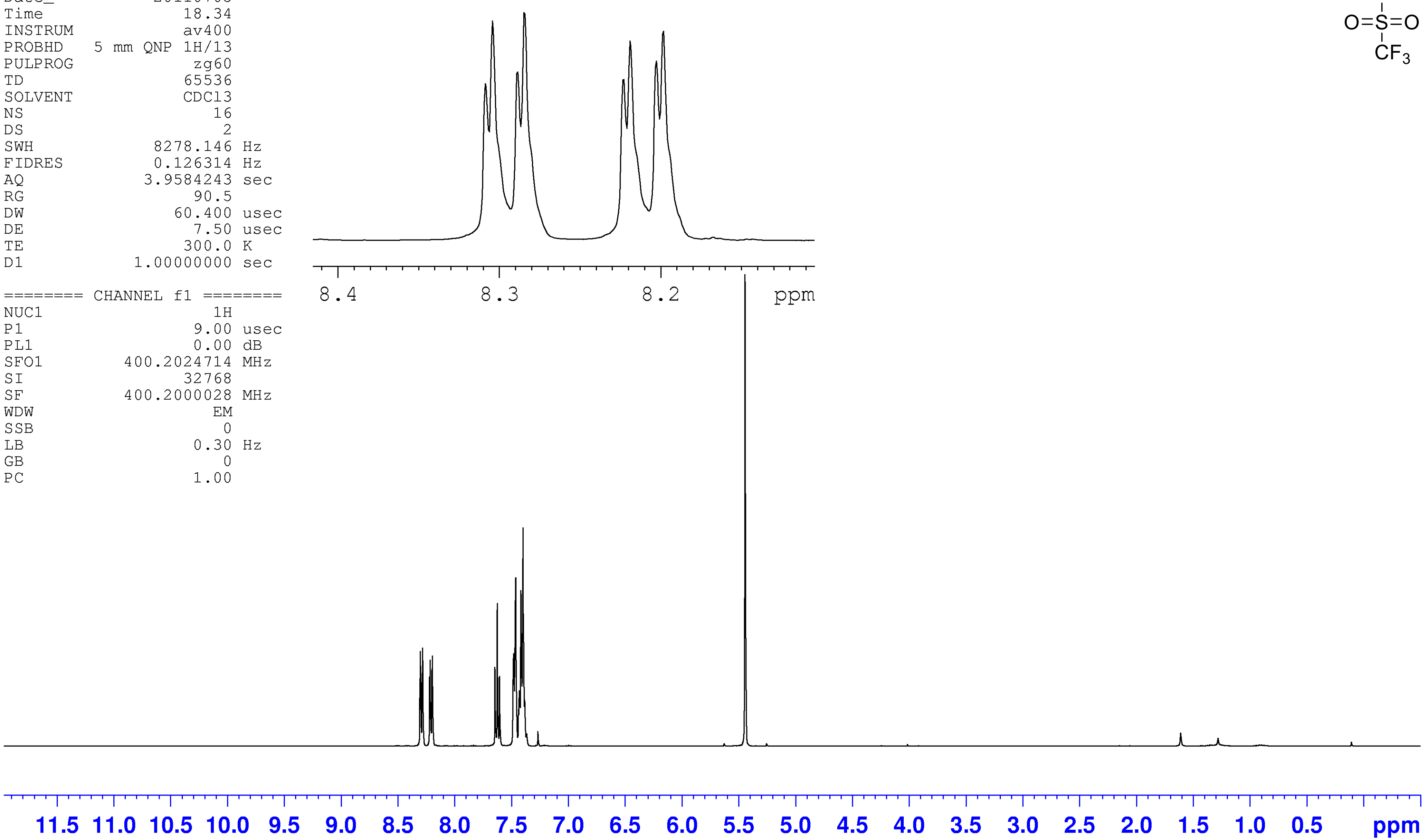

Benzyl 3-nitro-2-{[(trifluoromethyl)sulfonyl]oxy}benzoate **55** ^{13}C NMR

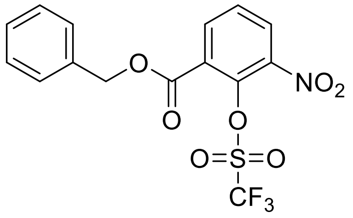


```
NAME              tr1084
EXPNO                  3
PROCNO                 1
Date_           20110705
Time               18.44
INSTRUM            av400
PROBHD   5 mm QNP 1H/13
PULPROG          zgpg30
TD                 32768
SOLVENT            CDCl3
NS                   256
DS                     4
SWH            26178.010 Hz
FIDRES          0.798889 Hz
AQ             0.6259188 sec
RG                 32768
DW                19.100 usec
DE                  7.50 usec
TE                 300.0 K
D1            1.00000000 sec
D11           0.03000000 sec
TD0                    1

======== CHANNEL f1 ========
NUC1                 13C
P1                  9.50 usec
PL1                 0.00 dB
SFO1         100.6403931 MHz

======== CHANNEL f2 ========
CPDPRG2          waltz16
NUC2                  1H
PCPD2              80.00 usec
PL2                 0.00 dB
PL12               19.00 dB
PL13               25.00 dB
SFO2         400.2016008 MHz
SI                 32768
SF           100.6303718 MHz
WDW                   EM
SSB                    0
LB                  1.00 Hz
GB                     0
PC                  0.11
```

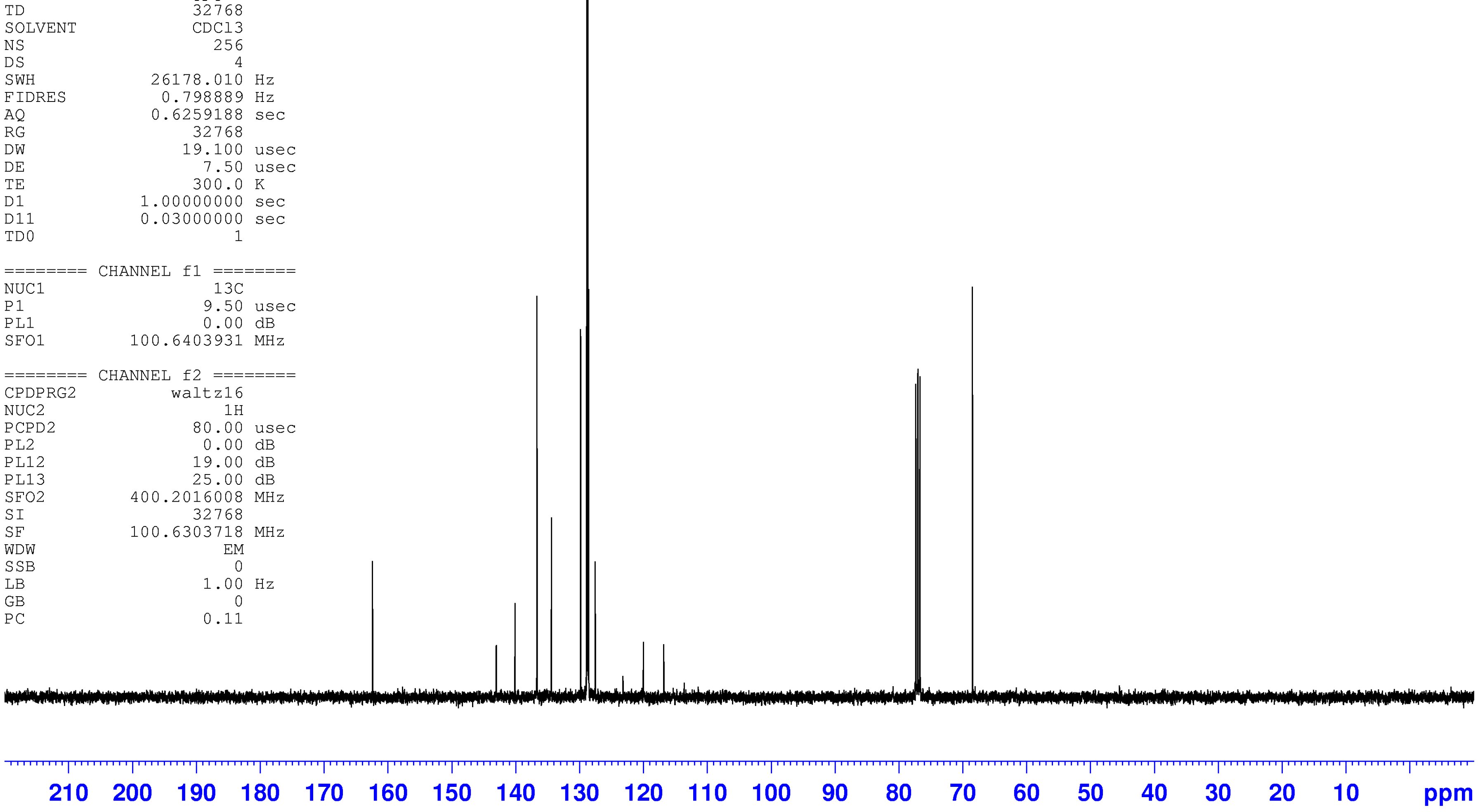

Benzyl 3-nitro-2-{[(trifluoromethyl)sulfonyl]oxy}benzoate **55** ^{19}F NMR

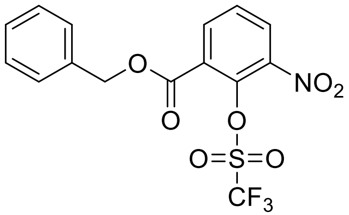

NAME	tr1084	
EXPNO	2	
PROCNO	1	
Date_	20110705	
Time	18.36	
INSTRUM	av400	
PROBHD	5 mm QNP 1H/13	
PULPROG	zgflqn	
TD	131072	
SOLVENT	CDCl3	
NS	16	
DS	2	
SWH	75187.969	Hz
FIDRES	0.573639	Hz
AQ	0.8716788	sec
RG	71.8	
DW	6.650	usec
DE	9.50	usec
TE	300.0	K
D1	1.00000000	sec
TD0	1	

======== CHANNEL f1 ========		
NUC1	19F	
P1	15.75	usec
PL1	0.00	dB
SFO1	376.5077354	MHz
SI	65536	
SF	376.5642200	MHz
WDW	EM	
SSB	0	
LB	0.30	Hz
GB	0	
PC	1.00	

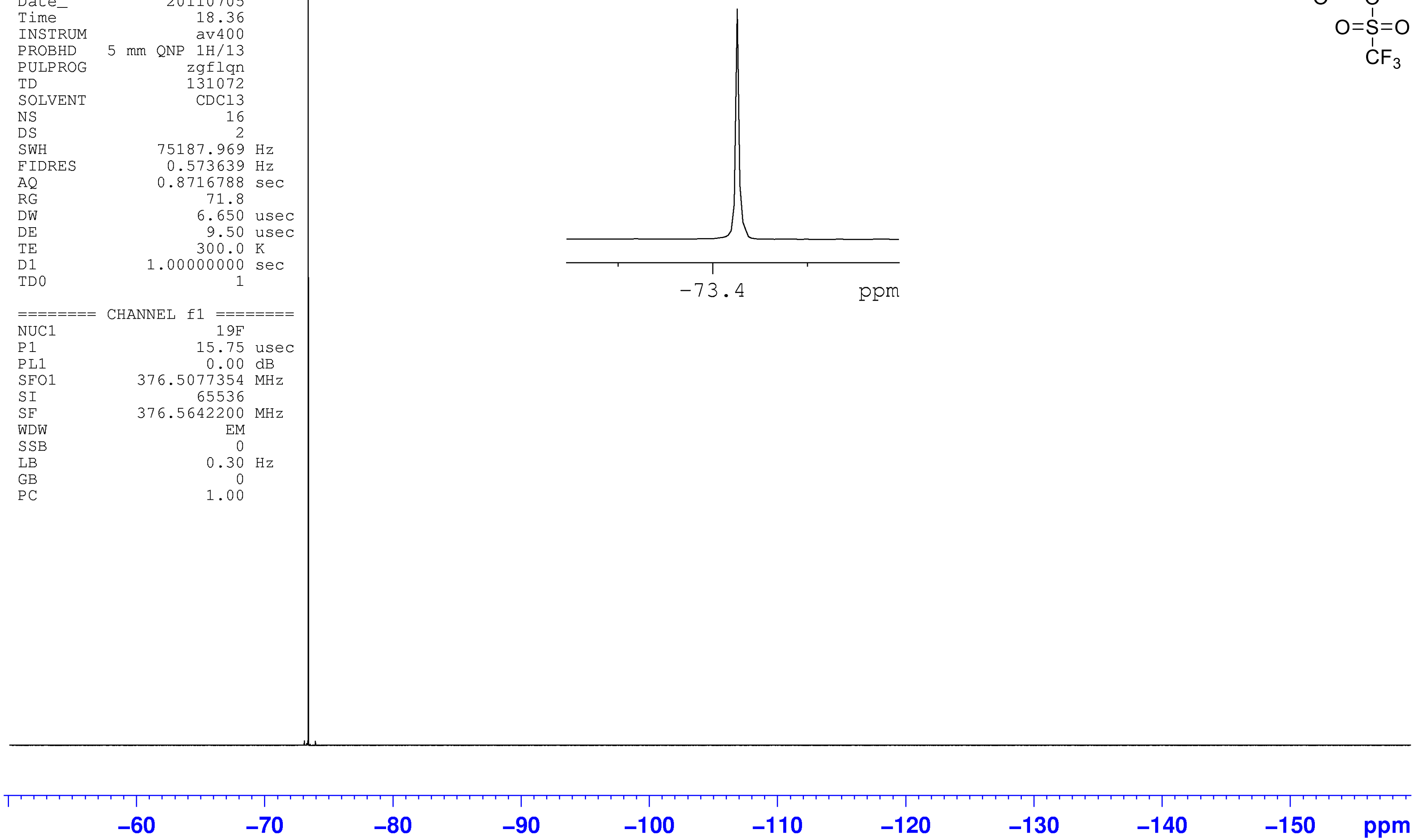

Methyl 3-nitro-2-{[(trifluoromethyl)sulfonyl]oxy}benzoate **56** ^{1}H NMR

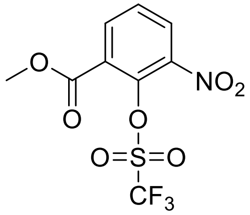


```
NAME              tr1038
EXPNO                  5
PROCNO                 1
Date_           20110601
Time               18.08
INSTRUM            av400
PROBHD    5 mm QNP 1H/13
PULPROG             zg60
TD                 65536
SOLVENT            CDCl3
NS                    16
DS                     2
SWH             8278.146 Hz
FIDRES          0.126314 Hz
AQ             3.9584243 sec
RG                   181
DW                60.400 usec
DE                  7.50 usec
TE                 300.0 K
D1            1.00000000 sec

======== CHANNEL f1 ========
NUC1                  1H
P1                  9.00 usec
PL1                 0.00 dB
SFO1         400.2024714 MHz
SI                 32768
SF           400.2000028 MHz
WDW                   EM
SSB                    0
LB                  0.30 Hz
GB                     0
PC                  1.00
```

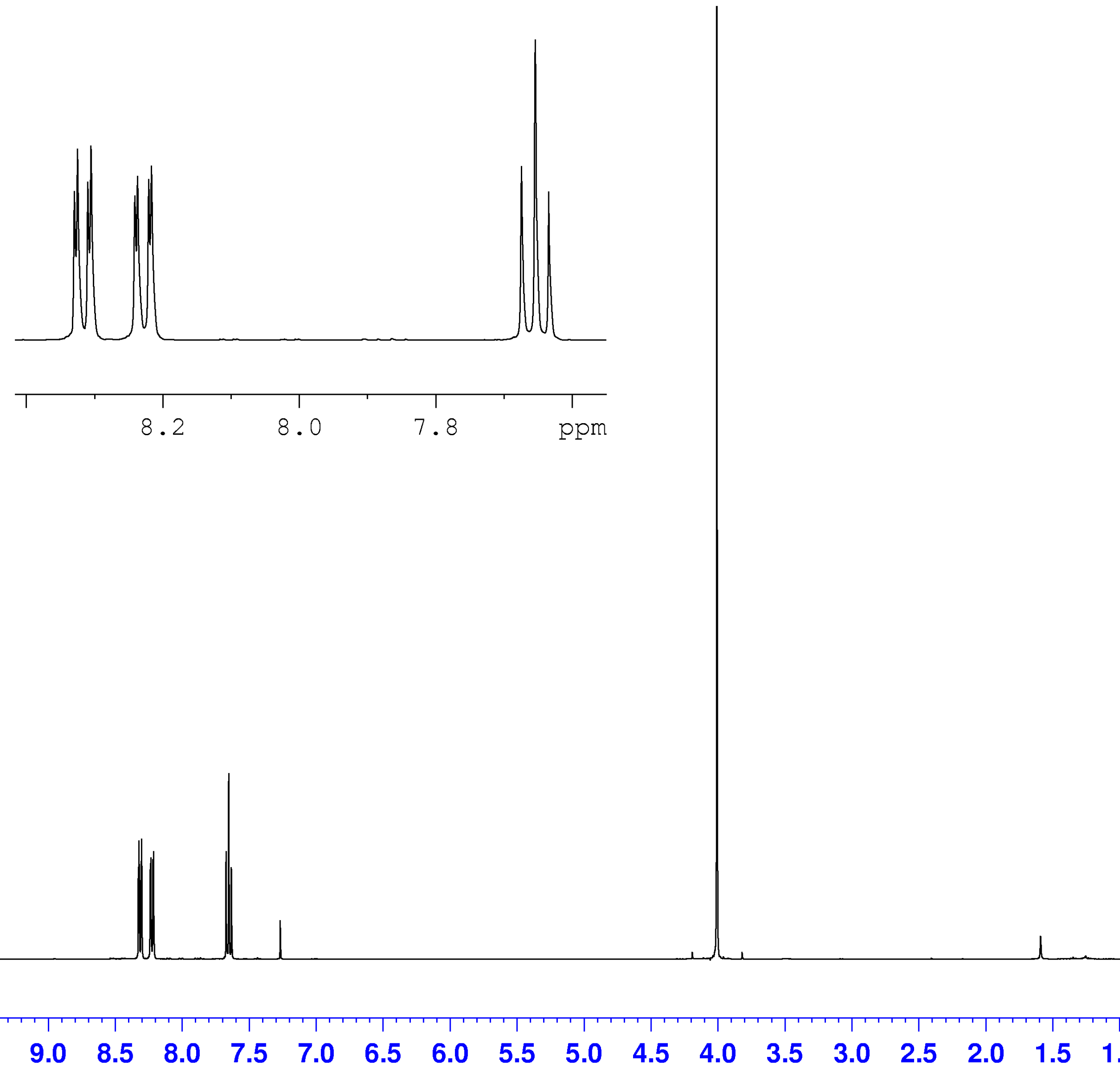

Methyl 3-nitro-2-{[(trifluoromethyl)sulfonyl]oxy}benzoate **56** ^{13}C NMR

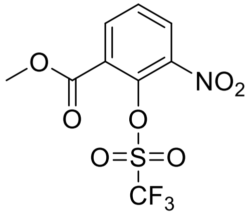


```
NAME               tr1038
EXPNO                   7
PROCNO                  1
Date_            20110601
Time                18.40
INSTRUM             av400
PROBHD   5 mm QNP 1H/13
PULPROG           zgpg30
TD                  32768
SOLVENT             CDCl3
NS                    256
DS                      4
SWH             26178.010 Hz
FIDRES           0.798889 Hz
AQ              0.6259188 sec
RG                  32768
DW                 19.100 usec
DE                   7.50 usec
TE                  300.0 K
D1             1.00000000 sec
D11            0.03000000 sec
TD0                     1

======== CHANNEL f1 ========
NUC1                  13C
P1                   9.50 usec
PL1                  0.00 dB
SFO1          100.6403931 MHz

======== CHANNEL f2 ========
CPDPRG2           waltz16
NUC2                   1H
PCPD2               80.00 usec
PL2                  0.00 dB
PL12                19.00 dB
PL13                25.00 dB
SFO2          400.2016008 MHz
SI                  32768
SF            100.6303718 MHz
WDW                    EM
SSB                     0
LB                   1.00 Hz
GB                      0
PC                   1.20
```

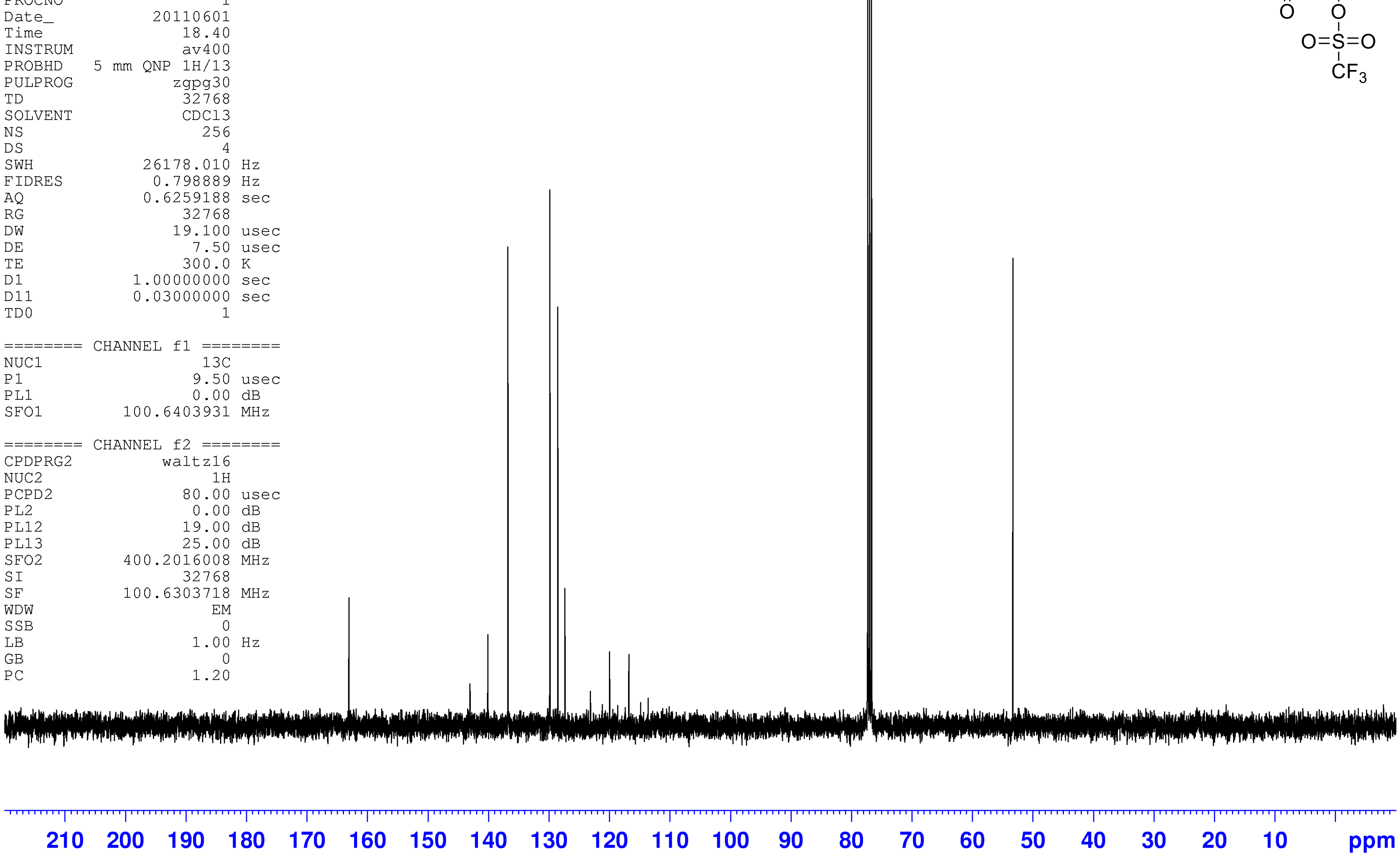

Methyl 3-nitro-2-{[(trifluoromethyl)sulfonyl]oxy}benzoate **56** ^{19}F NMR

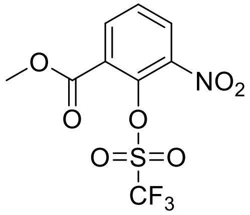

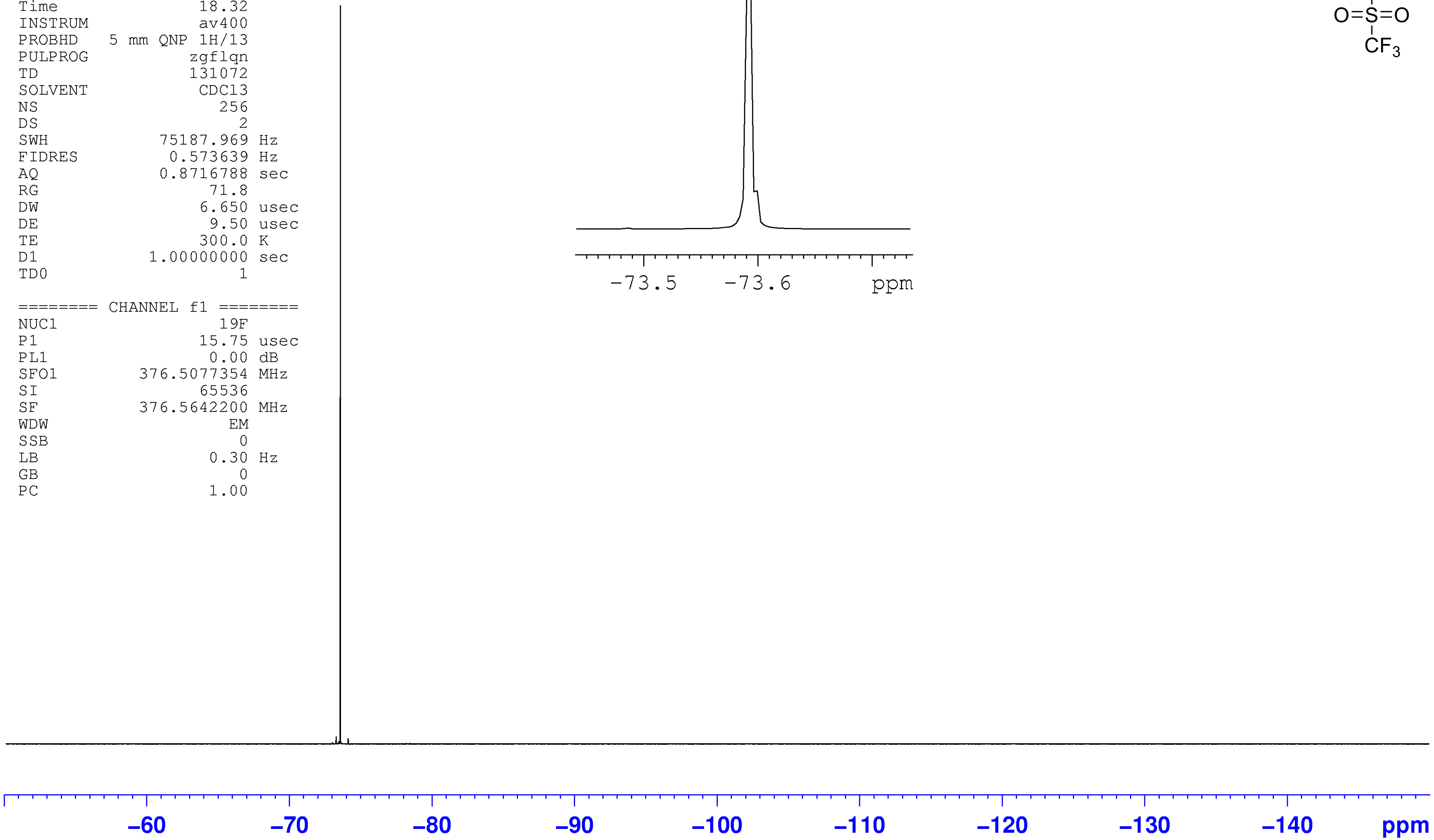

Methyl 2-[(1-methoxy-1-oxopropan-2-yl)amino]-3-nitrobenzoate **57** ^{1}H NMR

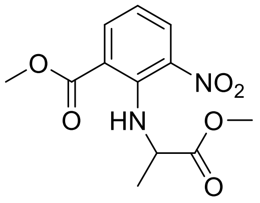

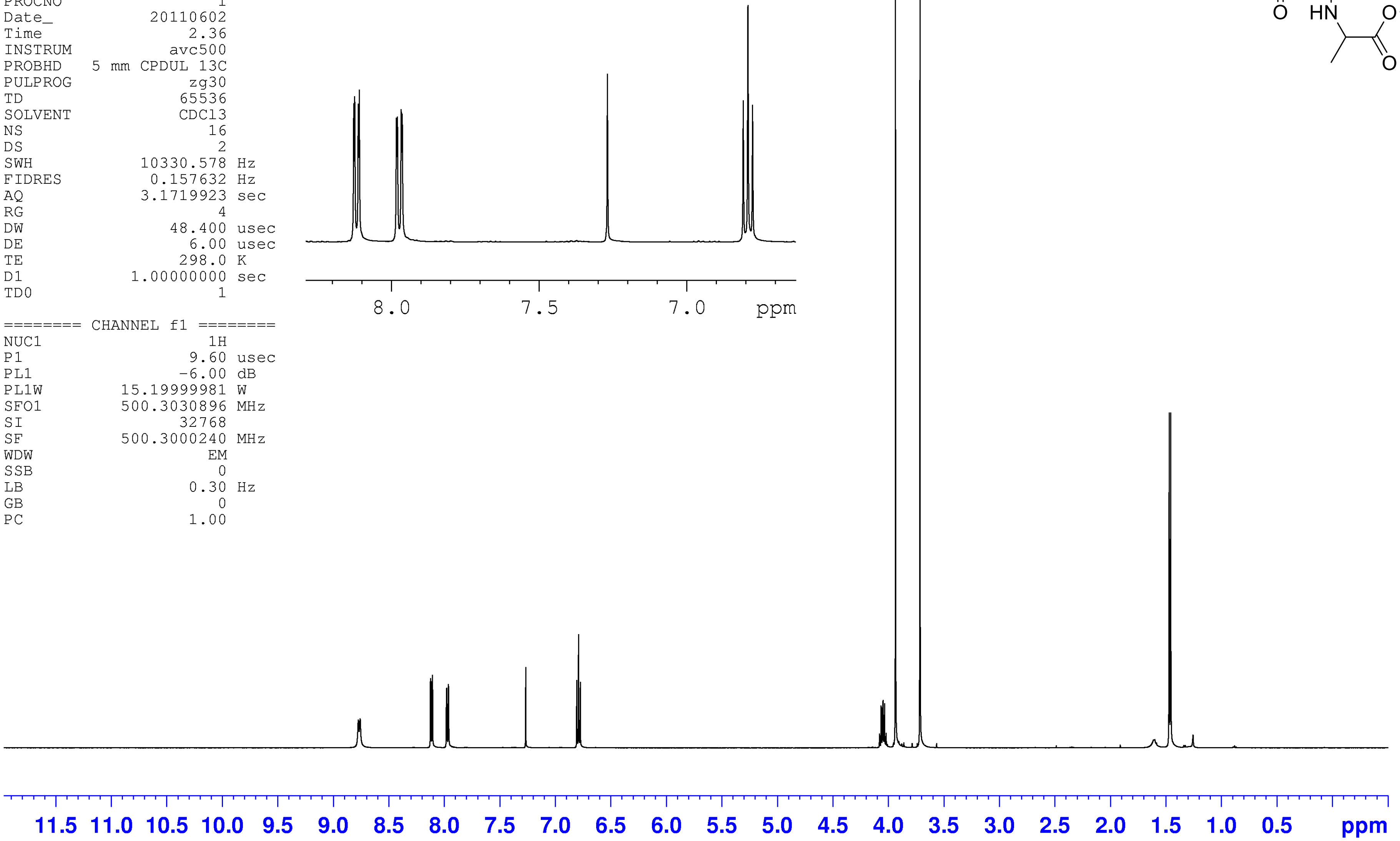

Methyl 2-[(1-methoxy-1-oxopropan-2-yl)amino]-3-nitrobenzoate **57** ^{13}C NMR

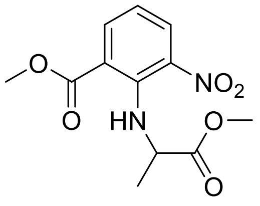

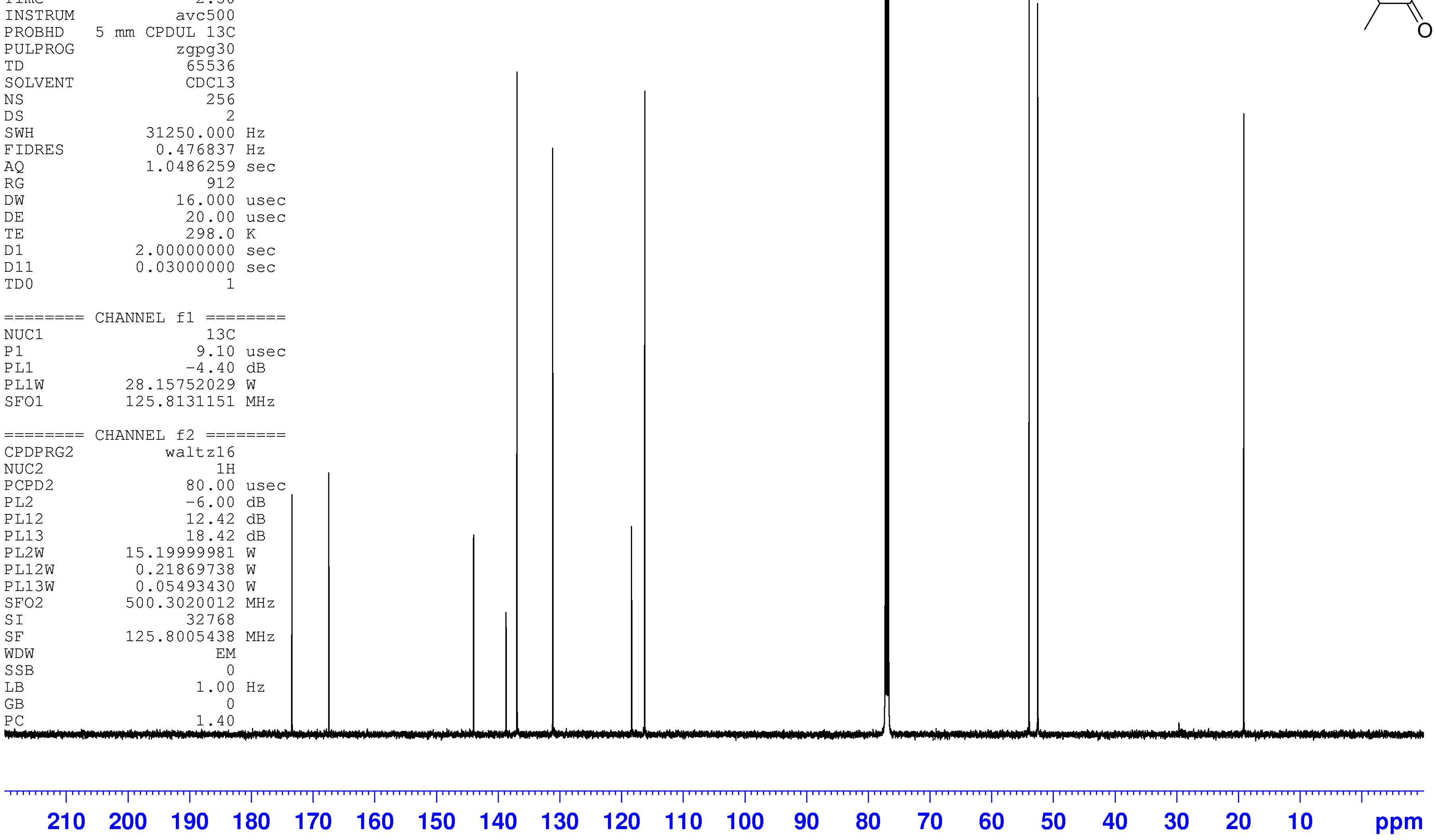

Methyl 3-methyl-2-oxo-1,2,3,4-tetrahydroquinoxaline-5-carboxylate **58** ^{1}H NMR

NAME	tr1053	
EXPNO	1	
PROCNO	1	
Date_	20110519	
Time	9.50	
INSTRUM	avc500	
PROBHD	5 mm CPDUL 13C	
PULPROG	zg30	
TD	65536	
SOLVENT	CDCl3	
NS	16	
DS	2	
SWH	10330.578	Hz
FIDRES	0.157632	Hz
AQ	3.1719923	sec
RG	4	
DW	48.400	usec
DE	6.00	usec
TE	298.0	K
D1	1.00000000	sec
TD0	1	
======== CHANNEL f1 ========		
NUC1	1H	
P1	9.60	usec
PL1	-6.00	dB
PL1W	15.19999981	W
SFO1	500.3030896	MHz
SI	32768	
SF	500.3000240	MHz
WDW	EM	
SSB	0	
LB	0.30	Hz
GB	0	
PC	1.00	

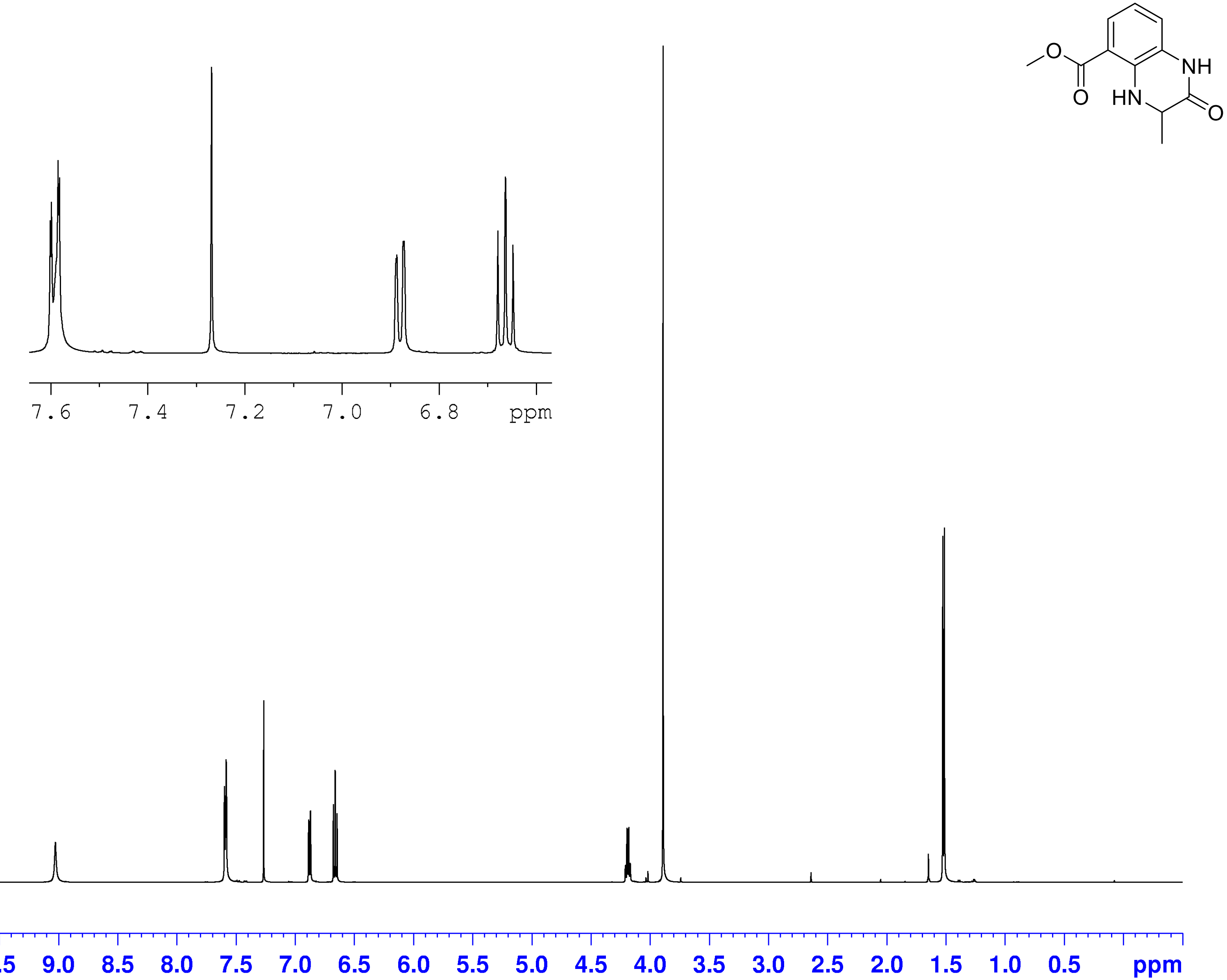

Methyl 3-methyl-2-oxo-1,2,3,4-tetrahydroquinoxaline-5-carboxylate **58** ^{13}C NMR

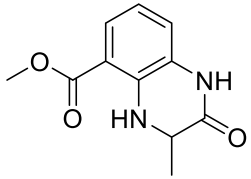


```
NAME              tr1053
EXPNO                  4
PROCNO                 1
Date_           20110519
Time               11.00
INSTRUM           avc500
PROBHD   5 mm CPDUL 13C
PULPROG          zgpg30
TD                 65536
SOLVENT            CDCl3
NS                  1024
DS                     2
SWH            31250.000 Hz
FIDRES          0.476837 Hz
AQ             1.0486259 sec
RG                  1820
DW                16.000 usec
DE                 20.00 usec
TE                 298.0 K
D1            2.00000000 sec
D11           0.03000000 sec
TD0                    1

======== CHANNEL f1 ========
NUC1                 13C
P1                  9.10 usec
PL1                -4.40 dB
PL1W         28.15752029 W
SFO1         125.8131151 MHz

======== CHANNEL f2 ========
CPDPRG2          waltz16
NUC2                  1H
PCPD2              80.00 usec
PL2                -6.00 dB
PL12               12.42 dB
PL13               18.42 dB
PL2W         15.19999981 W
PL12W         0.21869738 W
PL13W         0.05493430 W
SFO2         500.3020012 MHz
SI                 32768
SF           125.8005438 MHz
WDW                   EM
SSB                    0
LB                  1.00 Hz
GB                     0
PC                  1.40
```

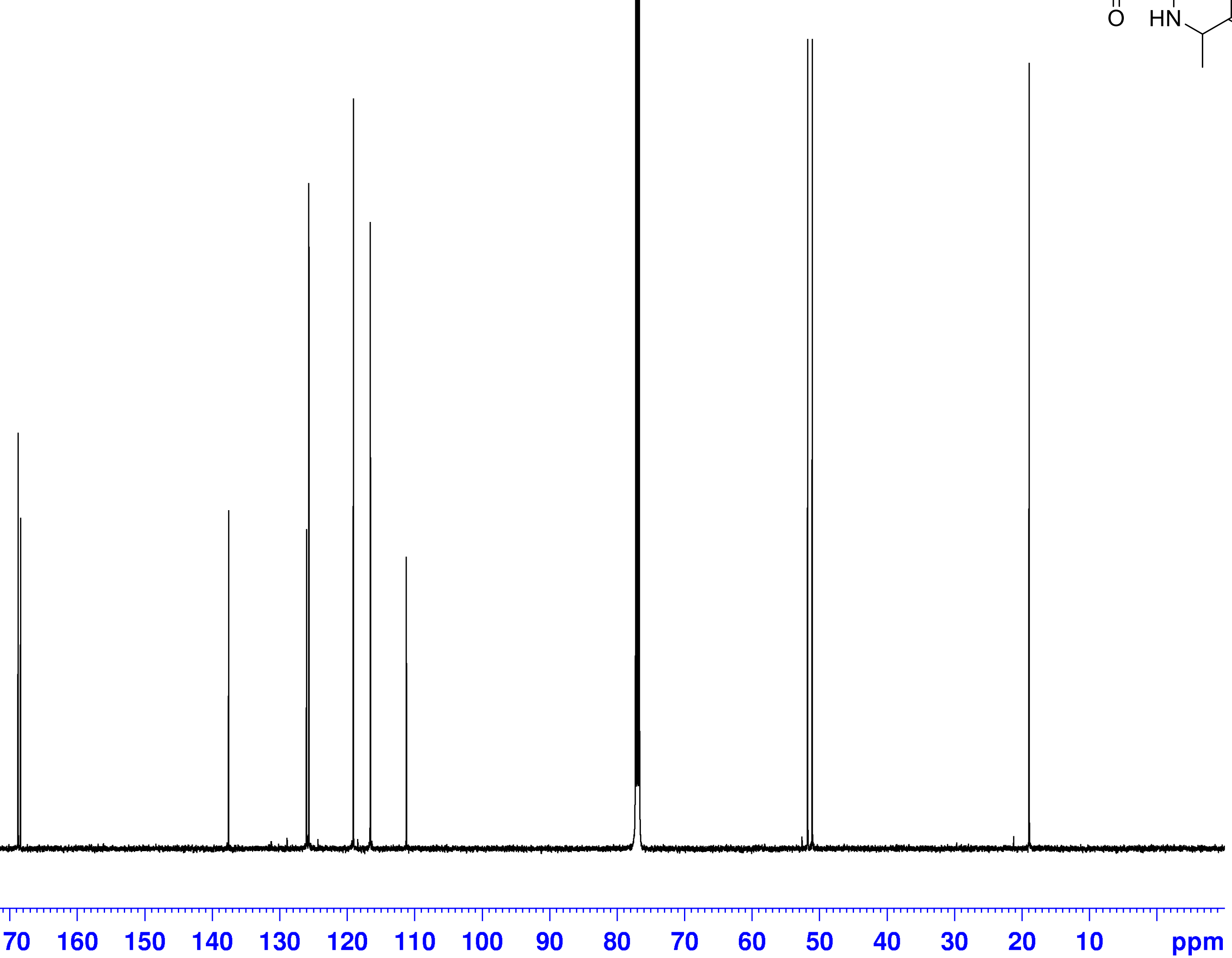

Methyl 3-(7-methoxy-3,4-dihydroquinolin-1(2*H*)-yl)propanoate **65** ^{1}H NMR

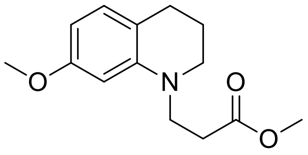


```
NAME             tr2070
EXPNO                 1
PROCNO                1
Date_          20121025
Time               5.19
INSTRUM           av400
PROBHD   5 mm QNP 1H/13
PULPROG            zg60
TD                65536
SOLVENT           CDCl3
NS                   16
DS                    2
SWH            8278.146 Hz
FIDRES         0.126314 Hz
AQ            3.9584243 sec
RG                 35.9
DW               60.400 usec
DE                 7.50 usec
TE                300.0 K
D1           1.00000000 sec

======== CHANNEL f1 ========
NUC1                 1H
P1                10.10 usec
PL1                0.00 dB
SFO1        400.2024714 MHz
SI                32768
SF          400.2000028 MHz
WDW                  EM
SSB                   0
LB                 0.30 Hz
GB                    0
PC                 1.00
```

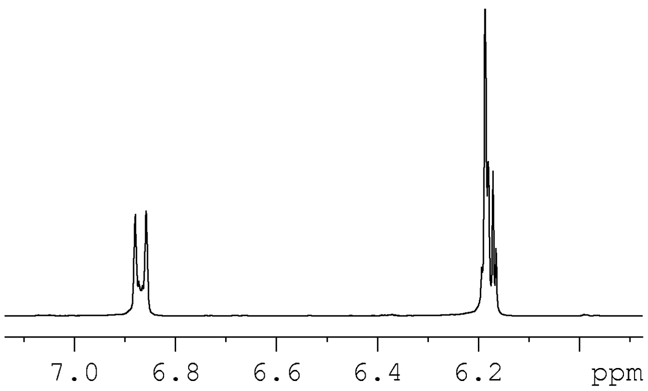

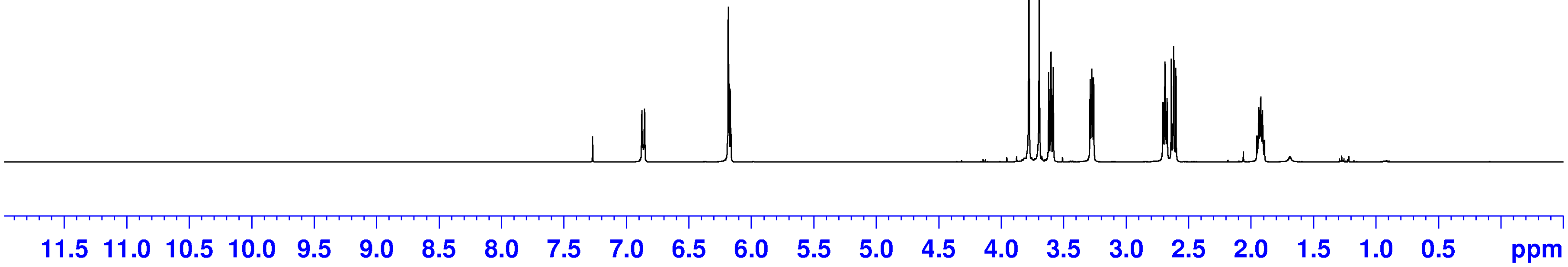

Methyl 3-(7-methoxy-3,4-dihydroquinolin-1(2*H*)-yl)propanoate **65** ^{13}C NMR

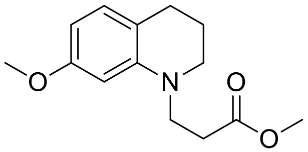


```
NAME               tr2070
EXPNO                   2
PROCNO                  1
Date_            20121025
Time                 5.27
INSTRUM             av400
PROBHD   5 mm QNP 1H/13
PULPROG           zgpg30
TD                  32768
SOLVENT             CDCl3
NS                    256
DS                      4
SWH            26178.010 Hz
FIDRES          0.798889 Hz
AQ             0.6259188 sec
RG                  32768
DW                 19.100 usec
DE                   7.50 usec
TE                  300.0 K
D1             1.00000000 sec
D11            0.03000000 sec
TD0                     1

======== CHANNEL f1 ========
NUC1                  13C
P1                   9.90 usec
PL1                  0.00 dB
SFO1          100.6403931 MHz

======== CHANNEL f2 ========
CPDPRG2           waltz16
NUC2                   1H
PCPD2               80.00 usec
PL2                  0.00 dB
PL12                19.00 dB
PL13                25.00 dB
SFO2          400.2016008 MHz
SI                  32768
SF            100.6303718 MHz
WDW                    EM
SSB                     0
LB                   1.00 Hz
GB                      0
PC                   1.40
```

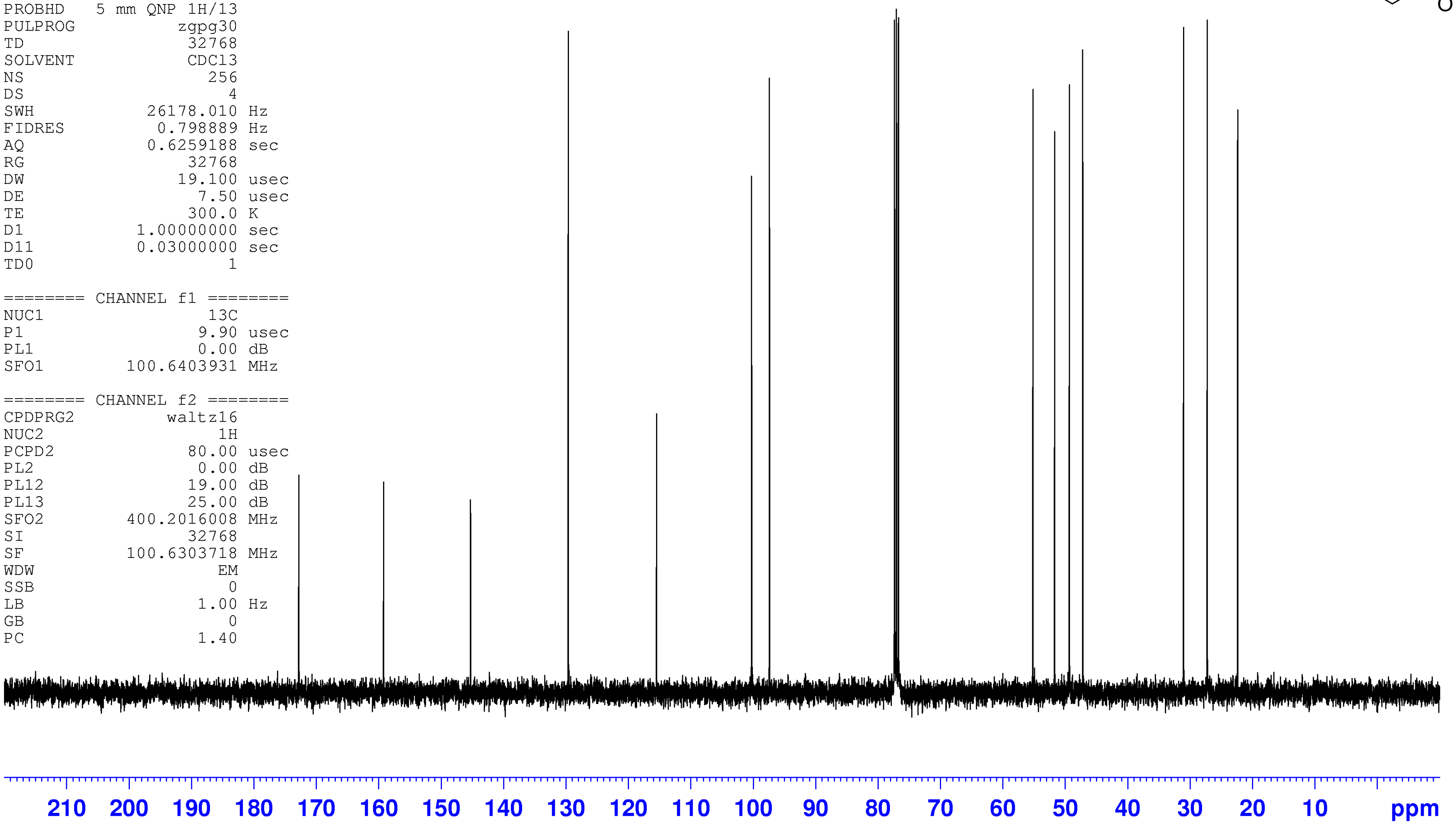

3-(7-methoxy-3,4-dihydroquinolin-1(2*H*)-yl)propan-1-ol **66** ^{1}H NMR

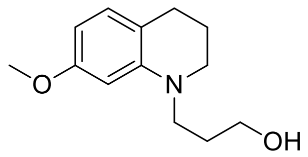

NAME	tr2075	
EXPNO	1	
PROCNO	1	
Date_	20120917	
Time	9.50	
INSTRUM	DPX300	
PROBHD	5 mm DUL 13C-1	
PULPROG	zg60	
TD	32768	
SOLVENT	CDCl3	
NS	16	
DS	2	
SWH	4789.272	Hz
FIDRES	0.146157	Hz
AQ	3.4210291	sec
RG	80.6	
DW	104.400	usec
DE	6.00	usec
TE	300.0	K
D1	1.00000000	sec
TD0	1	
======== CHANNEL f1 ========		
NUC1	1H	
P1	16.50	usec
PL1	-6.00	dB
SFO1	300.1315007	MHz
SI	32768	
SF	300.1300029	MHz
WDW	EM	
SSB	0	
LB	0.30	Hz
GB	0	
PC	1.00	

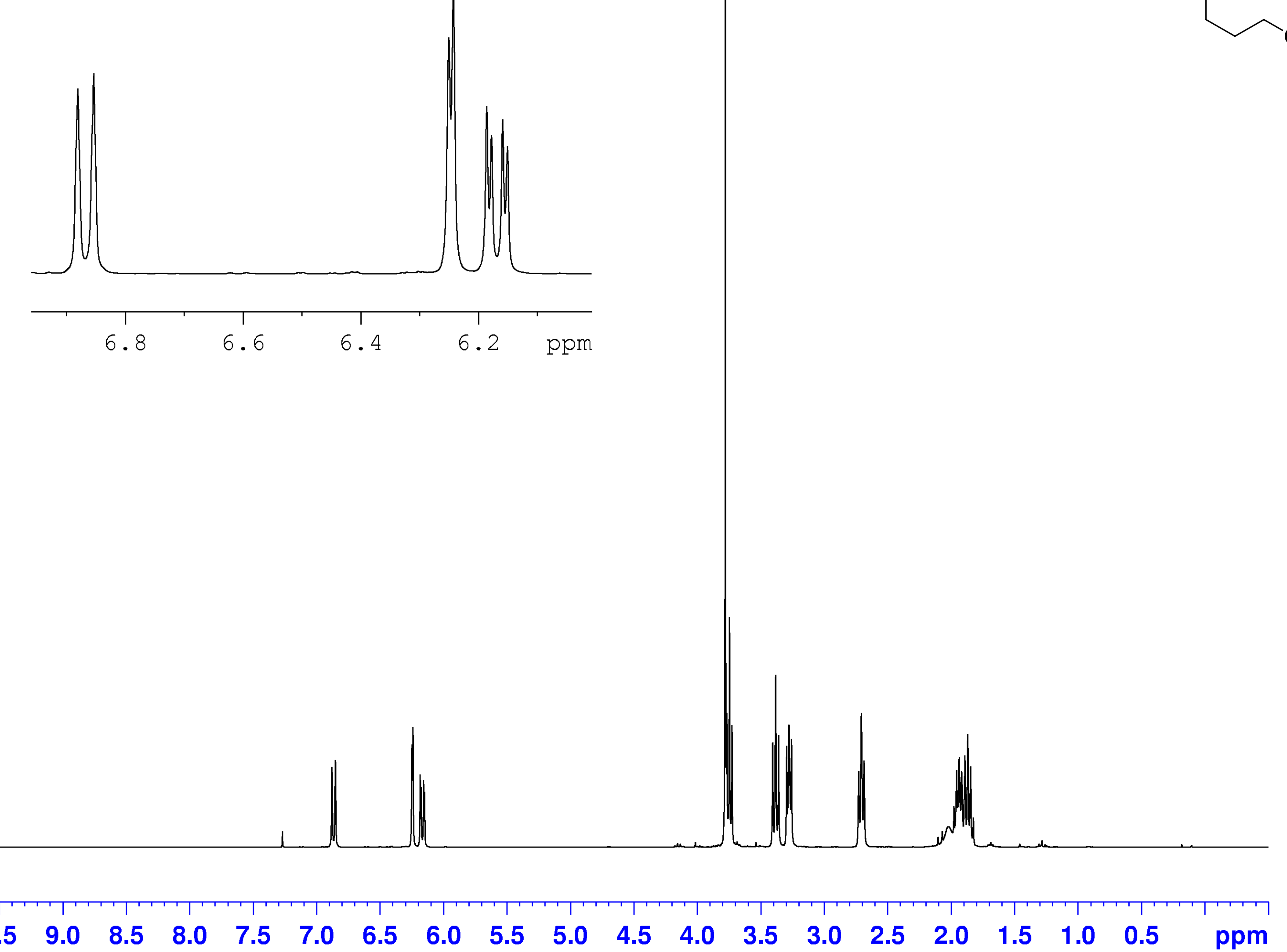

3-(7-methoxy-3,4-dihydroquinolin-1(2*H*)-yl)propan-1-ol **66** ^{13}C NMR

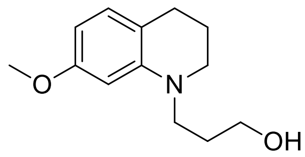

NAME	tr2075
EXPNO	2
PROCNO	1
Date_	20120917
Time	10.07
INSTRUM	DPX300
PROBHD	5 mm DUL 13C-1
PULPROG	zgpg30
TD	65536
SOLVENT	CDCl3
NS	256
DS	4
SWH	17985.611 Hz
FIDRES	0.274439 Hz
AQ	1.8219508 sec
RG	7298.2
DW	27.800 usec
DE	6.00 usec
TE	300.0 K
D1	2.00000000 sec
d11	0.03000000 sec
DELTA	1.89999998 sec
TD0	1

======== CHANNEL f1 ========

NUC1	13C
P1	8.25 usec
PL1	-2.40 dB
SFO1	75.4752953 MHz

======== CHANNEL f2 ========

CPDPRG2	waltz16
NUC2	1H
PCPD2	80.00 usec
PL2	-6.00 dB
PL12	11.00 dB
PL13	11.00 dB
SFO2	300.1312005 MHz
SI	32768
SF	75.4677490 MHz
WDW	EM
SSB	0
LB	1.00 Hz
GB	0
PC	1.40

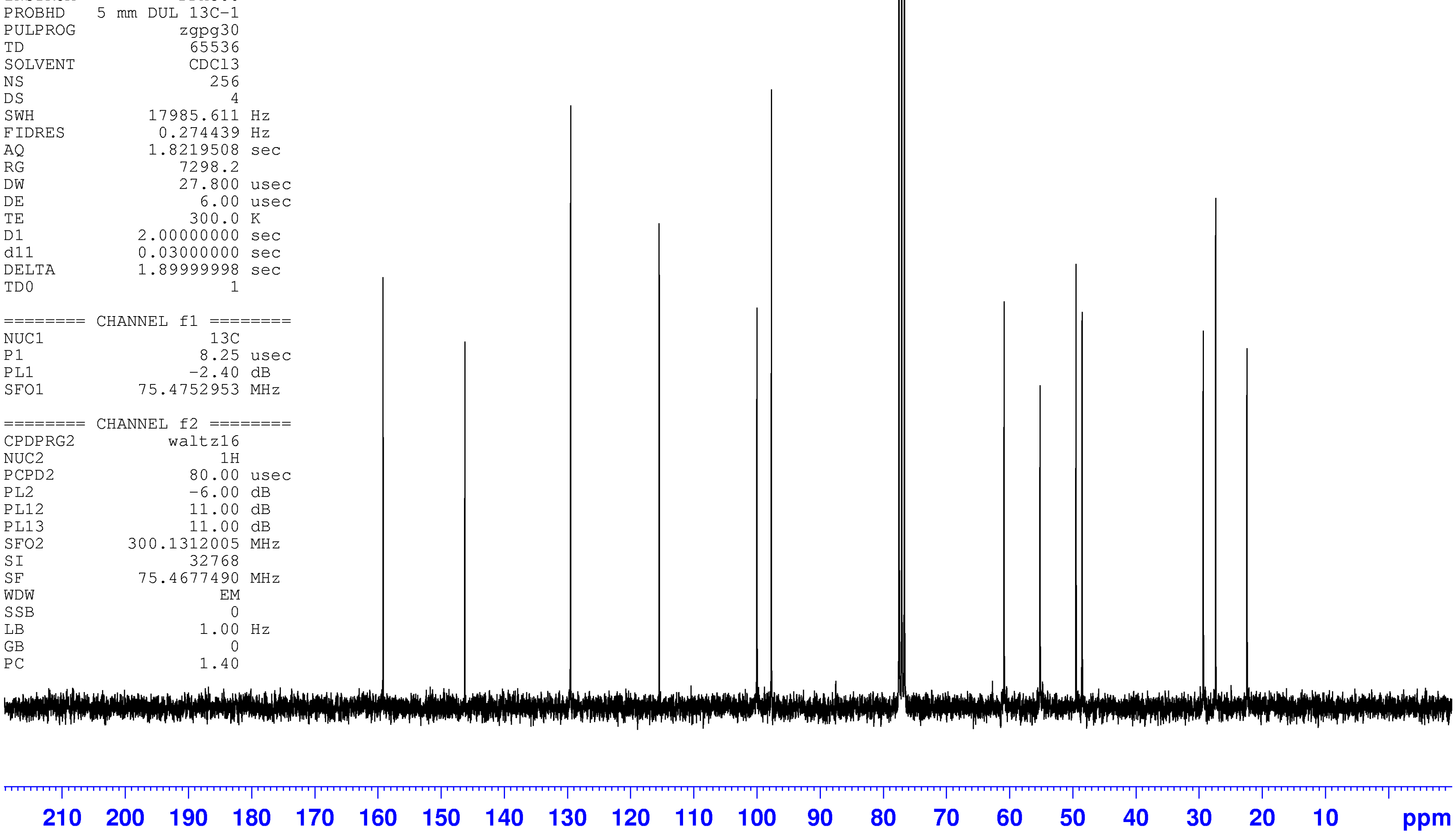

3-(3,4-Dihydroquinolin-1(2*H*)-yl)propan-1-ol **67** ^{1}H NMR

```
NAME              tr2040
EXPNO                  1
PROCNO                 1
Date_           20120105
Time                7.07
INSTRUM           avc500
PROBHD   5 mm CPDUL 13C
PULPROG             zg30
TD                 65536
SOLVENT            CDCl3
NS                    16
DS                     2
SWH            10330.578 Hz
FIDRES          0.157632 Hz
AQ             3.1719923 sec
RG                     4
DW                48.400 usec
DE                  6.00 usec
TE                 298.0 K
D1            1.00000000 sec
TD0                    1

======== CHANNEL f1 ========
NUC1                  1H
P1                  9.60 usec
PL1                -6.00 dB
PL1W         15.19999981 W
SFO1         500.3030896 MHz
SI                 32768
SF           500.3000240 MHz
WDW                   EM
SSB                    0
LB                  0.30 Hz
GB                     0
PC                  1.00
```

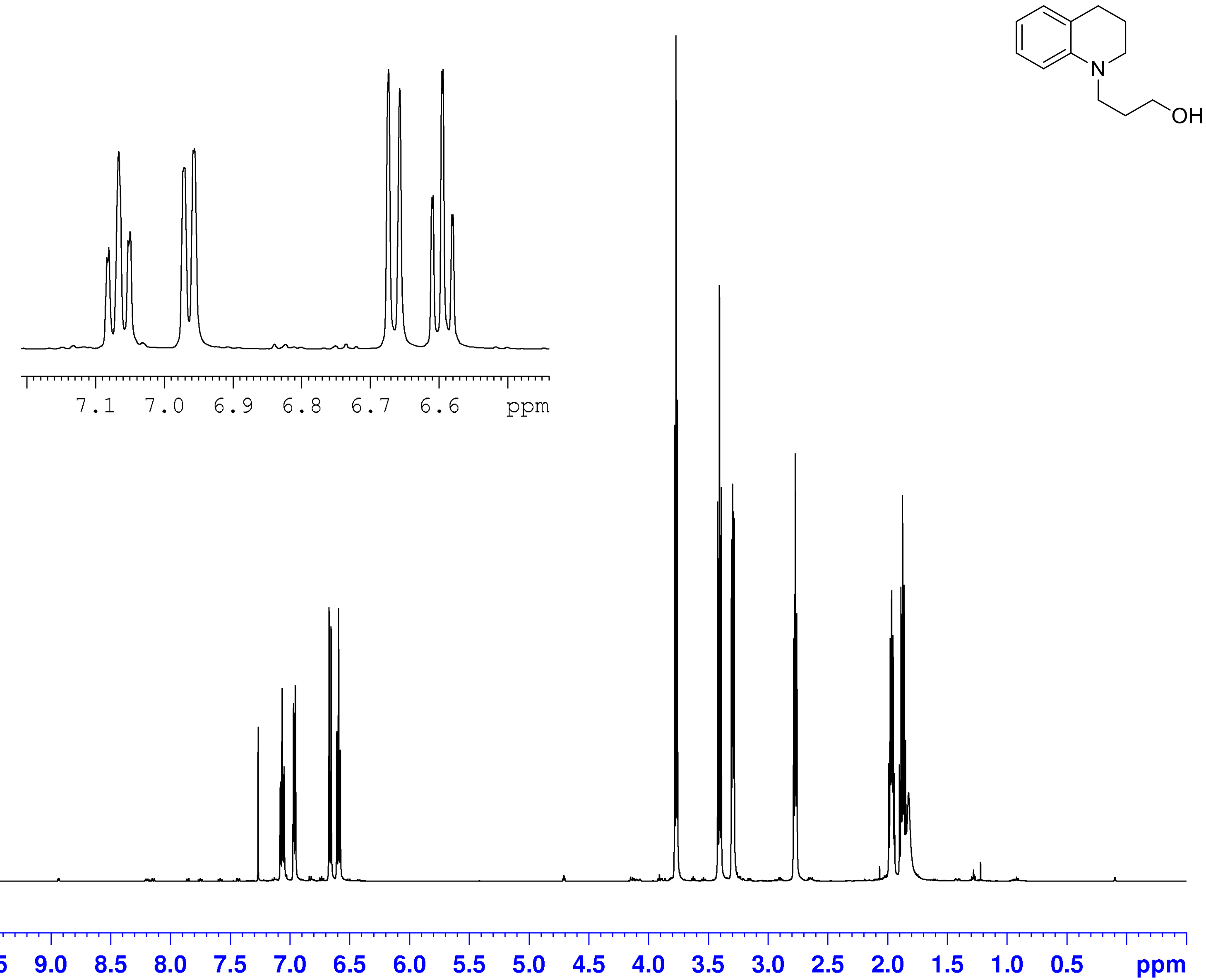

3-(3,4-Dihydroquinolin-1(2*H*)-yl)propan-1-ol **67** ^{13}C NMR

```
NAME               tr2040
EXPNO                   4
PROCNO                  1
Date_            20120105
Time                 7.55
INSTRUM            avc500
PROBHD   5 mm CPDUL 13C
PULPROG           zgpg30
TD                  65536
SOLVENT             CDCl3
NS                    512
DS                      2
SWH             31250.000 Hz
FIDRES           0.476837 Hz
AQ              1.0486259 sec
RG                   1820
DW                 16.000 usec
DE                  20.00 usec
TE                  298.0 K
D1             2.00000000 sec
D11            0.03000000 sec
TD0                     1

======== CHANNEL f1 ========
NUC1                  13C
P1                  10.00 usec
PL1                 -4.40 dB
PL1W          28.15752029 W
SFO1          125.8131151 MHz

======== CHANNEL f2 ========
CPDPRG2           waltz16
NUC2                   1H
PCPD2               80.00 usec
PL2                 -6.00 dB
PL12                12.42 dB
PL13                18.42 dB
PL2W          15.19999981 W
PL12W          0.21869738 W
PL13W          0.05493430 W
SFO2          500.3020012 MHz
SI                  32768
SF            125.8005438 MHz
WDW                    EM
SSB                     0
LB                   1.00 Hz
GB                      0
PC                   1.40
```

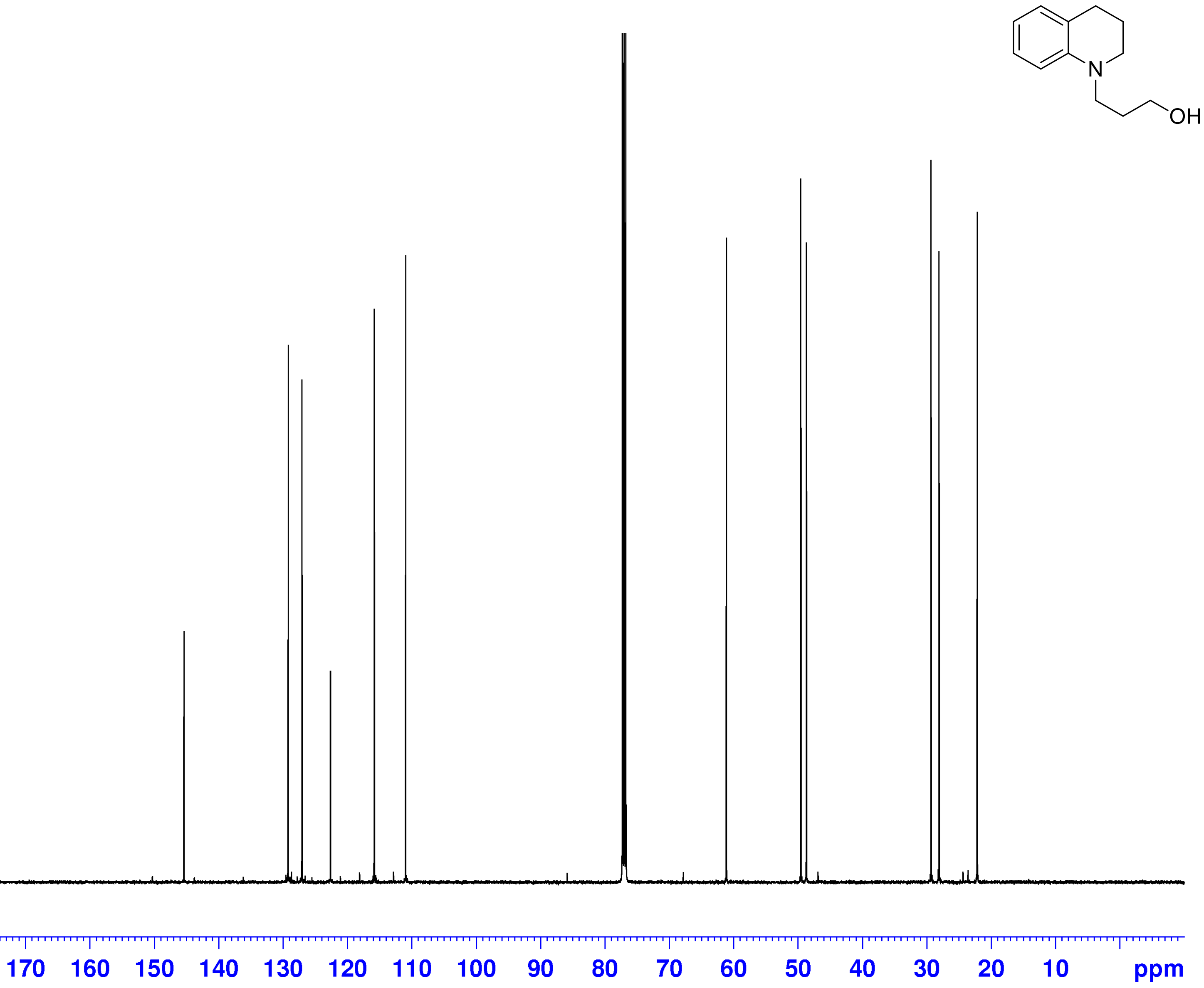

3-(6-Methoxy-3,4-dihydroquinolin-1(2*H*)-yl)propan-1-ol **68** ^{1}H NMR

```
NAME              tr2074
EXPNO                  1
PROCNO                 1
Date_           20131010
Time               13.01
INSTRUM           avf400
PROBHD    5 mm PABBO BB/
PULPROG             zg60
TD                 65536
SOLVENT            CDCl3
NS                    16
DS                     2
SWH             8012.820 Hz
FIDRES          0.122266 Hz
AQ             4.0894966 sec
RG                 20.19
DW                62.400 usec
DE                  6.50 usec
TE                 292.5 K
D1            1.00000000 sec
TD0                    1

======== CHANNEL f1 ========
SFO1         400.2524015 MHz
NUC1                  1H
P1                 12.65 usec
SI                 32768
SF           400.2500000 MHz
WDW                   EM
SSB                    0
LB                  0.30 Hz
GB                     0
PC                  1.00
```

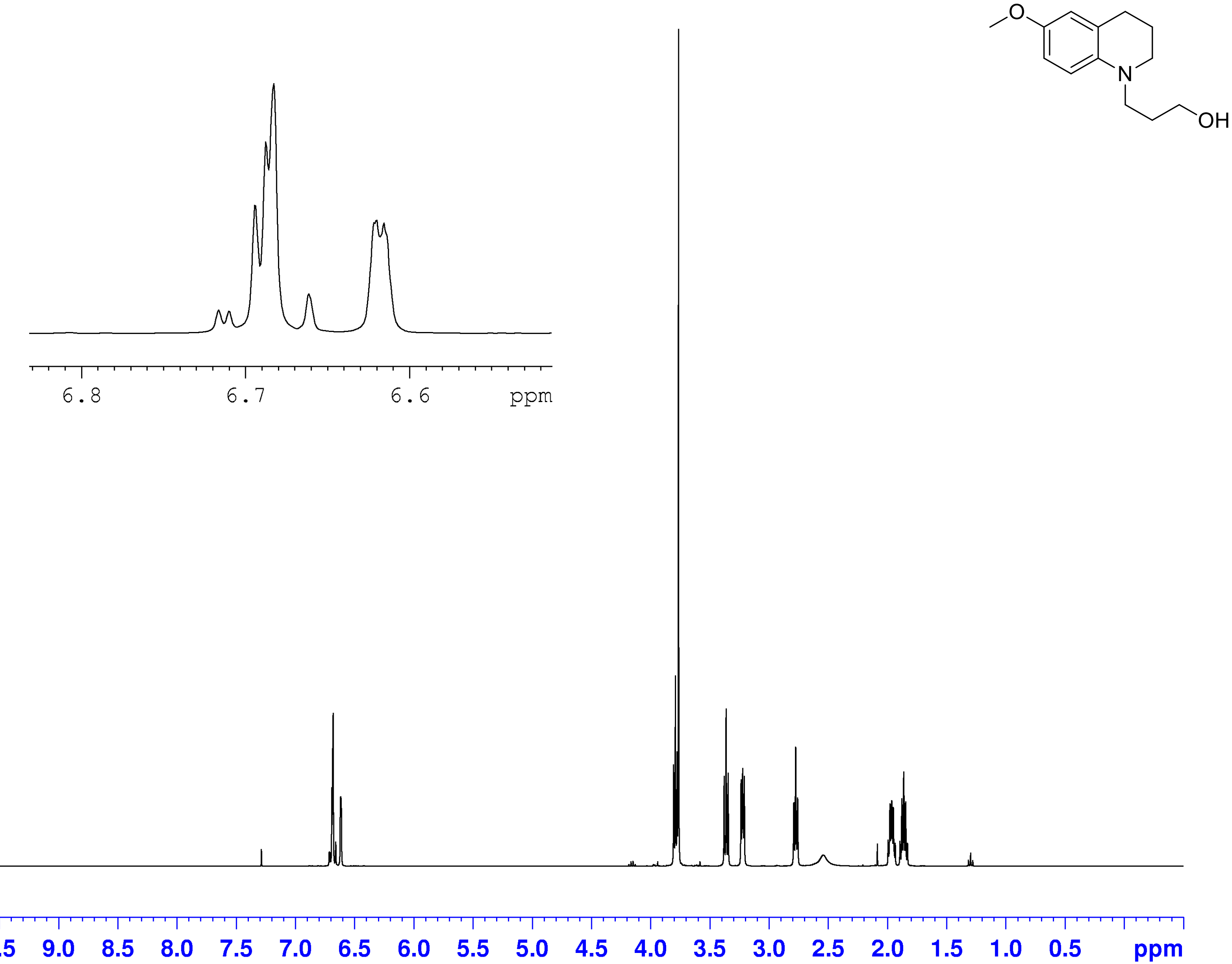

3-(6-Methoxy-3,4-dihydroquinolin-1(2*H*)-yl)propan-1-ol **68** ^{13}C NMR

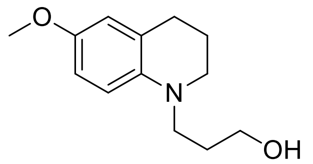


```
NAME               tr2074
EXPNO                   2
PROCNO                  1
Date_            20131010
Time                18.48
INSTRUM            avf400
PROBHD   5 mm PABBO BB/
PULPROG           zgpg30
TD                  32768
SOLVENT             CDCl3
NS                    256
DS                      4
SWH          26041.666 Hz
FIDRES        0.794729 Hz
AQ           0.6291956 sec
RG                 205.43
DW             19.200 usec
DE               6.50 usec
TE              293.8 K
D1         1.00000000 sec
D11        0.03000000 sec
TD0                     1

======== CHANNEL f1 ========
SFO1      100.6530073 MHz
NUC1                  13C
P1               9.00 usec
SI                  32768
SF        100.6429430 MHz
WDW                    EM
SSB                     0
LB               1.00 Hz
GB                      0
PC                   1.40
```

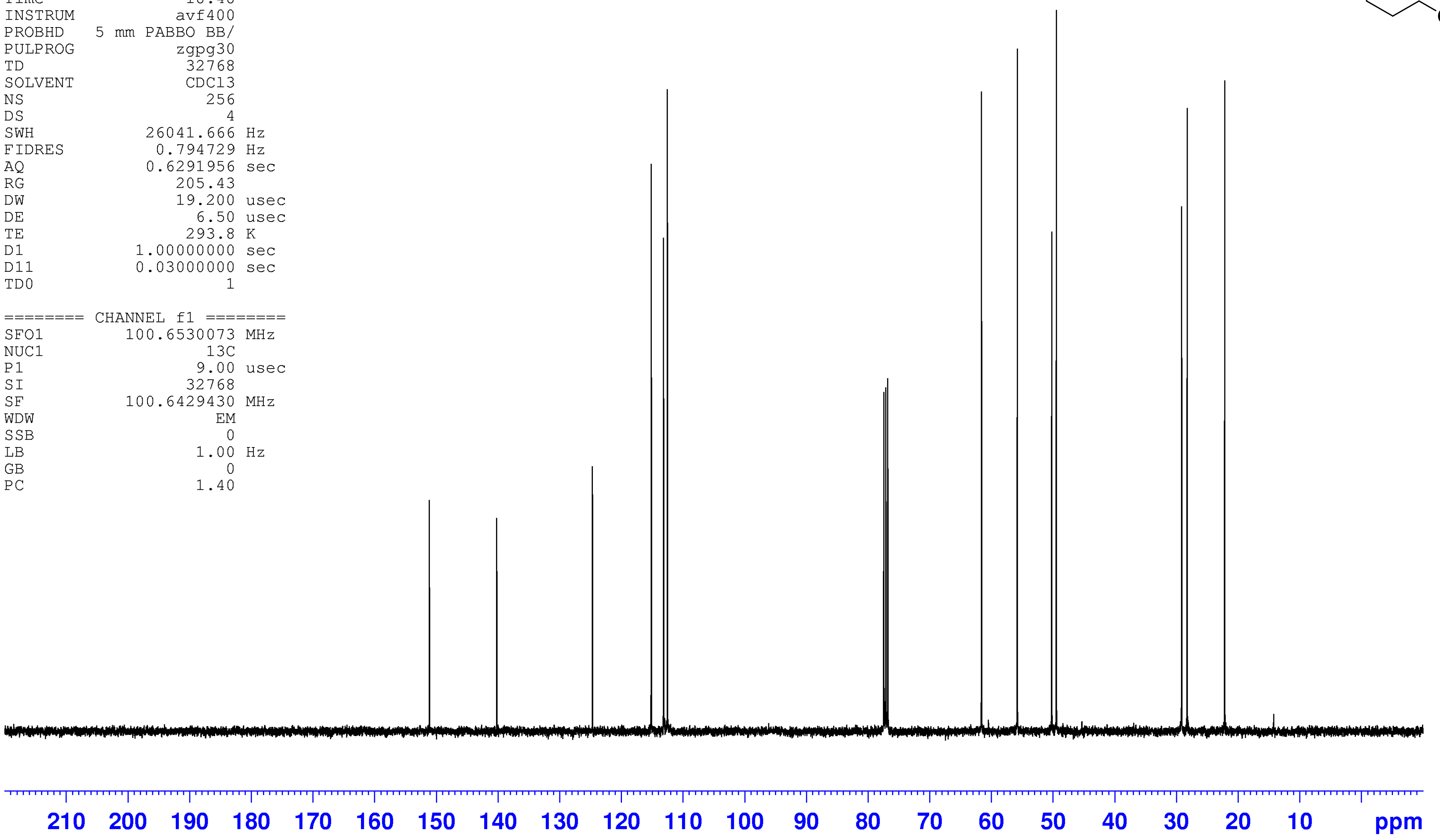

3-(2,3-Dihydro-4*H*-1,4-benzoxazin-4-yl)propan-1-ol **69** ^{1}H NMR

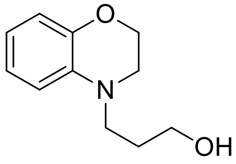

NAME	tr2076	
EXPNO	1	
PROCNO	1	
Date_	20131010	
Time	10.51	
INSTRUM	avg400	
PROBHD	5 mm QNP 1H/13	
PULPROG	zg30	
TD	65536	
SOLVENT	CDCl3	
NS	16	
DS	2	
SWH	10000.000	Hz
FIDRES	0.152588	Hz
AQ	3.2768500	sec
RG	166.74	
DW	50.000	usec
DE	6.50	usec
TE	294.5	K
D1	1.00000000	sec
TD0	1	

======== CHANNEL f1 ========

SFO1	400.2024714	MHz
NUC1	1H	
P1	12.23	usec
SI	65536	
SF	400.2000126	MHz
WDW	EM	
SSB	0	
LB	0.30	Hz
GB	0	
PC	1.00	

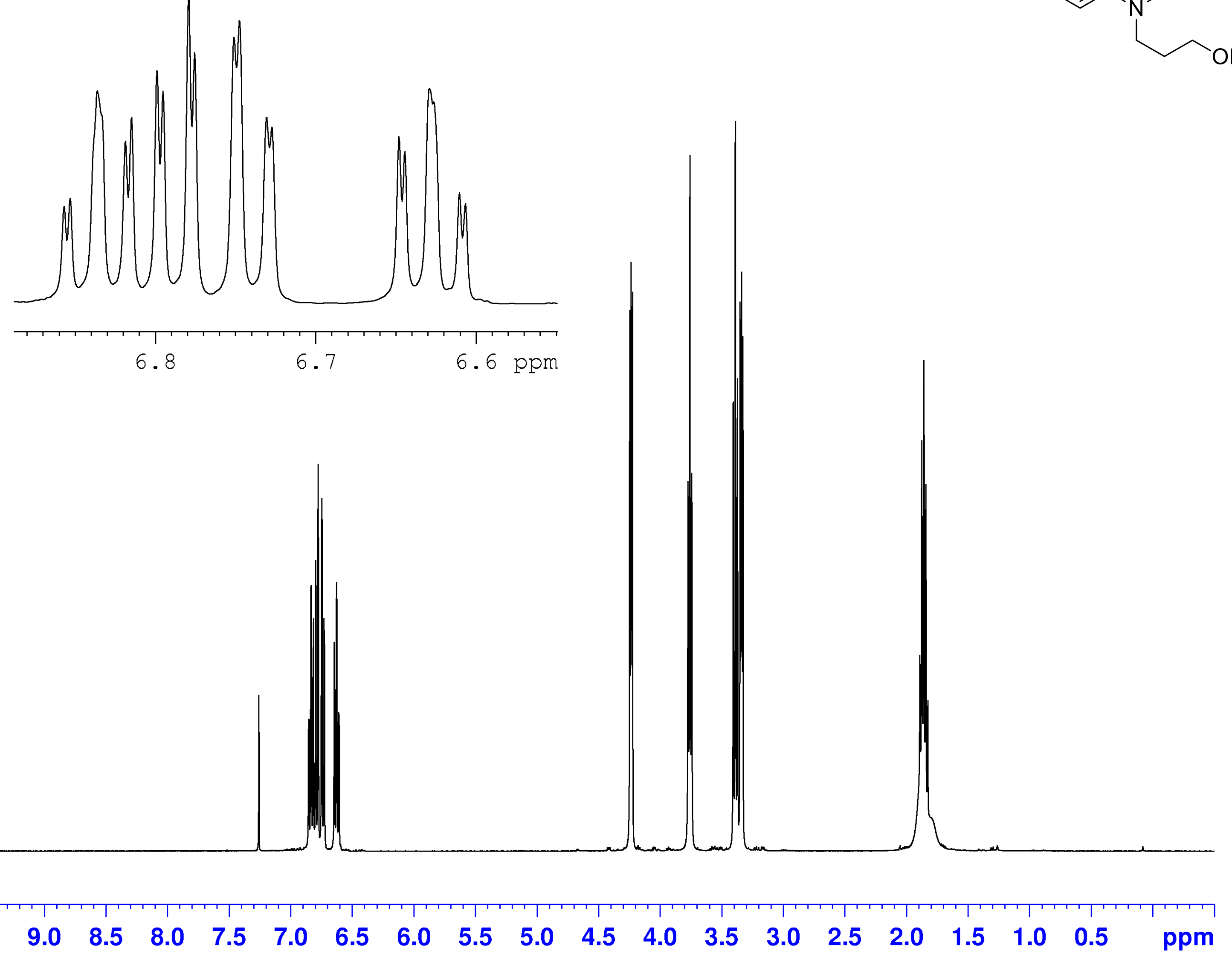

3-(2,3-Dihydro-4*H*-1,4-benzoxazin-4-yl)propan-1-ol **69** ^{13}C NMR

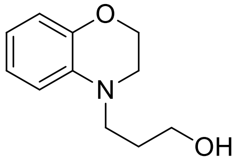


```
NAME              tr2076
EXPNO                  3
PROCNO                 1
Date_           20131011
Time                2.22
INSTRUM           avb400
PROBHD   5 mm PABBO BB/
PULPROG          zgpg30
TD                 65536
SOLVENT            CDCl3
NS                  1024
DS                     4
SWH            24038.461 Hz
FIDRES          0.366798 Hz
AQ             1.3631988 sec
RG                197.74
DW                20.800 usec
DE                  6.50 usec
TE                 298.0 K
D1            2.00000000 sec
D11           0.03000000 sec
TD0                    1

======== CHANNEL f1 ========
SFO1         100.6228303 MHz
NUC1                 13C
P1                 10.00 usec
SI                 32768
SF           100.6127690 MHz
WDW                   EM
SSB                    0
LB                  1.00 Hz
GB                     0
PC                  1.40
```

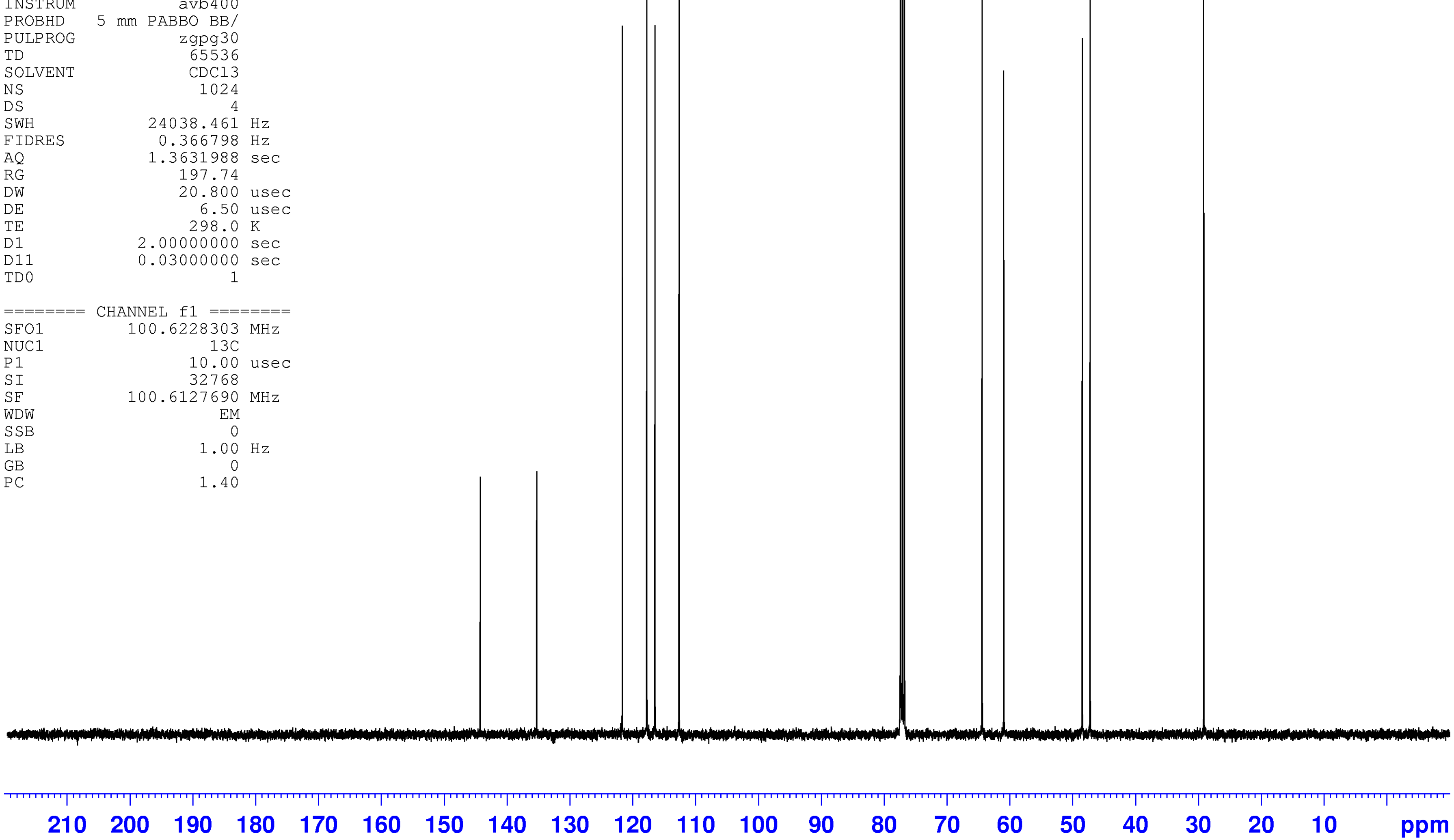

1-(3-azidopropyl)-7-methoxy-1,2,3,4-tetrahydroquinoline **70** ^{1}H NMR

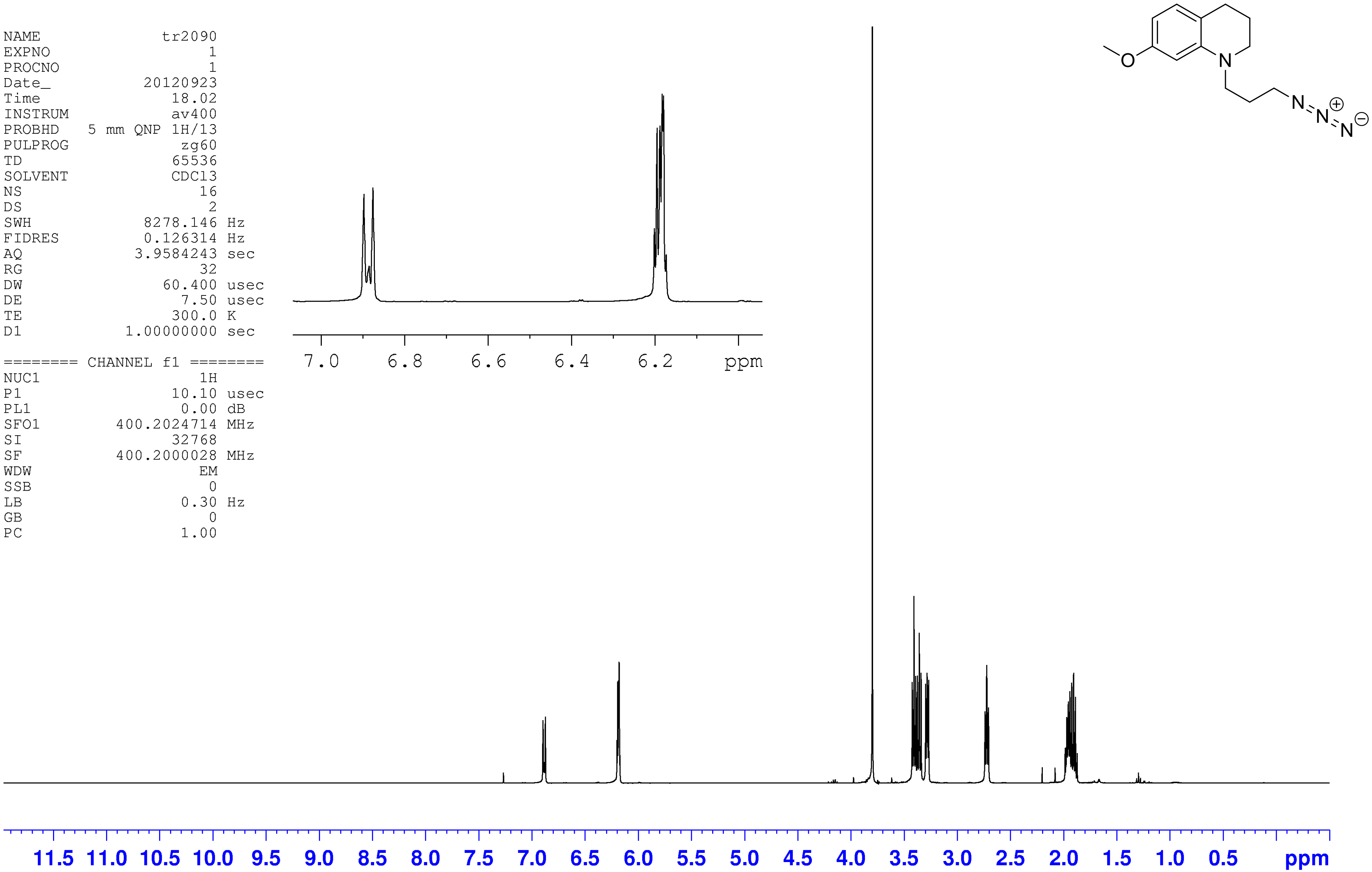

1-(3-azidopropyl)-7-methoxy-1,2,3,4-tetrahydroquinoline **70** ^{13}C NMR

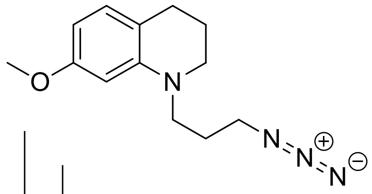


```
NAME               tr2090
EXPNO                   2
PROCNO                  1
Date_            20120923
Time                18.10
INSTRUM             av400
PROBHD   5 mm QNP 1H/13
PULPROG           zgpg30
TD                  32768
SOLVENT             CDCl3
NS                    256
DS                      4
SWH             26178.010 Hz
FIDRES           0.798889 Hz
AQ              0.6259188 sec
RG                  32768
DW                 19.100 usec
DE                   7.50 usec
TE                  300.0 K
D1             1.00000000 sec
D11            0.03000000 sec
TD0                     1

======== CHANNEL f1 ========
NUC1                  13C
P1                   9.90 usec
PL1                  0.00 dB
SFO1          100.6403931 MHz

======== CHANNEL f2 ========
CPDPRG2           waltz16
NUC2                   1H
PCPD2               80.00 usec
PL2                  0.00 dB
PL12                19.00 dB
PL13                25.00 dB
SFO2          400.2016008 MHz
SI                  32768
SF            100.6303718 MHz
WDW                    EM
SSB                     0
LB                   1.00 Hz
GB                      0
PC                   1.40
```

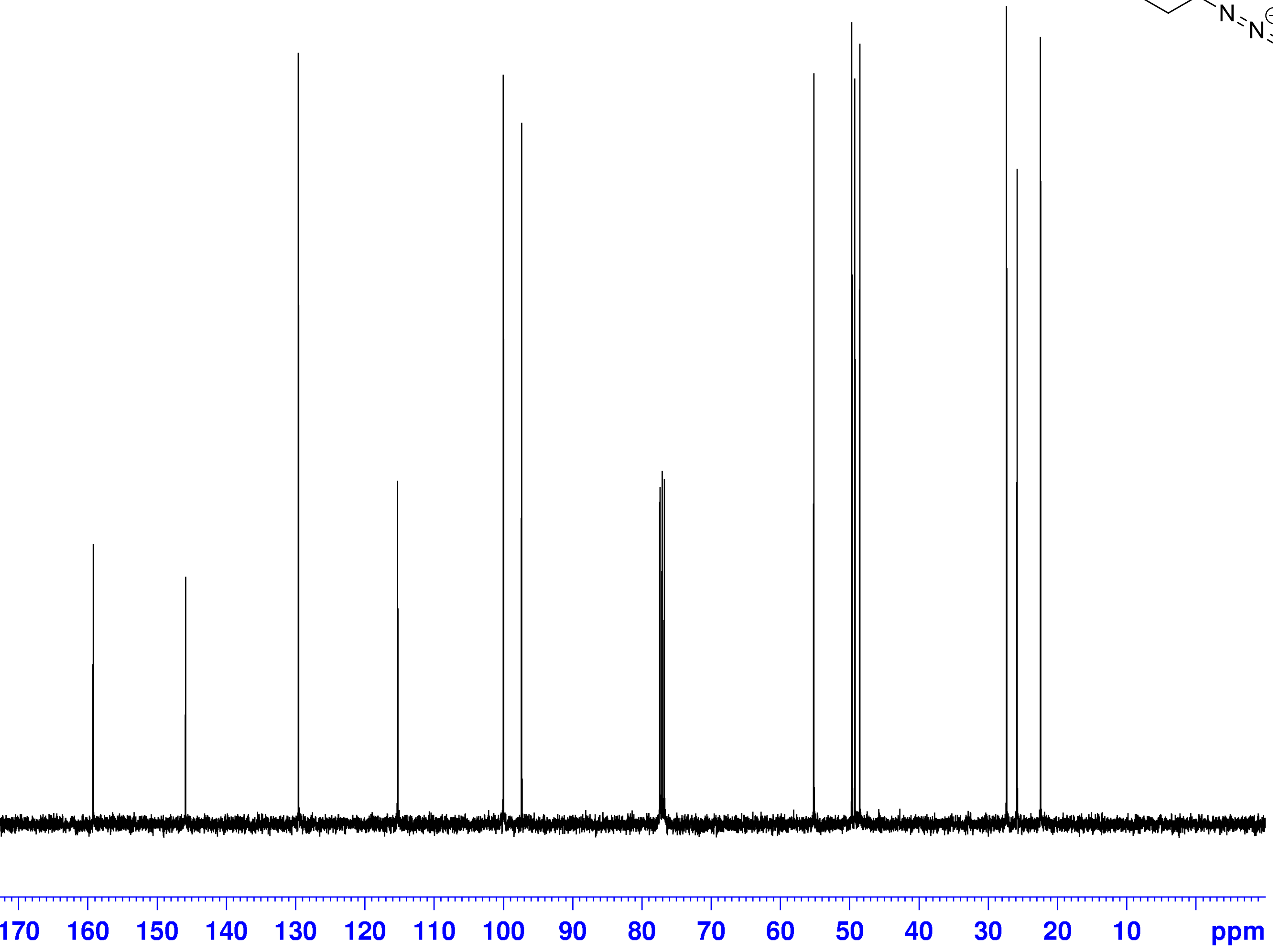

1-(3-Azidopropyl)-1,2,3,4-tetrahydroquinoline **71** ^{1}H NMR

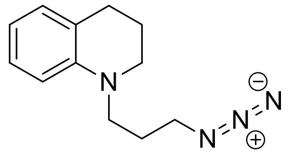

NAME	tr2058
EXPNO	1
PROCNO	1
Date_	20120406
Time	0.35
INSTRUM	av400
PROBHD	5 mm QNP 1H/13
PULPROG	zg60
TD	65536
SOLVENT	CDCl3
NS	16
DS	2
SWH	8278.146 Hz
FIDRES	0.126314 Hz
AQ	3.9584243 sec
RG	35.9
DW	60.400 usec
DE	7.50 usec
TE	300.0 K
D1	1.00000000 sec

======== CHANNEL f1 ========

NUC1	1H
P1	9.00 usec
PL1	0.00 dB
SFO1	400.2024714 MHz
SI	32768
SF	400.2000028 MHz
WDW	EM
SSB	0
LB	0.30 Hz
GB	0
PC	1.00

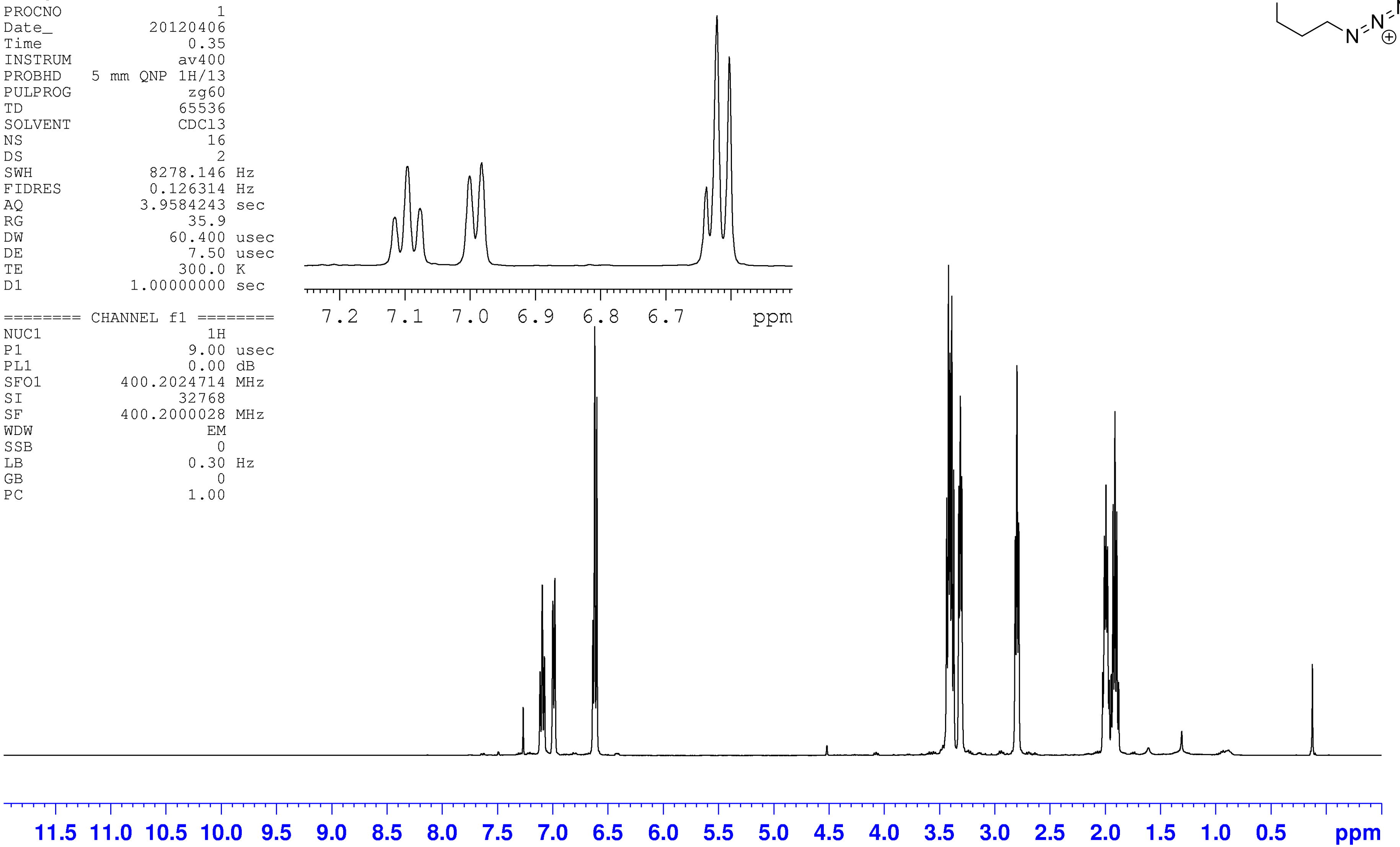

1-(3-Azidopropyl)-1,2,3,4-tetrahydroquinoline **71** ^{13}C NMR

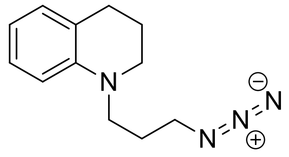


```
NAME              tr2058
EXPNO                  2
PROCNO                 1
Date_           20120406
Time                0.42
INSTRUM            av400
PROBHD   5 mm QNP 1H/13
PULPROG          zgpg30
TD                 32768
SOLVENT            CDCl3
NS                   256
DS                     4
SWH            26178.010 Hz
FIDRES          0.798889 Hz
AQ             0.6259188 sec
RG                 32768
DW                19.100 usec
DE                  7.50 usec
TE                 300.0 K
D1            1.00000000 sec
D11           0.03000000 sec
TD0                    1

======== CHANNEL f1 ========
NUC1                 13C
P1                  9.50 usec
PL1                 0.00 dB
SFO1         100.6403931 MHz

======== CHANNEL f2 ========
CPDPRG2          waltz16
NUC2                  1H
PCPD2              80.00 usec
PL2                 0.00 dB
PL12               19.00 dB
PL13               25.00 dB
SFO2         400.2016008 MHz
SI                 32768
SF           100.6303718 MHz
WDW                   EM
SSB                    0
LB                  1.00 Hz
GB                     0
PC                  1.40
```

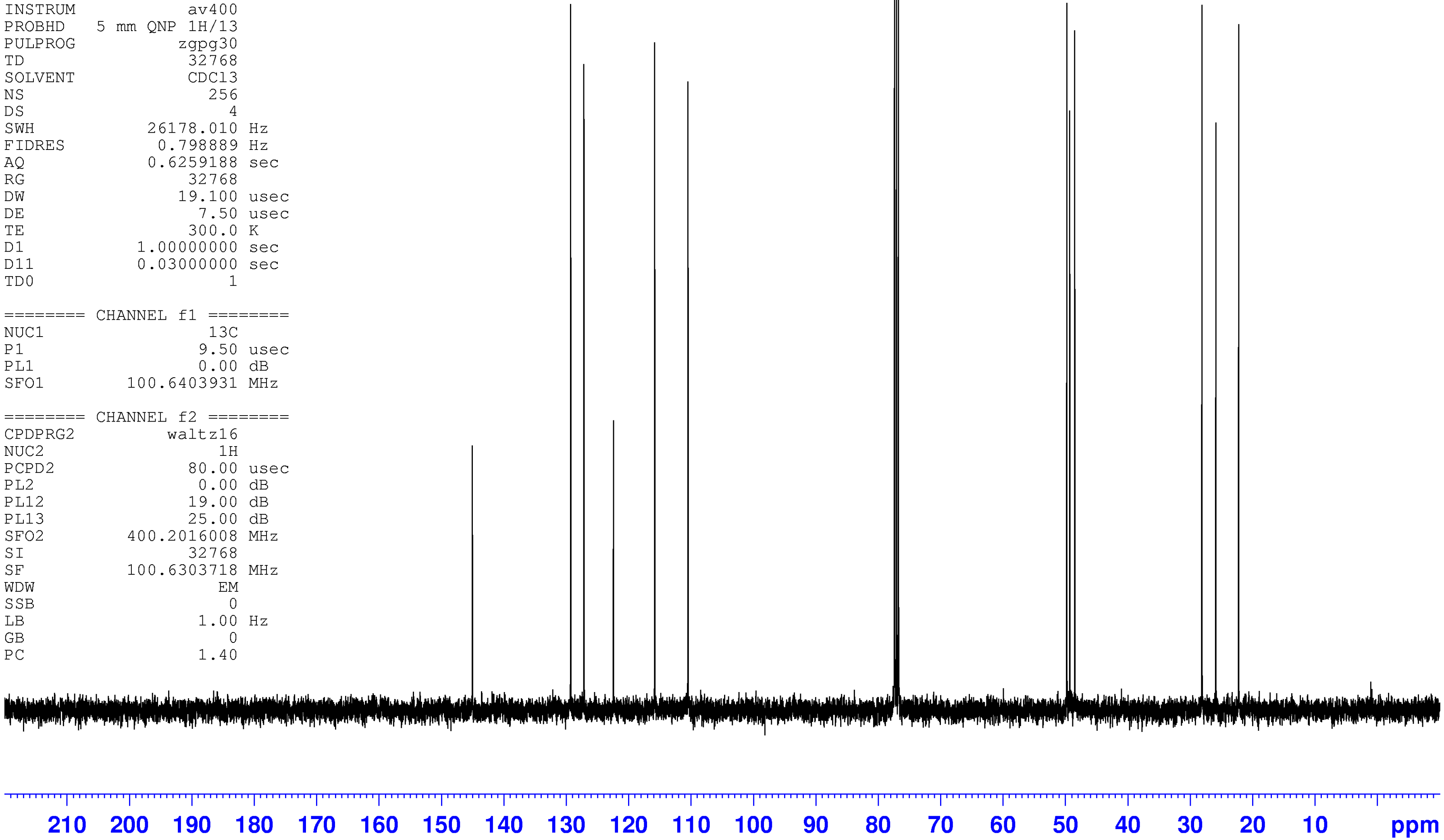

1-(3-Azidopropyl)-6-methoxy-1,2,3,4-tetrahydroquinoline **72** ^{1}H NMR

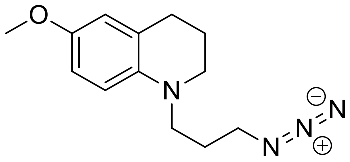

```
NAME              tr2089
EXPNO                  1
PROCNO                 1
Date_           20131010
Time               12.12
INSTRUM           avg400
PROBHD   5 mm QNP 1H/13
PULPROG             zg30
TD                 65536
SOLVENT            CDCl3
NS                    16
DS                     2
SWH            10000.000 Hz
FIDRES          0.152588 Hz
AQ             3.2768500 sec
RG                279.35
DW                50.000 usec
DE                  6.50 usec
TE                 294.5 K
D1            1.00000000 sec
TD0                    1

======== CHANNEL f1 ========
SFO1         400.2024714 MHz
NUC1                  1H
P1                 12.23 usec
SI                 65536
SF           400.2000127 MHz
WDW                   EM
SSB                    0
LB                  0.30 Hz
GB                     0
PC                  1.00
```

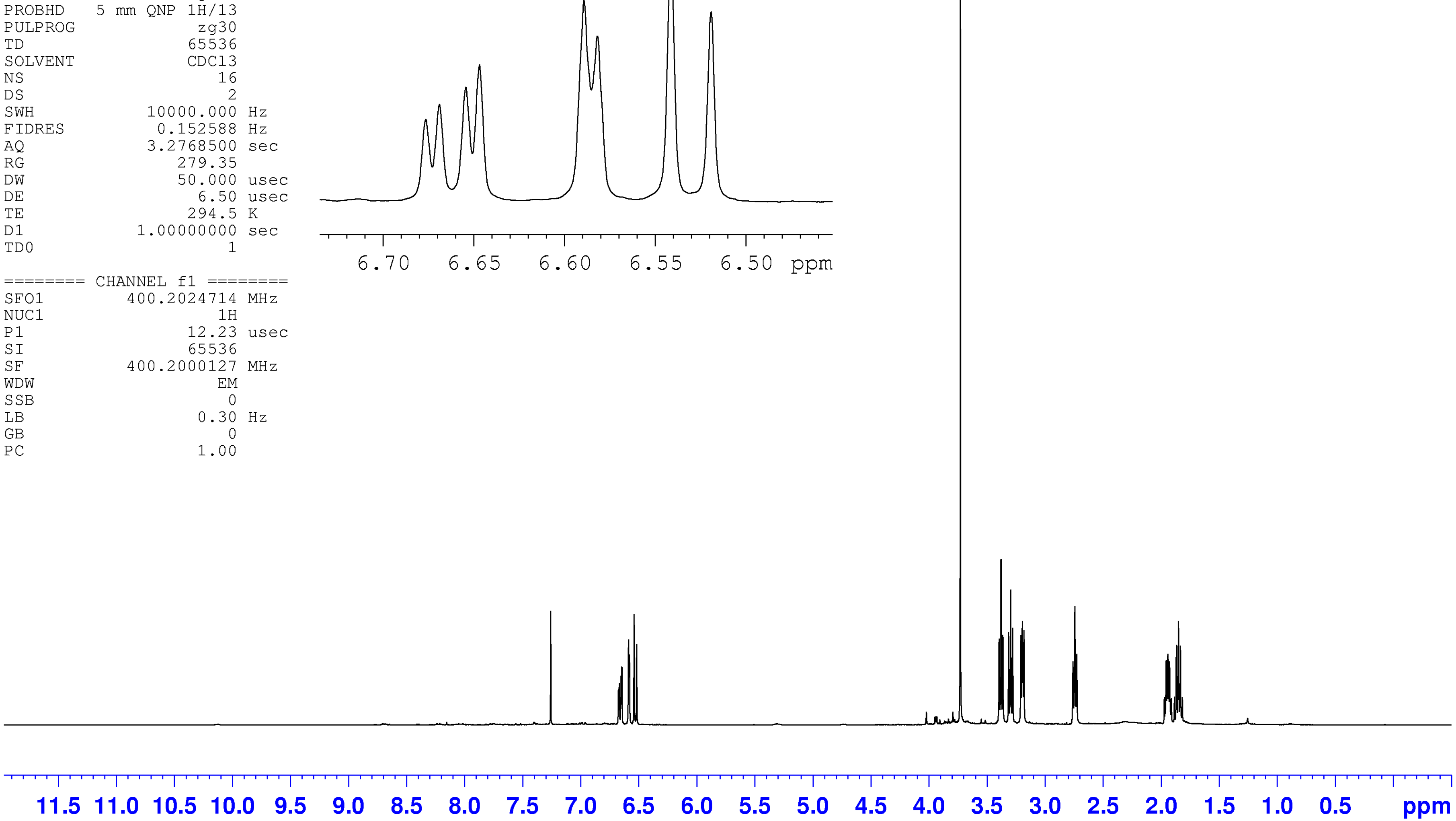

1-(3-Azidopropyl)-6-methoxy-1,2,3,4-tetrahydroquinoline **72** ^{13}C NMR

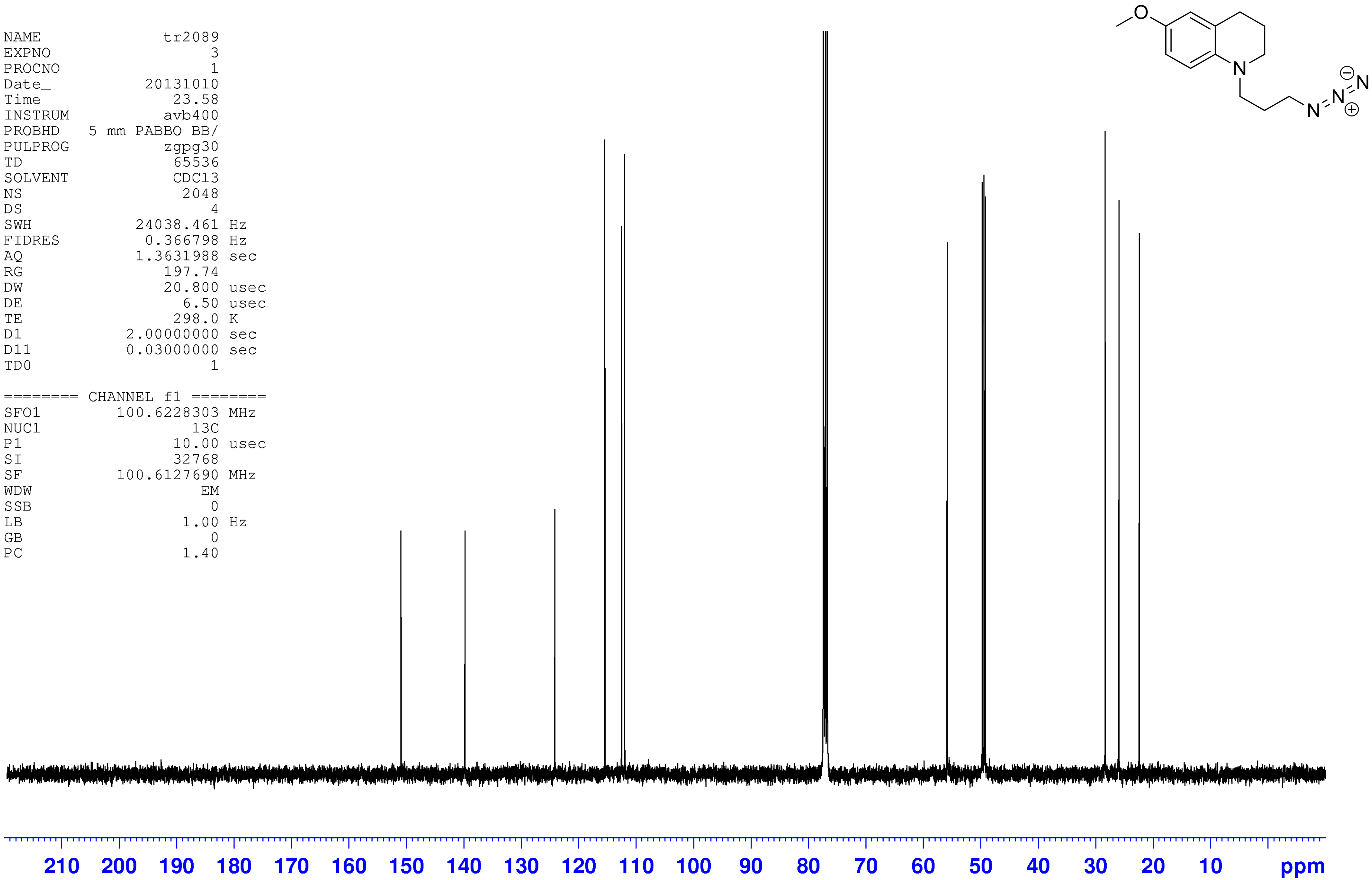

4-(3-Azidopropyl)-3,4-dihydro-2*H*-1,4-benzoxazine **73** ^{1}H NMR

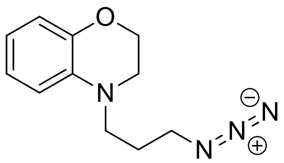

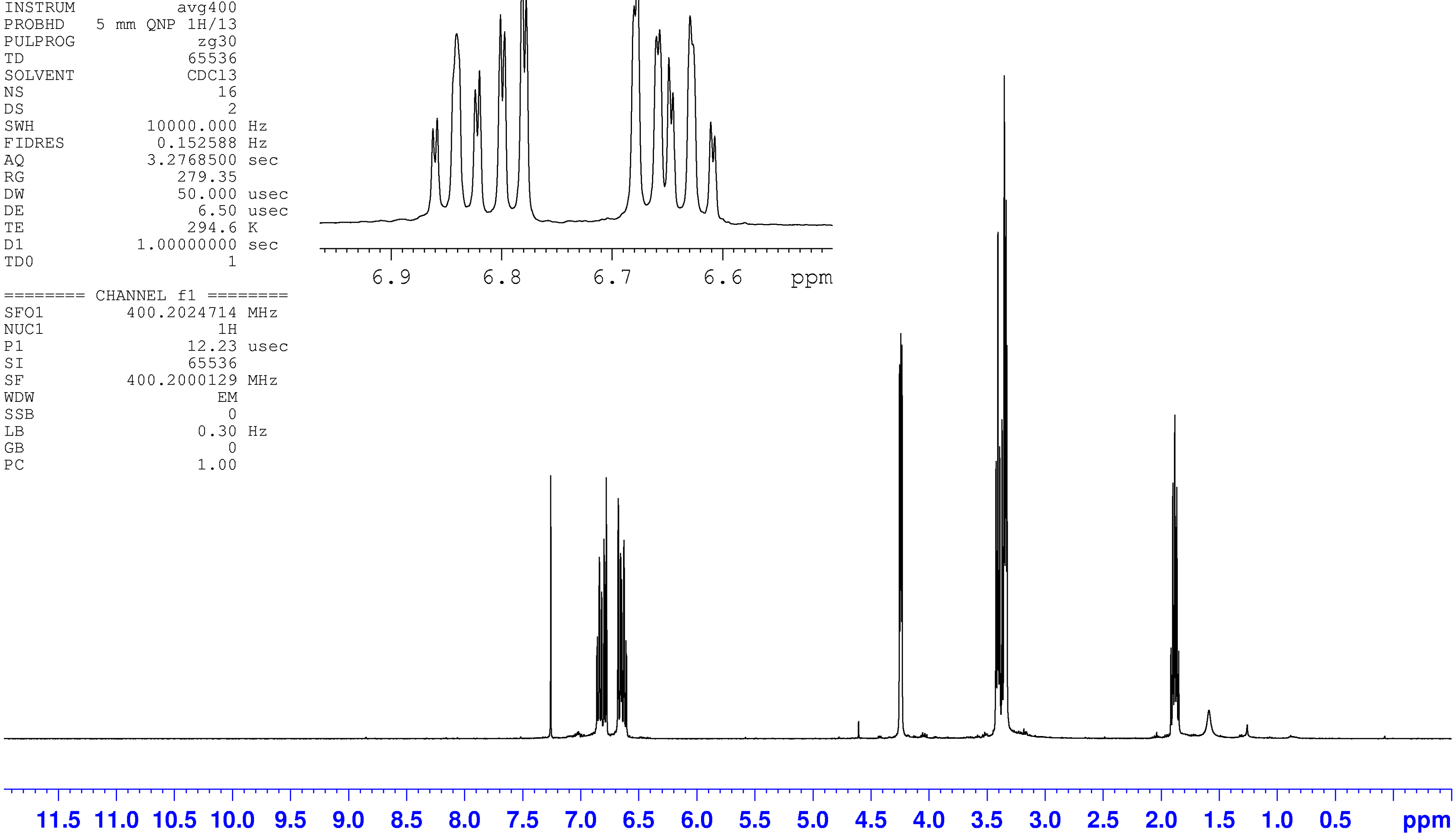

4-(3-Azidopropyl)-3,4-dihydro-2*H*-1,4-benzoxazine **73** ^{13}C NMR

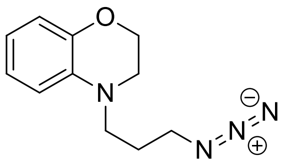


```
NAME              tr2091
EXPNO                  3
PROCNO                 1
Date_           20131010
Time               20.36
INSTRUM           avb400
PROBHD   5 mm PABBO BB/
PULPROG          zgpg30
TD                 65536
SOLVENT            CDCl3
NS                  2048
DS                     4
SWH            24038.461 Hz
FIDRES          0.366798 Hz
AQ             1.3631988 sec
RG                197.74
DW                20.800 usec
DE                  6.50 usec
TE                 298.0 K
D1            2.00000000 sec
D11           0.03000000 sec
TD0                    1

======== CHANNEL f1 ========
SFO1         100.6228303 MHz
NUC1                 13C
P1                 10.00 usec
SI                 32768
SF           100.6127554 MHz
WDW                   EM
SSB                    0
LB                  1.00 Hz
GB                     0
PC                  1.40
```

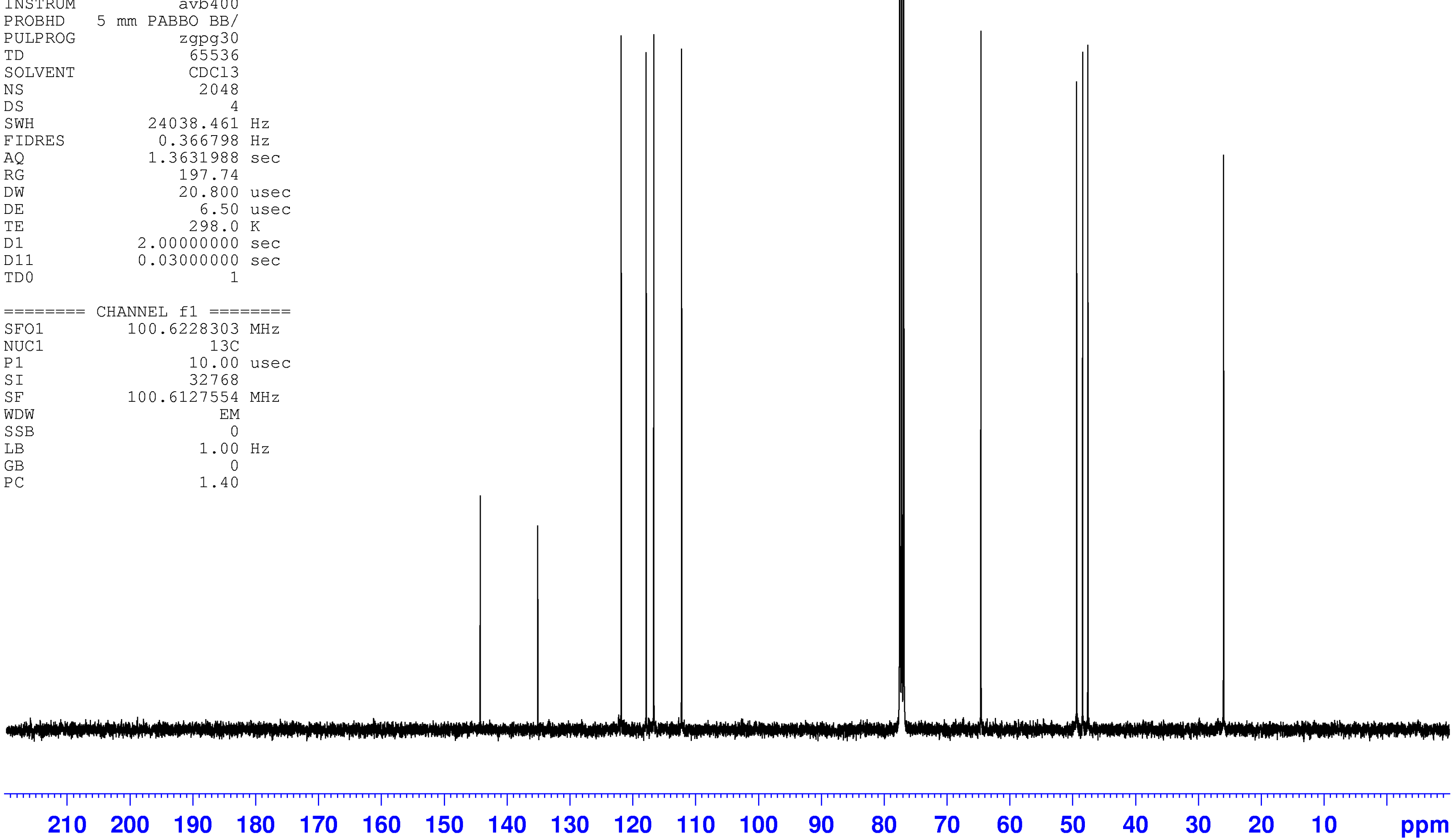

3-(7-methoxy-3,4-dihydroquinolin-1(2*H*)-yl)propan-1-amine **74** ^{1}H NMR

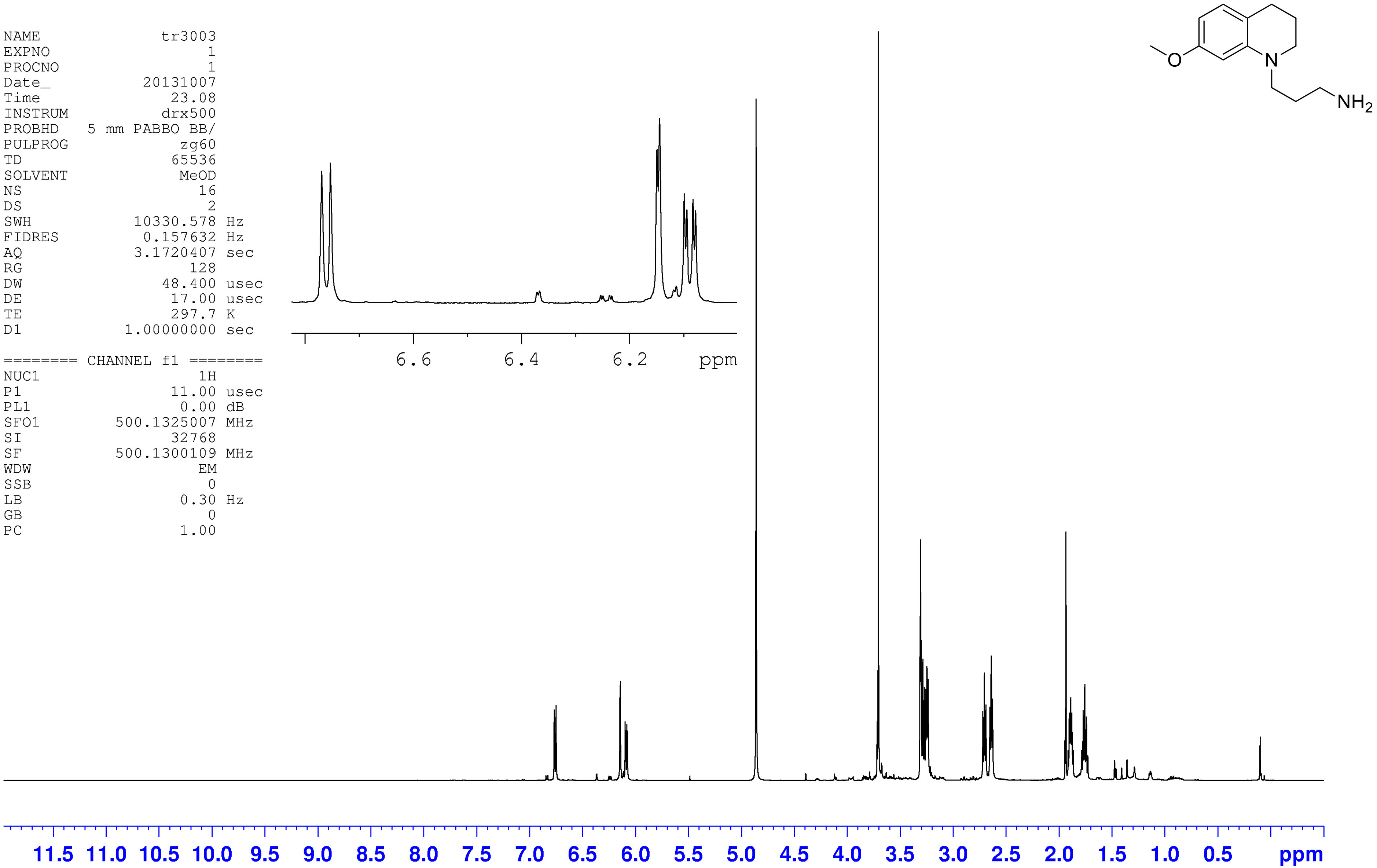

3-(7-methoxy-3,4-dihydroquinolin-1(2*H*)-yl)propan-1-amine **74** ^{13}C NMR

```
NAME              tr3003
EXPNO                  2
PROCNO                 1
Date_           20131008
Time                0.58
INSTRUM           drx500
PROBHD   5 mm PABBO BB/
PULPROG          zgpg30
TD                 65536
SOLVENT             MeOD
NS                  2048
DS                     4
SWH            30303.031 Hz
FIDRES          0.462388 Hz
AQ             1.0814105 sec
RG               11585.2
DW                16.500 usec
DE                 11.00 usec
TE                 297.7 K
D1            2.00000000 sec
d11           0.03000000 sec
DELTA         1.89999998 sec
TD0                    1

======== CHANNEL f1 ========
NUC1                 13C
P1                  6.80 usec
PL1                 5.00 dB
SFO1         125.7703148 MHz

======== CHANNEL f2 ========
CPDPRG2          waltz16
NUC2                  1H
PCPD2             100.00 usec
PL2                 0.00 dB
PL12               19.00 dB
PL13               23.00 dB
SFO2         500.1320005 MHz
SI                 32768
SF           125.7575604 MHz
WDW                   EM
SSB                    0
LB                  1.00 Hz
GB                     0
PC                  1.40
```

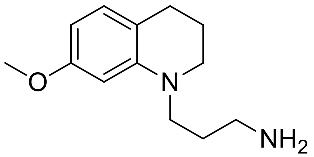

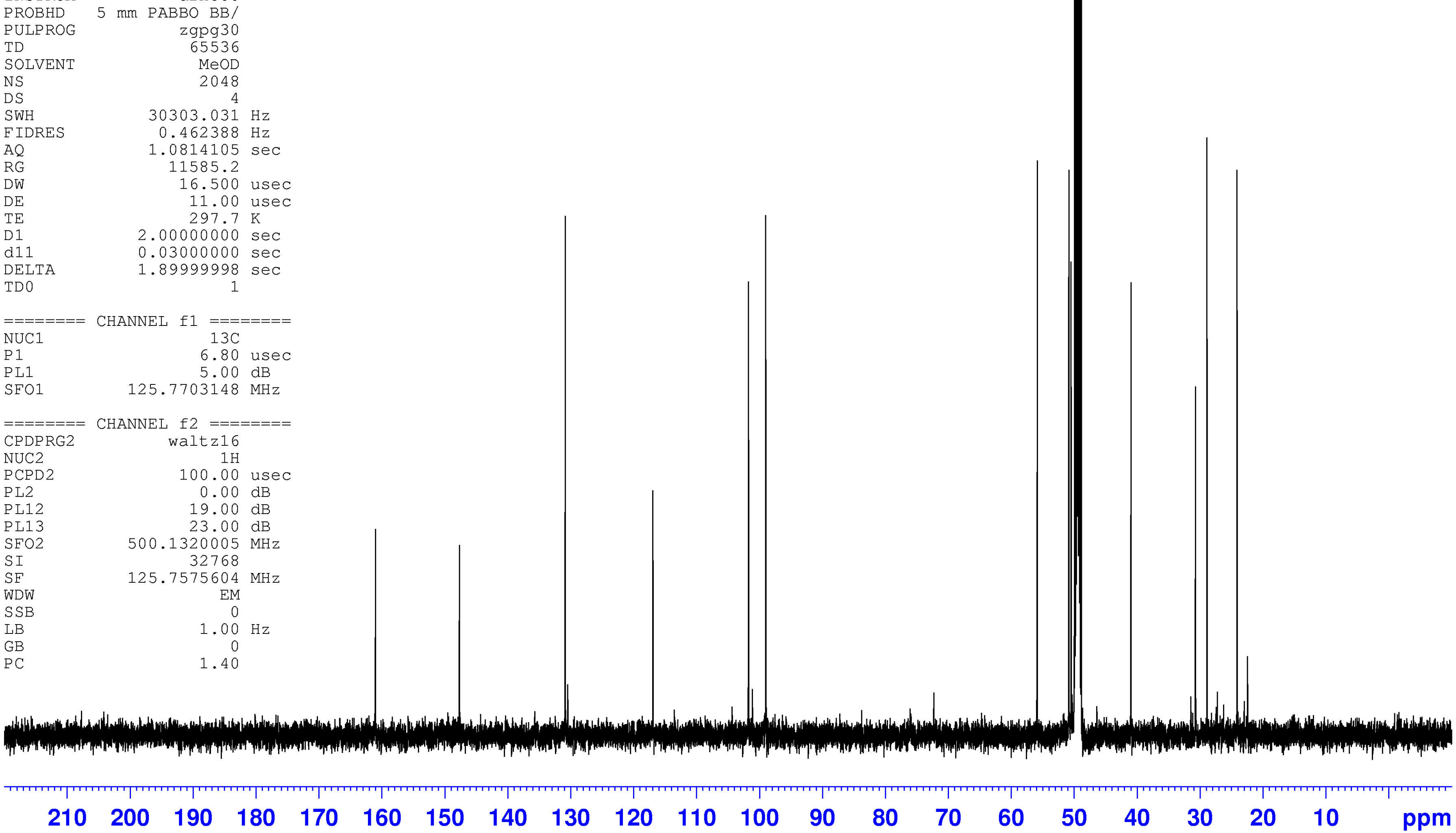

3-(3,4-Dihydroquinolin-1(2*H*)-yl)propan-1-amine **75** ^{1}H NMR

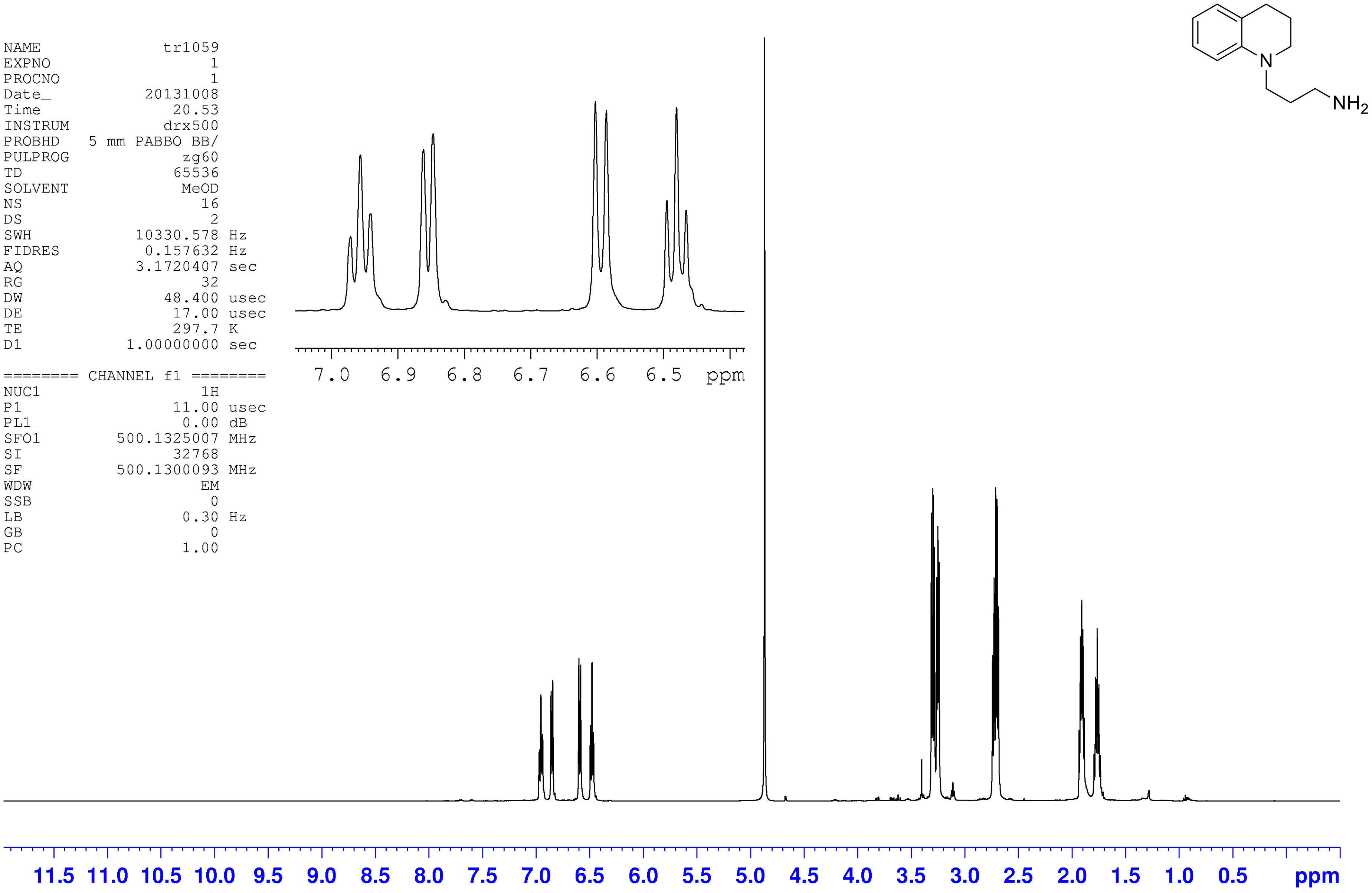

3-(3,4-Dihydroquinolin-1(2*H*)-yl)propan-1-amine **75** ^{13}C NMR

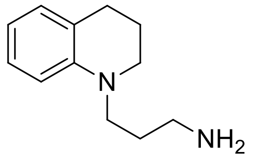


```
NAME              tr1059
EXPNO                  2
PROCNO                 1
Date_           20131008
Time               21.22
INSTRUM           drx500
PROBHD   5 mm PABBO BB/
PULPROG          zgpg30
TD                 65536
SOLVENT             MeOD
NS                   512
DS                     4
SWH            30303.031 Hz
FIDRES          0.462388 Hz
AQ             1.0814105 sec
RG                9195.2
DW                16.500 usec
DE                 11.00 usec
TE                 297.7 K
D1            2.00000000 sec
d11           0.03000000 sec
DELTA         1.89999998 sec
TD0                    1

======== CHANNEL f1 ========
NUC1                 13C
P1                  6.80 usec
PL1                 5.00 dB
SFO1         125.7703148 MHz

======== CHANNEL f2 ========
CPDPRG2          waltz16
NUC2                  1H
PCPD2             100.00 usec
PL2                 0.00 dB
PL12               19.00 dB
PL13               23.00 dB
SFO2         500.1320005 MHz
SI                 32768
SF           125.7575604 MHz
WDW                   EM
SSB                    0
LB                  1.00 Hz
GB                     0
PC                  1.40
```

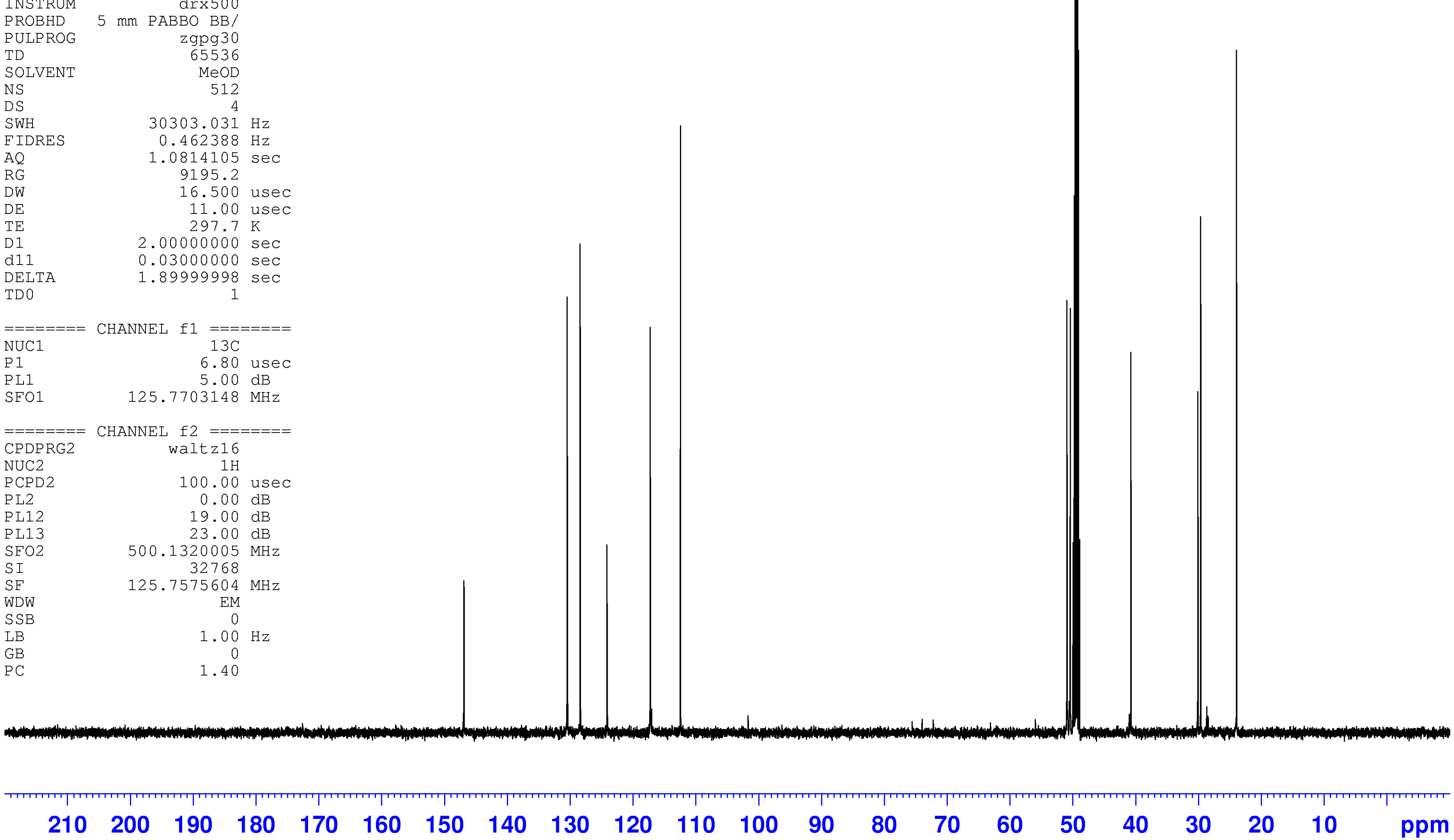

3-(6-Methoxy-3,4-dihydroquinolin-1(2*H*)-yl)propan-1-amine **76** ^{1}H NMR

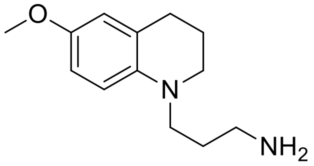


```
NAME              tr3002
EXPNO                  1
PROCNO                 1
Date_           20130625
Time                4.26
INSTRUM           drx500
PROBHD    5 mm PABBO BB/
PULPROG             zg60
TD                 65536
SOLVENT             MeOD
NS                    16
DS                     2
SWH            10330.578 Hz
FIDRES          0.157632 Hz
AQ             3.1720407 sec
RG                  20.2
DW                48.400 usec
DE                 17.00 usec
TE                 297.7 K
D1            1.00000000 sec

======== CHANNEL f1 ========
NUC1                  1H
P1                 11.00 usec
PL1                 0.00 dB
SFO1         500.1325007 MHz
SI                 32768
SF           500.1300000 MHz
WDW                   EM
SSB                    0
LB                  0.30 Hz
GB                     0
PC                  1.00
```

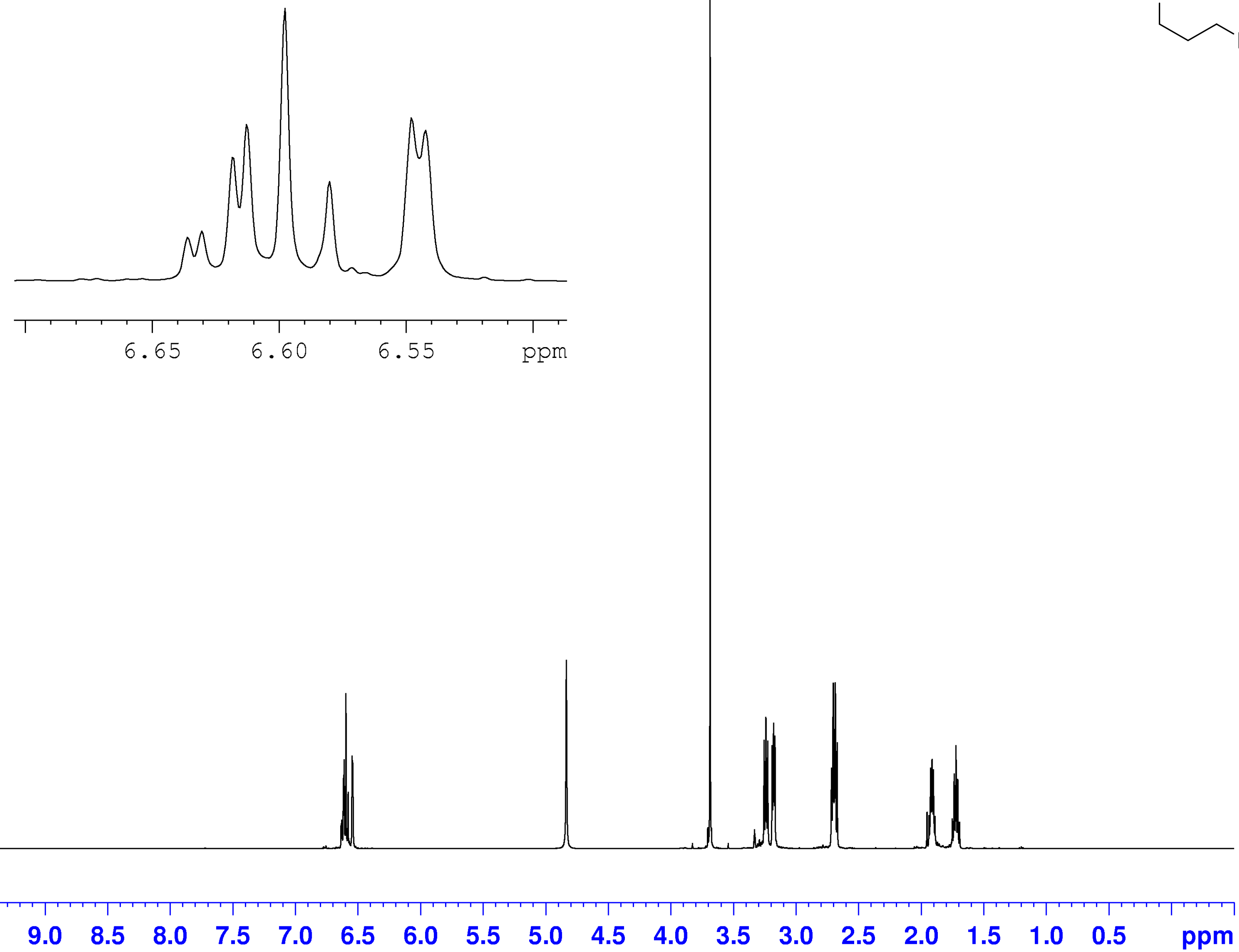

3-(6-Methoxy-3,4-dihydroquinolin-1(2*H*)-yl)propan-1-amine **76** ^{13}C NMR

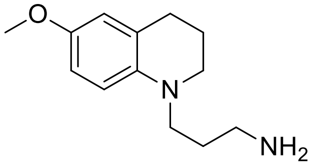

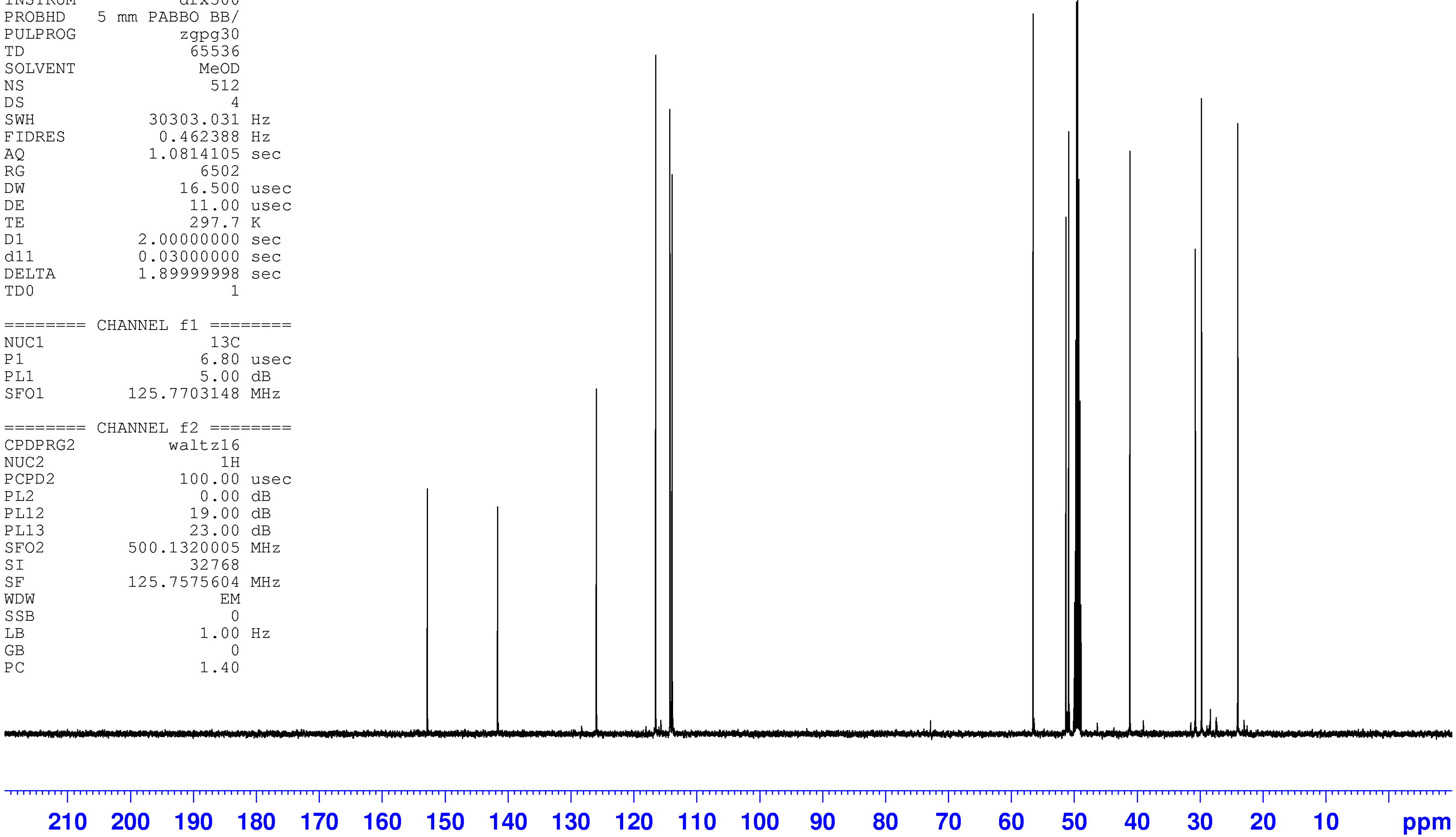

3-(2,3-Dihydro-4*H*-1,4-benzoxazin-4-yl)propan-1-amine **77** ^{1}H NMR

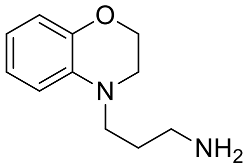


```
NAME            tr3004
EXPNO                1
PROCNO               1
Date_         20131119
Time             13.43
INSTRUM         avg400
PROBHD   5 mm QNP 1H/13
PULPROG           zg30
TD               65536
SOLVENT           MeOD
NS                  16
DS                   2
SWH          10000.000 Hz
FIDRES        0.152588 Hz
AQ           3.2768500 sec
RG              467.29
DW              50.000 usec
DE                6.50 usec
TE               294.3 K
D1          1.00000000 sec
TD0                  1

======== CHANNEL f1 ========
SFO1       400.2024714 MHz
NUC1                1H
P1               12.23 usec
SI               65536
SF         400.2000113 MHz
WDW                 EM
SSB                  0
LB                0.30 Hz
GB                   0
PC                1.00
```

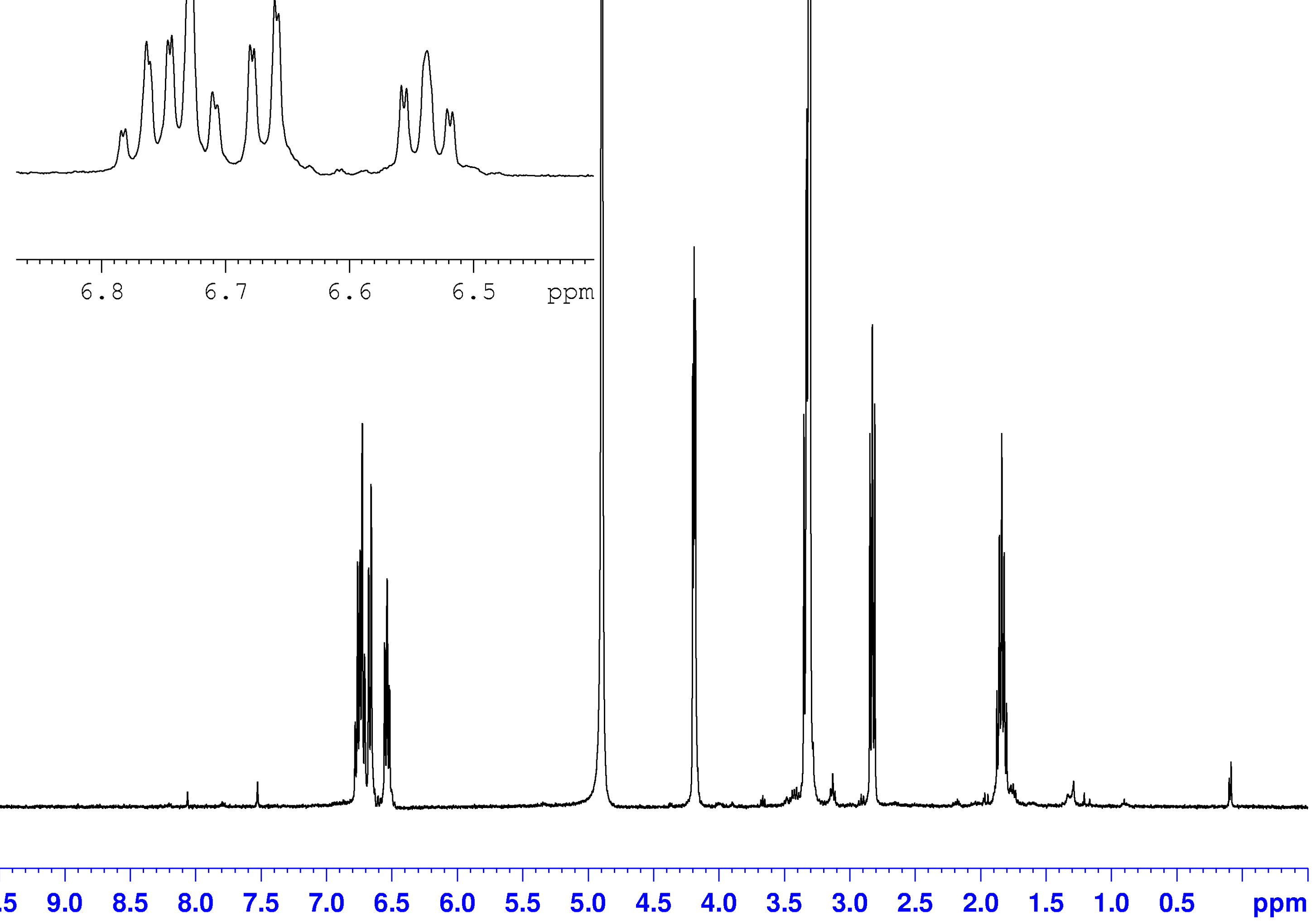

3-(2,3-Dihydro-4*H*-1,4-benzoxazin-4-yl)propan-1-amine **77** ^{13}C NMR

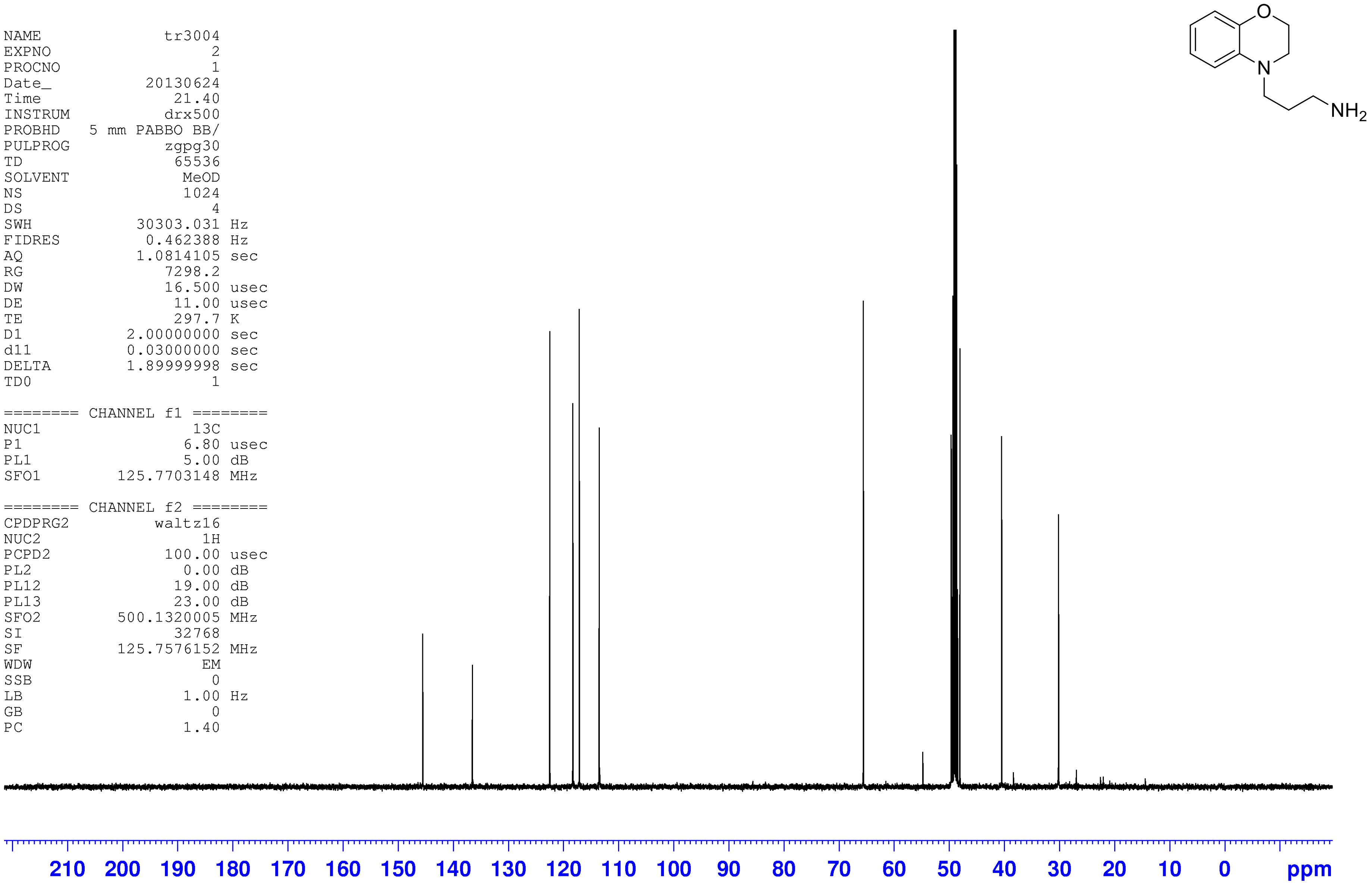

Supplement: Supplementary file 1 — miscellaneous_information [file anie0053-6126-SD1.pdf]
